# Sequential catalysis: exploiting a single rhodium(i) catalyst to promote an alkyne hydroacylation–aryl boronic acid conjugate addition sequence†
†Electronic supplementary information (ESI) available. See DOI: 10.1039/c6sc03066a



**DOI:** 10.1039/c6sc03066a

**Published:** 2016-09-09

**Authors:** Maitane Fernández, Matthias Castaing, Michael C. Willis

**Affiliations:** a Department of Chemistry , University of Oxford , Chemical Research Laboratory , Mansfield Road , Oxford , OX1 3TA , UK . Email: michael.willis@chem.ox.ac.uk

## Abstract

We demonstrate that a single Rh(i) complex can promote two mechanistically distinct C–C bond-forming reactions – alkyne hydroacylation and aryl boronic acid conjugate addition – to deliver substituted ketone products from the controlled assembly of three readily available fragments. This is a rare example of a Rh(i)/Rh(iii) cycle and a redox neutral Rh(i) cycle being promoted by a single catalyst. The process is broad in scope, allowing significant variation of all three reaction components. Incorporation of an enantiomerically pure bis-phosphine ligand renders the process enantioselective. Superior levels of enantioselectivity (up to >99% ee) can be achieved from using a two catalyst system, whereby two Rh(i) complexes, one incorporating an achiral bis-phosphine ligand and the second a chiral diene ligand, are introduced at the start of the reaction sequence.

## Abstract 2

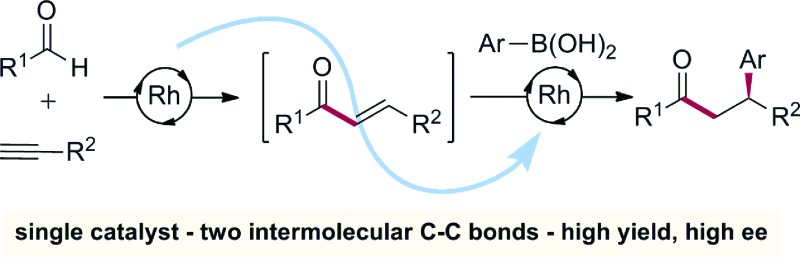
We demonstrate that a single Rh(i) complex can promote two mechanistically distinct C–C bond-forming reactions – alkyne hydroacylation and aryl boronic acid conjugate addition – to deliver substituted ketone products from the controlled assembly of three readily available fragments.

## Introduction

**Scheme 1:**
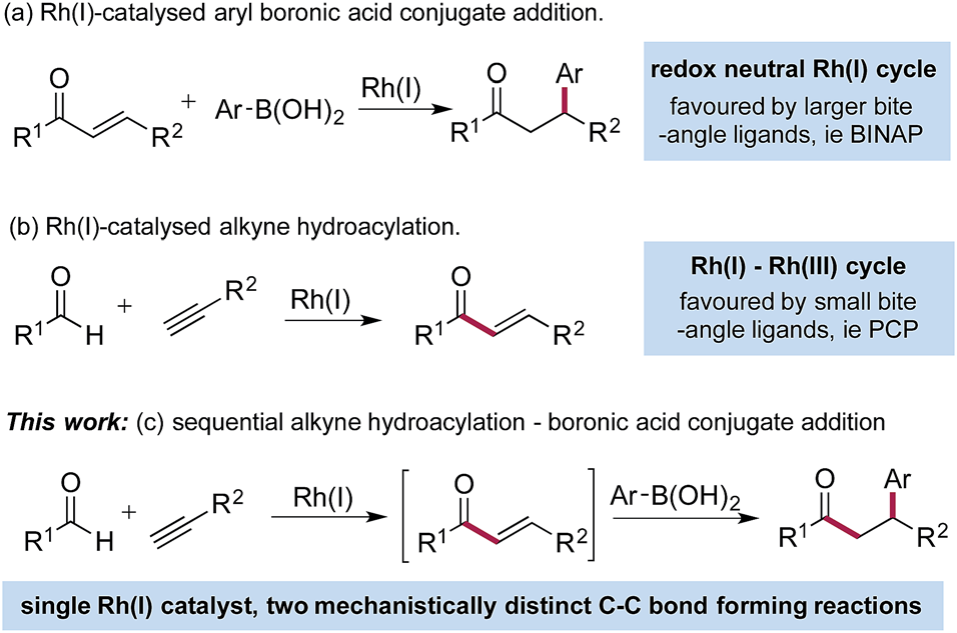
Rh(i)-catalysed boronic acid conjugate additions and alkyne hydroacylation reactions, together with a merged, sequential process.

Since the initial report from Miyaura in 1997,^1^ the Rh(i)-catalysed addition of aryl boronic acids to activated alkenes has become established as a versatile method for the formation of C–C bonds (Scheme 1a).^2^ The variety of activating groups that can be employed on the alkene, the availability of a wide range of boronic acid derivatives and the predictable, often high levels of stereocontrol that can be achieved,^3^ have combined to make these transformations popular choices for synthetic chemists,^4^ including those working in industry.^5^ Although less developed than the conjugate addition chemistry, Rh(i)-catalysed hydroacylation processes are emerging as powerful methods for synthesis.^6^ Alkyne hydroacylation, combining aldehydes with alkynes, is dominated by the use of Rh(i)-catalysts,^7^ allowing the use of mild reaction conditions and low catalyst loadings and represents a potent method for the preparation of enones (Scheme 1b).^8^ The juxtaposition of Rh(i) catalysts in these two processes – alkyne hydroacylation delivering enones as products, and conjugate additions, consuming enones as substrates – although mechanistically distinct, suggested the possibility of merging these two transformations to provide a unique three-component route to substituted, stereodefined ketones (Scheme 1c). Although many examples of single catalysts controlling two bond forming events in a cascade sequence are known,^9^ examples in which two C–C bonds are forged in an intermolecular manner,^10^ using two mechanistically disparate processes, including control of enantioselectivity,^11^ are extremely rare: this contribution documents such a process.

The Rh(i)-catalysed addition of aryl boronic acids to electron-poor alkenes is a redox neutral process which most commonly employs catalysts based on relatively large bite-angle bis-phosphine ligands such as BINAP.^1,4,12^ Conversely, Rh(i)-catalysed alkyne hydroacylation reactions involve a Rh(i)/Rh(iii) cycle, and often employ complexes based on small bite-angle bis-phosphines.^13^ The key to developing the proposed sequential catalytic alkyne hydroacylation–boronic acid conjugate addition sequence would be to identify a rhodium complex capable of mediating both of these mechanistically distinct processes in an efficient manner.

## Results and discussion

We began our study by exploring the combination of 2-aminobenzaldehyde **1a** and 1-octyne, followed by the addition of phenyl boronic acid (Table 1). This sequence delivers β-phenyl substituted *o*-amino-ketone **2a** as the product; *o*-amino-ketones such as this are useful synthetic units in their own right,^14^ and are also embedded in a variety of important heterocycles.^15^ We evaluated a range of bis-phosphine ligands in the proposed hydroacylation reaction and the results were comparable to our previous studies with amine-chelating aldehydes,^16^ with the smallest bite-angle dcpm and dppm bis-phosphines (entries 1 and 2), as well as dppe (entry 4), generating highly efficient catalysts. Increasing the bite angle further, as in the case of dppp, resulted in a poorly active hydroacylation catalyst (entry 5). As suggested from the literature,^1^ of the ligands successful in hydroacylation, only dppe, with a wider bite angle, was able to subsequently promote the conjugate addition, allowing for successful one-pot, two intermolecular C–C bond formation, to occur (entry 4).

**Table 1:** Ligand evaluation for the sequential combination of aldehyde **1a**, 1-octyne and phenyl boronic acid^*a*^

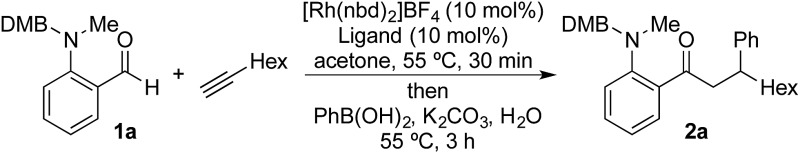				
Entry	Ligand	HA conv^*b*^. (%)	CA conv^*b*^. (%)	Yield (%)
1	dcpm	100	<5	—
2	dppm	100	10	—
3	dcpe	6	—	—
4	dppe	100	100	84
5	dppp	5	—	—
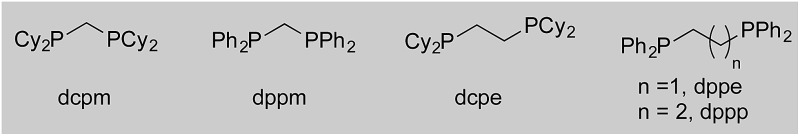				

^*a*^Reaction conditions: **1a** (1.0 equiv.), 1-octyne (1.3 equiv.), [Rh(nbd)_2_]BF_4_ (10 mol%), ligand (10 mol%), acetone, 55 °C, 30 min; then PhB(OH)_2_ (2.0 equiv.), K_2_CO_3_ (0.2 equiv.), acetone/water, 3 h. Isolated yield.

^*b*^Determined by ^1^H NMR spectroscopy. DMB = 3,4-dimethoxybenzyl.

We next explored the scope of the three-component transformation (Table 2), and for operational simplicity we used a pre-formed catalyst ([Rh(dppe)(C_6_H_5_F)]BAr^F^).^17^ In general, the developed reaction was very broad in scope, allowing excellent variation of all three components. A wide range of aryl boronic acids could be employed successfully, including substitution at all three positions of the phenyl ring, and a variety of electronically varied functional groups (**2a–n**). The use of heterocyclic (**2o–p**), 1- and 2-naphthyl (**2q–2r**) and several alkenyl boronic acids (**2s–2u**) was also compatible with the process, delivering the final products in good yields. 2-Aminobenzaldehydes with various substituents on the amine could be employed, in all cases obtaining the final β-substituted ketones in very high yields (products **2v–y**). Additionally, electronically varied substituents on the aromatic core of the aldehydes were also allowed (**2z–2ad**).

**Table 2:** Scope of achiral sequential alkyne hydroacylation – conjugate addition process^*a*^

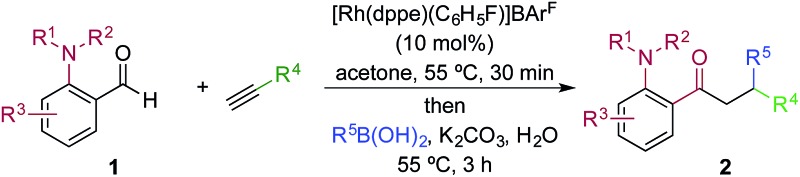
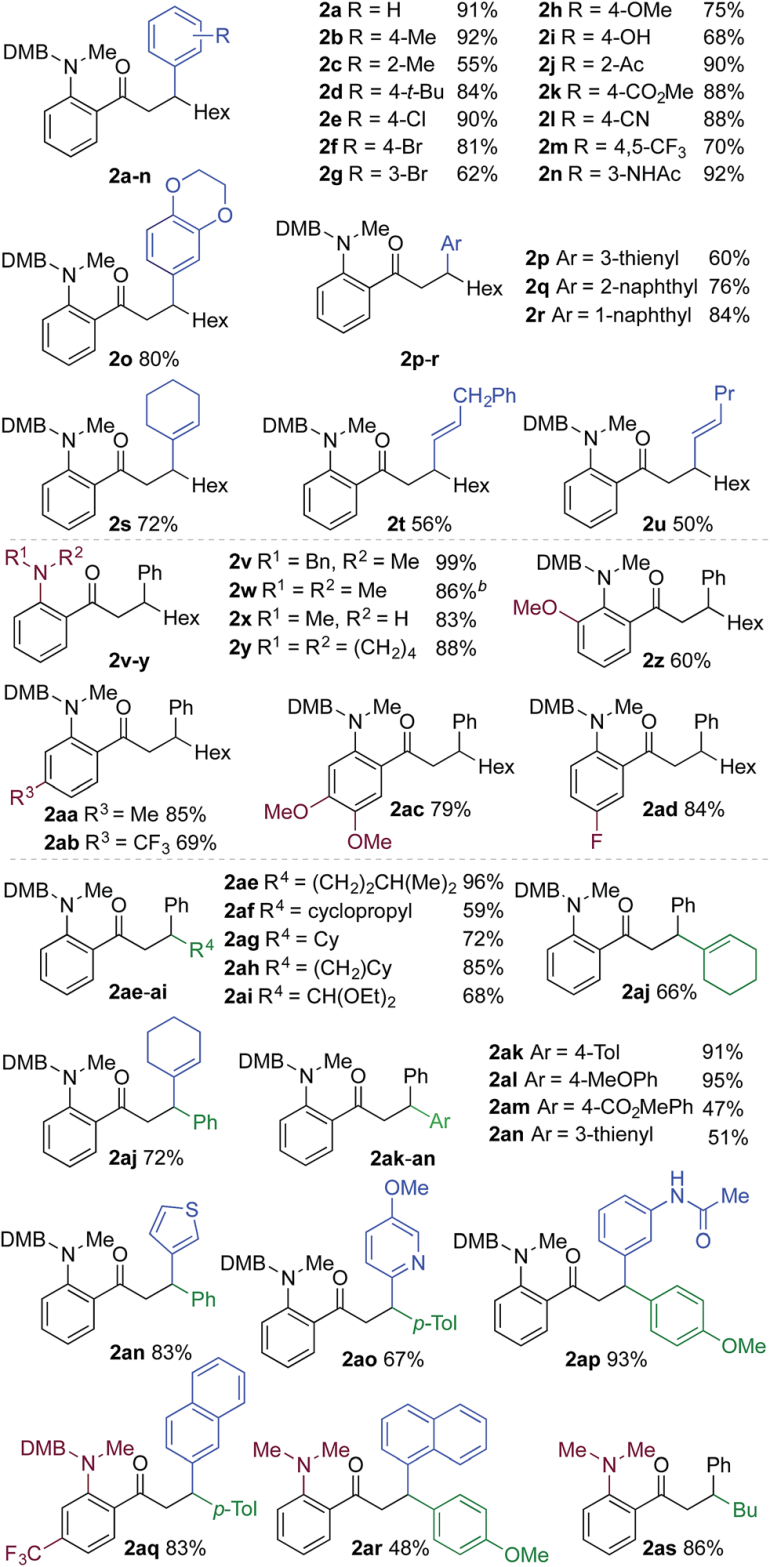

^*a*^Reaction conditions: **1** (0.20 mmol), alkyne (0.26 mmol), [Rh(dppe)(C_6_H_5_F)]BAr^F^ (10 mol%), acetone, 55 °C, 30 min; then boronic acid (0.40 mmol), K_2_CO_3_ (0.04 mmol), acetone/water, 3 h. Isolated yields.

^*b*^97% yield on a 3 mmol scale, using 5 mol% Rh catalyst.

With respect to the alkyne, again, wide variation was possible, including the use of alkyl chains, carbocycles, acetals and aromatic groups (**2ae–2as**). Several examples in Table 2 show variation of more than one component from the standard reaction (**2an–2as**, **2aj**), and give an indication of the structural range accessible using the developed chemistry. Ketones **2aj** and **2an** were prepared using both possible combinations of alkyne and boronic acid, demonstrating the flexibility of the approach to adapt to available feedstocks. Larger scale reactions were also possible; using 5 mol% of Rh, a 3 mmol scale experiment returned 1 gram of ketone **2w** in a 97% yield.

Having identified an achiral Rh-complex capable of delivering a hydroacylation-conjugate addition sequence of broad scope, our next task was to identify a chiral catalyst that would provide enantiomerically enriched products. We evaluated the performance of a series of chiral bis-phosphine ligands in our reaction (Table 3), mindful that the PCCP scaffold was the most efficient for the achiral reaction. Although the highest enantioselectivity was achieved with Chiraphos (86% ee), MeDuphos provided the best all round performance, delivering the ketone **2a** in reasonable-good yield and ee (76% yield, 78% ee).

**Table 3:** Chiral ligand evaluation for the **1a** → **2a** reaction sequence^*a*^

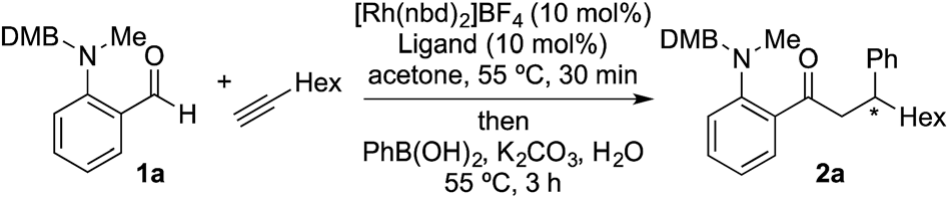
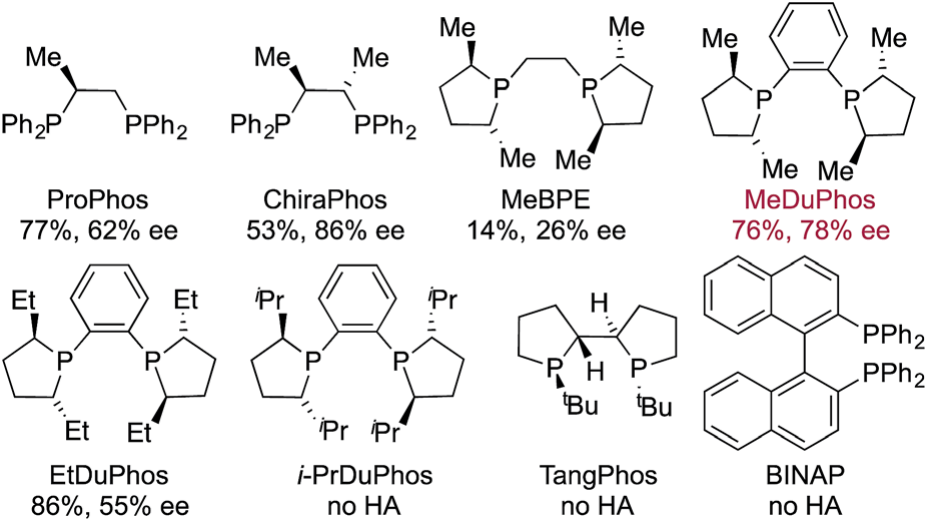

^*a*^Reaction conditions: **1a** (1.0 equiv.), 1-octyne (1.3 equiv.), [Rh(nbd)_2_]BF_4_ (10 mol%), ligand (10 mol%), acetone, 55 °C, 30 min; then PhB(OH)_2_ (2.0 equiv.), K_2_CO_3_ (0.2 equiv.), acetone/water, 3 h. Isolated yields. ees determined by chiral HPLC.

Using a MeDuPhos-derived catalyst, we investigated if variation of the substrate would have an impact on enantioselectivity (Table 4). Overall, the reactions delivered the product ketones in high to excellent yields; however, the enantioselectives were broadly consistent with the trial system and remained in the 75–86% ee region. The exception was the use of aryl-substituted alkynes, which led to a significant reduction in ee (**2ak**, **2an**).

**Table 4:** Sequential hydroacylation – conjugate addition reactions employing a MeDuPhos–Rh(i) catalyst^*a*^

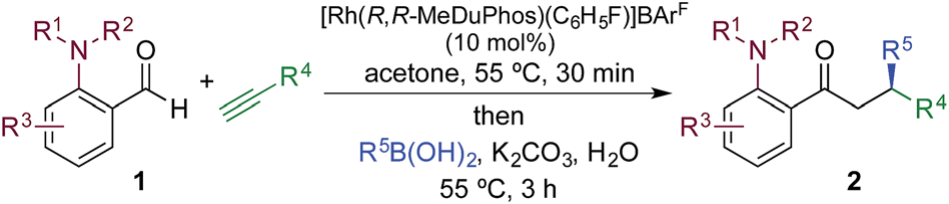
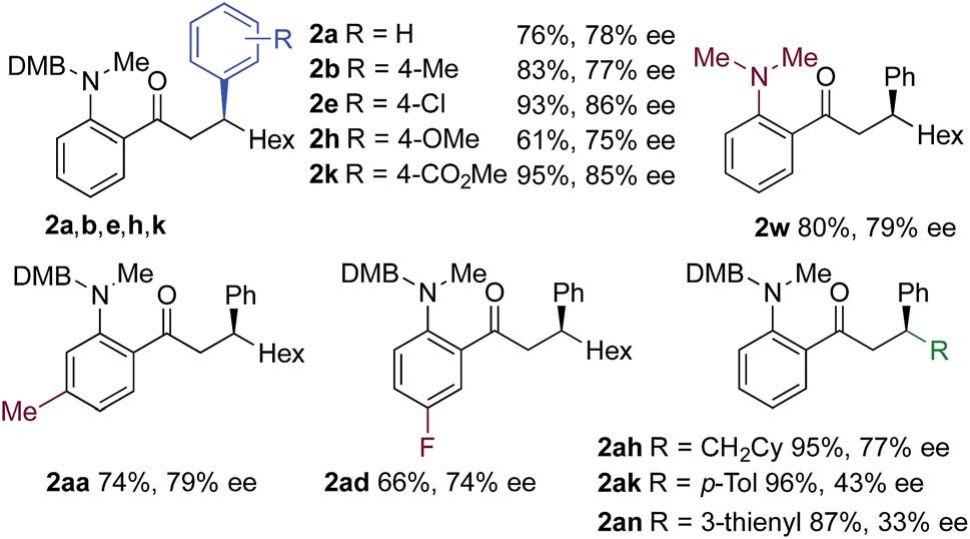

^*a*^Reaction conditions: **1** (0.20 mmol), alkyne (0.26 mmol), [Rh(*R*,*R*-MeDuPhos)(C_6_H_5_F)]BAr^F^ (10 mol%), acetone, 55 °C, 30 min; then boronic acid (0.40 mmol), K_2_CO_3_ (0.04 mmol), acetone/water, 3 h. Isolated yields. ees determined by chiral HPLC.

**Scheme 2:**
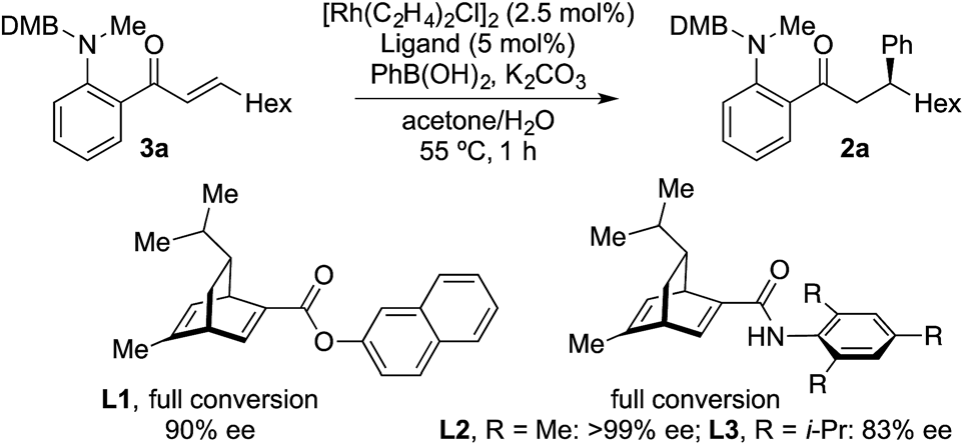
The use of chiral diene ligands in the conjugate addition to enone **3a**.

The examples in Table 4 show that it is possible for a single Rh-complex to catalyze two distinct intermolecular C–C bond-forming reactions, delivering products with high, but not excellent, enantioselectivity. While we were confident that evaluating further chiral phosphines would deliver a more selective catalyst, we reasoned that a more expedient approach would be to explore the use of a two catalyst system, where one catalyst is optimized for the hydroacylation step, and the second is tailored to deliver an efficient and highly enantioselective conjugate addition. For practicality it would be ideal if both catalysts were present in the reaction vessel from the start. From Table 1, and earlier reports,^16^ we were confident that a dcpm-supported catalyst would be efficient for alkyne hydroacylation. For the conjugate addition step we turned our attention to the use of chiral diene ligands,^18^ and chose to evaluate three ligands in a model system involving the addition of phenyl boronic acid to enone **3a** (Scheme 2). All three ligands provided efficient reactions. Although all three ligands also delivered levels of enantiocontrol that surpassed the results achieved using MeDuPhos, ligand **L2**, developed by Lam,^19^ was the stand-out choice, delivering ketone **2a** in >99% ee. All three diene ligands generated inactive hydroacylation catalysts.

We next explored the use of a two-catalyst system based on dcpm and chiral diene **L2**. For pragmatic reasons we used two preformed catalysts, [Rh(dcpm)(C_6_H_5_F)]BAr^F^ and [Rh(**L2**)(CH_3_CN)_2_]BAr^F^, which allowed the addition of both complexes at the start of the reaction. Pleasingly, using this approach we were able to obtain the desired β-phenylketone **2a** in 87% yield with an excellent 96% ee (Table 5). We explored the scope of this asymmetric process, and similar to the non-enantioselective variant, the reaction was broad in scope, allowing wide variation of the three components and providing the desired products in good yields and with excellent enantioselectivities (Table 5). A broad range of aryl boronic acids were successfully used, including those bearing substituents with different steric and electronic properties (products **2a–2n**), as well as examples of heteroaromatic (**2o–2p**), naphthyl (**2q–2r**) and alkenyl boronic acids (**2s**). Aldehydes with different chelating groups, or substituents on the aromatic core were tolerated (products **2v–2ad**), as were various alkyne reaction partners (**2ae–2as**). In particular, the use of ethynylbenzene derivatives offered very high levels of enantiocontrol, significantly improving the performance of several boronic acids that had shown only moderate selectivity when combined with 1-octyne (see **2aj** *vs.* **2s** and **2ap** *vs.* **2n**). Finally, the ability to synthesize both enantiomers of the target ketones by simply reversing the combination of alkyne and boronic acid, for example ketone **2an**, is a powerful feature of the developed sequence, significantly expanding the utility of the process.

**Table 5:** The use of a two-catalyst system for sequential enantioselective alkyne hydrocylation–boronic acid conjugate addition^*a*^

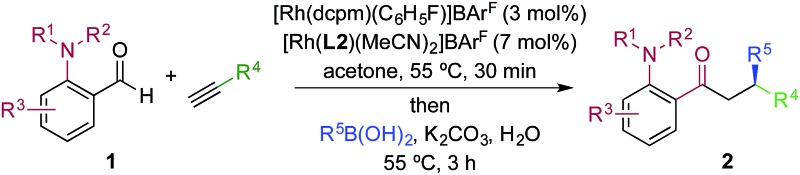
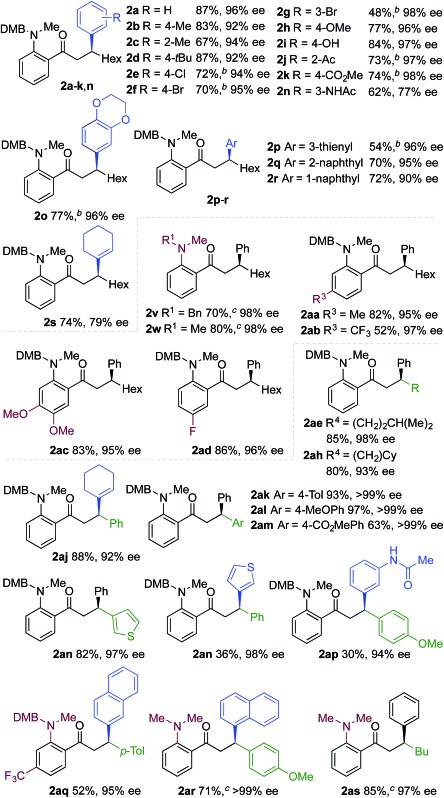

^*a*^Reaction conditions: **1** (0.20 mmol), alkyne (0.26 mmol), [Rh(dcpm)(C_6_H_5_F)]BAr^F^ (3 mol%), [Rh(**L2**)(MeCN)_2_]BAr^F^ (7 mol%), acetone, 55 °C, 30 min; then boronic acid (0.40 mmol), K_2_CO_3_ (0.04 mmol), acetone/water, 3 h. Isolated yields. ees determined by chiral HPLC.

^*b*^In DCE.

^*c*^0.80 mmol boronic acid.

## Conclusions

We have shown that a dppe–Rh(i) complex can catalyze sequential alkyne hydroacylation and boronic acid conjugate additions to provide β-substituted ketones with high efficiency. This sequence is a rare example of a single catalyst mediating two distinct intermolecular C–C bond-forming reactions. Use of a MeDuPhos-derived catalyst renders the process enantioselective, however, the highest selectivities are obtained using a two-catalyst system involving a chiral diene ligand, delivering ketones with excellent enantioselectvities.
