# Supplementary material for: Sequential catalysis: exploiting a single rhodium(i) catalyst to promote an alkyne hydroacylation–aryl boronic acid conjugate addition sequence
Source: Chem Sci. 2016 Sep 9;8(1):536–40. doi: 10.1039/c6sc03066a (PMC5351800; doi:10.1039/c6sc03066a)
Supplement: Supplementary file 1 [file SC-008-C6SC03066A-s001.pdf]

Electronic Supplementary Material (ESI) for Chemical Science.
This journal is © The Royal Society of Chemistry 2016

Sequential Catalysis: Exploiting a Single Rhodium(I) Catalyst to Promote an Alkyne Hydroacylation-Aryl Boronic Acid Conjugate Addition Sequence

Maitane Fernández, Matthias Castaing and Michael C. Willis*

*Department of Chemistry, University of Oxford

Chemistry Research Laboratory

Mansfield Road, OX1 1TA, UK

Email: michael.willis@chem.ox.ac.uk

Homepage: http://mcwillis.chem.ox.ac.uk/MCW/Home.html

Supporting Information

General Experimental Methods	page S2
Preparation of Rhodium Complexes	page S3
Preparation of new Aldehydes	page S4
Hydroacylation/Conjugate Addition: Characterization of new Compounds	page S5
Determination of Absolute Configuration	page S32
NMR spectra for new organic compounds	page S35
HPLC traces	page S85

General Experimental Methods

Reactions were performed under inert atmosphere of nitrogen with anhydrous solvent unless otherwise stated. All glassware was oven dried at >80 °C, and allowed to cool to room temperature under a positive nitrogen pressure. Reactions were monitored by TLC until deemed complete using aluminum backed silica plates. Plates were visualized under ultraviolet light and/or by staining with vanillin, *p*-anisaldehyde, phosphomolibdic acid or $KMnO_4$ stains.[1]

Reagents were purchased from Sigma-Aldrich Chemical Co. Ltd., Alfa Aesar, Acros Organics Ltd., Lancaster Synthesis Ltd, or Strem Chemicals Inc. and were used as supplied. Acetone was distilled from Drierite®. Dichloroethane and Fluorobenzene were distilled from calcium hydride. Petrol refers to the fractions obtained between 40 and 60 °C. Ether refers to diethyl ether. Flash chromatography was carried out using matrix 60 silica. $[Rh(COD)_2][BAr^F_4]$ (COD = 1,5-Cyclooctadiene),[2] $[Rh(dppe)(C_6H_5F)][BAr^F_4]$ (dppe = bis(diphenylphosphino)ethane)[3] and $[Rh(dcpm)(C_6H_5F)][BAr^F_4]$ (dcpm = bis(dicylohexylphosphino)methane)[4] were prepared using literature methods. Alkynes were distilled prior to use. Aldehydes were prepared according to literature procedures,[5] unless otherwise stated.

^{1}H NMR spectra were obtained on Bruker AVIII400 (400 MHz) or Bruker AVII500 (500 MHz) spectrometers using the residual solvent as an internal standard. ^{13}C NMR spectra were obtained on Bruker AVIII400 (100 MHz) or Bruker AVII500 (125 MHz) spectrometer using the residual solvent as an internal standard. Chemical shifts were reported in parts per million (ppm) with the multiplicities of the spectra reported as following: s, singlet; d, doublet; t, triplet; q, quartet; m, multiplet; b, broad. Low-resolution ESI mass spectra were recorded on a Waters LCT Premier spectrometer. High-resolution ESI mass spectrometry measurements were recorded on a Bruker Daltronics microTOF (ESI) spectrometer by the internal service at the Department of Organic Chemistry, University of Oxford. Infrared spectra were recorded as thin films on a Bruker Tensor 27 FT-IR spectrometer. Melting points were determined using a Stuart Scientific Melting Point Apparatus SMP1. Optical rotations were measured on a Schmidt Haensch UniPol L2000 polarimeter. The enantiomeric excess (ee) of the products was determined by chiral stationary phase HPLC in a Dionex P680 chromatograph with a Dionex UVD170U detector (Daicel Chiralpak AD-H, AS-H, IC, IA-3 and ID-3 columns).

[1] Stahl, E. *Thin Layer Chromatography*, Springer-Verlag, Berlin, **1969**.
[2] Guzel, B.; Omary, M. A.; Fackler, J. P.; Akgerman, A. *Inorg. Chim. Acta* **2001**, *325*, 45.
[3] Dallanegra, A.; Robertson, A. P. M.; Chaplin, A. B.; Manners, I.; Weller, A. S. *Chem. Commun.* **2011**, *47*, 3763.
[4] Chaplin, A. B.; Hooper, J. F.; Weller, A. S.; Willis, M. C. *J. Am. Chem. Soc.* **2012**, *134*, 4885.
[5] Castaing, M.; Wason, S. L.; Estepa, B.; Hooper, J. F.; Willis, M. C. *Angew. Chem. Int. Ed.* **2013**, *52*, 13280.

Preparation of Rhodium Complexes

Preparation of [Rh(R,R-MeDuPhos)(C_6H_5F)][BAr^F_4]

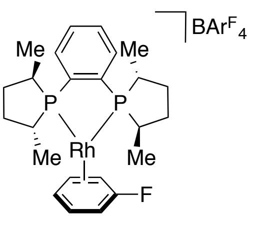

To a Schlenk flask charged with a solution of [Rh(COD)$_2$][BAr^F_4] (374 mg, 0.32 mmol) in C_6H_5F (1 mL) at -30 ºC, was added a solution of (R,R)-Me-DuPhos (97 mg, 0.32 mmol) in C_6H_5F (2 mL). The resulting solution was allowed to warm to room temperature prior to be placed under H_2 (1 atm) and it was then stirred at room temperature for 3 hours. The product was precipitated by addition of pentane. The resulting yellow solid was filtered *via* cannula, washed with more pentane and dried under vacuum. Yield: 82% (361 mg, 0.26 mmol). **^{1}H NMR** (500 MHz, CD_2Cl_2): δ 7.80-7.76 (bs, 8H), 7.64-7.56 (m, 8H), 7.03-6.99 (m, 1H), 6.97-6.93 (m, 1H), 6.90-6.87 (m, 1H), 6.83-6.79 (m, 1H), 6.10-6.06 (m, 1H), 2.61-2.50 (m, 2H), 2.48-2.15 (m, 6H), 1.80-1.68 (m, 2H), 1.54-1.42 (m, 2H), 1.28-1.20 (app. dd, J = 19.2, 7.0 Hz, 6H), 0.87-0.79 (app. dd, J = 15.9, 7.0 Hz, 6H); **^{13}C NMR** (126 MHz, CD_2Cl_2): δ 161.7 (q, $^1J_{BC}$ = 49.7 Hz, CB_{BArF4}), 141.9 (dd, $^1J_{FC}$ = 269.8 Hz, J_{RhC} = 2.9 Hz, i-C_6H_5F), 141.4 (app. td, $^1J_{PC}$ = 40.3 Hz, $^2J_{PC}$ = 5.7 Hz, C_{Ph}), 134.8 (s, CH_{BArF4}), 131.6 (app. t, $^3J_{PC}$ = 5.7 Hz, CH_{Ph}), 131.1 (app. td, $^2J_{PC}$ = 9.6 Hz, $^3J_{PC}$ = 1.7 Hz, C_{Ph}), 128.8 (qq, $^2J_{FC}$ = 31.5 Hz, $^3J_{BC}$ = 2.8 Hz, $CCF3_{BArF4}$), 124.6 (q, $^1J_{FC}$ = 272.3 Hz, $CF3_{BArF4}$), 117.4 (sept, $^3J_{FC}$ = 3.9 Hz, CH_{BArF4}), 101.4 (dd, $^3J_{FC}$ = 7.3 Hz, J_{RhC} = 2.7 Hz, m-C_6H_5F), 100.3 (dd, $^3J_{FC}$ = 7.6 Hz, J_{RhC} = 2.5 Hz, m-C_6H_5F), 93.3 (d, $^2J_{FC}$ = 20.2 Hz, o-C_6H_5F), 92.3 (d, J_{RhC} = 2.6 Hz, p-C_6H_5F), 91.9 (d, $^2J_{FC}$ = 20.2 Hz, o-C_6H_5F), 46.5 (m, CHP), 40.0 (m, CHP), 36.2 (s, CH_2), 35.6 (app. t, $^2J_{PC}$ and $^3J_{RhC}$ = 2.5 Hz, CH_2), 18.3 (app. t, $^2J_{PC}$ and $^3J_{RhC}$ = 3.9 Hz, Me), 13.0 (s, Me); **^{31}P-NMR** (162 MHz, CD_2Cl_2) δ 98.9 (dd, $^1J_{RhP}$ = 200.7 Hz, $^3J_{PP}$ = 2.7 Hz); **^{19}F-NMR** (377 MHz, CD_2Cl_2) δ **-** 62.8 (s, 24F), **-**123.0 (s, 1F).

Preparation of [Rh(L2)Cl]$_2$

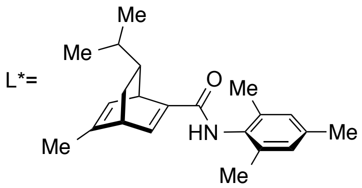

A round bottomed flask was charged with [Rh(C_2H_4)$_2$Cl]$_2$ (428 mg, 1.1 mmol, 2.2 mmol Rh) and the chiral ligand **L2** (647 mg, 2.0 mmol), and these were dissolved in DCM (50 mL). The resulting solution was stirred at room temperature for 3h. The mixture was filtered through a pad of Celite® and washed with DCM. The filtrates were concentrated *in vacuo* to provide an orange solid that did not require further purification. Yield: 96% (981 mg, 1.06 mmol). **^{1}H NMR** (500 MHz, $CDCl_3$): δ 7.35 (bs, 1H), 6.93-6.66 (m, 2H), 4.69-4.48 (m, 1H), 4.26-4.02 (bs, 1H), 3.84 (bs, 1H), 3.47-2.98 (m, 1H), 2.37-1.99 (m, 9H), 1.56 (s, 3H), 1.36 (ddd, J = 13.3, 9.9, 3.2 Hz, 1H), 1.27-1.06 (m, 2H), 0.98-0.67 (m, 7H); **^{13}C NMR** (126 MHz, $CDCl_3$): δ 168.6 (s, NHCO), 136.6 (s, CMe_{Mes}), 135.0 (bs, CMe_{Mes}), 131.1 (bs, CMe_{Mes}), 128.8 (s, CH_{Mes}), 73.9 (m, COC=CH), 58.1 (m, MeC=CH), 51.9 (m, COC=CH), 49.5 (m, MeC=CH), 48.4 (bs, MeCCH), 46.0 (bs, COCCH), 45.8 (s, CH_2CH^iPr), 31.0 (s, CH_2), 30.8 (s, CH_{iPr}), 21.4 (bs, Me), 20.9 (s, Me_{iPr}), 20.7 (s, Me_{iPr}), 20.5 (bs,

Me$_{Mes}$), 18.8 (bs, *Me*$_{Mes}$); **MS** (ESI$^+$) *m/z* (%) 426 (45), 887 (100), 889 (43); **MS** (ESI$^-$) *m/z* (%) 496 (100), 497 (62), 498 (27), 959 (12); **HRMS** (ESI$^+$) calc. for $C_{44}H_{58}O_2N_2ClRh_2$ (M-Cl)$^+$: 887.22914, found: 887.22943.

Preparation of [Rh(L2)(MeCN)$_2$][BAr^F$_4$]

To a Schlenk flask charged with [Rh(L2)Cl]$_2$ (231 mg, 0.25 mmol, 0.5 mmol Rh) and NaBAr^F$_4$ (443 mg, 0.50 mmol) were added DCM (12 mL) and acetonitrile (0.5 mL). The solution was stirred at room temperature overnight. The resulting solution was filter-cannulated into another schlenk and the majority of the solvent was removed under vacuum. The product was precipitated by addition of pentane. The resulting yellow solid was filtered *via* cannula, washed with more pentane and dried under vacuum. Yield: 82% (559 mg, 0.41 mmol). **^{1}H NMR** (500 MHz, $CDCl_3$): δ 7.70-7.66 (bs, 8H), 7.54 (s, 4H), 7.46 (bs, 1H), 6.91 (s, 2H), 4.51 (d, *J* = 6.0 Hz, 1H), 4.47 (d, *J* = 4.9 Hz, 1H), 4.21-4.19 (m, 1H), 3.66 (d, *J* = 6.0 Hz, 1H), 2.28 (s, 3H), 2.19 (s, 6H), 2.03 (s, 6H), 1.56 (s, 3H), 1.49-1.42 (m, 1H), 1.30 (dq, *J* = 13.7, 6.4 Hz, 1H), 0.98 (q, *J* = 7.6 Hz, 1H), 0.93 (d, *J* = 6.6 Hz, 3H), 0.86-0.78 (m, 4H); **^{13}C NMR** (126 MHz, CD_2Cl_2): δ 164.6 (s, NH*C*O), 161.7 (q, $^1J_{BC}$ = 49.9 Hz, *C*B$_{BArF4}$), 137.9 (s, *C*Me$_{Mes}$), 134.7 (s, *C*H$_{BArF4}$), 134.4 (s, *C*Me$_{Mes}$), 130.2 (s, *C*Me$_{Mes}$), 129.3 (s, *C*H$_{Mes}$), 128.9 (qq, $^2J_{FC}$ = 31.5 Hz, $^3J_{BC}$ = 2.9 Hz, *C*CF3$_{BArF4}$), 124.6 (q, $^1J_{FC}$ = 272.6 Hz, *C*F3$_{BArF4}$), 122.9 (bs, Me*C*N), 117.5 (sept, $^3J_{FC}$ = 3.8 Hz, *C*H$_{BArF4}$), 83.8 (d, $^1J_{RhC}$ = 9.1 Hz, CO*C*=CH), 64.3 (d, $^1J_{RhC}$ = 10.8 Hz, Me*C*=CH), 60.6 (d, $^1J_{RhC}$ = 9.7 Hz, COC=*C*H), 59.0 (d, $^1J_{RhC}$ = 9.3 Hz, MeC=*C*H), 48.1 (d, $^2J_{RhC}$ = 2.0 Hz, MeC*C*H), 47.6 (s, CH_2C*H*^{i}Pr), 46.3 (d, $^2J_{RhC}$ = 2.1 Hz, COC*C*H), 31.0 (s, *C*H$_2$), 30.5 (s, *C*H$_{iPr}$), 20.9 (s, *Me*), 20.8 (s, *Me*$_{Mes}$), 20.7 (s, *Me*$_{iPr}$), 20.6 (s, *Me*$_{iPr}$), 18.1 (s, *Me*$_{Mes}$), 2.4 (s, *Me*$_{MeCN}$); **^{19}F-NMR** (377 MHz, $CDCl_3$) *δ* -62.3 (s, 24F); **MS** (ESI$^+$) *m/z* (%) 324 (100), 426 (52), 508 (3), 897 (6), 979 (6); **MS** (ESI$^-$) *m/z* (%) 862 (20), 863 (100), 864 (33); **HRMS** (ESI$^-$) calc. for $C_{32}H_{12}{}^{11}BF_{24}$ (M)$^-$: 863.06543, found: 863.06148.

Preparation of new Aldehydes

2-[(3,4-Dimethoxybenzyl)(methyl)amino]-4-methylbenzaldehyde (1g)

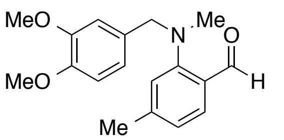

Prepared following a procedure adapted from Willis et al.[5] Potassium carbonate (1.02 g, 7.4 mmol) and 3,4-dimethoxy-*N*-methylbenzylamine (1.34 g, 7.4 mmol) were added to a previously backfilled flask containing 2-fluoro-4-methylbenzaldehyde (0.86 mL, 5.9 mmol) in DMF (6 mL). The solution was heated to 85 ºC under N_2 for 48 hours. The reaction was cooled down to room temperature and quenched with a saturated aqueous solution of potassium carbonate (15 mL). The water phase was extracted with DCM (3 x 15 mL) and the combined organic layers were washed with a saturated solution of lithium chloride (3 x 15 mL), and then dried over magnesium sulfate. The solvent was removed *in vacuo*, and

flash chromatography (gradient petrol:ether 4:1 to 1:1) afforded the title compound as a yellow solid in 35% yield (627 mg, 2.1 mmol). **^{1}H-NMR** (400 MHz, $CDCl_3$) δ 10.32 (s, 1H), 7.72 (d, J = 8.2 Hz, 1H), 6.89-6.87 (m, 2H), 6.81 (s, 2H), 6.74 (s, 1H), 4.25 (s, 2H), 3.86 (s, 3H), 3.79 (s, 3H), 2.76 (s, 3H), 2.35 (s, 3H); **^{13}C-NMR** (100 MHz, $CDCl_3$) δ 190.8, 155.7, 149.0, 148.3, 145.8, 130.5, 130.0, 125.8, 122.7, 120.3, 120.1, 111.0, 110.9, 62.2, 55.9, 55.8, 41.9, 22.1; **IR** (film, cm^{-1}) 2955, 2835, 1677, 1602, 1514, 1262, 1028, 807; **MS** (ESI^+) *m/z* (%) 151 (14), 300 (100), 301 (18), 621 (4); **HRMS** (ESI^+) calc. for $C_{18}H_{22}O_3N$ $(M+H)^+$: 300.15942, found: 300.15986. **m.p.** (ºC): 49-51.

2-[(3,4-Dimethoxybenzyl)(methyl)amino]-5-fluorobenzaldehyde (1j)

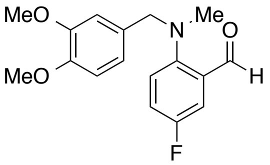

Prepared following a procedure adapted from Willis et al.[5] Potassium carbonate (1.80 g, 13.0 mmol) and 3,4-dimethoxy-*N*-methylbenzylamine (2.36 g, 20.0 mmol) were added to a previously backfilled flask containing 2,5-difluorobenzaldehyde (1.13 mL, 10.4 mmol) in DMF (10 mL). The solution was heated to 85 ºC under N_2 for 48 hours. The reaction was cooled down to room temperature and quenched with a saturated aqueous solution of potassium carbonate (20 mL). The water phase was extracted with DCM (3 x 20 mL) and the combined organic layers were washed with a saturated solution of lithium chloride (3 x 20 mL), and then dried over magnesium sulfate. The solvent was removed *in vacuo*, and flash chromatography (gradient petrol:ether 3:2 to 1:1) afforded the title compound as a yellow solid in 20% yield (628 mg, 2.1 mmol). **^{1}H-NMR** (400 MHz, $CDCl_3$) δ 10.43 (d, J = 2.9 Hz, 1H), 7.49 (dd, J = 8.5, 3.1 Hz, 1H), 7.20 (app. td, J = 8.3, 3.1 Hz, 1H), 7.10 (dd, J = 8.9, 4.4 Hz, 1H), 6.81-6.76 (m, 2H), 6.70 (s, 1H), 4.17 (s, 2H), 3.86 (s, 3H), 3.80 (s, 3H), 2.76 (s, 3H); **^{13}C-NMR** (100 MHz, $CDCl_3$) δ 190.4, 158.4 (d, $^1J_{CF}$ = 243.5 Hz), 152.3, 149.0, 148.4, 130.3 (d, $^3J_{CF}$ = 6.1 Hz), 129.5, 122.3 (d, $^3J_{CF}$ = 7.0 Hz), 121.7 (d, $^2J_{CF}$ = 22.4 Hz), 120.6, 114.8 (d, $^2J_{CF}$ = 22.7 Hz), 111.2, 110.9, 62.8, 55.9, 55.8, 42.8; **^{19}F-NMR** (377 MHz, $CDCl_3$) δ -119.9; **IR** (film, cm^{-1}) 2958, 1683, 1514, 1491, 1264, 1144, 1028, 813; **MS** (ESI^+) *m/z* (%) 151 (37), 304 (6), 326 (44), 336 (100); **HRMS** (ESI^+) calc. for $C_{17}H_{18}O_3NF^{23}Na$ $(M+Na)^+$: 326.11629, found: 326.11655. **m.p.** (ºC): 67-68.

Hydroacylation/Conjugate Addition

General procedure A (racemic version, using dppe)

To an oven dried reaction tube containing a magnetic stirrer was added $[Rh(dppe)(C_6H_5F)][BAr^F_4]$ (29.2 mg, 0.02 mmol, 10 mol%) and the flask was backfilled with N_2 prior to dissolving in acetone (1 mL). To this reaction tube was added a solution of aminobenzaldehyde (0.20 mmol, 1 equiv.) and alkyne (0.26 mmol, 1.3 equiv.) in acetone (0.5 mL). The resulting solution was heated at 55 °C for 30 min, after which boronic acid (0.40 mmol, 2 equiv.) and potassium carbonate (0.04 mmol, 0.2 equiv.) were added in a mixture of acetone:water (7:3, 0.5 mL). The resulting mixture was stirred for 3 h and

then allowed to cool to room temperature. Solvents were evaporated and the residual crude material was directly charged onto silica gel and subjected to flash column chromatographical purification (FC) to afford the corresponding pure product.

General procedure B (asymmetric version, using (*R*,*R*)-MeDuPhos)

To an oven dried reaction tube containing a magnetic stirrer was added [Rh(*R*,*R*-MeDuPhos)(C_6H_5F)][BAr^F_4] (27.4 mg, 0.02 mmol, 10 mol%) and the flask was backfilled with N_2 prior to dissolving in acetone (1 mL). To this reaction tube was added a solution of aminobenzaldehyde (0.20 mmol, 1 equiv.) and alkyne (0.26 mmol, 1.3 equiv.) in acetone (0.5 mL). The resulting solution was heated at 55 °C for 30 min, after which boronic acid (0.40 mmol, 2 equiv.) and potassium carbonate (0.04 mmol, 0.2 equiv.) were added in a mixture of acetone:water (7:3, 0.5 mL). The resulting mixture was stirred for 3 h and then allowed to cool to room temperature. Solvents were evaporated and the residual crude material was directly charged onto silica gel and subjected to flash column chromatographical purification (FC) to afford the corresponding pure product.

General procedure C (asymmetric version, using the chiral diene in acetone)

To an oven dried reaction tube containing a magnetic stirrer were added [Rh(dcpm)(C_6H_5F)][BAr^F_4] (8.8 mg, 0.006 mmol, 3 mol%) and [Rh(L2)($MeCN)_2$][BAr^F_4] (19.2 mg, 0.014 mmol, 7 mol%) and the flask was backfilled with N_2 prior to dissolving in acetone (1 mL). To this reaction flask was added a solution of aminobenzaldehyde (0.20 mmol, 1 equiv.) and alkyne (0.26 mmol, 1.3 equiv.) in acetone (0.5 mL). The resulting solution was heated at 55 °C for 30 min, after which boronic acid (0.40 mmol, 2 equiv.) and potassium carbonate (0.04 mmol, 0.2 equiv.) were added in a mixture of acetone:water (7:3, 0.5 mL). The resulting mixture was stirred for 3 h and then allowed to cool to room temperature. Solvents were evaporated and the residual crude material was directly charged onto silica gel and subjected to flash column chromatographical purification (FC) to afford the corresponding pure product.

General procedure D (asymmetric version, using the chiral diene in dichloroethane)

To an oven dried reaction tube containing a magnetic stirrer were added [Rh(dcpm)(C_6H_5F)][BAr^F_4] (8.8 mg, 0.006 mmol, 3 mol%) and [Rh(L2)($MeCN)_2$][BAr^F_4] (19.2 mg, 0.014 mmol, 7 mol%) and the flask was backfilled with N_2 prior to dissolving in dichloroethane (1 mL). To this reaction flask was added a solution of aminobenzaldehyde (0.20 mmol, 1 equiv.) and alkyne (0.26 mmol, 1.3 equiv.) in dichloroethane (0.5 mL). The resulting solution was heated at 55 °C for 30 min, after which boronic acid (0.40 mmol, 2 equiv.), potassium carbonate (0.04 mmol, 0.2 equiv.), dichloroethane (0.35 mL) and water (0.15 mL) were added. The resulting mixture was stirred for 3 h and then allowed to cool to room temperature. Solvents were evaporated and the residual crude material was

directly charged onto silica gel and subjected to flash column chromatographical purification (FC) to afford the corresponding pure product.

1-{2-[(3,4-Dimethoxybenzyl)(methyl)amino]phenyl}-3-phenylnonan-1-one (2a)

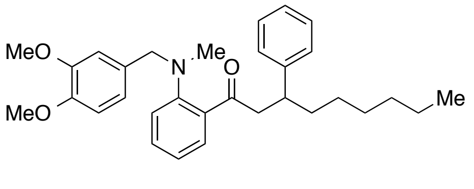

Following the general procedure and starting from 2-[(3,4-dimethoxybenzyl)(methyl)amino]benzaldehyde (57 mg, 0.20 mmol), 1-octyne (38 μL, 0.26 mmol) and phenylboronic acid (48 mg, 0.40 mmol), the product was isolated by FC (petrol/ether 3:1) as a yellow oil.

General procedure A: 91% yield (86 mg, 0.18 mmol).

General procedure B: 76% yield (72 mg, 0.17 mmol). 78% ee (*S*).

General procedure C: 87% yield (82 mg, 0.17 mmol). 96% ee (*S*). $[\alpha]_D^{25}$: −3.7 (c = 1.0, $CHCl_3$).

^{1}H-NMR (400 MHz, $CDCl_3$) δ 7.29 (ddd, J = 8.2, 7.2, 1.7 Hz, 1H), 7.25-7.11 (m, 6H), 6.94-6.90 (m, 2H), 6.75 (d, J = 8.2 Hz, 1H), 6.69-6.66 (m, 1H), 6.56 (d, J = 1.9 Hz, 1H), 3.97 (app. q, J = 12.9 Hz, 2H), 3.87 (s, 3H), 3.70 (s, 3H), 3.42 (dd, J = 15.9, 7.7 Hz, 1H), 3.29 (dd, J = 15.9, 6.7 Hz, 1H), 3.24-3.18 (m, 1H), 2.53 (s, 3H), 1.67-1.56 (m, 2H), 1.26-1.08 (m, 8H), 0.86-0.81 (m, 3H); **^{13}C-NMR** (100 MHz, $CDCl_3$) δ 206.0, 150.9, 148.9, 148.3, 145.0, 134.7, 131.3, 129.9, 129.2, 128.4, 127.8, 126.2, 121.4, 120.9, 119.3, 111.6, 110.8, 60.8, 56.0, 55.8, 49.3, 41.9, 41.3, 36.6, 31.8, 29.3, 27.5, 22.7, 14.2; **IR** (film, cm^{-1}) 2927, 2854, 1678, 1593, 1514; **MS** (ESI^+) *m/z* (%) 474 (100), 475 (31), 496 (10); **HRMS** (ESI^+) calc. for $C_{31}H_{40}O_3N$ $(M+H)^+$: 474.30027, found: 474.30029; The ee was determined by HPLC using a Chiralpak AD-H column [*n*-hexane/*i*-PrOH (97:3)]; flow rate 1.0 mL/min; τ_{major} = 33.48 min, τ_{minor} = 37.45 min.

1-{2-[(3,4-Dimethoxybenzyl)(methyl)amino]phenyl}-3-(*p*-toluenesulfonyl)nonan-1-one (2b)

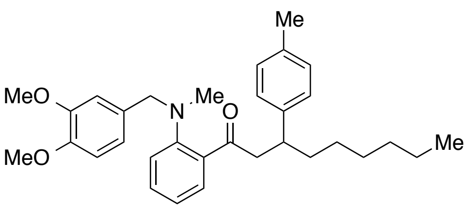

Following the general procedure and starting from 2-[(3,4-dimethoxybenzyl)(methyl)amino]benzaldehyde (57 mg, 0.20 mmol), 1-octyne (38 μL, 0.26 mmol) and 4-methylphenylboronic acid (54 mg, 0.40 mmol), the product was isolated by FC (petrol/ether 85:15) as a yellow oil.

General procedure A: 92% yield (90 mg, 0.18 mmol).

General procedure B: 83% yield (81 mg, 0.17 mmol). 77% ee (*S*).

General procedure C: 83% yield (81 mg, 0.17 mmol). 92% ee (*S*). $[\alpha]_D^{25}$: −1.8 (c = 1.0, $CHCl_3$).

^{1}H-NMR (400 MHz, $CDCl_3$) δ 7.32-7.28 (m, 1H), 7.19 (dd, J = 7.5, 1.7 Hz, 1H), 7.02 (s, 4H), 6.95-6.91 (m, 2H), 6.75 (d, J = 8.1 Hz, 1H), 6.67 (dd, J = 8.1, 1.8 Hz, 1H), 6.57 (d, J = 1.8 Hz, 1H), 3.97 (app. q, J = 13.0 Hz, 2H), 3.86 (s, 3H), 3.69 (s, 3H), 3.37 (dd, J = 15.9, 7.6 Hz, 1H), 3.30 (dd, J =

15.9, 6.9 Hz, 1H), 3.19-3.12 (m, 1H), 2.54 (s, 3H), 2.28 (s, 3H), 1.57 (s, 2H), 1.26-1.13 (m, 8H), 0.83 (t, J = 7.0 Hz, 3H); **^{13}C-NMR** (100 MHz, $CDCl_3$) δ 206.1, 150.9, 148.9, 148.3, 141.9, 135.6, 134.7, 131.3, 129.9, 129.2, 129.0, 127.6, 121.4, 120.9, 119.2, 111.6, 110.7, 60.8, 55.93, 55.74, 49.4, 41.5, 41.3, 36.6, 31.8, 29.3, 27.5, 22.7, 21.1, 14.2; **IR** (film, cm^{-1}) 2926, 1677, 1592, 1514, 1260, 1029; **MS** (ESI^+) *m/z* (%) 488 (100), 489 (32), 510 (8); **HRMS** (ESI^+) calc. for $C_{32}H_{41}O_3NNa$ $(M+Na)^+$: 510.29787, found: 510.29712; The ee was determined by HPLC using a Chiralpak AD-H column [*n*-hexane/*i*-PrOH (97:3)]; flow rate 1.0 mL/min; τ_{major} = 31.28 min, τ_{minor} = 33.98 min.

1-{2-[(3,4-Dimethoxybenzyl)(methyl)amino]phenyl}-3-(*o*-toluenesulfonyl)nonan-1-one (2c)

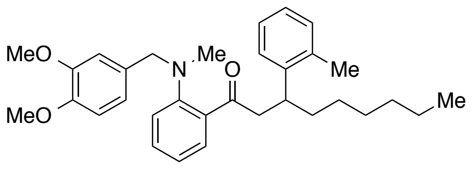

Following the general procedure and starting from 2-[(3,4-dimethoxybenzyl)(methyl)amino]benzaldehyde (57 mg, 0.20 mmol), 1-octyne (38 μL, 0.26 mmol) and 2-methylphenylboronic acid (54 mg, 0.40 mmol), the product was isolated by FC (petrol/ether 85:15) as a yellow oil.

General procedure A: 55% yield (54 mg, 0.11 mmol).

General procedure C: 67% yield (65 mg, 0.13 mmol). 94% ee (*S*). $[\alpha]_D^{25}$: −1.8 (c = 1.0, $CHCl_3$).

^{1}H-NMR (400 MHz, $CDCl_3$) δ 7.29 (ddd, J = 8.2, 7.2, 1.7 Hz, 1H), 7.14-6.99 (m, 5H), 6.95-6.88 (m, 2H), 6.74 (d, J = 8.1 Hz, 1H), 6.66 (dd, J = 8.1, 2.0 Hz, 1H), 6.56 (d, J = 2.0 Hz, 1H), 4.05-3.91 (m, 2H), 3.85 (s, 3H), 3.68 (s, 3H), 3.55-3.48 (m, 1H), 3.40 (dd, J = 15.9, 7.5 Hz, 1H), 3.30 (dd, J = 15.9, 7.0 Hz, 1H), 2.53 (s, 3H), 2.25 (s, 3H), 1.72-1.49 (m, 2H), 1.33-1.08 (m, 8H), 0.84 (t, J = 6.9 Hz, 3H); **^{13}C-NMR** (100 MHz, $CDCl_3$) δ 206.2, 150.8, 148.9, 148.3, 143.3, 136.3, 134.7, 131.3, 130.3, 129.9, 129.1, 126.1, 126.0, 125.8, 121.4, 120.9, 119.2, 111.6, 110.8, 60.8, 56.0, 55.8, 48.9, 41.3, 36.8, 36.4, 31.9, 29.6, 27.4, 22.7, 19.9, 14.2; **IR** (film, cm^{-1}) 2927, 1675, 1590, 1512, 1261, 1027; **MS** (ESI^+) *m/z* (%) 488 (100), 489 (28), 510 (7); **HRMS** (ESI^+) calc. for $C_{32}H_{41}O_3NNa$ $(M+Na)^+$: 510.29787, found: 510.29737; The ee was determined by HPLC using a Chiralpak AD-H column [*n*-hexane/*i*-PrOH (95:5)]; flow rate 1.0 mL/min; τ_{major} = 12.87 min, τ_{minor} = 17.78 min.

3-[4-(*tert*-Butyl)phenyl]-1-{2-[(3,4-dimethoxybenzyl)(methyl)amino]phenyl}nonan-1-one (2d)

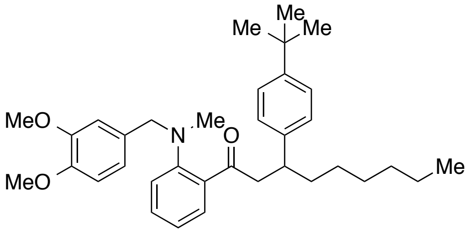

Following the general procedure A and starting from 2-[(3,4-dimethoxybenzyl)(methyl)amino]benzaldehyde (57 mg, 0.20 mmol), 1-octyne (38 μL, 0.26 mmol) and 4-*tert*-butylphenylboronic acid (71 mg, 0.40 mmol), the product was isolated by FC (petrol/ether 85:15) as a yellow oil.

General procedure A: 84% yield (89 mg, 0.17 mmol).

General procedure C: 87% yield (92 mg, 0.17 mmol). 92% ee (*S*). $[\alpha]_D^{25}$: −5.9 (c = 1.0, $CHCl_3$).

^{1}H-NMR (400 MHz, $CDCl_3$) *δ* 7.30-7.26 (m, 1H), 7.21-7.19 (m, 2H), 7.13 (d, *J* = 1.5 Hz, 1H), 7.05-7.03 (m, 2H), 6.91 (dd, *J* = 8.1, 1.9 Hz, 2H), 6.75 (d, *J* = 8.1 Hz, 1H), 6.67 (d, *J* = 1.9 Hz, 1H), 6.57 (d, *J* = 1.5 Hz, 1H), 4.01-3.92 (m, 2H), 3.85 (s, 3H), 3.69 (s, 3H), 3.40 (dd, *J* = 15.6, 7.5 Hz, 1H), 3.28-3.13 (m, 2H), 2.50 (s, 3H), 1.67-1.52 (m, 2H), 1.29-1.14 (m, 17H), 0.83 (t, *J* = 7.1 Hz, 3H); **^{13}C-NMR** (100 MHz, $CDCl_3$) *δ* 206.4, 150.8, 148.9, 148.8, 148.3, 141.8, 134.9, 131.2, 129.9, 129.2, 127.4, 125.2, 121.4, 120.9, 119.2, 111.6, 110.8, 60.7, 56.0, 55.8, 49.4, 41.5, 41.3, 36.5, 34.4, 31.9, 31.5, 29.4, 27.6, 22.8, 14.2; **IR** (film, cm^{-1}) 2927, 2856, 1677, 1593, 1514; **MS** (ESI^+) *m/z* (%) 530 (100), 531 (37); **HRMS** (ESI^+) calc. for $C_{35}H_{48}O_3N$ $(M+H)^+$: 530.36287, found: 530.36244; The ee was determined by HPLC using a Chiralpak AD-H column [*n*-hexane/*i*-PrOH (97:3)]; flow rate 1.0 mL/min; τ_{major} = 19.51 min, τ_{minor} = 24.30 min.

3-(4-Chlorophenyl)-1-2-[(3,4-dimethoxybenzyl)(methyl)amino]phenyl}nonan-1-one (2e)

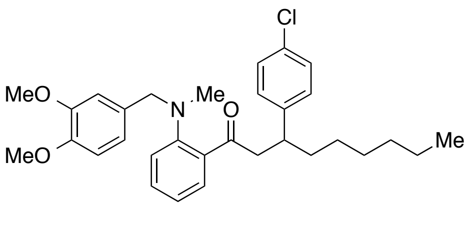

Following the general procedure and starting from 2-[(3,4-dimethoxybenzyl)(methyl)amino]benzaldehyde (57 mg, 0.20 mmol), 1-octyne (38 µL, 0.26 mmol) and 4-chlorophenylboronic acid (63 mg, 0.40 mmol), the product was isolated by FC (petrol/ether 3:1) as a yellow oil.

General procedure A: 90% yield (91 mg, 0.18 mmol).

General procedure B: 93% yield (94 mg, 0.19 mmol). 86% ee (*S*).

General procedure D: 72% yield (73 mg, 0.14 mmol). 94% ee (*S*). $[\alpha]_D^{25}$: −8.0 (*c* = 1.0, $CHCl_3$).

^{1}H-NMR (400 MHz, $CDCl_3$) *δ* 7.35-7.27 (m, 1H), 7.21-7.13 (m, 3H), 7.09-7.02 (m, 2H), 6.97-6.90 (m, 2H), 6.78-6.74 (m, 1H), 6.68-6.64 (m, 1H), 6.54 (d, *J* = 8.4 Hz, 1H), 4.02-3.83 (m, 5H), 3.71(s, 3H), 3.39 (dd, *J* = 16.1, 8.2 Hz, 1H), 3.27 (dd, *J* = 16.1, 6.2 Hz, 1H), 3.23-3.14 (m, 1H), 2.52 (s, 3H), 1.69-1.48 (m, 2H), 1.35-1.02 (m, 8H), 0.84 (t, *J* = 7.0 Hz 3H); **^{13}C-NMR** (100 MHz, $CDCl_3$) *δ* 205.5, 151.0, 148.9, 148.4, 143.5, 134.6, 131.8, 131.4, 129.8, 129.22, 129.19, 128.5, 121.5, 121.0, 119.3, 111.7, 110.9, 60.9, 56.0, 55.8, 49.1, 41.3, 41.2, 36.6, 31.8, 29.3, 27.5, 22.7, 14.2; **IR** (film, cm^{-1}) 2927, 2855, 1677, 1592, 1515, 762, 737; **MS** (ESI^+) *m/z* (%) 508 (100), 509 (33); **HRMS** (ESI^+) calc. for $C_{31}H_{39}O_3N^{35}Cl$ $(M+H)^+$: 508.26130, found: 508.26086; The ee was determined by HPLC using a Chiralpak AD-H column [*n*-hexane/*i*-PrOH (95:5)]; flow rate 1.0 mL/min; τ_{major} = 22.32 min, τ_{minor} = 24.12 min.

3-(4-Bromophenyl)-1-{2-[(3,4-dimethoxybenzyl)(methyl)amino]phenyl}nonan-1-one (2f)

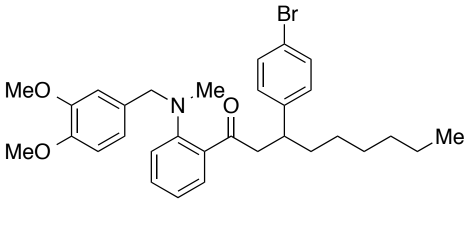

Following the general procedure and starting from 2-[(3,4-dimethoxybenzyl)(methyl)amino]benzaldehyde (57 mg, 0.20 mmol), 1-octyne (38 µL, 0.26 mmol) and 4-

bromophenylboronic acid (80 mg, 0.40 mmol), the product was isolated by FC (petrol/ether 4:1) as a yellow oil.

General procedure A: 81% yield (89 mg, 0.16 mmol).

General procedure D: 70% yield (77 mg, 0.14 mmol). 95% ee (*S*). $[\alpha]_D^{25}$: −8.6 (*c* = 1.0, $CHCl_3$).

^{1}H-NMR (400 MHz, $CDCl_3$) δ 7.34-7.27 (m, 3H), 7.16 (dd, *J* = 7.9, 1.7 Hz, 1H), 7.01-6.98 (m, 2H), 6.95-6.90 (m, 2H), 6.75 (d, *J* = 8.2 Hz, 1H), 6.65 (dd, *J* = 8.2, 2.0 Hz, 1H), 6.55 (d, *J* = 2.0 Hz, 1H), 4.01-3.87 (m, 2H), 3.86 (s, 3H), 3.71 (s, 3H), 3.38 (dd, *J* = 16.1, 8.2 Hz, 1H), 3.26 (dd, *J* = 16.1, 6.2 Hz, 1H), 3.21-3.14 (m, 1H), 2.52 (s, 3H), 1.70-1.47 (m, 2H), 1.33-0.99 (m, 8H), 0.83 (t, *J* = 7.0 Hz, 3H); **^{13}C-NMR** (100 MHz, $CDCl_3$) δ 205.5, 150.9, 148.9, 148.4, 144.0, 134.5, 131.42, 131.44, 129.8, 129.6, 129.2, 121.5, 120.9, 119.9, 119.3, 111.7, 110.8, 60.9, 56.0, 55.8, 49.1, 41.3, 41.2, 36.5, 31.8, 29.3, 27.4, 22.7, 14.2; **IR** (film, cm^{-1}) 2928, 1676, 1593, 1515, 729; **MS** (ESI^+) *m/z* (%) 552 (100), 553 (29), 554 (97), 555 (28); **HRMS** (ESI^+) calc. for $C_{31}H_{39}O_3N^{79}Br$ $(M+H)^+$: 552.21078, found: 552.21069; The ee was determined by HPLC using a Chiralpak AD-H column [*n*-hexane/*i*-PrOH (95:5)]; flow rate 1.0 mL/min; τ_{major} = 22.52 min, τ_{minor} = 25.05 min.

3-(3-Bromophenyl)-1-{2-[(3,4-dimethoxybenzyl)(methyl)amino]phenyl}nonan-1-one (2g)

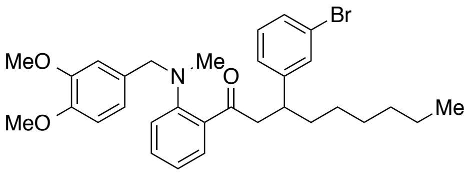

Following the general procedure and starting from 2-[(3,4-dimethoxybenzyl)(methyl)amino]benzaldehyde (57 mg, 0.20 mmol), 1-octyne (38 μL, 0.26 mmol) and 3-bromophenylboronic acid (80 mg, 0.40 mmol), the product was isolated by FC (petrol/ether 7:3) as a yellow oil.

General procedure A: 62% yield (69 mg, 0.13 mmol).

General procedure D: 48% yield (53 mg, 0.10 mmol). 98% ee (*S*). $[\alpha]_D^{25}$: −6.2 (*c* = 1.0, $CHCl_3$).

^{1}H-NMR (400 MHz, $CDCl_3$) δ 7.31-7.23 (m, 3H), 7.16 (dd, *J* = 7.8, 1.7 Hz, 1H), 7.08-7.06 (m, 2H), 6.94-6.90 (m, 2H), 6.75 (d, *J* = 8.2 Hz, 1H), 6.65 (dd, *J* = 8.2, 1.9 Hz, 1H), 6.52 (d, *J* = 1.9 Hz, 1H), 3.95 (app. q, *J* = 12.7 Hz, 2H), 3.85 (s, 3H), 3.70 (s, 3H), 3.42 (dd, *J* = 16.0, 8.0 Hz, 1H), 3.27-3.15 (m, 2H), 2.53 (s, 3H), 1.64-1.52 (m, 2H), 1.25-1.07 (m, 8H), 0.83 (t, *J* = 7.0 Hz, 3H); **^{13}C-NMR** (100 MHz, $CDCl_3$) δ 205.5, 150.9, 148.8, 148.3, 147.5, 134.5, 131.4, 130.7, 129.9, 129.7, 129.3, 129.2, 126.8, 122.5, 121.5, 120.9, 119.4, 111.6, 110.8, 60.9, 56.0, 55.8, 48.9, 41.7, 41.2, 36.5, 31.8, 29.3, 27.4, 22.7, 14.2; **IR** (film, cm^{-1}) 2928, 1676, 1593, 1515, 1261, 1029, 762; **MS** (ESI^+) *m/z* (%) 396 (49), 397 (10), 398 (11), 552 (86), 553 (29), 554 (100), 555 (31); **HRMS** (ESI^+) calc. for $C_{31}H_{38}O_3N^{79}BrNa$ $(M+Na)^+$: 574.19273, found: 574.19244; The ee was determined by HPLC using a Chiralpak AD-H column [*n*-hexane/*i*-PrOH (95:5)]; flow rate 1.0 mL/min; τ_{major} = 18.67 min, τ_{minor} = 21.54 min.

1-{2-[(3,4-Dimethoxybenzyl)(methyl)amino]phenyl}-3-(4-methoxyphenyl)nonan-1-one (2h)

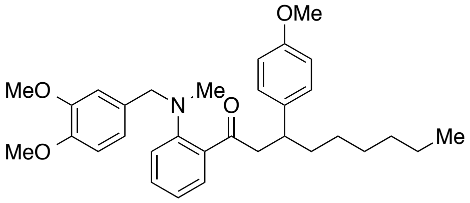

Following the general procedure and starting from 2-[(3,4-dimethoxybenzyl)(methyl)amino]benzaldehyde (57 mg, 0.20 mmol), 1-octyne (38 μL, 0.26 mmol) and 4-methoxyphenylboronic acid (61 mg, 0.40 mmol), the product was isolated by FC (petrol/ether 85:15) as a yellow oil.

General procedure A: 75% yield (76 mg, 0.15 mmol).

General procedure B: 61% yield (61 mg, 0.12 mmol). 75% ee (*S*).

General procedure C: 77% yield (78 mg, 0.15 mmol). 96% ee (*S*). $[\alpha]_D^{25}$: −2.6 (*c* = 1.0, $CHCl_3$).

^{1}H-NMR (400 MHz, $CDCl_3$) *δ* 7.29 (ddd, *J* = 8.2, 7.2, 1.7 Hz, 1H), 7.16 (dd, *J* = 7.6, 1.7 Hz, 1H), 7.05-7.02 (m, 2H), 6.94-6.90 (m, 2H), 6.76-6.71 (m, 3H), 6.67 (dd, *J* = 8.1, 1.9 Hz, 1H), 6.57 (d, *J* = 1.9 Hz, 1H), 4.01-3.90 (m, 2H), 3.85 (s, 3H), 3.75 (s, 3H), 3.68 (s, 3H), 3.37 (dd, *J* = 15.9, 8.0 Hz, 1H), 3.27 (dd, *J* = 15.9, 6.5 Hz, 1H), 3.18-3.13 (m, 1H), 2.53 (s, 3H), 1.65-1.50 (m, 2H), 1.29-1.07 (m, 8H), 0.83 (t, *J* = 7.0 Hz, 3H); **^{13}C-NMR** (100 MHz, $CDCl_3$) *δ* 206.1, 158.0, 150.9, 148.9, 148.3, 137.0, 134.7, 131.3, 130.0, 129.2, 128.7, 121.4, 120.9, 119.2, 113.7, 111.6, 110.8, 60.8, 56.0, 55.8, 55.3, 49.5, 41.26, 41.10, 36.8, 31.8, 29.3, 27.5, 22.7, 14.2; **IR** (film, cm^{-1}) 2927, 1677, 1592, 1511; **MS** (ESI^+) *m/z* (%) 504 (100), 507 (34); **HRMS** (ESI^+) calc. for $C_{32}H_{42}O_4N$ $(M+H)^+$: 504.31084, found: 504.31024; The ee was determined by HPLC using a Chiralpak AD-H column [*n*-hexane/*i*-PrOH (95:5)]; flow rate 1.0 mL/min; τ_{major} = 28.93 min, τ_{minor} = 34.41 min.

1-{2-[(3,4-Dimethoxybenzyl)(methyl)amino]phenyl}-3-(4-hydroxyphenyl)nonan-1-one (2i)

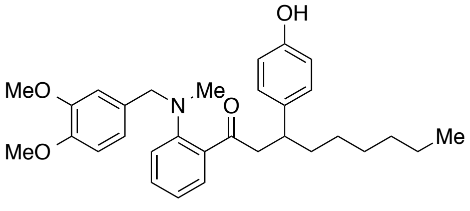

Following the general procedure and starting from 2-[(3,4-dimethoxybenzyl)(methyl)amino]benzaldehyde (57 mg, 0.20 mmol), 1-octyne (38 μL, 0.26 mmol) and 4-hydroxyphenylboronic acid (56 mg, 0.40 mmol), the product was isolated by FC (petrol/ethyl acetate 7:3) as a yellow oil.

General procedure A: 68% yield (67 mg, 0.14 mmol).

General procedure C: 84% yield (82 mg, 0.17 mmol). 97% ee (*S*). $[\alpha]_D^{25}$: −1.7 (*c* = 1.0, $CHCl_3$).

^{1}H-NMR (400 MHz, $CDCl_3$) *δ* 7.29 (ddd, *J* = 8.2, 7.3, 1.7 Hz, 1H), 7.15 (dd, *J* = 7.6, 1.5 Hz, 1H), 6.97-6.89 (m, 4H), 6.75 (d, *J* = 8.2 Hz, 1H), 6.69-6.63 (m, 3H), 6.56 (d, *J* = 1.9 Hz, 1H), 5.44 (s, 1H), 4.02-3.92 (m, 2H), 3.86 (s, 3H), 3.71 (s, 3H), 3.36 (dd, *J* = 15.9, 8.3 Hz, 1H), 3.28 (dd, *J* = 15.9, 6.3 Hz, 1H), 3.15-3.07 (m, 1H), 2.50 (s, 3H), 1.65-1.48 (m, 2H), 1.28-1.05 (m, 8H), 0.83 (t, *J* = 7.0 Hz, 3H); **^{13}C-NMR** (100 MHz, $CDCl_3$) *δ* 206.6, 154.0, 150.8, 148.7, 148.1, 136.6, 134.3, 131.3, 129.8, 129.1, 128.7, 121.3, 120.8, 119.1, 115.1, 111.5, 110.7, 60.7, 55.8, 55.7, 49.3, 41.2, 41.1, 36.7, 31.7,

29.2, 27.4, 22.6, 14.1; **IR** (film, cm^{-1}) 3406, 2928, 1676, 1592, 1514, 1260; **MS** (ESI^{+}) *m/z* (%) 490 (100), 491 (35), 512 (5); **HRMS** (ESI^{+}) calc. for $C_{31}H_{39}O_4NNa$ $(M+Na)^+$: 512.27713, found: 512.27649; The ee was determined by HPLC using a Chiralpak AD-H column [*n*-hexane/*i*-PrOH (85:15)]; flow rate 1.0 mL/min; τ_{major} = 31.54 min, τ_{minor} = 25.19 min.

3-(4-Acetylphenyl)-1-{2-[(3,4-dimethoxybenzyl)(methyl)amino]phenyl}nonan-1-one (2j)

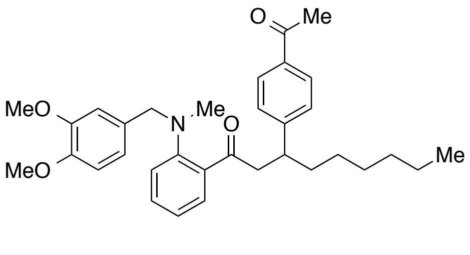

Following the general procedure and starting from 2-[(3,4-dimethoxybenzyl)(methyl)amino]benzaldehyde (57 mg, 0.20 mmol), 1-octyne (38 μL, 0.26 mmol) and 4-acetylphenylboronic acid (66 mg, 0.40 mmol), the product was isolated by FC (gradient petrol/ether 4:1 to 7:3) as a yellow oil.

General procedure A: 90% yield (93 mg, 0.18 mmol).

General procedure D: 73% yield (75 mg, 0.14 mmol). 97% ee (*S*). $[\alpha]_D^{25}$: −7.9 (*c* = 1.0, $CHCl_3$)

^{1}H-NMR (400 MHz, $CDCl_3$) *δ* 7.80 (d, *J* = 8.1 Hz, 2H), 7.31-7.27 (m, 1H), 7.23-7.21 (m, 2H), 7.15 (dd, *J* = 7.6, 1.6 Hz, 1H), 6.93-6.89 (m, 2H), 6.75-6.72 (m, 1H), 6.63 (dd, *J* = 8.1, 1.8 Hz, 1H), 6.53 (d, *J* = 1.8 Hz, 1H), 3.98-3.87 (m, 2H), 3.85 (s, 3H), 3.70 (s, 3H), 3.43 (dd, *J* = 15.8, 8.0 Hz, 1H), 3.35-3.25 (m, 2H), 2.54 (s, 3H), 2.51 (s, 3H), 1.71-1.55 (m, 2H), 1.26-1.09 (m, 8H), 0.82 (t, *J* = 7.0 Hz, 3H); **^{13}C-NMR** (100 MHz, $CDCl_3$) *δ* 205.1, 197.8, 150.8, 150.7, 148.7, 148.2, 135.3, 134.2, 131.4, 129.6, 129.1, 128.4, 128.0, 121.4, 120.8, 119.3, 111.6, 110.7, 60.8, 55.8, 55.6, 48.6, 41.7, 41.0, 36.4, 31.7, 29.2, 27.4, 26.5, 22.6, 14.0; **IR** (film, cm^{-1}) 2925, 1679, 1514, 1264, 1140, 1028; **MS** (ESI^{+}) *m/z* (%) 516 (100), 517 (39); **HRMS** (ESI^{+}) calc. for $C_{33}H_{41}O_4NNa$ $(M+Na)^+$: 538.29278, found: 538.29236; The ee was determined by HPLC using a Chiralpak AD-H column [*n*-hexane/*i*-PrOH (90:10)]; flow rate 1.0 mL/min; τ_{major} = 35.59 min, τ_{minor} = 46.42 min.

Methyl 4-{1-{2-[(3,4-dimethoxybenzyl)(methyl)amino]phenyl}-1-oxononan-3-yl}benzoate (2k)

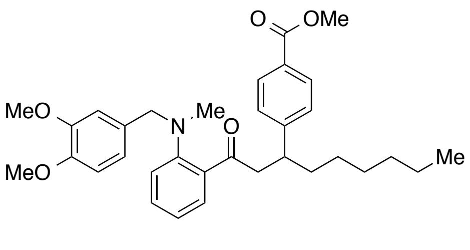

Following the general procedure and starting from 2-[(3,4-dimethoxybenzyl)(methyl)amino]benzaldehyde (57 mg, 0.20 mmol), 1-octyne (38 μL, 0.26 mmol) and 4-(methoxycarbonyl)phenylboronic acid (74 mg, 0.40 mmol), the product was isolated by FC (gradient petrol/ether 4:1 to 7:3) as a yellow oil.

General procedure A: 88% yield (94 mg, 0.18 mmol).

General procedure B: 95% yield (101 mg, 0.19 mmol). 85% ee (*S*).

General procedure D: 74% yield (79 mg, 0.15 mmol). 98% ee (*S*). $[\alpha]_D^{25}$: −10.6 (*c* = 1.0, $CHCl_3$).

^{1}H-NMR (400 MHz, $CDCl_3$) δ 7.88 (d, J = 8.4, 2H), 7.29 (ddd, J = 8.2, 7.3, 1.7 Hz, 1H), 7.20 (d, J = 8.4 Hz, 2H), 7.15 (dd, J = 7.6, 1.7 Hz, 1H), 6.93-6.89 (m, 2H), 6.73 (d, J = 8.1 Hz, 1H), 6.64 (dd, J = 8.1, 1.9 Hz, 1H), 6.53 (d, J = 1.9 Hz, 1H), 3.99-3.86 (m, 5H), 3.85 (s, 3H), 3.70 (s, 3H), 3.46-3.40 (m, 1H), 3.34-3.26 (m, 2H), 2.51 (s, 3H), 1.70-1.56 (m, 2H), 1.25-1.04 (m, 8H), 0.82 (t, J = 7.0 Hz, 3H); **^{13}C-NMR** (100 MHz, $CDCl_3$) δ 205.3, 167.1, 150.9, 150.6, 148.8, 148.3, 134.4, 131.4, 129.71, 129.68, 129.1, 128.2, 127.9, 121.5, 120.9, 119.3, 111.7, 110.8, 60.9, 55.9, 55.8, 52.0, 48.8, 41.8, 41.2, 36.4, 31.8, 29.2, 27.4, 22.7, 14.1; **IR** (film, cm^{-1}) 2928, 1720, 1677, 1592, 1515, 1279; **MS** (ESI^+) *m/z* (%) 532 (100), 533 (35); **HRMS** (ESI^+) calc. for $C_{33}H_{41}O_5NNa$ $(M+Na)^+$: 554.28769, found: 554.28699; The ee was determined by HPLC using a Chiralpak AD-H column [*n*-hexane/*i*-PrOH (90:10)]; flow rate 1.0 mL/min; τ_{major} = 27.66 min, τ_{minor} = 36.02 min.

4-{1-{2-[(3,4-Dimethoxybenzyl)(methyl)amino]phenyl}-1-oxononan-3-yl}benzonitrile (2l)

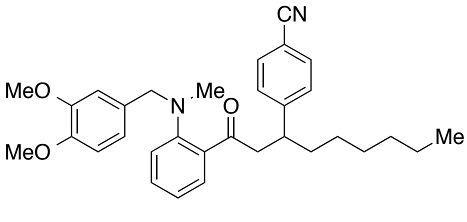

Following the general procedure A and starting from 2-[(3,4-dimethoxybenzyl)(methyl)amino]benzaldehyde (57 mg, 0.20 mmol), 1-octyne (38 μL, 0.26 mmol) and 4-cyanophenylboronic acid (59 mg, 0.40 mmol), the product (88 mg, 0.18 mmol) was isolated by FC (gradient petrol/ether 4:1 to 7:3) in 88% yield as a yellow oil. **^{1}H-NMR** (400 MHz, $CDCl_3$) δ 7.47 (d, J = 8.4 Hz, 2H), 7.30 (ddd, J = 8.2, 7.3, 1.6 Hz, 1H), 7.22 (d, J = 8.4 Hz, 2H), 7.12 (dd, J = 7.7, 1.6 Hz, 1H), 6.93-6.89 (m, 2H), 6.75 (d, J = 8.2 Hz, 1H), 6.64 (dd, J = 8.2, 1.9 Hz, 1H), 6.53 (d, J = 1.9 Hz, 1H), 3.98-3.85 (m, 5H), 3.73 (s, 3H), 3.45-3.39 (m, 1H), 3.34-3.26 (m, 2H), 2.50 (s, 3H), 1.69-1.54 (m, 2H), 1.27-1.01 (m, 8H), 0.83 (t, J = 7.0 Hz, 3H); **^{13}C-NMR** (100 MHz, $CDCl_3$) δ 204.8, 150.8, 150.7, 148.7, 148.3, 134.1, 132.1, 131.5, 129.4, 129.0, 128.6, 121.4, 120.9, 119.3, 119.0, 111.6, 110.7, 109.9, 60.9, 55.9, 55.7, 48.4, 41.9, 41.0, 36.3, 31.6, 29.1, 27.3, 22.6, 14.0; **IR** (film, cm^{-1}) 2928, 2227, 1678, 1592, 1515, 1261, 1028; **MS** (ESI^+) *m/z* (%) 499 (100), 500 (41); **HRMS** (ESI^+) calc. for $C_{32}H_{38}O_3N_2Na$ $(M+Na)^+$: 521.27746, found: 521.27710.

3-[3,5-Bis(trifluoromethyl)phenyl]-1-{2-[(3,4-dimethoxybenzyl)(methyl)amino]phenyl}nonan-1-one (2m)

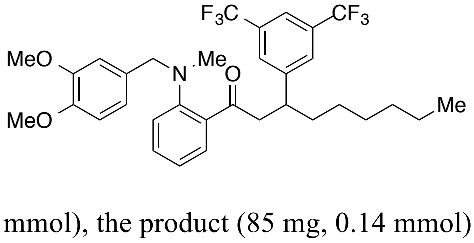

Following the general procedure A and starting from 2-[(3,4-dimethoxybenzyl)(methyl)amino]benzaldehyde (57 mg, 0.20 mmol), 1-octyne (38 μL, 0.26 mmol) and 3,5-bis(trifluoromethyl)phenylboronic acid (103 mg, 0.40 mmol), the product (85 mg, 0.14 mmol) was isolated by FC (petrol/ether 9:1) in 70% yield as a yellow oil. **^{1}H-NMR** (400 MHz, $CDCl_3$) δ 7.65 (s, 1H), 7.57 (s, 2H), 7.26 (ddd, J = 8.2, 7.3, 1.7 Hz, 1H), 7.11 (dd, J = 7.7, 1.7 Hz, 1H), 6.90 (app. dt, J = 7.7, 3.5 Hz, 2H), 6.75 (d, J = 8.2 Hz, 1H), 6.63 (dd, J

= 8.2, 1.8 Hz, 1H), 6.47 (d, J = 1.8 Hz, 1H), 3.99-3.90 (m, 2H), 3.84 (s, 3H), 3.70 (s, 3H), 3.55-3.48 (m, 1H), 3.39-3.32 (m, 2H), 2.58 (s, 3H), 1.76-1.57 (m, 2H), 1.32-1.04 (m, 8H), 0.84 (t, J = 7.1 Hz, 3H); **^{13}C-NMR** (100 MHz, $CDCl_3$) δ 204.8, 150.8, 148.8, 148.4, 147.7, 134.1, 131.7, 131.5 (q, $^2J_{CF}$ = 34.1 Hz), 129.24, 129.20, 128.0 (q, $^3J_{CF}$ = 2.3 Hz), 123.5 (q, $^1J_{CF}$ = 272.8 Hz), 121.6, 120.9, 120.4 (q, $^3J_{CF}$ = 3.5 Hz), 119.5, 111.7, 110.8, 61.2, 55.9, 55.8, 48.3, 42.0, 40.9, 36.5, 31.7, 29.1, 27.4, 22.7, 14.1; **^{19}F-NMR** (377 MHz, $CDCl_3$) δ -62.7; **IR** (film, cm^{-1}) 2931, 1677, 1593, 1516, 1170, 1130; **MS** (ESI^+) *m/z* (%) 610 (100), 611 (35); **HRMS** (ESI^+) calc. for $C_{33}H_{38}O_3NF_6$ $(M+H)^+$: 610.27504, found: 610.27502.

_N_-{3-{1-{2-[(3,4-Dimethoxybenzyl)(methyl)amino]phenyl}-1-oxononan-3-yl}phenyl}acetamide (2n)

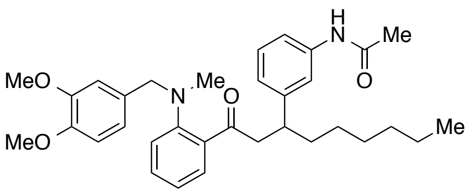

Following the general procedure and starting from 2-[(3,4-dimethoxybenzyl)(methyl)amino]benzaldehyde (57 mg, 0.20 mmol), 1-octyne (38 μL, 0.26 mmol) and 3-acetamidophenylboronic acid (73 mg, 0.40 mmol), the product was isolated by FC (gradient petrol/ethyl acetate 7:3 to 1:1) as a yellow oil.

General procedure A: 92% yield (98 mg, 0.18 mmol).

General procedure C: 62% yield (66 mg, 0.12 mmol). 77% ee (*S*). $[\alpha]_D^{25}$: –3.2 (c = 1.0, $CHCl_3$).

^{1}H-NMR (400 MHz, $CDCl_3$) δ 7.44 (dd, J = 8.0, 1.1 Hz, 1H), 7.38 (s, 1H), 7.29 (ddd, J = 8.3, 7.3, 1.7 Hz, 1H), 7.19-7.12 (m, 3H), 6.95-6.85 (m, 3H), 6.75 (d, J = 8.2 Hz, 1H), 6.67 (dd, J = 8.2, 1.9 Hz, 1H), 6.58 (d, J = 1.9 Hz, 1H), 4.00-3.92 (m, 2H), 3.82 (s, 3H), 3.70 (s, 3H), 3.37 (dd, J = 16.2, 7.7 Hz, 1H), 3.27 (dd, J = 16.2, 6.6 Hz, 1H), 3.21-3.15 (m, 1H), 2.52 (s, 3H), 2.12 (s, 3H), 1.65-1.51 (m, 2H), 1.27-1.06 (m, 8H), 0.82 (t, J = 7.0 Hz, 3H); **^{13}C-NMR** (100 MHz, $CDCl_3$) δ 205.8, 168.3, 150.8, 148.7, 148.2, 145.8, 138.0, 134.5, 131.3, 129.9, 129.1, 128.9, 123.6, 121.3, 120.9, 119.2, 118.8, 117.7, 111.7, 110.7, 60.6, 55.83, 55.78, 48.9, 41.7, 41.3, 36.4, 31.7, 29.2, 27.4, 24.6, 22.6, 14.1; **IR** (film, cm^{-1}) 3263, 2928, 1694, 1667, 1611, 1592, 1514, 1260, 1027; **MS** (ESI^+) *m/z* (%) 531 (100), 532 (26); **HRMS** (ESI^+) calc. for $C_{33}H_{42}O_4N_2Na$ $(M+Na)^+$: 553.30368, found: 553.30261; The ee was determined by HPLC using a Chiralpak AD-H column [*n*-hexane/*i*-PrOH (85:15)]; flow rate 1.0 mL/min; τ_{major} = 18.93 min, τ_{minor} = 23.91 min.

3-(2,3-Dihydrobenzo[*b*][1,4]dioxin-6-yl)-1-{2-[(3,4-dimethoxybenzyl)(methyl)amino]phenyl} nonan-1-one (2o)

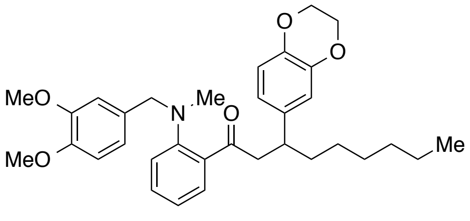

Following the general procedure and starting from 2-[(3,4-dimethoxybenzyl)(methyl)amino]benzaldehyde (57 mg, 0.20 mmol), 1-octyne (38 μL, 0.26 mmol) and (2,3-dihydrobenzo[*b*][1,4]dioxin-6-yl)boronic acid (74 mg, 0.40

mmol), the product was isolated by FC (gradient petrol/ether 4:1 to 7:3) as a yellow oil.

General procedure A: 80% yield (85 mg, 0.16 mmol).

General procedure D: 77% yield (82 mg, 0.15 mmol). 96% ee (*S*). $[\alpha]_D^{25}$: −7.1 (*c* = 1.0, $CHCl_3$).

^{1}H-NMR (400 MHz, $CDCl_3$) δ 7.29 (ddd, *J* = 8.1, 7.4, 1.7 Hz, 1H), 7.20 (dd, *J* = 7.8, 1.7 Hz, 1H), 6.94-6.90 (m, 2H), 6.76 (d, *J* = 8.2 Hz, 1H), 6.71-6.66 (m, 2H), 6.63 (d, *J* = 2.0 Hz, 1H), 6.59 (dd, *J* = 8.2, 2.0 Hz, 1H), 6.56 (d, *J* = 2.0 Hz, 1H), 4.18 (s, 4H), 4.02-3.93 (m, 2H), 3.86 (s, 3H), 3.72 (s, 3H), 3.35 (dd, *J* = 15.9, 7.7 Hz, 1H), 3.24 (dd, *J* = 15.9, 6.8 Hz, 1H), 3.10-3.06 (m, 1H), 2.55 (s, 3H), 1.61-1.48 (m, 2H), 1.27-1.09 (m, 8H), 0.83 (t, *J* = 7.0 Hz, 3H); **^{13}C-NMR** (100 MHz, $CDCl_3$) δ 205.9, 150.9, 148.8, 148.3, 143.3, 141.8, 138.3, 134.6, 131.3, 129.9, 129.2, 121.3, 120.88, 120.83, 119.2, 116.9, 116.2, 111.6, 110.8, 64.44, 64.37, 60.8, 55.9, 55.8, 49.4, 41.3, 41.2, 36.6, 31.8, 29.3, 27.5, 22.7, 14.2; **IR** (film, cm^{-1}) 2928, 1676, 1591, 1514, 1259; **MS** (ESI^+) *m/z* (%) 532 (100), 533 (33); **HRMS** (ESI^+) calc. for $C_{33}H_{41}O_5NNa$ $(M+Na)^+$: 554.28769, found: 554.28680; The ee was determined by HPLC using a Chiralpak AD-H column [*n*-hexane/*i*-PrOH (93:7)]; flow rate 1.0 mL/min; τ_{major} = 43.19 min, τ_{minor} = 47.28 min.

1-{2-[(3,4-Dimethoxybenzyl)(methyl)amino]phenyl}-3-(thiophen-3-yl)nonan-1-one (2p)

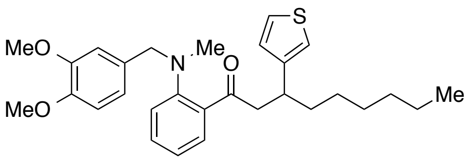

Following the general procedure and starting from 2-[(3,4-dimethoxybenzyl)(methyl)amino]benzaldehyde (57 mg, 0.20 mmol), 1-octyne (38 μL, 0.26 mmol) and thiophen-3-ylboronic acid (51 mg, 0.40 mmol), the product was isolated by FC (petrol/ether 4:1) as a yellow oil.

General procedure A: 60% yield (58 mg, 0.12 mmol).

General procedure D: 54% yield (52 mg, 0.11 mmol). 96% ee (*S*). $[\alpha]_D^{25}$: −2.6 (*c* = 1.0, $CHCl_3$).

^{1}H-NMR (400 MHz, $CDCl_3$) δ 7.32-7.27 (m, 1H), 7.19-7.15 (m, 2H), 6.95-6.91 (m, 2H), 6.90-6.86 (m, 2H), 6.76 (d, *J* = 8.0 Hz, 1H), 6.68 (dd, *J* = 8.0, 1.9 Hz, 1H), 6.57 (d, *J* = 1.9 Hz, 1H), 4.04-3.95 (m, 2H), 3.85 (s, 3H), 3.74 (s, 3H), 3.41-3.33 (m, 2H), 3.30-3.22 (m, 1H), 2.53 (s, 3H), 1.67-1.52 (m, 2H), 1.32-1.11 (m, 8H), 0.84 (d, *J* = 7.0 Hz, 3H); **^{13}C-NMR** (100 MHz, $CDCl_3$) δ 206.0, 150.9, 148.9, 148.3, 145.7, 134.6, 131.3, 129.9, 129.2, 126.9, 125.3, 121.4, 120.9, 120.5, 119.3, 111.7, 110.8, 60.8, 56.0, 55.8, 49.0, 41.3, 37.2, 36.5, 31.9, 29.3, 27.5, 22.7, 14.2; **IR** (film, cm^{-1}) 2927, 2854, 1677, 1592, 1514; **MS** (ESI^+) *m/z* (%) 480 (100), 481 (30); **HRMS** (ESI^+) calc. for $C_{29}H_{38}O_3N^{32}S$ $(M+H)^+$: 480.25669, found: 480.25616. The ee was determined by HPLC using a Chiralpak AD-H column [*n*-hexane/*i*-PrOH (95:5)]; flow rate 1.0 mL/min; τ_{major} = 23.51 min, τ_{minor} = 26.03 min.

1-{2-[(3,4-Dimethoxybenzyl)(methyl)amino]phenyl}-3-(naphthalen-2-yl)nonan-1-one (2q)

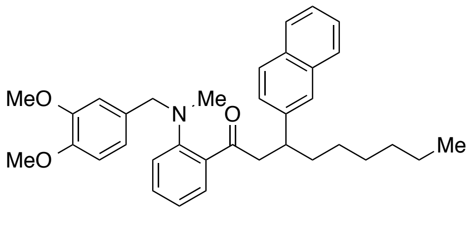

Following the general procedure and starting from 2-[(3,4-dimethoxybenzyl)(methyl)amino]benzaldehyde (57 mg, 0.20 mmol), 1-octyne (38 μL, 0.26 mmol) and naphthalen-2-ylboronic acid (69 mg, 0.40 mmol), the product was isolated by FC (petrol/ether 4:1) as a yellow oil.

General procedure A: 76% yield (80 mg, 0.15 mmol).

General procedure C: 70% yield (73 mg, 0.14 mmol). 95% ee (*S*). $[\alpha]_D^{25}$: −11.9 (*c* = 1.0, $CHCl_3$).

^{1}H-NMR (400 MHz, $CDCl_3$) δ 7.78-7.76 (m, 1H), 7.73-7.71 (m, 2H), 7.56 (d, *J* = 1.0 Hz, 1H), 7.45-7.38 (m, 2H), 7.32-7.27 (m, 2H), 7.17 (dd, *J* = 7.6, 1.6 Hz, 1H), 6.93 (dd, *J* = 8.2, 1.0 Hz, 1H), 6.89 (app. td, *J* = 7.4, 1.0 Hz, 1H), 6.66 (d, *J* = 8.2 Hz, 1H), 6.59 (dd, *J* = 8.2, 1.9 Hz, 1H), 6.50 (d, *J* = 1.9 Hz, 1H), 3.98 (d, *J* = 14.0 Hz, 1H), 3.87 (d, *J* = 14.0 Hz, 1H), 3.81 (s, 3H), 3.63 (s, 3H), 3.58-3.51 (m, 1H), 3.41-3.34 (m, 2H), 2.52 (s, 3H), 1.75-1.67 (m, 2H), 1.27-1.09 (m, 8H), 0.83 (t, *J* = 7.0, Hz 3H); **^{13}C-NMR** (100 MHz, $CDCl_3$) δ 205.9, 151.0, 148.8, 148.2, 142.4, 134.7, 133.6, 132.4, 131.3, 129.8, 129.2, 128.0, 127.7, 127.6, 126.5, 126.1, 125.9, 125.3, 121.4, 120.8, 119.2, 111.5, 110.7, 60.8, 55.9, 55.7, 49.3, 42.0, 41.3, 36.5, 31.8, 29.4, 27.6, 22.7, 14.2; **IR** (film, cm^{-1}) 2928, 1678, 1593, 1514, 1448, 1262, 1029; **MS** (ESI^+) *m/z* (%) 524 (100), 525 (39); **HRMS** (ESI^+) calc. for $C_{35}H_{42}O_3N$ $(M+H)^+$: 524.31592, found: 524.31616; The ee was determined by HPLC using a Chiralpak IC column [*n*-hexane/*i*-PrOH (97:3)]; flow rate 1.0 mL/min; τ_{major} = 45.95 min, τ_{minor} = 41.47 min.

1-{2-[(3,4-Dimethoxybenzyl)(methyl)amino]phenyl}-3-(naphthalen-1-yl)nonan-1-one (2r)

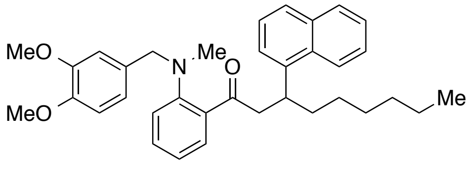

Following the general procedure and starting from 2-[(3,4-dimethoxybenzyl)(methyl)amino]benzaldehyde (57 mg, 0.20 mmol), 1-octyne (38 μL, 0.26 mmol) and naphthalen-1-ylboronic acid (69 mg, 0.40 mmol), the product was isolated by FC (petrol/ether 4:1) as a yellow oil.

General procedure A: 84% yield (88 mg, 0.17 mmol).

General procedure C: 72% yield (75 mg, 0.14 mmol). 90% ee (*S*). $[\alpha]_D^{25}$: −28.4 (*c* = 1.0, $CHCl_3$).

^{1}H-NMR (400 MHz, $CDCl_3$) δ 8.15-8.13 (m, 1H), 7.84-7.80 (m, 1H), 7.67 (dd, *J* = 7.5, 1.6 Hz, 1H), 7.49-7.42 (m, 2H), 7.40-7.34 (m, 2H), 7.32-7.27 (m, 1H), 7.20 (dd, *J* = 7.6, 1.5 Hz, 1H), 6.95 (d, *J* = 8.1 Hz, 1H), 6.90 (app. td, *J* = 7.4, 0.8 Hz, 1H), 6.69-6.67 (m, 1H), 6.61 (dd, *J* = 8.1, 1.3 Hz, 1H), 6.49 (d, *J* = 1.3 Hz, 1H), 4.16 (bs, 1H), 4.01-3.93 (m, 2H), 3.84 (s, 3H), 3.60-3.51 (m, 4H), 3.45 (dd, *J* = 16.0, 7.9 Hz, 1H), 2.52 (s, 3H), 1.83-1.77 (m, 2H), 1.27-1.12 (m, 8H), 0.82 (t, *J* = 7.0 Hz, 3H); **^{13}C-NMR** (100 MHz, $CDCl_3$) δ 206.0, 150.8, 148.7, 148.1, 141.2, 134.6, 134.0, 132.0, 131.3, 129.7, 129.1, 128.8, 126.6, 125.8, 125.4, 125.3, 123.3, 123.2, 121.4, 120.7, 119.1, 111.4, 110.6, 60.7, 55.8,

55.5, 49.0, 41.3, 35.9, 35.0, 31.7, 29.4, 27.4, 22.6, 14.1; **IR** (film, cm^{-1}) 2928, 1677, 1593, 1514, 1448, 1261, 1029; **MS** (ESI$^+$) *m/z* (%) 524 (100), 525 (33); **HRMS** (ESI$^+$) calc. for $C_{35}H_{42}O_3N$ $(M+H)^+$: 524.31592, found: 524.31616; The ee was determined by HPLC using a Chiralpak IA-3 column [*n*-hexane/*i*-PrOH (93:7)]; flow rate 1.0 mL/min; τ_{major} = 15.66 min, τ_{minor} = 14.82 min.

3-(Cyclohex-1-en-1-yl)-1-{2-[(3,4-dimethoxybenzyl)(methyl)amino]phenyl}nonan-1-one (2s)

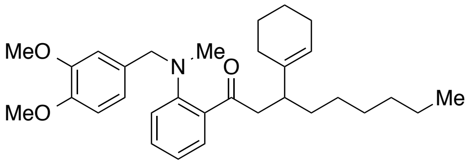

Following the general procedure and starting from 2-[(3,4-dimethoxybenzyl)(methyl)amino]benzaldehyde (57 mg, 0.20 mmol), 1-octyne (38 μL, 0.26 mmol) and cyclohex-1-en-1-ylboronic acid (50 mg, 0.40 mmol), the product was isolated by FC (petrol/ether 85:15) as a yellow oil.

General procedure A: 72% yield (69 mg, 0.14 mmol).

General procedure C: 74% yield (71 mg, 0.15 mmol). 79% ee (*S*). $[\alpha]_D^{25}$: −17.4 (*c* = 1.0, $CHCl_3$).

^{1}H-NMR (400 MHz, $CDCl_3$) *δ* 7.33-7.29 (m, 2H), 6.99-6.94 (m, 2H), 6.77 (d, *J* = 8.2 Hz, 1H), 6.73 (dd, *J* = 8.2, 1.9 Hz, 1H), 6.62 (d, *J* = 1.9 Hz, 1H), 5.30-5.28 (m, 1H), 4.10 (app. q, *J* = 11.8 Hz, 2H), 3.85 (s, 3H), 3.70 (s, 3H), 3.22 (dd, *J* = 14.4, 9.1 Hz, 1H), 2.96 (dd, *J* = 14.4, 5.8 Hz, 1H), 2.63 (s, 3H), 2.48-2.44 (m, 1H), 1.87-1.84 (m, 2H), 1.72-1.71 (m, 2H), 1.47-1.13 (m, 14H), 0.86 (t, *J* = 6.9 Hz, 3H); **^{13}C-NMR** (100 MHz, $CDCl_3$) *δ* 207.1, 150.9, 148.8, 148.2, 138.3, 134.7, 131.2, 129.8, 129.7, 123.1, 121.2, 120.8, 118.9, 111.5, 110.7, 61.0, 55.8, 55.7, 45.9, 44.7, 41.1, 33.5, 31.8, 29.3, 27.3, 25.2, 24.4, 22.8, 22.7, 22.6, 14.1; **IR** (film, cm^{-1}) 2926, 1672, 1593, 1515, 1445, 1261, 1030; **MS** (ESI$^+$) *m/z* (%) 478 (100), 479 (32); **HRMS** (ESI$^+$) calc. for $C_{31}H_{43}O_3NNa$ $(M+Na)^+$: 500.31352, found: 500.31271; The ee was determined by HPLC using a Chiralpak AD-H column [*n*-hexane/*i*-PrOH (95:5)]; flow rate 1.0 mL/min; τ_{major} = 12.33 min, τ_{minor} = 13.61 min.

(*E*)-1-{2-[(3,4-Dimethoxybenzyl)(methyl)amino]phenyl}-3-(3-phenylprop-1-en-1-yl)nonan-1-one (2t)

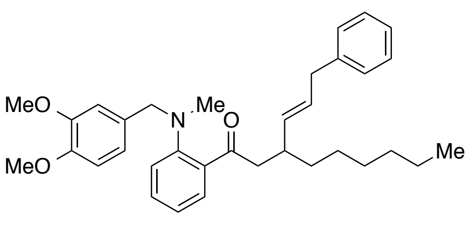

Following the general procedure A and starting from 2-[(3,4-dimethoxybenzyl)(methyl)amino]benzaldehyde (57 mg, 0.20 mmol), 1-octyne (38 μL, 0.26 mmol) and (*E*)-(3-phenylprop-1-en-1-yl)boronic acid (68 mg, 0.40 mmol), the product (58 mg, 0.11 mmol) was isolated by FC (petrol/ether 85:15) in a 56% yield as a yellow oil. **^{1}H-NMR** (400 MHz, $CDCl_3$) *δ* 7.34-7.29 (m, 1H), 7.26 (m, 3H), 7.19-7.15 (m, 1H), 7.13-7.11 (m, 2H), 6.96-6.92 (m, 2H), 6.77 (d, *J* = 8.2 Hz, 1H), 6.72 (dd, *J* = 8.2, 1.8 Hz, 1H), 6.61 (d, *J* = 1.8 Hz, 1H), 5.49 (dt, *J* = 15.1, 6.9 Hz, 1H), 5.25 (ddt, *J* = 15.1, 8.7, 3.2 Hz, 1H), 4.09 (s, 2H), 3.86 (s, 3H), 3.74 (s, 3H), 3.26 (app. d, *J* = 6.7 Hz, 2H), 3.12 (dd, *J* = 15.2, 5.9 Hz, 1H), 3.05 (dd, *J* = 15.2, 8.2 Hz, 1H), 2.64-2.56 (m, 4H), 1.44-1.23 (m, 10H), 0.87 (t, *J* =

6.8 Hz, 3H); **^{13}C-NMR** (100 MHz, $CDCl_3$) δ 206.3, 150.8, 148.8, 148.2, 140.7, 134.9, 134.7, 131.2, 129.7, 129.41, 129.39, 128.5, 128.30, 125.9, 121.5, 120.8, 119.2, 111.5, 110.7, 61.0, 55.8, 55.7, 47.6, 41.3, 39.3, 39.0, 35.3, 31.8, 29.2, 27.2, 22.6, 14.1; **IR** (film, cm^{-1}) 2927, 1676, 1592, 1515, 1465, 1260, 1029; **MS** (ESI^+) *m/z* (%) 514 (100), 515 (32), 536 (17); **HRMS** (ESI^+) calc. for $C_{34}H_{43}O_3NNa$ $(M+Na)^+$: 536.31352, found: 536.31323.

(*E*)-1-{2-[(3,4-Dimethoxybenzyl)(methyl)amino]phenyl}-3-(pent-1-en-1-yl)nonan-1-one (2u)

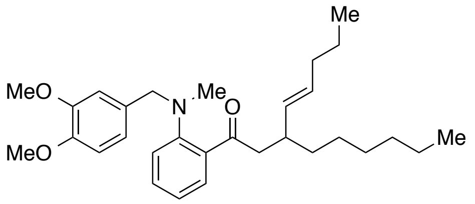

Following the general procedure A and starting from 2-[(3,4-dimethoxybenzyl)(methyl)amino]benzaldehyde (57 mg, 0.20 mmol), 1-octyne (38 μL, 0.26 mmol) and (*E*)-pent-1-en-1-ylboronic acid (46 mg, 0.40 mmol), the product (46 mg, 0.10 mmol) was isolated by FC (petrol/ether 85:15) in a 50% yield as a yellow oil. **^{1}H-NMR** (400 MHz, $CDCl_3$) δ 7.34-7.29 (m, 2H), 7.00-6.95 (m, 2H), 6.78 (d, J = 8.2 Hz, 1H), 6.73 (dd, J = 8.2, 1.9 Hz, 1H), 6.63 (d, J = 1.9 Hz, 1H), 5.30 (dd, J = 15.2, 6.7 Hz, 1H), 5.13 (ddt, J = 15.2, 8.6, 1.3 Hz, 1H), 4.09 (s, 2H), 3.86 (s, 3H), 3.77 (s, 3H), 3.09-2.99 (m, 2H), 2.62 (s, 3H), 2.57-2.49 (m, 1H), 1.90-1.85 (m, 2H), 1.37-1.20 (m, 12H), 0.85 (app. q, J = 7.2 Hz, 6H); **^{13}C-NMR** (100 MHz, $CDCl_3$) δ 206.5, 150.9, 148.8, 148.2, 135.0, 133.2, 131.1, 130.8, 129.8, 129.4, 121.4, 120.8, 119.2, 111.5, 110.7, 60.9, 55.8, 55.7, 47.9, 41.3, 39.4, 35.4, 34.6, 31.8, 29.3, 27.1, 22.64, 22.59, 14.1, 13.7; **IR** (film, cm^{-1}) 2927, 1677, 1593, 1515, 1465, 1261, 1030; **MS** (ESI^+) *m/z* (%) 466 (100), 467 (32); **HRMS** (ESI^+) calc. for $C_{30}H_{43}O_3NNa$ $(M+Na)^+$: 488.31352, found: 488.31326.

1-{2-[Benzyl(methyl)amino]phenyl}-3-phenylnonan-1-one (2v)

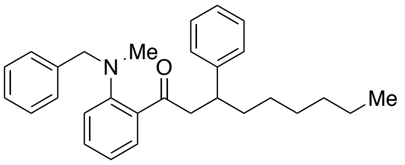

Following the general procedure and starting from 2-[benzyl(methyl)amino]benzaldehyde (45 mg, 0.20 mmol), 1-octyne (38 μL, 0.26 mmol) and phenylboronic acid (48 mg, 0.40 mmol), the product was isolated by FC (petrol/ether 19:1) as a yellow oil.

General procedure A: 99% yield (82 mg, 0.20 mmol).

General procedure C (4 equiv. of boronic acid): 70% yield (58 mg, 0.14 mmol). 98% ee (*S*). $[\alpha]_D^{25}$: −9.5 (c = 1.0, $CHCl_3$).

^{1}H-NMR (400 MHz, $CDCl_3$) δ 7.33-7.13 (m, 12H), 6.98 (dd, J = 8.2, 0.8 Hz, 1H), 6.93 (app. td, J = 7.4, 1.0 Hz, 1H), 4.11 (d, J = 14.1 Hz, 1H), 4.05 (d, J = 14.1 Hz, 1H), 3.43 (dd, J = 16.1, 7.8 Hz, 1H), 3.34 (dd, J = 16.1, 6.6 Hz, 1H), 3.26-3.21 (m, 1H), 2.53 (s, 3H), 1.71-1.57 (m, 2H), 1.31-1.10 (m, 8H), 0.85 (t, J = 7.0 Hz, 3H); **^{13}C-NMR** (100 MHz, $CDCl_3$) δ 205.8, 150.9, 144.9, 137.4, 134.5, 131.3, 129.1, 128.6, 128.3, 128.2, 127.8, 127.3, 126.1, 121.3, 119.0, 60.5, 49.1, 41.9, 41.7, 36.5, 31.7,

29.3, 27.4, 22.6, 14.1; **IR** (film, cm^{-1}) 2925, 1679, 1593, 1486, 1451; **MS** (ESI^{+}) *m/z* (%) 414 (100), 415 (33), 426 (19); **HRMS** (ESI^{+}) calc. for $C_{29}H_{36}ON$ $(M+H)^{+}$: 414.27914, found: 414.27765; The ee was determined by HPLC using a Chiralpak AD-H column [*n*-hexane/*i*-PrOH (97:3)]; flow rate 1.0 mL/min; τ_{major} = 9.10 min, τ_{minor} = 11.11 min.

1-[2-(Dimethylamino)phenyl]-3-phenylnonan-1-one (2w)

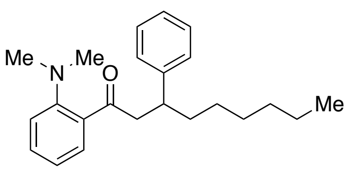

Following the general procedure and starting from 2-(dimethylamino)benzaldehyde (30 mg, 0.20 mmol), 1-octyne (38 μL, 0.26 mmol) and phenylboronic acid (48 mg, 0.40 mmol), the product was isolated by FC (petrol/ether 9:1) as a yellow oil.

General procedure A: 86% yield (58 mg, 0.17 mmol).

General procedure A (3 mmol scale; 5 mol% catalyst; 18 h CA): 97% yield (982 mg, 2.91 mmol).

General procedure B: 80% yield (54 mg, 0.16 mmol). 79% ee (*S*).

General procedure C (4 equiv. of boronic acid): 80% yield (54 mg, 0.16 mmol). 98% ee (*S*). $[\alpha]_D^{25}$: −8.0 (*c* = 1.0, $CHCl_3$).

^{1}H-NMR (400 MHz, $CDCl_3$) *δ* 7.31 (ddd, *J* = 8.2, 7.3, 1.7 Hz, 1H), 7.24-7.21 (m, 2H), 7.16-7.12 (m, 4H), 6.95 (dd, *J* = 8.2, 0.6 Hz, 1H), 6.86 (app. td, *J* = 7.4, 0.9 Hz, 1H), 3.33 (dd, *J* = 15.7, 8.0 Hz, 1H), 3.26-3.13 (m, 2H), 2.63 (s, 6H), 1.66-1.55 (m, 2H), 1.27-1.06 (m, 8H), 0.84 (t, *J* = 7.0 Hz, 3H); **^{13}C-NMR** (100 MHz, $CDCl_3$) *δ* 205.9, 151.5, 144.9, 133.5, 131.3, 129.2, 128.2, 127.8, 126.0, 120.5, 117.0, 48.7, 44.5, 41.9, 36.5, 31.7, 29.2, 27.4, 22.6, 14.1; **IR** (film, cm^{-1}) 2925, 1677, 1594, 1489, 1453; **MS** (ESI^{+}) *m/z* (%) 338 (100), 339 (28), 360 (6); **HRMS** (ESI^{+}) calc. for $C_{23}H_{32}ON$ $(M+H)^{+}$: 338.24784, found: 338.24744; The ee was determined by HPLC using a Chiralpak AD-H column [*n*-hexane/*i*-PrOH (99:1)]; flow rate 1.0 mL/min; τ_{major} = 12.98 min, τ_{minor} = 11.90 min.

1-[2-(Methylamino)phenyl]-3-phenylnonan-1-one (2x)

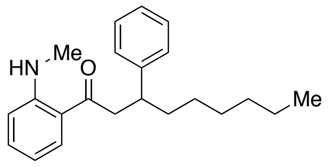

Following the general procedure A and starting from 2-(methylamino)benzaldehyde (27 mg, 0.20 mmol), 1-octyne (38 μL, 0.26 mmol) and phenylboronic acid (48 mg, 0.40 mmol), the product (54 mg, 0.17 mmol) was isolated by FC (petrol/ether 19:1) in a 83% yield as a yellow oil. **^{1}H-NMR** (400 MHz, $CDCl_3$) *δ* 8.78 (bq, *J* = 4.4 Hz, 1H), 7.76 (dd, *J* = 8.1, 1.4 Hz, 1H), 7.38-7.34 (m, 1H), 7.31-7.16 (m, 5H), 6.67 (d, *J* = 8.4 Hz, 1H), 6.59-6.55 (m, 1H), 3.30-3.17 (m, 3H), 2.87 (d, *J* = 5.1 Hz, 3H), 1.73-1.58 (m, 2H), 1.31-1.09 (m, 8H), 0.83 (t, *J* = 6.9 Hz, 3H); **^{13}C-NMR** (100 MHz, $CDCl_3$) *δ* 201.5, 152.0, 145.4, 134.8, 131.7, 128.4, 127.6, 126.1, 117.5, 113.7, 111.3, 46.3, 41.8, 36.5, 31.8, 29.33, 29.28, 27.5, 22.6, 14.1; **IR** (film, cm^{-1}) 3323, 2925, 1634, 1571, 1519, 1425, 1255, 1164; **MS** (ESI^{+}) *m/z* (%) 324 (100), 325 (23), 346 (19); **HRMS** (ESI^{+}) calc. for $C_{22}H_{30}ON$ $(M+H)^{+}$: 324.23219, found: 324.23090.

3-Phenyl-1-[2-(pyrrolidin-1-yl)phenyl]nonan-1-one (2y)

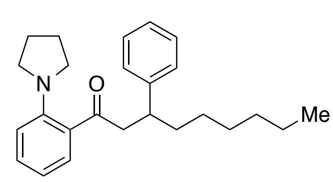

Following the general procedure A and starting from 2-(pyrrolidin-1-yl)benzaldehyde (35 mg, 0.20 mmol), 1-octyne (38 μL, 0.26 mmol) and phenylboronic acid (48 mg, 0.40 mmol), the product (64 mg, 0.18 mmol) was isolated by FC (petrol/ether 85:15) in a 88% yield as a yellow oil. **^{1}H-NMR** (400 MHz, $CDCl_3$) δ 7.41 (dd, J = 7.8, 1.6 Hz, 1H), 7.32-7.24 (m, 5H), 7.20-7.15 (m, 1H), 6.78 (dd, J = 8.5, 0.8 Hz, 1H), 6.72-6.68 (m, 1H), 3.40-3.24 (m, 3H), 2.91-2.85 (m, 2H), 2.72-2.66 (m, 2H), 1.84-1.65 (m, 6H), 1.34-1.15 (m, 8H), 0.88 (t, J = 6.9 Hz, 3H); **^{13}C-NMR** (100 MHz, $CDCl_3$) δ 201.7, 147.3, 145.0, 131.6, 129.3, 128.2, 128.0, 126.7, 126.1, 115.3, 114.2, 51.6, 48.7, 41.7, 36.7, 31.8, 29.3, 27.5, 25.8, 22.7, 14.1; **IR** (film, cm^{-1}) 2925, 1666, 1597, 1446, 1367, 1180; **MS** (ESI^{+}) *m/z* (%) 364 (100), 365 (30); **HRMS** (ESI^{+}) calc. for $C_{25}H_{34}ON$ $(M+H)^{+}$: 364.26349, found: 364.26248.

1-{2-[(3,4-Dimethoxybenzyl)(methyl)amino]-3-methoxyphenyl}-3-phenylnonan-1-one (2z)

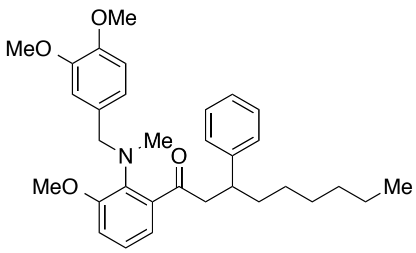

Following the general procedure A and starting from 2-[(3,4-dimethoxybenzyl)(methyl)amino]-3-methoxybenzaldehyde (63 mg, 0.20 mmol), 1-octyne (38 μL, 0.26 mmol) and phenylboronic acid (48 mg, 0.40 mmol), the product (60 mg, 0.12 mmol) was isolated by FC (gradient petrol/ether 4:1 to 3:1) in a 60% yield as a yellow oil. **^{1}H-NMR** (400 MHz, $CDCl_3$) δ 7.26-7.22 (m, 2H), 7.18-7.12 (m, 3H), 7.06 (app. t, J = 7.9 Hz, 1H), 6.89 (dd, J = 8.2, 1.3 Hz, 1H), 6.79-6.73 (m, 3H), 6.52 (dd, J = 7.6, 1.3 Hz, 1H), 3.95 (s, 2H), 3.85 (app. d, J = 10.7 Hz, 6H), 3.83 (s, 3H), 3.21-3.12 (m, 3H), 2.62 (s, 3H), 1.68-1.61 (m, 1H), 1.58-1.49 (m, 1H), 1.26-1.04 (m, 8H), 0.83 (t, J = 7.0 Hz, 3H); **^{13}C-NMR** (100 MHz, $CDCl_3$) δ 206.5, 158.3, 148.7, 147.9, 144.9, 142.7, 138.3, 132.0, 128.3, 127.7, 126.1, 126.0, 121.0, 118.6, 113.0, 112.2, 110.5, 60.3, 55.8, 55.7, 55.3, 50.9, 41.5, 40.4, 36.6, 31.7, 29.3, 27.4, 22.6, 14.1; **IR** (film, cm^{-1}) 2928, 1690, 1514, 1464, 1260, 1029, 909; **MS** (ESI^{+}) *m/z* (%) 504 (100), 505 (34); **HRMS** (ESI^{+}) calc. for $C_{32}H_{42}O_4N$ $(M+H)^{+}$: 504.31084, found: 504.30969.

1-{2-[(3,4-Dimethoxybenzyl)(methyl)amino]-4-methylphenyl}-3-phenylnonan-1-one (2aa)

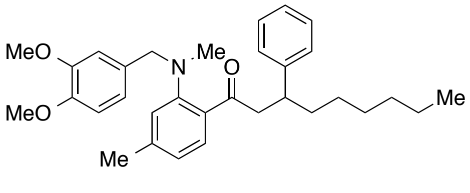

Following the general procedure and starting from 2-[(3,4-dimethoxybenzyl)(methyl)amino]-4-methylbenzaldehyde (60 mg, 0.20 mmol), 1-octyne (38 μL, 0.26 mmol) and phenylboronic acid (48 mg, 0.40 mmol), the product was isolated by FC (petrol/ether 3:1) as a yellow oil.

General procedure A: 85% yield (83 mg, 0.17 mmol).

General procedure B: 74% yield (72 mg, 0.15 mmol). 79% ee (*S*).

General procedure C: 82% yield (80 mg, 0.16 mmol). 95% ee (*S*). $[\alpha]_D^{25}$: −2.4 (*c* = 1.0, $CHCl_3$).

^{1}H-NMR (400 MHz, $CDCl_3$) δ 7.23-7.19 (m, 2H), 7.14-7.11 (m, 4H), 6.76-6.72 (m, 3H), 6.68 (dd, *J* = 8.1, 1.9 Hz, 1H), 6.58 (d, *J* = 1.9 Hz, 1H), 3.99 (d, *J* = 14.0 Hz, 1H), 3.94 (d, *J* = 14.0 Hz, 1H), 3.86 (s, 3H), 3.70 (s, 3H), 3.40 (dd, *J* = 15.8, 7.6 Hz, 1H), 3.29 (dd, *J* = 15.8, 6.9 Hz, 1H), 3.24-3.16 (m, 1H), 2.50 (s, 3H), 2.30 (s, 3H), 1.63-1.59 (m, 2H), 1.26-1.11 (m, 8H), 0.83 (t, *J* = 7.0 Hz, 3H); **^{13}C-NMR** (100 MHz, $CDCl_3$) δ 205.2, 151.1, 148.7, 148.1, 145.0, 141.7, 131.6, 130.0, 129.4, 128.2, 127.7, 126.0, 122.0, 120.8, 119.7, 111.5, 110.7, 60.6, 55.8, 55.6, 49.1, 41.8, 41.2, 36.4, 31.7, 29.2, 27.4, 22.6, 21.7, 14.1; **IR** (film, cm^{-1}) 2927, 1673, 1602, 1514, 1260, 1139, 1028; **MS** (ESI^+) *m/z* (%) 488 (100), 489 (32); **HRMS** (ESI^+) calc. for $C_{32}H_{42}O_3N$ $(M+H)^+$: 488.31592, found: 488.31512; The ee was determined by HPLC using a Chiralpak IC column [*n*-hexane/*i*-PrOH (97:3)]; flow rate 1.0 mL/min; τ_{major} = 34.53 min, τ_{minor} = 41.03 min.

1-{2-[(3,4-Dimethoxybenzyl)(methyl)amino]-4-(trifluoromethyl)phenyl}-3-phenylnonan-1-one (2ab)

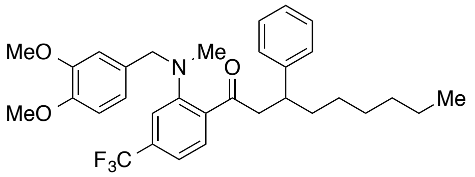

Following the general procedure and starting from 2-[(3,4-dimethoxybenzyl)(methyl)amino]-4-(trifluoromethyl) benzaldehyde (71 mg, 0.20 mmol), 1-octyne (38 μL, 0.26 mmol) and phenylboronic acid (48 mg, 0.40 mmol), the product was isolated by FC (petrol/ether 4:1) as a yellow oil.

General procedure A: 69% yield (75 mg, 0.14 mmol).

General procedure C: 52% yield (56 mg, 0.10 mmol). 97% ee (*S*). $[\alpha]_D^{25}$: −7.7 (*c* = 1.0, $CHCl_3$).

^{1}H-NMR (400 MHz, $CDCl_3$) δ 7.22-7.09 (m, 8H), 6.76 (d, *J* = 8.2 Hz, 1H), 6.66 (dd, *J* = 8.2, 1.9 Hz, 1H), 6.54 (d, *J* = 1.9 Hz, 1H), 4.03 (d, *J* = 14.1 Hz, 1H), 3.97 (d, *J* = 14.1 Hz, 1H), 3.86 (s, 3H), 3.71 (s, 3H), 3.39 (dd, *J* = 15.6, 8.0 Hz, 1H), 3.26-3.18 (m, 2H), 2.55 (s, 3H), 1.69-1.53 (m, 2H), 1.25-1.11 (m, 8H), 0.83 (t, *J* = 7.0 Hz, 3H); **^{13}C-NMR** (100 MHz, $CDCl_3$) δ 205.0, 150.8, 148.9, 148.4, 144.3, 137.0, 132.8 (q, $^2J_{CF}$ = 31.9 Hz), 129.4, 129.1, 128.3, 127.7, 126.3, 123.7 (q, $^1J_{CF}$ = 273.1 Hz), 120.8, 117.5 (q, $^3J_{CF}$ = 4.4 Hz), 115.6 (q, $^3J_{CF}$ = 3.1 Hz), 111.2, 110.8, 60.3, 55.8, 55.6, 49.0, 41.8, 41.0, 36.6, 31.7, 29.2, 27.4, 22.6, 14.1; **^{19}F-NMR** (377 MHz, $CDCl_3$) δ -63.0; **IR** (film, cm^{-1}) 2928, 1685, 1515, 1411, 1260, 1167, 1124, 1029, 700; **MS** (ESI^+) *m/z* (%) 542 (100), 543 (33), 564 (41); **HRMS** (ESI^+) calc. for $C_{32}H_{39}O_3NF_3$ $(M+H)^+$: 542.28766, found: 542.28680; The ee was determined by HPLC using a Chiralpak AD-H column [*n*-hexane/*i*-PrOH (97:3)]; flow rate 1.0 mL/min; τ_{major} = 14.98 min, τ_{minor} = 13.22 min.

1-{2-[(3,4-Dimethoxybenzyl)(methyl)amino]-4,5-dimethoxyphenyl}-3-phenylnonan-1-one (2ac)

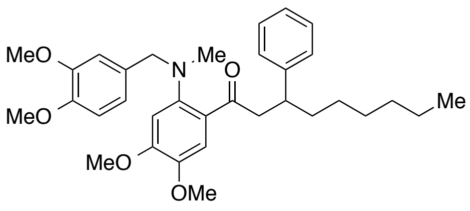

Following the general procedure and starting from 2-[(3,4-dimethoxybenzyl)(methyl)amino]-4,5-dimethoxybenzaldehyde (69 mg, 0.20 mmol), 1-octyne (38 μL, 0.26 mmol) and phenylboronic acid (48 mg, 0.40 mmol), the product was isolated by FC (petrol/ethyl acetate 3:1) as a yellow oil.

General procedure A: 79% yield (84 mg, 0.16 mmol).

General procedure C: 83% yield (89 mg, 0.17 mmol). 95% ee (*S*). $[\alpha]_D^{25}$: −3.4 (*c* = 1.0, $CHCl_3$).

^{1}H-NMR (400 MHz, $CDCl_3$) δ 7.22-7.18 (m, 2H), 7.14-7.08 (m, 3H), 6.75 (d, J = 8.2 Hz, 1H), 6.69-6.67 (m, 2H), 6.60 (d, J = 1.9 Hz, 1H), 6.49 (s, 1H), 3.99-3.91 (m, 2H), 3.85 (s, 3H), 3.83 (s, 3H), 3.75 (s, 3H), 3.71 (s, 3H), 3.46 (dd, J = 15.4, 8.1 Hz, 1H), 3.38 (dd, J = 15.4, 6.7 Hz, 1H), 3.19-3.15 (m, 1H), 2.55 (s, 3H), 1.65-1.55 (m, 2H), 1.26-1.08 (m, 8H), 0.82 (t, J = 7.0 Hz, 3H); **^{13}C-NMR** (100 MHz, $CDCl_3$) δ 204.7, 151.4, 148.8, 148.3, 146.4, 145.0, 144.3, 129.8, 128.3, 127.8, 127.5, 126.1, 121.2, 112.2, 111.9, 110.7, 103.8, 61.9, 56.1, 56.0, 55.9, 55.8, 49.3, 42.6, 42.1, 36.7, 31.8, 29.4, 27.5, 22.7, 14.2; **IR** (film, cm^{-1}) 2927, 1666, 1598, 1510, 1452, 1257, 1138, 1028; **MS** (ESI^+) *m/z* (%) 534 (100), 535 (38); **HRMS** (ESI^+) calc. for $C_{33}H_{44}O_5N$ $(M+H)^+$: 534.32140, found: 534.32056; The ee was determined by HPLC using a Chiralpak ID3 column [*n*-hexane/*i*-PrOH (85:15)]; flow rate 1.0 mL/min; τ_{major} = 32.07 min, τ_{minor} = 29.96 min.

1-{2-[(3,4-Dimethoxybenzyl)(methyl)amino]-5-fluorophenyl}-3-phenylnonan-1-one (2ad)

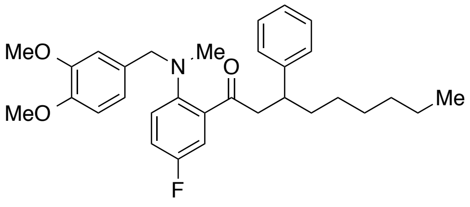

Following the general procedure and starting from 2-[(3,4-dimethoxybenzyl)(methyl)amino]-5-fluorobenzaldehyde (61 mg, 0.20 mmol), 1-octyne (38 μL, 0.26 mmol) and phenylboronic acid (48 mg, 0.40 mmol), the product was isolated by FC (petrol/ether 3:1) as a yellow oil.

General procedure A: 84% yield (83 mg, 0.17 mmol).

General procedure B: 66% yield (65 mg, 0.13 mmol). 74% ee (*S*).

General procedure C: 86% yield (85 mg, 0.17 mmol). 96% ee (*S*). $[\alpha]_D^{25}$: −5.1 (*c* = 1.0, $CHCl_3$).

^{1}H-NMR (400 MHz, $CDCl_3$) δ 7.22 (app. tt, J = 7.3, 1.5 Hz, 2H), 7.17-7.10 (m, 3H), 6.99 (ddd, J = 8.8, 7.7, 3.0 Hz, 1H), 6.91 (dd, J = 8.8, 4.6 Hz, 1H), 6.79-6.74 (m, 2H), 6.66 (dd, J = 8.1, 1.9 Hz, 1H), 6.56 (d, J = 1.9 Hz, 1H), 3.94-3.83 (m, 5H), 3.70 (s, 3H), 3.42 (dd, J = 16.0, 8.0 Hz, 1H), 3.29 (dd, J = 16.0, 6.6 Hz, 1H), 3.21-3.14 (m, 1H), 2.51 (s, 3H), 1.68-1.56 (m, 2H), 1.29-1.07 (m, 8H), 0.83 (t, J = 7.0 Hz, 3H); **^{13}C-NMR** (100 MHz, $CDCl_3$) δ 204.9, 158.0 (d, $^1J_{CF}$ = 243.0 Hz), 148.7, 148.3, 146.9, 144.5, 137.1 (d, $^3J_{CF}$ = 4.9 Hz), 129.4, 128.3, 127.7, 126.2, 121.4 (d, $^3J_{CF}$ = 6.7 Hz), 121.1, 117.5 (d,

$^2J_{CF}$ = 22.2 Hz), 115.2 (d, $^2J_{CF}$ = 23.4 Hz), 111.7, 110.7, 61.4, 55.8, 55.7, 49.4, 41.9, 41.7, 36.5, 31.7, 29.2, 27.4, 22.6, 14.1; **^{19}F-NMR** (377 MHz, $CDCl_3$) δ -120.9; **IR** (film, cm^{-1}) 2980, 2929, 1682, 1514, 1491, 1411, 1262, 1153, 1029; **MS** (ESI$^+$) *m/z* (%) 492 (100), 493 (37); **HRMS** (ESI$^+$) calc. for $C_{31}H_{39}O_3NF$ $(M+H)^+$: 492.29085, found: 492.29034; The ee was determined by HPLC using a Chiralpak AD-H column [*n*-hexane/*i*-PrOH (97:3)]; flow rate 1.0 mL/min; τ_{major} = 19.22 min, τ_{minor} = 21.62 min.

1-{2-[(3,4-Dimethoxybenzyl)(methyl)amino]phenyl}-6-methyl-3-phenylheptan-1-one (2ae)

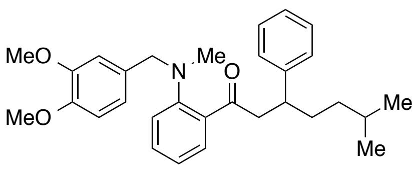

Following the general procedure and starting from 2-[(3,4-dimethoxybenzyl)(methyl)amino]benzaldehyde (57 mg, 0.20 mmol), 5-methylhex-1-yne (34 μL, 0.26 mmol) and phenylboronic acid (48 mg, 0.40 mmol), the product was isolated by FC (petrol/ether 4:1) as a yellow oil.

General procedure A: 96% yield (88 mg, 0.19 mmol).

General procedure C: 80% yield (74 mg, 0.16 mmol). 93% ee (*S*). $[\alpha]_D^{25}$: −2.5 (*c* = 1.0, $CHCl_3$).

^{1}H-NMR (400 MHz, $CDCl_3$) δ 7.29 (ddd, *J* = 8.2, 7.2, 1.7 Hz, 1H), 7.24-7.19 (m, 2H), 7.18-7.11 (m, 4H), 6.94-6.90 (m, 2H), 6.75 (d, *J* = 8.2 Hz, 1H), 6.67 (dd, *J* = 8.2, 1.9 Hz, 1H), 6.56 (d, *J* = 1.9 Hz, 1H), 4.00 (d, *J* = 14.0 Hz, 1H), 3.94 (d, *J* = 14.0 Hz, 1H), 3.85 (s, 3H), 3.69 (s, 3H), 3.42 (dd, *J* = 16.0, 7.7 Hz, 1H), 3.30 (dd, *J* = 16.0, 6.7 Hz, 1H), 3.21-3.15 (m, 1H), 2.53 (s, 3H), 1.70-1.55 (m, 2H), 1.49-1.42 (m, 1H), 1.13-1.04 (m, 1H), 1.01-0.91 (m, 1H), 0.79 (app. dd, *J* = 6.6, 5.1 Hz, 6H); **^{13}C-NMR** (100 MHz, $CDCl_3$) δ 206.0, 150.9, 148.8, 148.3, 145.0, 134.7, 131.3, 129.8, 129.2, 128.4, 127.8, 126.2, 121.4, 120.9, 119.3, 111.6, 110.8, 60.7, 55.9, 55.8, 49.3, 42.1, 41.3, 36.7, 34.3, 28.0, 22.8, 22.4; **IR** (film, cm^{-1}) 2929, 1678, 1592, 1514, 1449, 1260, 1028; **MS** (ESI$^+$) *m/z* (%) 460 (100), 461 (20), 482 (23); **HRMS** (ESI$^+$) calc. for $C_{30}H_{38}O_3N$ $(M+H)^+$: 460.28462, found: 460.28360; The ee was determined by HPLC using a Chiralpak IC column [*n*-hexane/*i*-PrOH (95:5)]; flow rate 1.0 mL/min; τ_{major} = 20.08 min, τ_{minor} = 22.61 min.

3-Cyclopropyl-1-{2-[(3,4-dimethoxybenzyl)(methyl)amino]phenyl}-3-phenylpropan-1-one (2af)

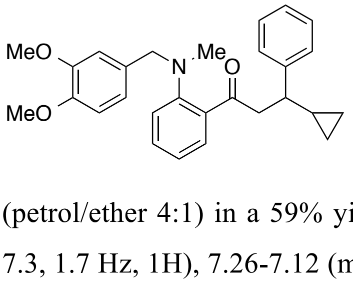

Following the general procedure A and starting from 2-[(3,4-dimethoxybenzyl)(methyl)amino]benzaldehyde (57 mg, 0.20 mmol), ethynylcyclopropane (22 μL, 0.26 mmol) and phenylboronic acid (48 mg, 0.40 mmol), the product (51 mg, 0.12 mmol) was isolated by FC (petrol/ether 4:1) in a 59% yield as a yellow oil. **^{1}H-NMR** (400 MHz, $CDCl_3$) δ 7.30 (ddd, *J* = 8.1, 7.3, 1.7 Hz, 1H), 7.26-7.12 (m, 6H), 6.95-6.90 (m, 2H), 6.75 (d, *J* = 8.1 Hz, 1H), 6.67 (dd, *J* = 8.1, 1.9 Hz, 1H), 6.55 (d, *J* = 1.9 Hz, 1H), 4.02 (d, *J* = 14.0 Hz, 1H), 3.95 (d, *J* = 14.0 Hz, 1H), 3.86 (s, 3H), 3.69 (s, 3H), 3.54 (app. dd, *J* = 7.3, 1.4 Hz, 2H), 2.59-2.51 (m, 4H), 1.06-0.97 (m, 1H), 0.57-0.50 (m,

1H), 0.42-0.35 (m, 1H), 0.31-0.25 (m, 1H), 0.16-0.10 (m, 1H); **^{13}C-NMR** (100 MHz, $CDCl_3$) δ 205.6, 150.8, 148.7, 148.2, 144.8, 134.5, 131.3, 129.7, 129.4, 128.2, 127.6, 126.2, 121.2, 120.8, 119.2, 111.5, 110.6, 60.7, 55.9, 55.7, 48.8, 46.7, 41.1, 17.6, 5.8, 4.3; **IR** (film, cm^{-1}) 2927, 1677, 1592, 1514, 1448, 1260, 1027; **MS** (ESI$^+$) *m/z* (%) 430 (100), 431 (35), 452 (26); **HRMS** (ESI$^+$) calc. for $C_{28}H_{32}O_3N$ $(M+H)^+$: 430.23767, found: 430.23712.

3-Cyclohexyl-1-{2-[(3,4-dimethoxybenzyl)(methyl)amino]phenyl}-3-phenylpropan-1-one (2ag)

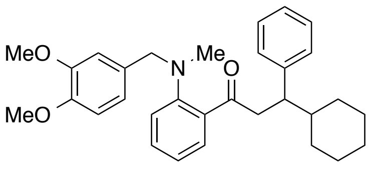

Following the general procedure A and starting from 2-[(3,4-dimethoxybenzyl)(methyl)amino]benzaldehyde (57 mg, 0.20 mmol), ethynylcyclohexane (34 µL, 0.26 mmol) and phenylboronic acid (48 mg, 0.40 mmol), the product (68 mg, 0.14 mmol) was isolated by FC (petrol/ether 4:1) in a 72% yield as a yellow oil. **^{1}H-NMR** (400 MHz, $CDCl_3$) δ 7.26 (ddd, *J* = 8.1, 7.3, 1.7 Hz, 1H), 7.18-7.13 (m, 2H), 7.12-7.07 (m, 1H), 7.05-7.03 (m, 2H), 7.00 (dd, *J* = 7.6, 1.6 Hz, 1H), 6.90 (dd, *J* = 8.2, 0.8 Hz, 1H), 6.86 (app. td, *J* = 7.4, 1.0 Hz, 1H), 6.75 (d, *J* = 8.2 Hz, 1H), 6.67 (dd, *J* = 8.2, 1.9 Hz, 1H), 6.54 (d, *J* = 1.9 Hz, 1H), 3.97 (d, *J* = 14.1 Hz, 1H), 3.92 (d, *J* = 14.1 Hz, 1H), 3.86 (s, 3H), 3.74 (s, 3H), 3.48 (dd, *J* = 15.9, 5.4 Hz, 1H), 3.40 (dd, *J* = 15.9, 9.3 Hz, 1H), 3.01 (ddd, *J* = 9.3, 7.8, 5.4 Hz, 1H), 2.51 (s, 3H), 1.87-1.84 (m, 1H), 1.75-1.71 (m, 1H), 1.64-1.58 (m, 2H), 1.50-1.41 (m, 2H), 1.26-1.19 (m, 1H), 1.11-1.04 (m, 2H), 1.00-0.90 (m, 1H), 0.81-0.77 (m, 1H); **^{13}C-NMR** (100 MHz, $CDCl_3$) δ 206.4, 150.6, 148.7, 148.1, 143.4, 134.6, 131.0, 129.7, 129.1, 128.6, 127.9, 126.0, 121.2, 120.8, 119.1, 111.6, 110.6, 60.8, 55.8, 55.7, 47.9, 45.6, 43.1, 40.9, 31.4, 30.7, 26.6, 26.44, 26.41; **IR** (film, cm^{-1}) 2924, 1678, 1592, 1514, 1448, 1260, 1028; **MS** (ESI$^+$) *m/z* (%) 472 (100), 473 (35), 494 (15); **HRMS** (ESI$^+$) calc. for $C_{31}H_{38}O_3N$ $(M+H)^+$: 472.28462, found: 472.28513.

4-Cyclohexyl-1-{2-[(3,4-dimethoxybenzyl)(methyl)amino]phenyl}-3-phenylbutan-1-one (2ah)

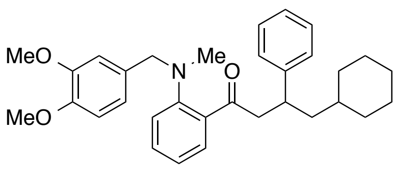

Following the general procedure and starting from 2-[(3,4-dimethoxybenzyl)(methyl)amino]benzaldehyde (57 mg, 0.20 mmol), prop-2-yn-1-ylcyclohexane (29 µL, 0.26 mmol) and phenylboronic acid (48 mg, 0.40 mmol), the product was isolated by FC (petrol/ether 4:1) as a yellow oil.

General procedure A: 85% yield (83 mg, 0.17 mmol).

General procedure B: 95% yield (92 mg, 0.19 mmol). 77% ee (*S*).

General procedure C: 85% yield (83 mg, 0.17 mmol). 98% ee (*S*). $[\alpha]_D^{25}$: −7.8 (*c* = 1.0, $CHCl_3$).

^{1}H-NMR (400 MHz, $CDCl_3$) δ 7.29 (ddd, *J* = 8.2, 7.2, 1.7 Hz, 1H), 7.23-7.19 (m, 2H), 7.15-7.11 (m, 4H), 6.93-6.89 (m, 2H), 6.75 (d, *J* = 8.2 Hz, 1H), 6.66 (dd, *J* = 8.2, 1.9 Hz, 1H), 6.56 (d, *J* = 1.9 Hz, 1H), 3.99 (d, *J* = 14.0 Hz, 1H), 3.94 (d, *J* = 14.0 Hz, 1H), 3.85 (s, 3H), 3.69 (s, 3H), 3.41-3.34 (m, 2H), 3.23 (dd, *J* = 14.7, 5.7 Hz, 1H), 2.52 (s, 3H), 1.82-1.77 (m, 1H), 1.65-1.44 (m, 6H), 1.09-1.05

(m, 4H), 0.86-0.83 (m, 2H); **^{13}C-NMR** (100 MHz, $CDCl_3$) δ 205.9, 150.8, 148.7, 148.2, 144.9, 134.6, 131.2, 129.7, 129.1, 128.3, 127.7, 126.1, 121.3, 120.8, 119.1, 111.5, 110.7, 60.7, 55.8, 55.7, 49.7, 44.2, 41.1, 38.7, 34.7, 34.1, 32.5, 26.6, 26.2, 26.1; **IR** (film, cm^{-1}) 2921, 1678, 1593, 1515, 1447, 1260, 1153, 1029; **MS** (ESI$^+$) *m/z* (%) 486 (100), 487 (33), 508 (11); **HRMS** (ESI$^+$) calc. for $C_{32}H_{39}O_3N$ (M+H)$^+$: 486.30027, found: 486.29932; The ee was determined by HPLC using a Chiralpak AD-H column [*n*-hexane/*i*-PrOH (90:10)]; flow rate 1.0 mL/min; τ_{major} = 16.13 min, τ_{minor} = 21.73 min.

1-{2-[(3,4-Dimethoxybenzyl)(methyl)amino]phenyl}-4,4-diethoxy-3-phenylbutan-1-one (2ai)

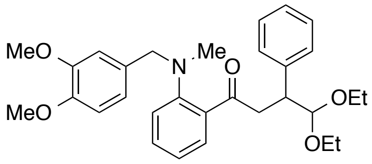

Following the general procedure A and starting from 2-[(3,4-dimethoxybenzyl)(methyl)amino]benzaldehyde (57 mg, 0.20 mmol), 3,3-diethoxyprop-1-yne (37 μL, 0.26 mmol) and phenylboronic acid (48 mg, 0.40 mmol), the product (67 mg, 0.14 mmol) was isolated by FC (gradient petrol/ether 85:15 to 7:3) in a 68% yield as a yellow oil. **^{1}H-NMR** (400 MHz, $CDCl_3$) δ 7.29-7.12 (m, 7H), 6.91-6.87 (m, 2H), 6.74 (d, *J* = 8.2 Hz, 1H), 6.65 (dd, *J* = 8.2, 1.9 Hz, 1H), 6.57 (d, *J* = 1.9 Hz, 1H), 4.55 (d, *J* = 5.2 Hz, 1H), 3.97 (d, *J* = 14.2 Hz, 1H), 3.88-3.85 (m, 4H), 3.73 (s, 3H), 3.71-3.48 (m, 5H), 3.44-3.36 (m, 2H), 2.48 (s, 3H), 1.15 (t, *J* = 7.0 Hz, 3H), 1.05 (t, *J* = 7.0 Hz, 3H); **^{13}C-NMR** (100 MHz, $CDCl_3$) δ 205.2, 150.6, 148.7, 148.1, 140.7, 134.2, 131.1, 129.9, 129.2, 129.0, 128.0, 126.5, 120.9, 120.7, 119.1, 111.5, 110.6, 105.6, 63.3, 62.8, 60.5, 55.8, 55.7, 45.4, 43.0, 41.0, 15.20, 15.17; **IR** (film, cm^{-1}) 1680, 1593, 1514, 1448, 1260, 1057, 1027; **MS** (ESI$^+$) *m/z* (%) 492 (100), 493 (35), 514 (15); **HRMS** (ESI$^+$) calc. for $C_{30}H_{38}O_5N$ (M+H)$^+$: 492.27445, found: 492.27313.

3-(Cyclohex-1-en-1-yl)-1-{2-[(3,4-dimethoxybenzyl)(methyl)amino]phenyl}-3-phenylpropan-1-one (2aj)

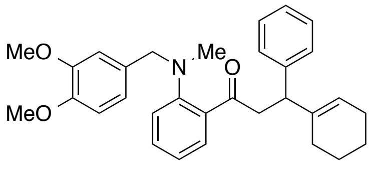

Following the general procedure A and starting from 2-[(3,4-dimethoxybenzyl)(methyl)amino]benzaldehyde (57 mg, 0.20 mmol), 1-ethynylcyclohex-1-ene (31 μL, 0.26 mmol) and phenylboronic acid (48 mg, 0.40 mmol), the product (62 mg, 0.13 mmol) was isolated by FC (petrol/ether 4:1) in a 66% yield as a yellow oil. **^{1}H-NMR** (400 MHz, $CDCl_3$) δ 7.31 (ddd, *J* = 8.2, 7.2, 1.7 Hz, 1H), 7.25-7.13 (m, 6H), 6.97-6.92 (m, 2H), 6.74 (d, *J* = 8.2 Hz, 1H), 6.69 (dd, *J* = 8.2, 1.9 Hz, 1H), 6.59 (d, *J* = 1.9 Hz, 1H), 5.55-5.53 (m, 1H), 4.06 (d, *J* = 13.9 Hz, 1H), 3.98 (d, *J* = 13.9 Hz, 1H), 3.86 (s, 3H), 3.79 (t, *J* = 7.7 Hz, 1H), 3.68 (s, 3H), 3.55 (dd, *J* = 15.5, 8.2 Hz, 1H), 3.47 (dd, *J* = 15.5, 7.3 Hz, 1H), 2.56 (s, 3H), 2.01-1.97 (m, 2H), 1.77-1.72 (m, 2H), 1.52-1.38 (m, 4H); **^{13}C-NMR** (100 MHz, $CDCl_3$) δ 205.8, 151.0, 148.9, 148.3, 143.6, 139.2, 134.7, 131.3, 129.9, 129.5, 128.3, 128.0, 126.3, 122.0, 121.4, 121.0, 119.1, 111.6, 110.8, 60.8, 56.0, 55.8, 48.9,

45.9, 41.5, 27.4, 25.4, 23.0, 22.5; **IR** (film, cm^{-1}) 2928, 1677, 1592, 1514, 1447, 1260, 1028; **MS** (ESI$^+$) *m/z* (%) 470 (100), 471 (31), 492 (30); **HRMS** (ESI$^+$) calc. for $C_{31}H_{36}O_3N$ (M+H)$^+$: 470.26897, found: 470.26816.

3-(Cyclohex-1-en-1-yl)-1-{2-[(3,4-dimethoxybenzyl)(methyl)amino]phenyl}-3-phenylpropan-1-one (2aj)

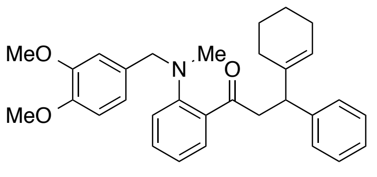

Following the general procedure and starting from 2-[(3,4-dimethoxybenzyl)(methyl)amino]benzaldehyde (57 mg, 0.20 mmol), ethynylbenzene (29 μL, 0.26 mmol) and cyclohex-1-en-1-ylboronic acid (50 mg, 0.40 mmol), the product was isolated by FC (petrol/ether 4:1) as a yellow oil.

General procedure A: 72% yield (68 mg, 0.14 mmol).

General procedure C: 88% yield (83 mg, 0.18 mmol). 92% ee (*S*). $[\alpha]_D^{25}$: −21.9 (*c* = 1.0, $CHCl_3$). The ee was determined by HPLC using a Chiralpak IA-3 column [*n*-hexane/*i*-PrOH (95:5)]; flow rate 1.0 mL/min; τ_{major} = 19.01 min, τ_{minor} = 20.65 min. Experimental data in agreement to the one reported above (compound **2aj**).

1-{2-[(3,4-Dimethoxybenzyl)(methyl)amino]phenyl}-3-phenyl-3-(*p*-tolyl)propan-1-one (2ak)

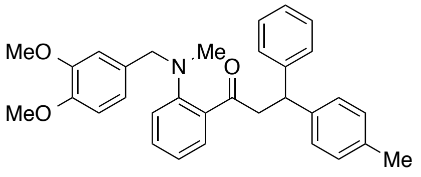

Following the general procedure and starting from 2-[(3,4-dimethoxybenzyl)(methyl)amino]benzaldehyde (57 mg, 0.20 mmol), 1-ethynyl-4-methylbenzene (33 μL, 0.26 mmol) and phenylboronic acid (48 mg, 0.40 mmol), the product was isolated by FC (petrol/ether 4:1) as a yellow oil.

General procedure A: 91% yield (87 mg, 0.18 mmol).

General procedure B: 96% yield (92 mg, 0.19 mmol). 43% ee (*S*).

General procedure C: 93% yield (89 mg, 0.19 mmol). >99% ee (*S*). $[\alpha]_D^{25}$: +3.9 (*c* = 1.0, $CHCl_3$).

^{1}H-NMR (400 MHz, $CDCl_3$) δ 7.33 (ddd, *J* = 8.2, 7.3, 1.7 Hz, 1H), 7.25-7.21 (m, 4H), 7.17-7.10 (m, 4H), 7.04 (d, *J* = 7.9 Hz, 2H), 6.98 (dd, *J* = 8.2, 0.7 Hz, 1H), 6.92 (app. td, *J* = 7.4, 0.9 Hz, 1H), 6.73 (d, *J* = 8.2 Hz, 1H), 6.67 (dd, *J* = 8.2, 1.9 Hz, 1H), 6.56 (d, *J* = 1.9 Hz, 1H), 4.68 (t, *J* = 7.7 Hz, 1H), 3.97 (s, 2H), 3.86 (s, 3H), 3.83 (dd, *J* = 7.7, 3.5 Hz, 2H), 3.64 (s, 3H), 2.54 (s, 3H), 2.28 (s, 3H); **^{13}C-NMR** (100 MHz, $CDCl_3$) δ 204.8, 150.9, 148.8, 148.2, 144.3, 141.1, 135.8, 134.5, 131.4, 129.8, 129.3, 129.2, 128.4, 127.9, 127.8, 126.2, 121.4, 120.9, 119.2, 111.5, 110.7, 60.6, 55.9, 55.6, 48.1, 46.4, 41.5, 21.0; **IR** (film, cm^{-1}) 2928, 1679, 1593, 1513, 1448, 1261, 1028; **MS** (ESI$^+$) *m/z* (%) 480 (100), 481 (34), 502 (32); **HRMS** (ESI$^+$) calc. for $C_{32}H_{34}O_3N$ (M+H)$^+$: 480.25332, found: 480.25250; The ee was determined by HPLC using a Chiralpak IC column [*n*-hexane/*i*-PrOH (93:7)]; flow rate 1.0 mL/min; τ_{major} = 28.53 min, τ_{minor} = 30.74 min.

1-{2-[(3,4-Dimethoxybenzyl)(methyl)amino]phenyl}-3-(4-methoxyphenyl)-3-phenylpropan-1-one (2al)

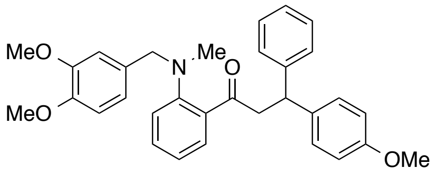

Following the general procedure and starting from 2-[(3,4-dimethoxybenzyl)(methyl)amino]benzaldehyde (57 mg, 0.20 mmol), 1-ethynyl-4-methoxybenzene (34 μL, 0.26 mmol) and phenylboronic acid (48 mg, 0.40 mmol), the product was isolated by FC (gradient petrol/ether 4:1 to 7:3) as a brown oil.

General procedure A: 95% yield (94 mg, 0.19 mmol).

General procedure C: 97% yield (96 mg, 0.19 mmol). >99% ee (*S*). $[\alpha]_D^{25}$: +2.0 (*c* = 1.0, $CHCl_3$).

^{1}H-NMR (400 MHz, $CDCl_3$) δ 7.32 (ddd, *J* = 8.2, 7.3, 1.7 Hz, 1H), 7.25-7.20 (m, 4H), 7.16-7.09 (m, 4H), 6.98 (dd, *J* = 8.2, 0.7 Hz, 1H), 6.91 (app. td, *J* = 7.4, 1.0 Hz, 1H), 6.78-6.72 (m, 3H), 6.66 (dd, *J* = 8.1, 1.9 Hz, 1H), 6.56 (d, *J* = 1.9 Hz, 1H), 4.65 (t, *J* = 7.7 Hz, 1H), 3.97 (s, 2H), 3.85 (s, 3H), 3.80 (d, *J* = 7.7 Hz, 2H), 3.74 (s, 3H), 3.63 (s, 3H), 2.53 (s, 3H); **^{13}C-NMR** (100 MHz, $CDCl_3$) δ 204.9, 158.1, 151.0, 148.9, 148.3, 144.6, 136.2, 134.5, 131.5, 129.9, 129.4, 129.0, 128.5, 127.9, 126.3, 121.5, 121.0, 119.3, 113.9, 111.6, 110.8, 60.7, 56.0, 55.7, 55.3, 48.3, 46.1, 41.5; **IR** (film, cm^{-1}) 2980, 1678, 1511, 1448, 1246, 1113, 1028; **MS** (ESI^+) *m/z* (%) 496 (100), 497 (32), 518 (5); **HRMS** (ESI^+) calc. for $C_{32}H_{34}O_4N$ $(M+H)^+$: 496.24824, found: 496.24796; The ee was determined by HPLC using a Chiralpak AD-H column [*n*-hexane/*i*-PrOH (90:10)]; flow rate 1.0 mL/min; τ_{major} = 49.66 min, τ_{minor} = 43.45 min.

Methyl 4-{3-{2-[(3,4-dimethoxybenzyl)(methyl)amino]phenyl}-3-oxo-1-phenylpropyl}benzoate (2am)

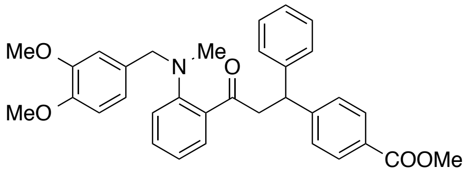

Following the general procedure and starting from 2-[(3,4-dimethoxybenzyl)(methyl)amino]benzaldehyde (57 mg, 0.20 mmol), methyl 4-ethynylbenzoate (42 mg, 0.26 mmol) and phenylboronic acid (48 mg, 0.40 mmol), the product was isolated by FC (gradient petrol/ether 4:1 to 3:2) as a yellow oil.

General procedure A: 47% yield (49 mg, 0.09 mmol).

General procedure C: 63% yield (66 mg, 0.13 mmol). >99% ee (*S*). $[\alpha]_D^{25}$: +13.9 (*c* = 1.0, $CHCl_3$).

^{1}H-NMR (400 MHz, $CDCl_3$) δ 7.91-7.88 (m, 2H), 7.34-7.14 (m, 8H), 7.11 (dd, *J* = 7.6, 1.7 Hz, 1H), 6.97 (d, *J* = 8.1 Hz, 1H), 6.92 (td, *J* = 7.4, 0.8 Hz, 1H), 6.71 (d, *J* = 8.2 Hz, 1H), 6.64 (dd, *J* = 8.2, 1.9 Hz, 1H), 6.52 (d, *J* = 1.9 Hz, 1H), 4.77 (t, *J* = 7.6 Hz, 1H), 3.95 (s, 2H), 3.87 (s, 3H), 3.85-3.83 (m, 5H), 3.62 (s, 3H), 2.51 (s, 3H); **^{13}C-NMR** (100 MHz, $CDCl_3$) δ 204.1, 166.9, 150.9, 149.3, 148.7, 148.2, 143.3, 134.2, 131.5, 129.8, 129.5, 129.2, 128.6, 128.2, 128.0, 127.9, 126.6, 121.5, 120.9, 119.3, 111.5, 110.7, 60.7, 55.8, 55.6, 52.0, 47.6, 46.6, 41.5; **IR** (film, cm^{-1}) 2981, 1718, 1679, 1593, 1514, 1448, 1278, 1261, 1106, 1027; **MS** (ESI^+) *m/z* (%) 524 (100), 525 (31); **HRMS** (ESI^+) calc. for

$C_{33}H_{34}O_5N$ $(M+H)^+$: 524.24315, found: 524.24274; The ee was determined by HPLC using a Chiralpak AD-H column [*n*-hexane/*i*-PrOH (80:20)]; flow rate 1.0 mL/min; τ_{major} = 49.48 min, τ_{minor} = 40.56 min.

1-{2-[(3,4-Dimethoxybenzyl)(methyl)amin]phenyl}-3-phenyl-3-(thiophen-3-yl)propan-1-one (2an)

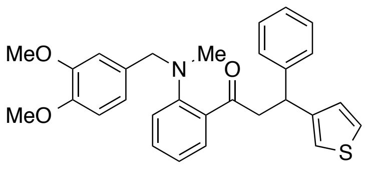

Following the general procedure and starting from 2-[(3,4-dimethoxybenzyl)(methyl)amino]benzaldehyde (57 mg, 0.20 mmol), 3-ethynylthiophene (26 μL, 0.26 mmol) and phenylboronic acid (48 mg, 0.40 mmol), the product was isolated by FC (gradient petrol/ether 85:15 to 4:1) as a yellow oil.

General procedure A: 51% yield (48 mg, 0.10 mmol).

General procedure B: 87% yield (82 mg, 0.17 mmol). 33% ee (*S*).

General procedure C: 82% yield (77 mg, 0.16 mmol). 97% ee (*S*). $[\alpha]_D^{25}$: +15.7 (*c* = 1.0, $CHCl_3$).

^{1}H-NMR (400 MHz, $CDCl_3$) δ 7.32 (ddd, *J* = 8.2, 7.3, 1.7 Hz, 1H), 7.25-7.14 (m, 6H), 7.11 (dd, *J* = 7.6, 1.7 Hz, 1H), 6.96 (dd, *J* = 8.2, 0.8 Hz, 1H), 6.94-6.89 (m, 2H), 6.87 (dd, *J* = 5.0, 1.3 Hz, 1H), 6.73 (d, *J* = 8.2 Hz, 1H), 6.66 (dd, *J* = 8.2, 1.9 Hz, 1H), 6.55 (d, *J* = 1.9 Hz, 1H), 4.74 (t, *J* = 7.6 Hz, 1H), 4.02-3.93 (m, 2H), 3.85 (s, 3H), 3.78 (dd, *J* = 7.6, 2.0 Hz, 2H), 3.64 (s, 3H), 2.52 (s, 3H); **^{13}C-NMR** (100 MHz, $CDCl_3$) δ 204.6, 150.9, 148.7, 148.2, 144.8, 143.8, 134.3, 131.4, 129.7, 129.2, 128.5, 127.9, 127.8, 126.4, 125.6, 121.4, 120.9, 120.5, 119.2, 111.5, 110.7, 60.6, 55.9, 55.6, 48.5, 42.5, 41.4; **IR** (film, cm^{-1}) 1678, 1592, 1514, 1448, 1260, 1139, 1027; **MS** (ESI^+) *m/z* (%) 472 (100), 473 (31), 494 (9); **HRMS** (ESI^+) calc. for $C_{29}H_{30}O_3NS$ $(M+H)^+$: 472.19409, found: 472.19354; The ee was determined by HPLC using a Chiralpak AS-H column [*n*-hexane/*i*-PrOH (93:7)]; flow rate 1.0 mL/min; τ_{major} = 17.73 min, τ_{minor} = 13.39 min.

1-{2-[(3,4-Dimethoxybenzyl)(methyl)amino]phenyl}-3-phenyl-3-(thiophen-3-yl)propan-1-one (2an)

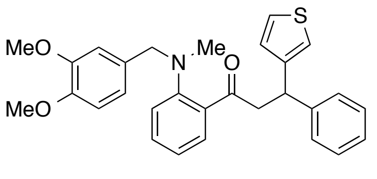

Following the general procedure and starting from 2-[(3,4-dimethoxybenzyl)(methyl)amino]-4-methylbenzaldehyde (57 mg, 0.20 mmol), ethynylbenzene (29 μL, 0.26 mmol) and thiophen-3-ylboronic acid (51 mg, 0.40 mmol), the product was isolated by FC (gradient petrol/ether 85:15 to 4:1) as a yellow oil.

General procedure A: 83% yield (78 mg, 0.17 mmol).

General procedure C: 36% yield (34 mg, 0.06 mmol). 98% ee (*R*). $[\alpha]_D^{25}$: −18.7 (*c* = 1.0, $CHCl_3$). The ee was determined by HPLC using a Chiralpak AS-H column [*n*-hexane/*i*-PrOH (93:7)]; flow rate 1.0

mL/min; τ_{major} = 13.39 min, τ_{minor} = 17.73 min. Experimental data in agreement to the one reported above (compound **2an**).

1-{2-[(3,4-Dimethoxybenzyl)(methyl)amino]phenyl}-3-(5-methoxypyridin-2-yl)-3-(*p*-tolyl)propan-1-one (2ao)

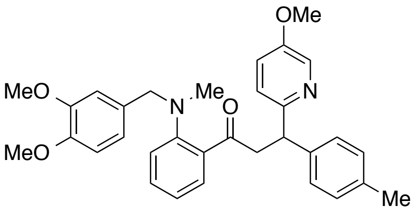

Following the general procedure A and starting from 2-[(3,4-dimethoxybenzyl)(methyl)amino]benzaldehyde (57 mg, 0.20 mmol), 1-ethynyl-4-methylbenzene (33 μL, 0.26 mmol) and (5-methoxypyridin-2-yl)boronic acid (61 mg, 0.40 mmol), the product (68 mg, 0.13 mmol) was isolated by FC (petrol/ethyl acetate 4:1) in a 67% yield as a yellow oil. **^{1}H-NMR** (400 MHz, $CDCl_3$) δ 8.03 (d, J = 2.5 Hz, 1H), 7.37 (dd, J = 8.6, 2.5 Hz, 1H), 7.32 (ddd, J = 8.2, 7.3, 1.7 Hz, 1H), 7.12 (dd, J = 7.5, 1.7 Hz, 1H), 7.09-7.03 (m, 4H), 6.96 (dd, J = 8.2, 0.7 Hz, 1H), 6.92 (app. td, J = 7.5, 1.0 Hz, 1H), 6.72 (d, J = 8.2 Hz, 1H), 6.64 (dd, J = 8.2, 1.9 Hz, 1H), 6.59 (dd, J = 8.6, 0.4 Hz, 1H), 6.52 (d, J = 1.9 Hz, 1H), 4.59 (t, J = 7.7 Hz, 1H), 3.96 (s, 2H), 3.87 (s, 3H), 3.84 (s, 3H), 3.78 (d, J = 7.7 Hz, 2H), 3.64 (s, 3H), 2.54 (s, 3H), 2.27 (s, 3H); **^{13}C-NMR** (100 MHz, $CDCl_3$) δ 204.3, 162.8, 150.9, 148.7, 148.2, 145.7, 140.5, 138.4, 136.1, 134.2, 132.4, 131.5, 129.6, 129.28, 129.26, 127.6, 121.5, 120.9, 119.3, 111.5, 110.7, 110.6, 60.8, 55.8, 55.6, 53.3, 47.7, 43.2, 41.3, 20.9; **IR** (film, cm^{-1}) 2927, 1678, 1593, 1513, 1490, 1446, 1259, 1026; **MS** (ESI^{+}) *m/z* (%) 511 (100), 512 (36), 534 (9); **HRMS** (ESI^{+}) calc. for $C_{32}H_{35}O_4N_2$ $(M+H)^{+}$: 511.25913, found: 511.25815.

***N*-{3-{3-{2-[(3,4-Dimethoxybenzyl)(methyl)amino]phenyl}-1-(4-methoxyphenyl)-3-oxopropyl} phenyl}acetamide (2ap)**

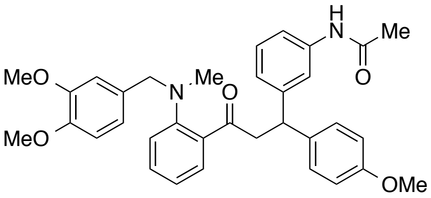

Following the general procedure and starting from 2-[(3,4-dimethoxybenzyl)(methyl)amino]-4-methylbenzaldehyde (57 mg, 0.20 mmol), 1-ethynyl-4-methoxybenzene (34 μL, 0.26 mmol) and 3-acetamidophenylboronic acid (73 mg, 0.40 mmol), the product was isolated by FC (gradient petrol/ethyl acetate 3:2 to 3:7) as a yellow oil.

General procedure A: 93% yield (103 mg, 0.19 mmol).

General procedure C: 30% yield (33 mg, 0.06 mmol). 94% ee (*R*). $[\alpha]_D^{25}$: −0.5 (*c* = 1.0, $CHCl_3$).

^{1}H-NMR (400 MHz, $CDCl_3$) δ 7.44 (dd, J = 8.0, 1.3 Hz, 1H), 7.31 (ddd, J = 8.2, 7.3, 1.7 Hz, 1H), 7.22 (bs, 1H), 7.18-7.14 (m, 2H), 7.11-7.08 (m, 3H), 6.99-6.88 (m, 3H), 6.75-6.72 (m, 3H), 6.66 (dd, J = 8.1, 1.9 Hz, 1H), 6.57 (d, J = 1.9 Hz, 1H), 4.61 (t, J = 7.6 Hz, 1H), 3.95 (d, J = 3.8 Hz, 2H), 3.84 (s, 3H), 3.76-3.73 (m, 5H), 3.63 (s, 3H), 2.51 (s, 3H), 2.11 (s, 3H); **^{13}C-NMR** (100 MHz, $CDCl_3$) δ 204.8, 168.4, 158.1, 151.0, 148.8, 148.3, 145.5, 138.2, 135.9, 134.4, 131.5, 130.0, 129.3, 129.2, 129.0, 123.8, 121.5, 121.1, 119.3, 119.1, 118.0, 113.9, 111.8, 110.8, 60.6, 55.9, 55.8, 55.3, 48.1, 45.9, 41.6,

24.6; **IR** (film, cm^{-1}) 3242, 2934, 1672, 1593, 1512, 1255, 1029; **MS** (ESI^{+}) *m/z* (%) 553 (100), 554 (35); **HRMS** (ESI^{+}) calc. for $C_{34}H_{37}O_5N_2$ $(M+H)^+$: 553.26970, found: 553.26862; The ee was determined by HPLC using a Chiralpak AD-H column [*n*-hexane/*i*-PrOH (80:20)]; flow rate 1.0 mL/min; τ_{major} = 31.17 min, τ_{minor} = 42.19 min.

1-{2-[(3,4-Dimethoxybenzyl)(methyl)amino]-4-(trifluoromethyl)phenyl}-3-(naphthalen-2-yl)-3-(*p*-tolyl)propan-1-one (2aq)

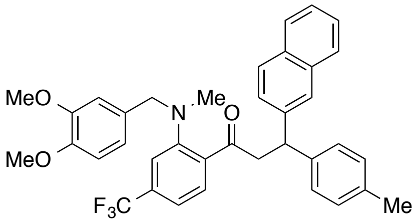

Following the general procedure and starting from 2-[(3,4-dimethoxybenzyl)(methyl)amino]-4-(trifluoromethyl) benzaldehyde (71 mg, 0.20 mmol), 1-ethynyl-4-methylbenzene (33 μL, 0.26 mmol) and naphthalen-2-ylboronic acid (69 mg, 0.40 mmol), the product was isolated by FC (petrol/ether 4:1) as a yellow oil.

General procedure A: 83% yield (99 mg, 0.17 mmol).

General procedure C: 52% yield (62 mg, 0.10 mmol). 95% ee (*R*). $[\alpha]_D^{25}$: −12.1 (*c* = 1.0, $CHCl_3$).

^{1}H-NMR (400 MHz, $CDCl_3$) δ 7.77-7.68 (m, 3H), 7.64 (d, *J* = 1.0 Hz, 1H), 7.46-7.40 (m, 2H), 7.30 (dd, *J* = 8.5, 1.8 Hz, 1H), 7.21 (s, 1H), 7.15-7.04 (m, 6H), 6.67 (d, *J* = 8.2 Hz, 1H), 6.61 (dd, *J* = 8.2, 1.9 Hz, 1H), 6.51 (d, *J* = 1.9 Hz, 1H), 4.81 (t, *J* = 7.7 Hz, 1H), 3.98 (s, 2H), 3.92 (dd, *J* = 16.2, 7.8 Hz, 1H), 3.86-3.81 (m, 4H), 3.57 (s, 3H), 2.55 (s, 3H), 2.28 (s, 3H); **^{13}C-NMR** (100 MHz, $CDCl_3$) δ 204.0, 151.0, 148.9, 148.4, 141.3, 140.5, 136.9, 136.1, 133.4, 133.0 (q, $^2J_{CF}$ = 32.0 Hz), 132.2, 129.8, 129.3, 129.0, 128.3, 127.8, 127.7, 127.5, 126.6, 126.1, 125.8, 125.6, 123.7 (q, $^1J_{CF}$ = 273.5 Hz), 120.8, 117.6 (q, $^3J_{CF}$ = 4.4 Hz), 115.6 (q, $^3J_{CF}$ = 3.4 Hz), 111.2, 110.8, 60.3, 55.8, 55.5, 47.8, 46.5, 41.3, 21.0; **^{19}F-NMR** (377 MHz, $CDCl_3$) δ -62.9; **IR** (film, cm^{-1}) 2978, 1685, 1514, 1412, 1261, 1126, 1028; **MS** (ESI^{+}) *m/z* (%) 598 (100), 599 (38), 620 (13); **HRMS** (ESI^{+}) calc. for $C_{37}H_{35}O_3NF_3$ $(M+H)^+$: 598.25636, found: 598.25616; The ee was determined by HPLC using a Chiralpak AD-H column [*n*-hexane/*i*-PrOH (98:2)]; flow rate 1.0 mL/min; τ_{major} = 82.94 min, τ_{minor} = 88.27 min.

1-[2-(Dimethylamino)phenyl]-3-(4-methoxyphenyl)-3-(naphthalen-1-yl)propan-1-one (2ar)

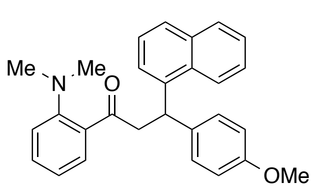

Following the general procedure and starting from 2-(dimethylamino)-4-methylbenzaldehyde (30 mg, 0.20 mmol), 1-ethynyl-4methoxybenzene (34 μL, 0.26 mmol) and naphthalen-1-ylboronic acid (69 mg, 0.40 mmol), the product was isolated by FC (petrol/ether 85:15) as a yellow oil.

General procedure A: 48% yield (39 mg, 0.10 mmol).

General procedure C (4 equiv. of boronic acid): 71% yield (58 mg, 0.14 mmol). >99% ee (*R*). $[\alpha]_D^{25}$: −4.8 (*c* = 1.0, $CHCl_3$).

^{1}**H-NMR** (400 MHz, $CDCl_3$) *δ* 8.18-8.16 (m, 1H), 7.83-7.82 (m, 1H), 7.70 (dd, *J* = 7.7, 1.2 Hz, 1H), 7.47-7.42 (m, 2H), 7.40-7.29 (m, 3H), 7.21-7.19 (m, 2H), 7.05 (dd, *J* = 7.7, 1.7 Hz, 1H), 6.99 (dd, *J* = 8.2, 0.6 Hz, 1H), 6.82 (app. td, *J* = 7.4, 0.9 Hz, 1H), 6.78-6.75 (m, 2H), 5.46 (dd, *J* = 8.6, 6.6 Hz, 1H), 3.89 (dd, *J* = 16.4, 8.6 Hz, 1H), 3.80 (dd, *J* = 16.4, 6.6 Hz, 1H), 3.74 (s, 3H), 2.74 (s, 6H); ^{13}**C-NMR** (100 MHz, $CDCl_3$) *δ* 205.1, 158.0, 151.6, 140.1, 136.0, 134.1, 133.5, 131.7, 131.6, 129.4, 129.3, 128.8, 127.2, 126.1, 125.5, 125.3, 124.7, 124.0, 120.8, 117.2, 113.9, 55.3, 48.2, 44.7, 41.6; **IR** (film, cm^{-1}) 2935, 1678, 1594, 1510, 1249, 1180, 1034; **MS** (ESI^+) *m/z* (%) 410 (100), 411 (29); **HRMS** (ESI^+) calc. for $C_{28}H_{28}O_2N$ $(M+H)^+$: 410.21146, found: 410.21068; The ee was determined by HPLC using a Chiralpak AD-H column [*n*-hexane/*i*-PrOH (97:3)]; flow rate 1.0 mL/min; τ_{major} = 37.82 min, τ_{minor} = 46.06 min.

1-[2-(Dimethylamino)phenyl]-3-phenylheptan-1-one (2as)

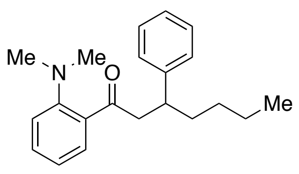

Following the general procedure and starting from 2-(dimethylamino)-4-methylbenzaldehyde (30 mg, 0.20 mmol), 1-hexyne (30 μL, 0.26 mmol) and phenylboronic acid (48 mg, 0.40 mmol), the product was isolated by FC (petrol/ether 9:1) as a yellow oil.

General procedure A: 86% yield (53 mg, 0.17 mmol).

General procedure C (0.3 mmol scale, 4 equiv. of boronic acid): 85% yield (79 mg, 0.26 mmol). 97% ee (*S*). $[\alpha]_D^{25}$: −6.0 (*c* = 1.0, $CHCl_3$).

^{1}**H-NMR** (400 MHz, $CDCl_3$) *δ* 7.31 (ddd, *J* = 8.2, 7.3, 1.6 Hz, 1H), 7.25-7.21 (m, 2H), 7.16-7.12 (m, 4H), 6.95 (d, *J* = 8.2 Hz, 1H), 6.86 (app. td, *J* = 7.4, 0.8 Hz, 1H), 3.33 (dd, *J* = 15.8, 8.0 Hz, 1H), 3.27-3.13 (m, 2H), 2.64 (s, 6H), 1.68-1.55 (m, 2H), 1.30-1.06 (m, 4H), 0.81 (t, *J* = 7.2 Hz, 3H); ^{13}**C-NMR** (100 MHz, $CDCl_3$) *δ* 205.9, 151.5, 144.9, 133.5, 131.3, 129.2, 128.2, 127.8, 126.0, 120.6, 117.0, 48.7, 44.5, 41.8, 36.2, 29.6, 22.6, 14.0; **IR** (film, cm^{-1}) 2927, 1677, 1594, 1490, 1542, 946; **MS** (ESI^+) *m/z* (%) 310 (100), 311 (25), 332 (26); **HRMS** (ESI^+) calc. for $C_{21}H_{28}ON$ $(M+H)^+$: 310.21654, found: 310.21643; The ee was determined by HPLC using a Chiralpak AD-H column [*n*-hexane/*i*-PrOH (99:1)]; flow rate 1.0 mL/min; τ_{major} = 18.18 min, τ_{minor} = 16.45 min.

Determination of the Absolute Configuration

The absolute configuration was determined by derivatization of two of the synthesized ketones into compounds known in the literature. The stereochemistry for the remaining products was assigned by analogy. The stereochemical outcome obtained for the reaction is in agreement with previous reports from the literature.[6]

Ruthenium catalysed reductive deamination of the *N,N*-dimethylaniline **2as** led to the formation of 1,3-diphenylheptan-1-one, known in the literature, allowing the determination of its absolute stereostructure (Scheme 1a). In a similar way, DMB deprotection of product **2ak** and subsequent deamination provided the known compound 1,3-diphenyl-3-(*p*-tolyl)propan-1-one (Scheme 1b).

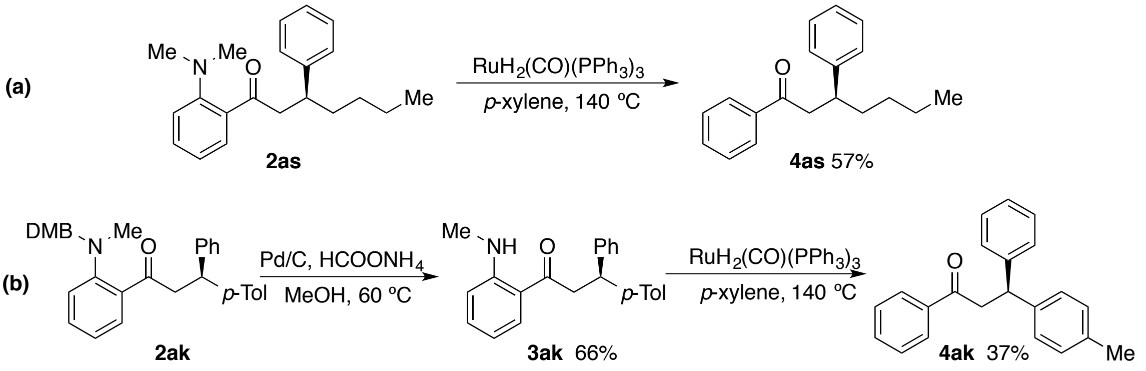

Scheme 1

(*S*)-1,3-Diphenylheptan-1-one (4as)[7]

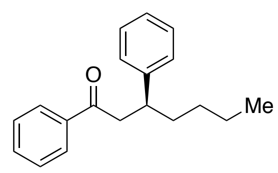

Prepared following a procedure adapted from Koreeda et al.[8] To an oven dried reaction tube containing a magnetic stirrer were added $RuH_2(CO)(PPh_3)_3$ (107 mg, 0.12 mmol, 50 mol%) and the *N,N*-dimethylaniline (-)-**2as** (73 mg, 0.23 mmol) and the flask was backfilled with N_2 prior to dissolving in *p*-xylene (0.35 mL). The reaction was heated at 140 ºC for 18 h, and then allowed to cool down to room temperature and filtered through a small pad of silica. Solvents were evaporated and the crude material was directly loaded onto silica. Flash chromatography (petrol/ether 19:1) afforded the title compound as a light yellow solid in 57% yield (35 mg, 0.13 mmol). **^{1}H-NMR** (400 MHz, $CDCl_3$) δ 7.92-7.89 (m, 2H), 7.56-7.51 (m, 1H), 7.45-7.41 (m, 2H), 7.31-7.27 (m, 2H), 7.24 (app. dt, J = 8.1, 1.8 Hz, 2H), 7.21-7.16 (m, 1H), 3.35-3.21 (m, 3H), 1.77-1.61 (m, 2H), 1.34-1.10 (m, 4H), 0.83 (t, J = 7.0 Hz, 3H); **^{13}C-NMR** (100 MHz, $CDCl_3$) δ 199.3,

---

[6] (a) Pattison, G.; Piraux, G.; Lam, H. W. *J. Am. Chem. Soc.* **2010**, *132*, 14373; (b) Saxena, A.: Lam, H. W. *Chem. Sci.* **2011**, *2*, 2326; (c) Roy, I. D.; Burns, A. R.; Pattison, G.; Michel, B.; Parker, A. J.; Lam, H. W. *Chem. Commun.* **2014**, *50*, 2865; (b) Le Nôtre, J.; Allen, J. C. Frost; C. G. *Chem. Commun.* **2008**, 3795.

[7] (a) Huttenloch, O.; Spieler, J.; Waldmann, H. *Chem. Eur. J.* **2001**, *7*, 671; (b) Endo, K.; Ogawa, M.; Shibata, T. *Angew. Chem. Int. Ed.* **2010**, *49*, 2410; (c) Turner, H. M.; Patel, J.; Niljianskul, N.; Chong, J. M. *Org. Lett.* **2011**, *13*, 5796; (d) Tseng, C.-H.; Hung, Y.-M.; Uang, B.-J. *Tetrahedron Asymmetry* **2012**, *23*, 130.

[8] Koreeda, T.; Kochi, T.; Kakiuchi, F. *J. Organometallic Chem.* **2013**, *741*, 148.

145.1, 137.4, 133.0, 128.6, 128.5, 128.2, 127.7, 126.4, 46.1, 41.4, 36.2, 29.8, 22.8, 14.1; **IR** (film, cm^{-1}) 2928, 1685, 1598, 1449; **MS** (ESI$^+$) *m/z* (%) 267 (62), 289 (100); The ee was determined by HPLC using a Chiralpak AD-H column [*n*-hexane/*i*-PrOH (99:1)]; flow rate 1.0 mL/min; τ_{major} = 10.50 min, τ_{minor} = 14.61 min.[7b,d] 96% ee. $[\alpha]_D^{25}$: +8.9 (*c* = 2.5, CCl_4) {ref.[7a]: $[\alpha]_D^{20}$: +14.8 (*c* = 2.6, CCl_4) for *S* enantiomer with 81% ee}. These data are consistent with the previously reported values.[7]

(*S*)-1-[2-(Methylamino)phenyl]-3-phenyl-3-(*p*-tolyl)propan-1-one (3ak)

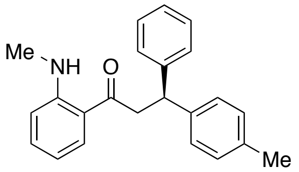

A solution of the bis-protected aniline (-)-**2ak** (100 mg, 0.21 mmol) in MeOH (2 mL) was stirred with activated Pd/C (45 mg, 10%) and ammonium formate (14 mg, 0.21 mmol) at 60 ºC for 15 h. The mixture was cooled down to room temperature and then filtered through Celite®. Solvents were removed under reduced pressure and the crude residue was redissolved in CH_2Cl_2 (10 mL) and washed with water (10 mL). After drying the organic phase ($MgSO_4$), the crude material was purified by flash chromatography (petrol/ether 4:1) to afford the title compound as a yellow oil in 66% yield (45 mg, 0.14 mmol). **^{1}H-NMR** (400 MHz, $CDCl_3$) δ 8.74 (bq, *J* = 4.3 Hz, 1H), 7.86 (dd, *J* = 8.1, 1.5 Hz, 1H), 7.38 (dddd, *J* = 8.6, 7.0, 1.5, 0.5 Hz, 1H), 7.30-7.25 (m, 4H), 7.19-7.15 (m, 3H), 7.10-7.08 (m, 2H), 6.67 (dd, *J* = 8.6, 0.8 Hz, 1H), 6.59 (ddd, *J* = 8.1, 7.0, 1.1 Hz, 1H), 4.76 (t, *J* = 7.3 Hz, 1H), 3.72 (d, *J* = 7.3 Hz, 2H), 2.85 (d, *J* = 5.1 Hz, 3H), 2.30 (s, 3H); **^{13}C-NMR** (100 MHz, $CDCl_3$) δ 200.0, 152.1, 144.8, 141.5, 135.7, 135.0, 131.5, 129.2, 128.5, 127.8, 127.7, 126.2, 117.2, 113.8, 111.4, 45.8, 44.9, 29.3, 21.0; **IR** (film, cm^{-1}) 3327, 2921, 1635, 1571, 1519, 1424, 1252, 1165; **MS** (ESI$^+$) *m/z* (%) 330 (100), 331 (26), 352 (26); **HRMS** (ESI$^+$) calc. for $C_{23}H_{24}ON$ $(M+H)^+$: 330.18524, found: 330.18563; The ee was determined by HPLC using a Chiralpak AD-H column [*n*-hexane/*i*-PrOH (98:2)]; flow rate 0.5 mL/min; τ_{major} = 25.49 min, τ_{minor} = 22.19 min. 98% ee. $[\alpha]_D^{25}$: −9.6 (*c* = 1.0, $CHCl_3$).

(*S*)-1,3-Diphenyl-3-(*p*-tolyl)propan-1-one (4ak)[7c,9]

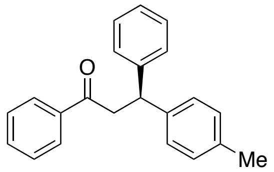

Prepared following a procedure adapted from Koreeda et al.[8] To an oven dried reaction tube containing a magnetic stirrer were added $RuH_2(CO)(PPh_3)_3$ (126 mg, 0.14 mmol, 50 mol%) and the *N*-methylaniline (-)-**3ak** (90 mg, 0.27 mmol) and the flask was backfilled with N_2 prior to dissolving in *p*-xylene (0.40 mL). The reaction was heated at 140 ºC for 18 h, and then allowed to cool down to room temperature and filtered through a small pad of silica. Solvents were evaporated and the crude material was directly loaded onto silica. Flash chromatography (DCM/Hexane 1:1 then re-column in pure toluene) afforded the title compound as a white solid in 37% yield (30 mg, 0.10

[9] (a) Chen, G.; Gui, J.; Li, L.; Liao, J. *Angew. Chem. Int. Ed.* **2011**, *50*, 7681; (b) Wong, J.; Gan, K.; Chen, H. J.; Pullarkat, S. A. *Adv. Synth. Catal.* **2014**, *356*, 3391.

mmol). **^{1}H-NMR** (400 MHz, $CDCl_3$) δ 7.98-7.92 (m, 2H), 7.58-7.53 (m, 1H), 7.48-7.42 (m, 2H), 7.28 (m, 4H), 7.23-7.13 (m, 3H), 7.10 (d, J = 7.8 Hz, 2H), 4.81 (t, J = 7.3 Hz, 1H), 3.74 (d, J = 7.3 Hz, 2H), 2.30 (s, 3H); **^{13}C-NMR** (100 MHz, $CDCl_3$) δ 198.1, 144.4, 141.2, 137.1, 135.9, 133.1, 129.3, 128.6, 128.6, 128.1, 127.8, 127.7, 126.3, 45.6, 44.8, 21.0; **IR** (film, cm^{-1}) 2919, 1686, 1449; **MS** (ESI^{+}) *m/z* (%) 181 (90), 323 (100), 324 (64); The ee was determined by HPLC using a Chiralpak AD-H column [*n*-hexane/*i*-PrOH (98:2)]; flow rate 0.5 mL/min; τ_{major} = 35.03 min, τ_{minor} = 29.05 min.[9b] 98% ee. $[\alpha]_D^{25}$: +6.3 (c = 1.0, $CHCl_3$) {ref.[7c]: $[\alpha]_D^{25}$: −2.7 (c = 1.0, $CHCl_3$) for *R* enantiomer with 71% ee}. These data are consistent with the previously reported values.[7c,9]

NMR spectra for organic compounds

^{1}H NMR, 400 MHz, $CDCl_3$

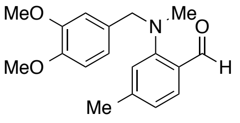

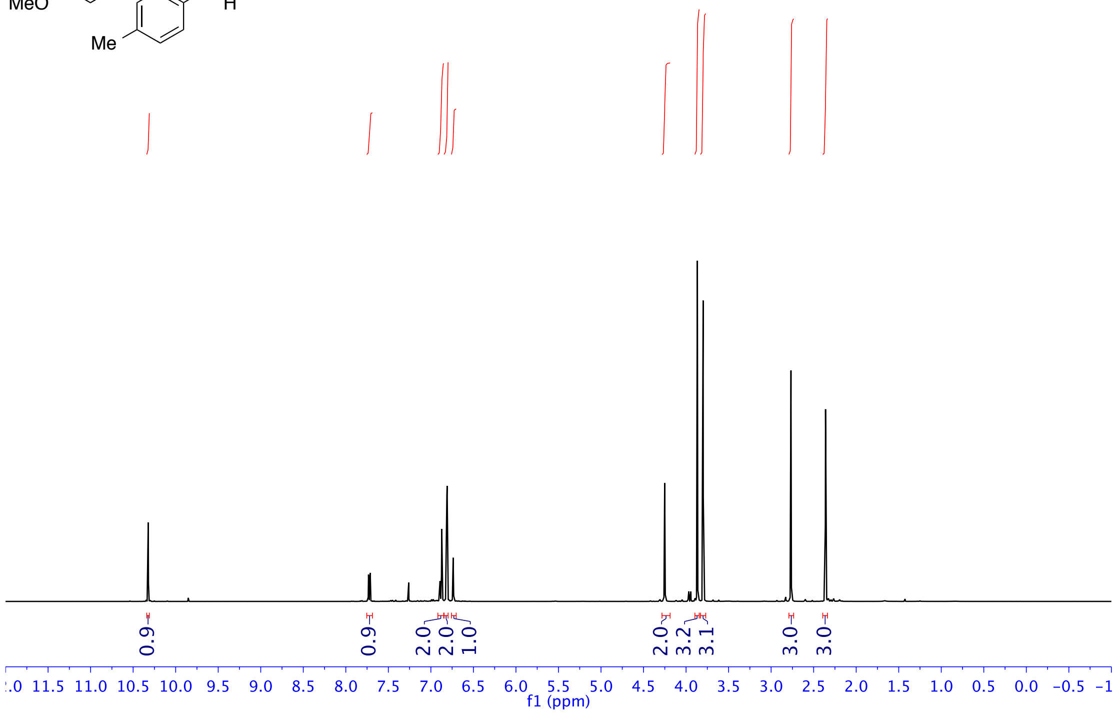

^{13}C NMR, 100 MHz, $CDCl_3$

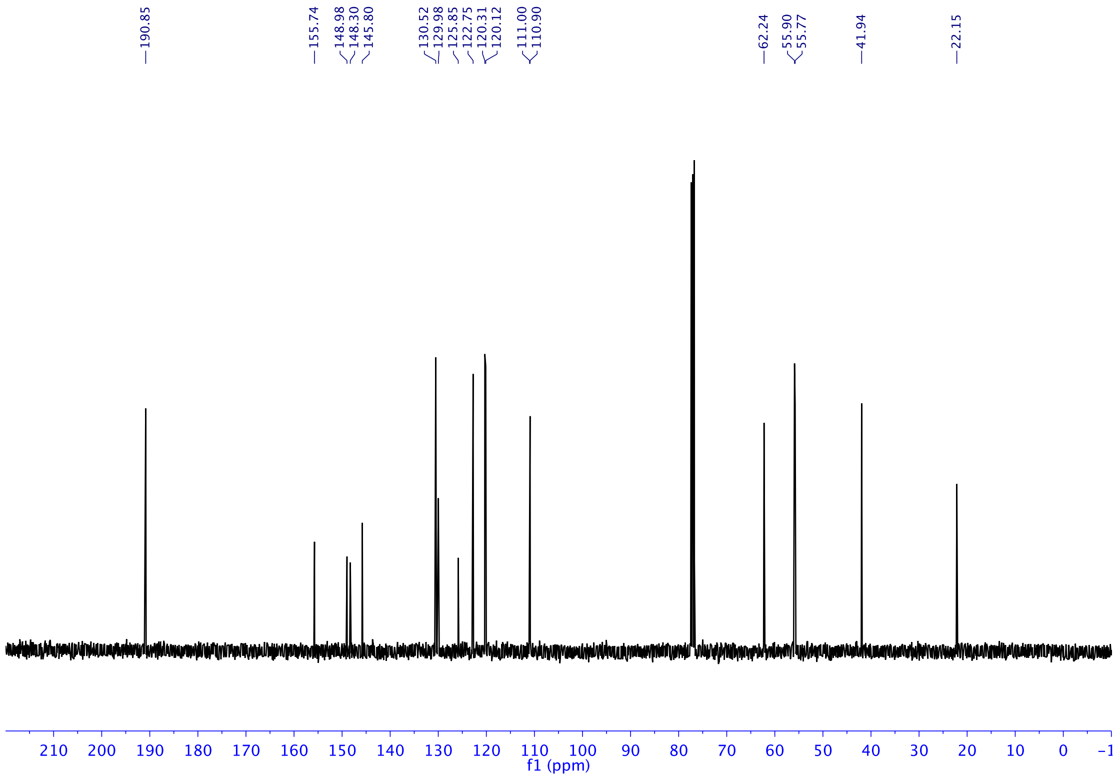

Figure 1. ^{1}H-NMR and ^{13}C-NMR spectra of compound **1g**

^{1}H NMR, 400 MHz, $CDCl_3$

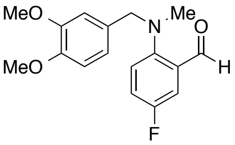

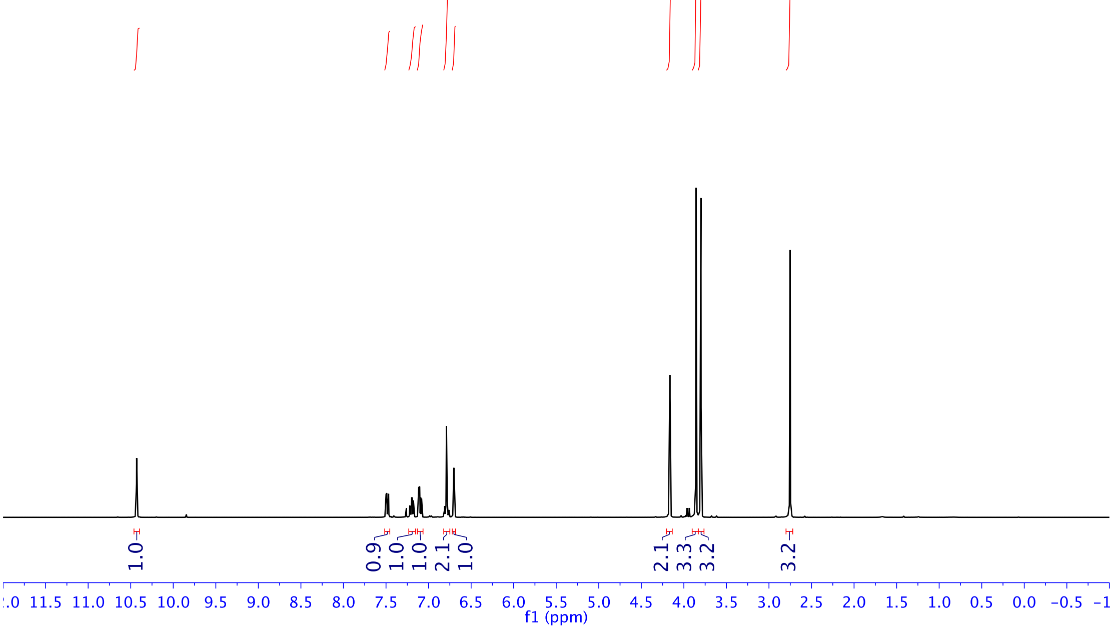

^{13}C NMR, 100 MHz, $CDCl_3$

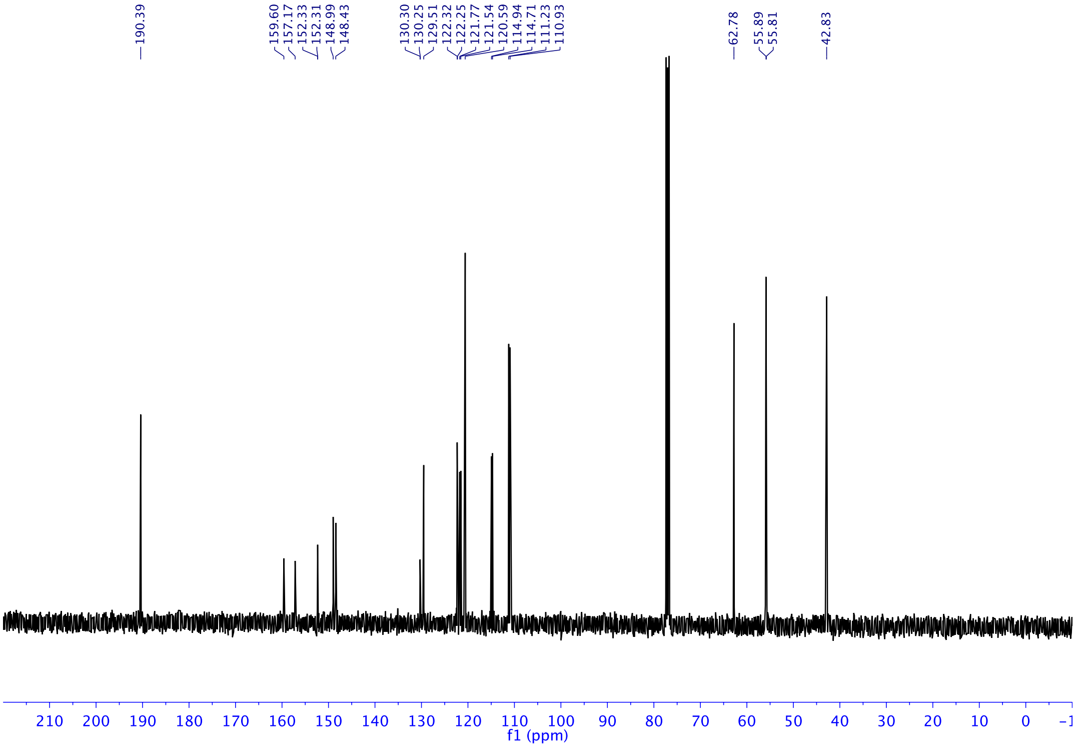

Figure 2. ^{1}H-NMR and ^{13}C-NMR spectra of compound **1j**

^{1}H NMR, 400 MHz, $CDCl_3$

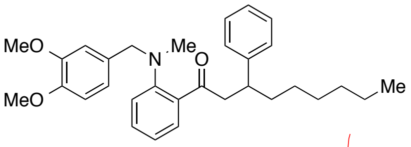

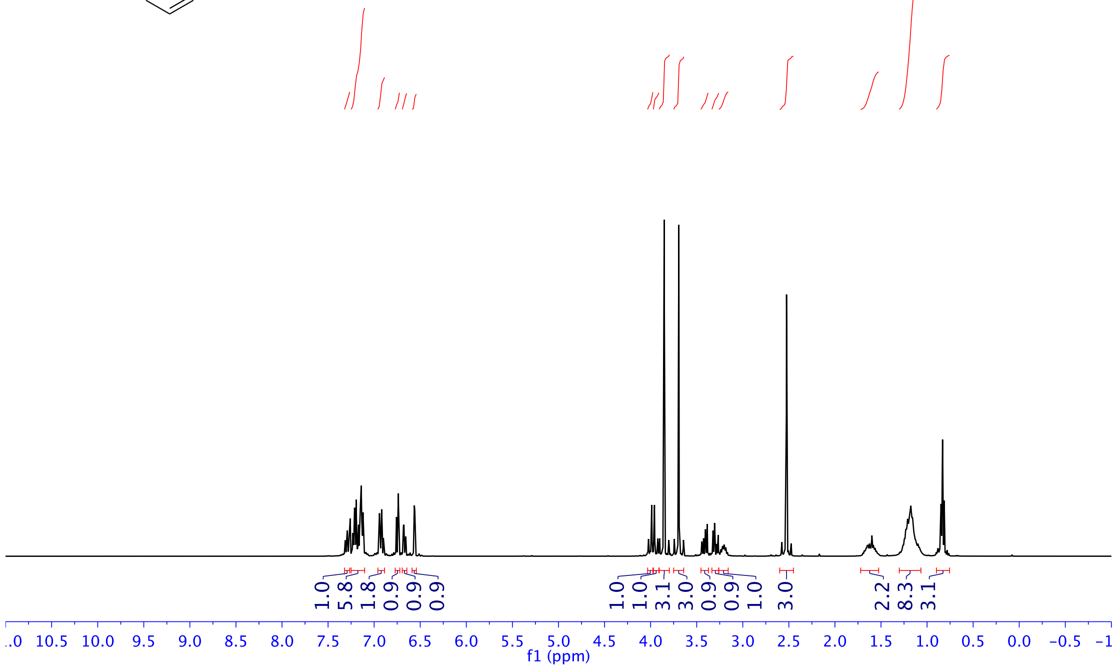

^{13}C NMR, 100 MHz, $CDCl_3$

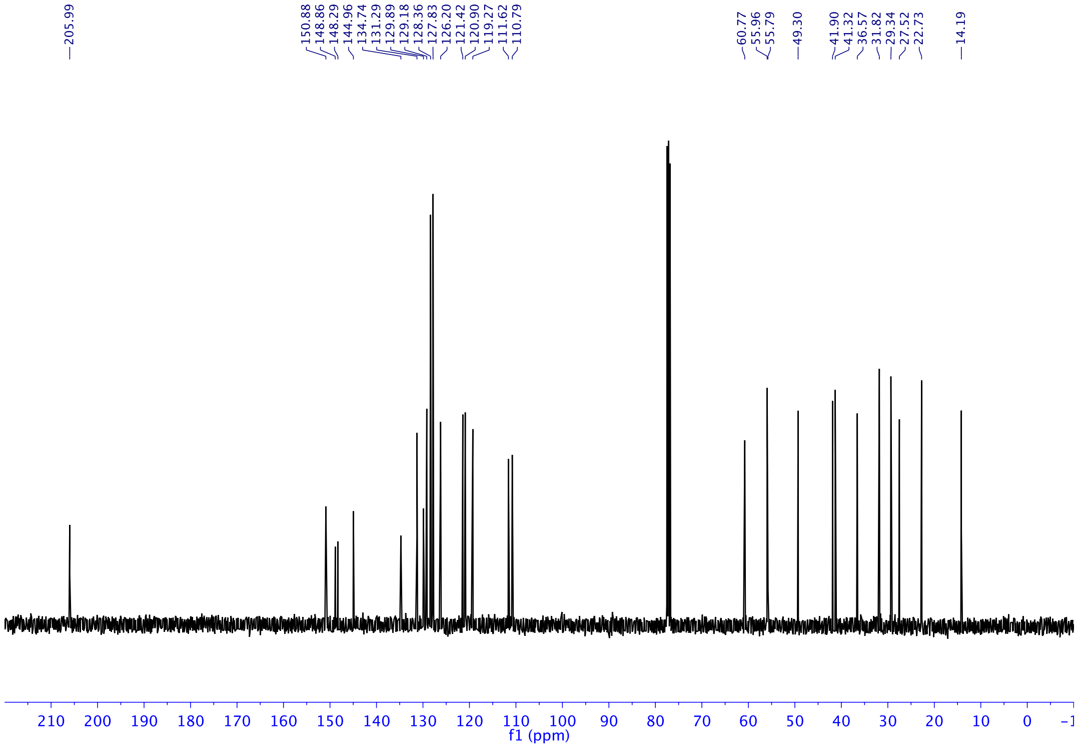

Figure 3. ^{1}H-NMR and ^{13}C-NMR spectra of compound **2a**

^{1}H NMR, 400 MHz, $CDCl_3$

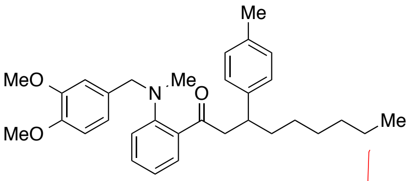

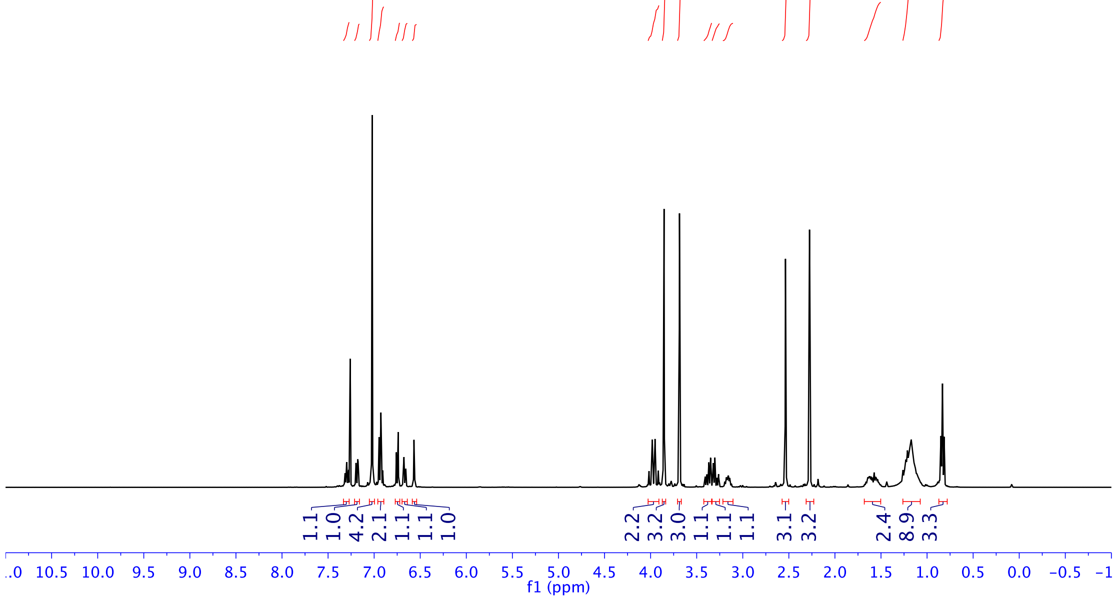

^{13}C NMR, 100 MHz, $CDCl_3$

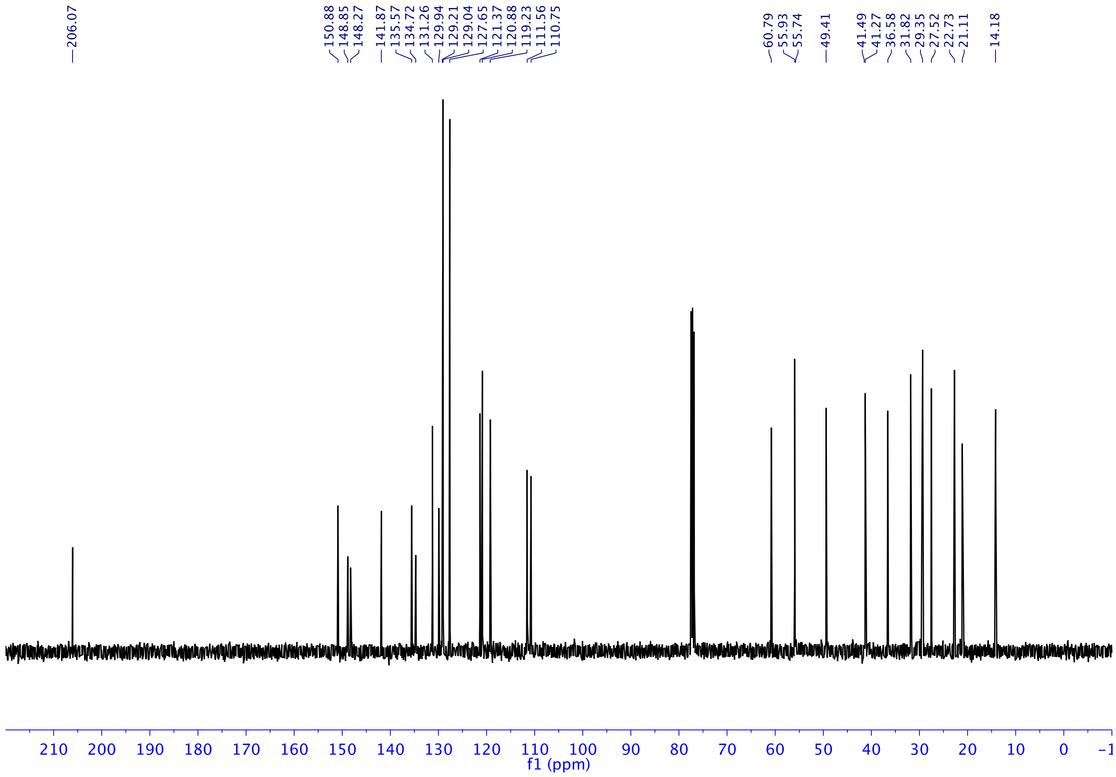

Figure 4. ^{1}H-NMR and ^{13}C-NMR spectra of compound **2b**

^{1}H NMR, 400 MHz, $CDCl_3$

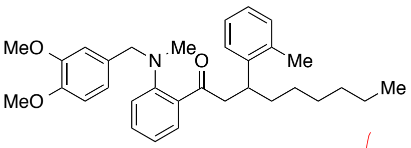

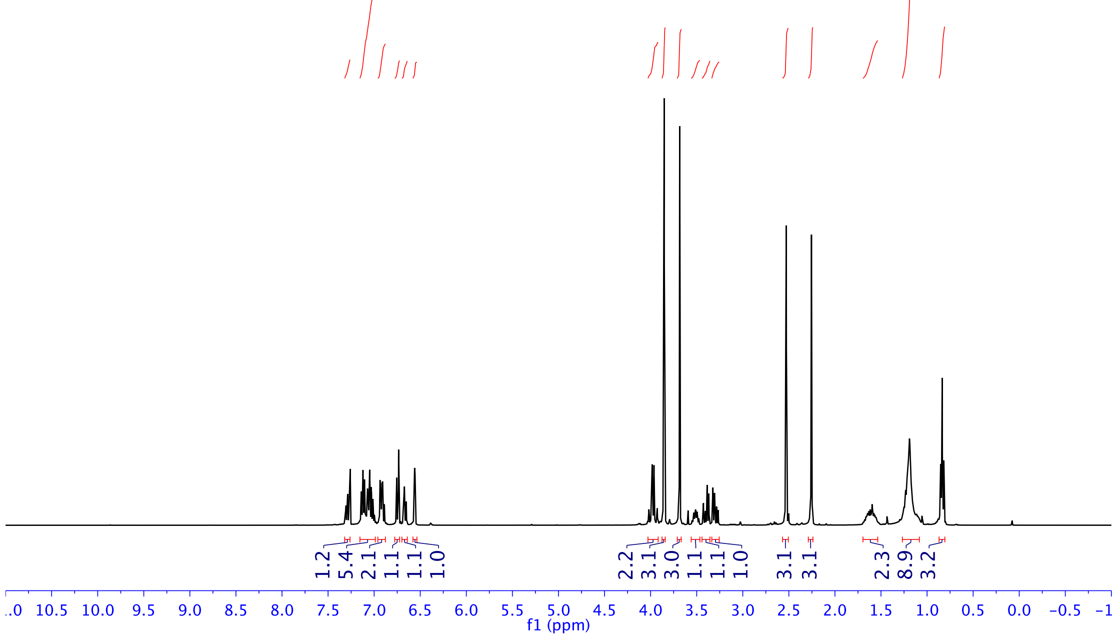

^{13}C NMR, 100 MHz, $CDCl_3$

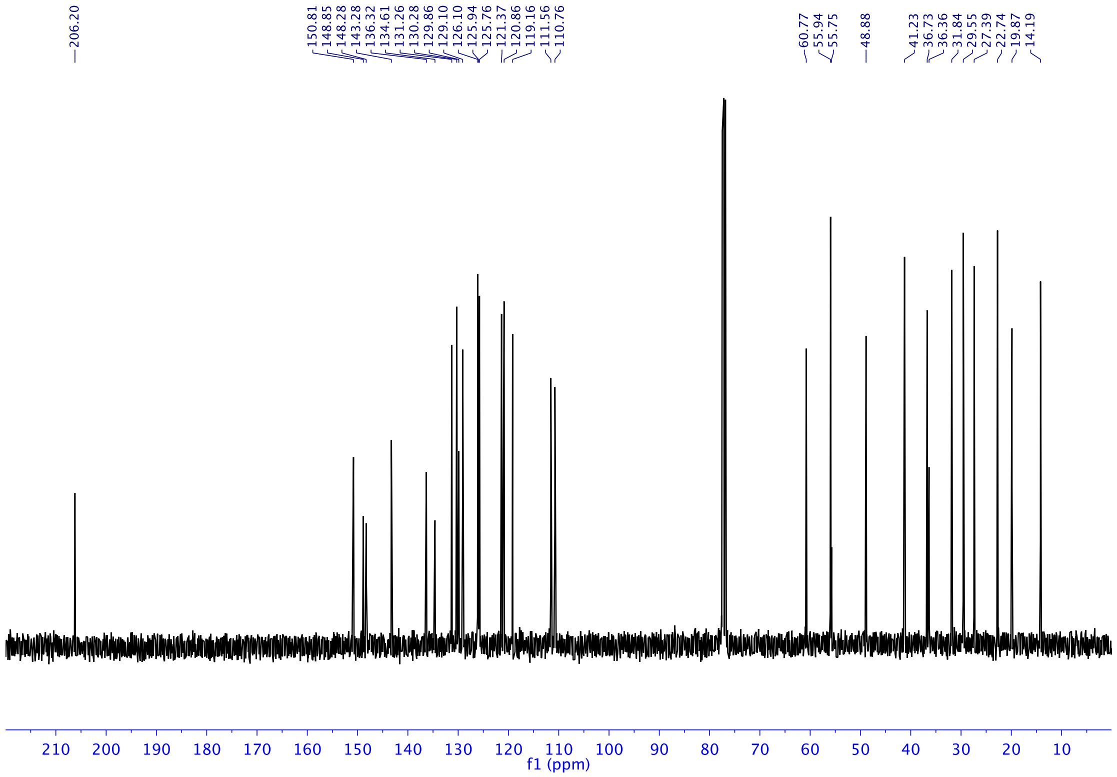

Figure 5. ^{1}H-NMR and ^{13}C-NMR spectra of compound **2c**

^{1}H NMR, 400 MHz, $CDCl_3$

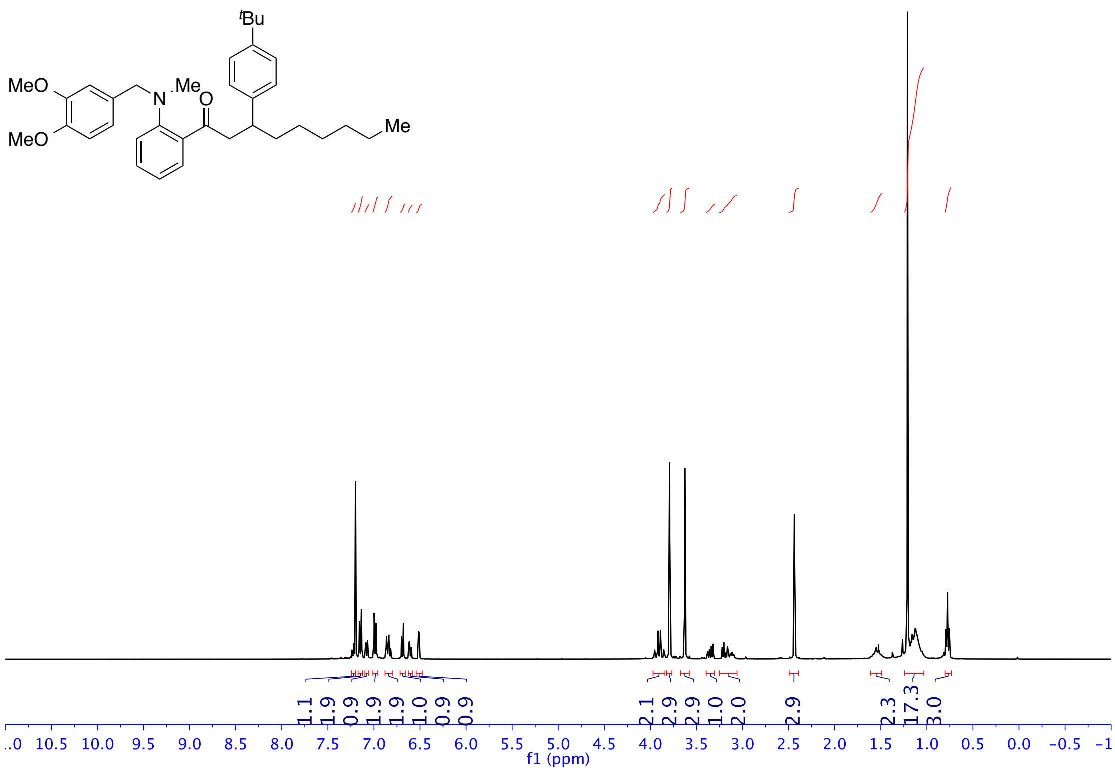

^{13}C NMR, 100 MHz, $CDCl_3$

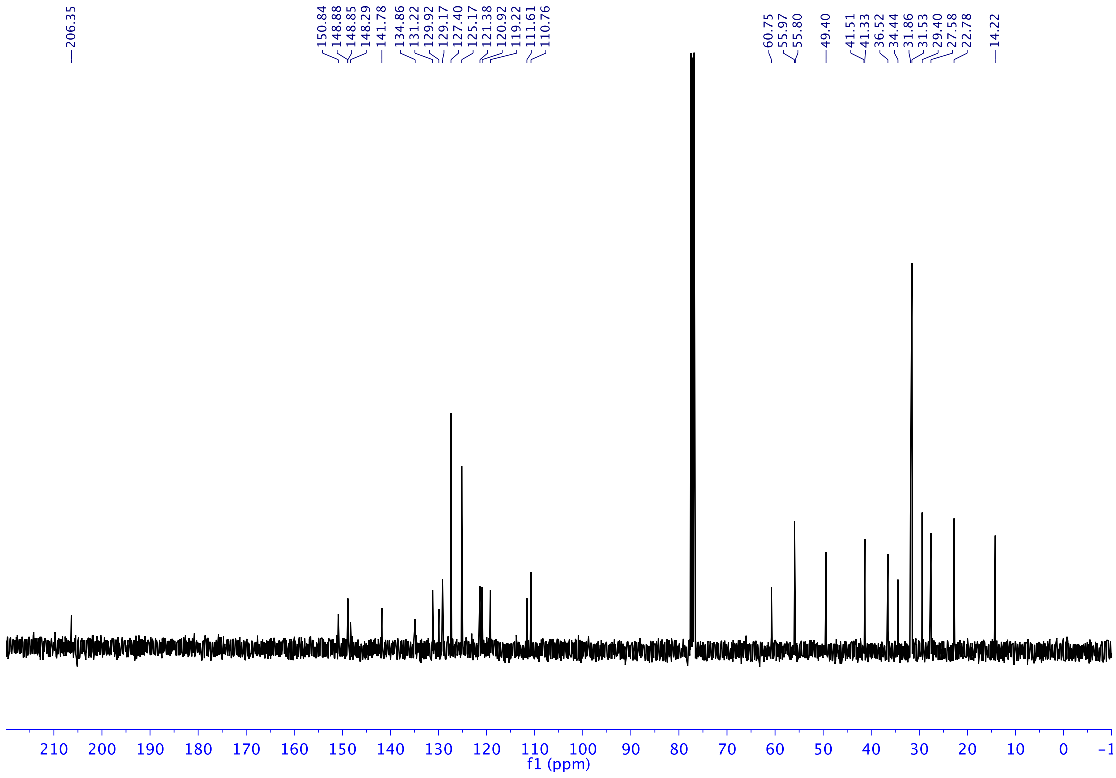

Figure 6. ^{1}H-NMR and ^{13}C-NMR spectra of compound **2d**

^{1}H NMR, 400 MHz, $CDCl_3$

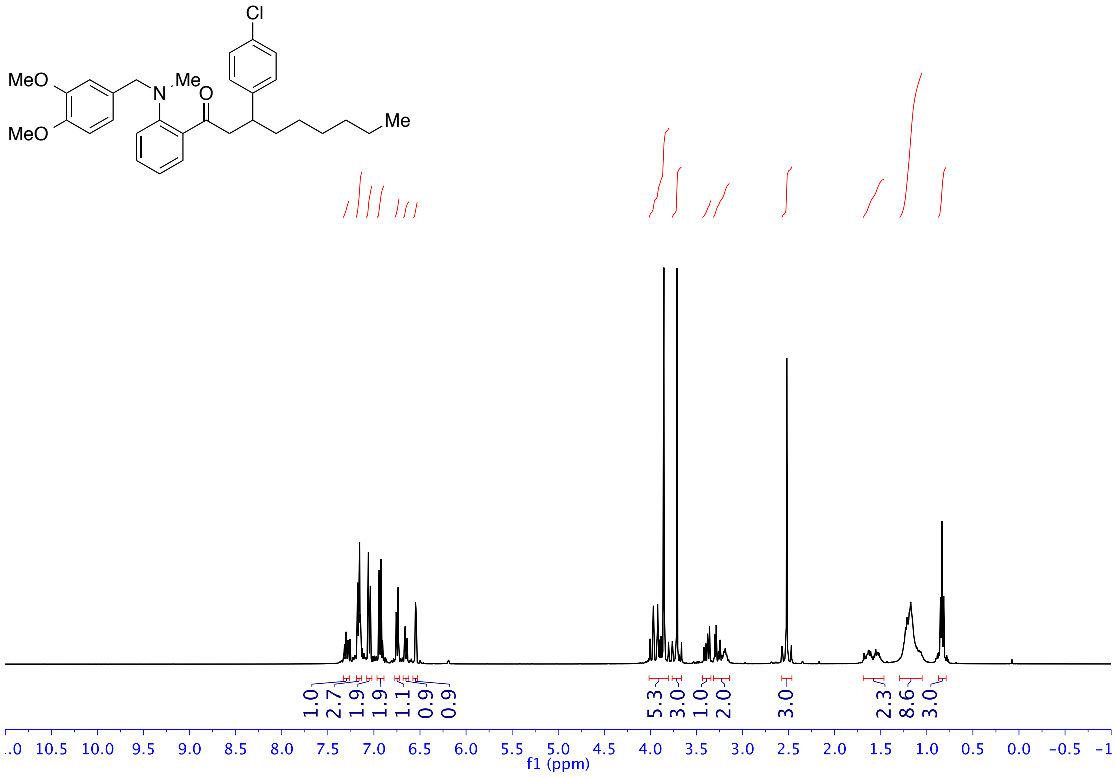

^{13}C NMR, 100 MHz, $CDCl_3$

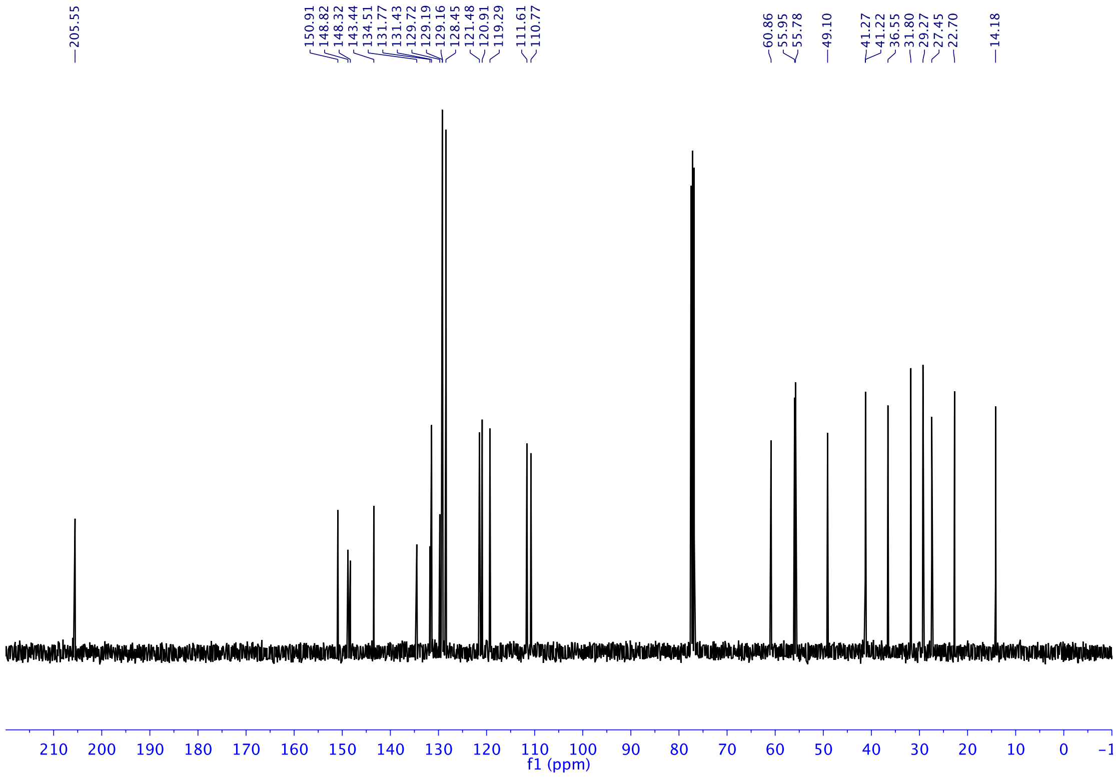

Figure 7. ^{1}H-NMR and ^{13}C-NMR spectra of compound **2e**

^{1}H NMR, 400 MHz, $CDCl_3$

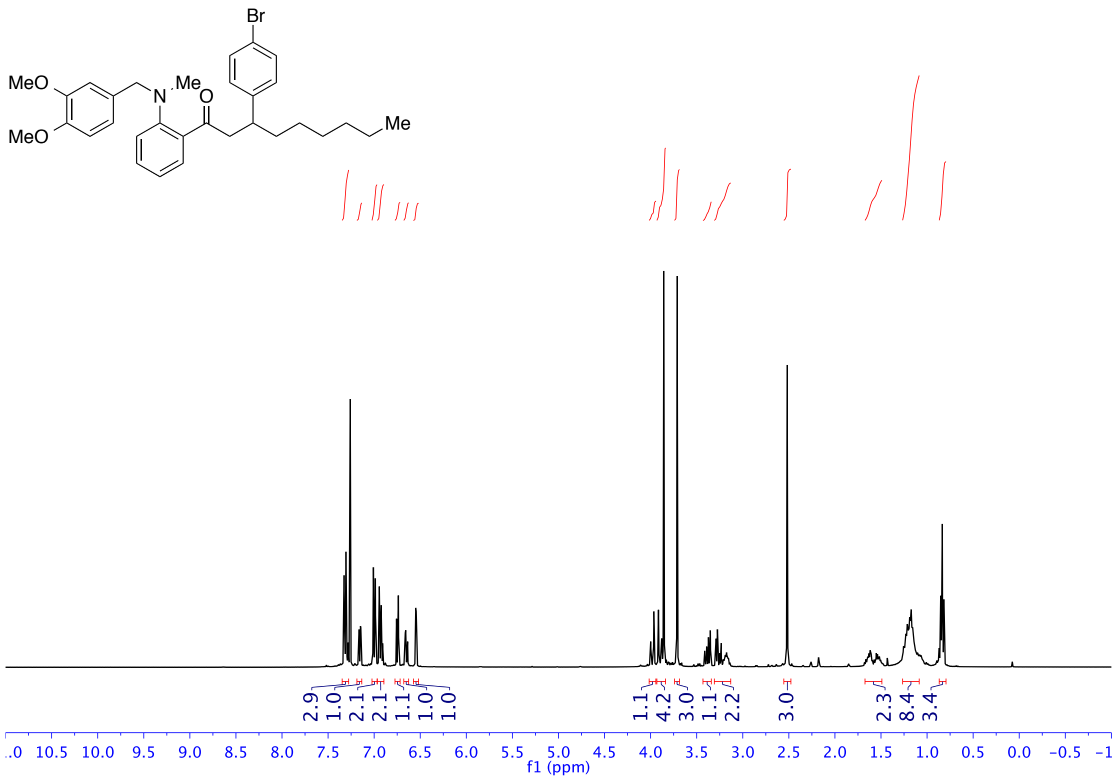

^{13}C NMR, 100 MHz, $CDCl_3$

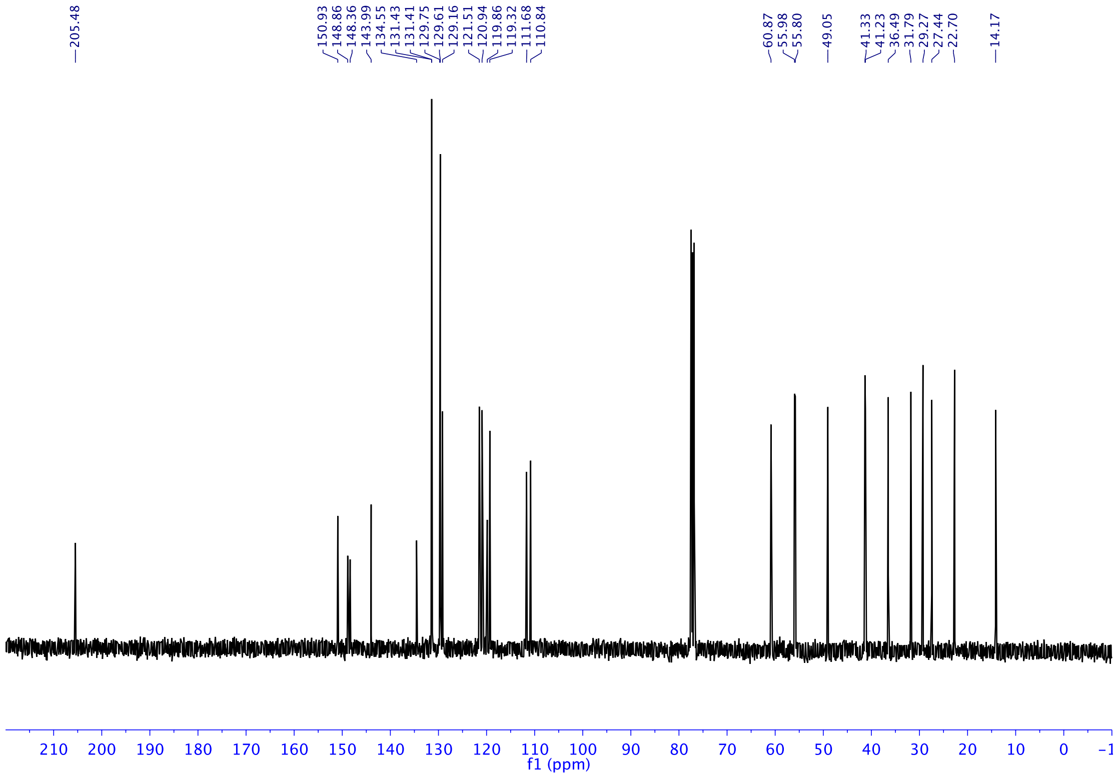

Figure 8. ^{1}H-NMR and ^{13}C-NMR spectra of compound **2f**

^{1}H NMR, 400 MHz, $CDCl_3$

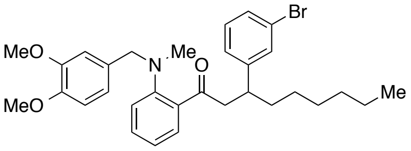

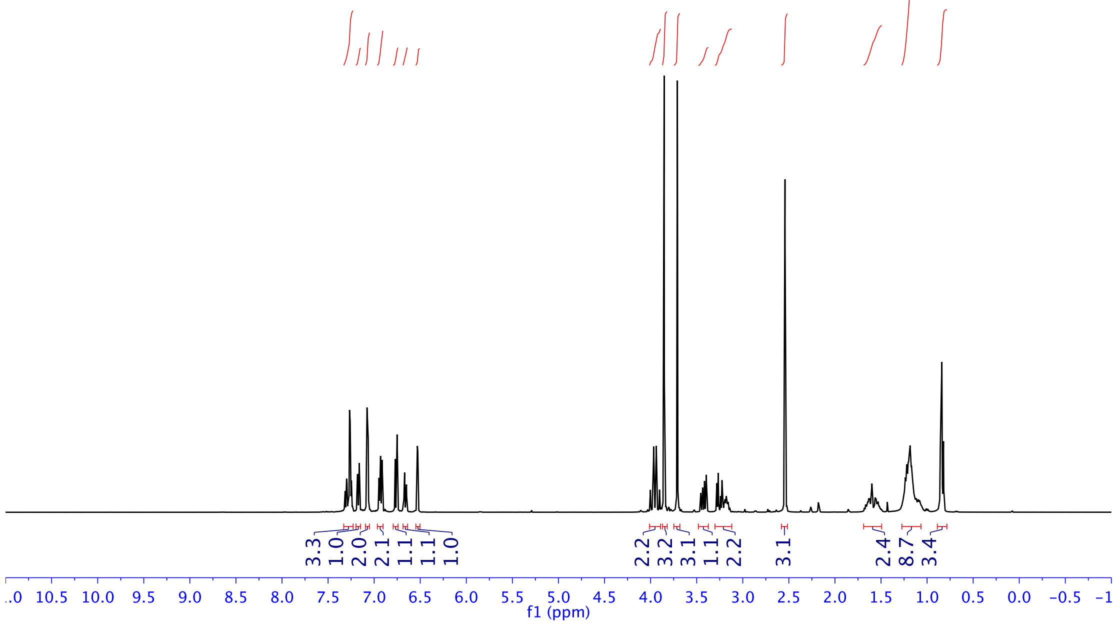

^{13}C NMR, 100 MHz, $CDCl_3$

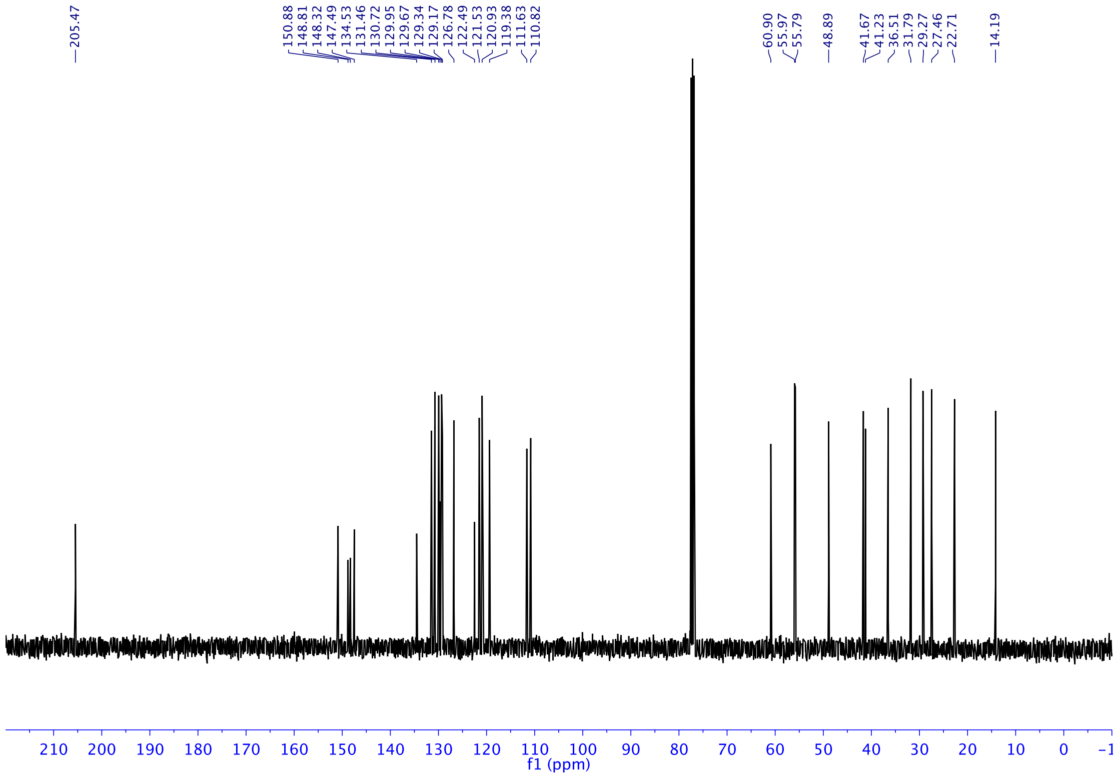

Figure 9. ^{1}H-NMR and ^{13}C-NMR spectra of compound **2g**

^{1}H NMR, 400 MHz, $CDCl_3$

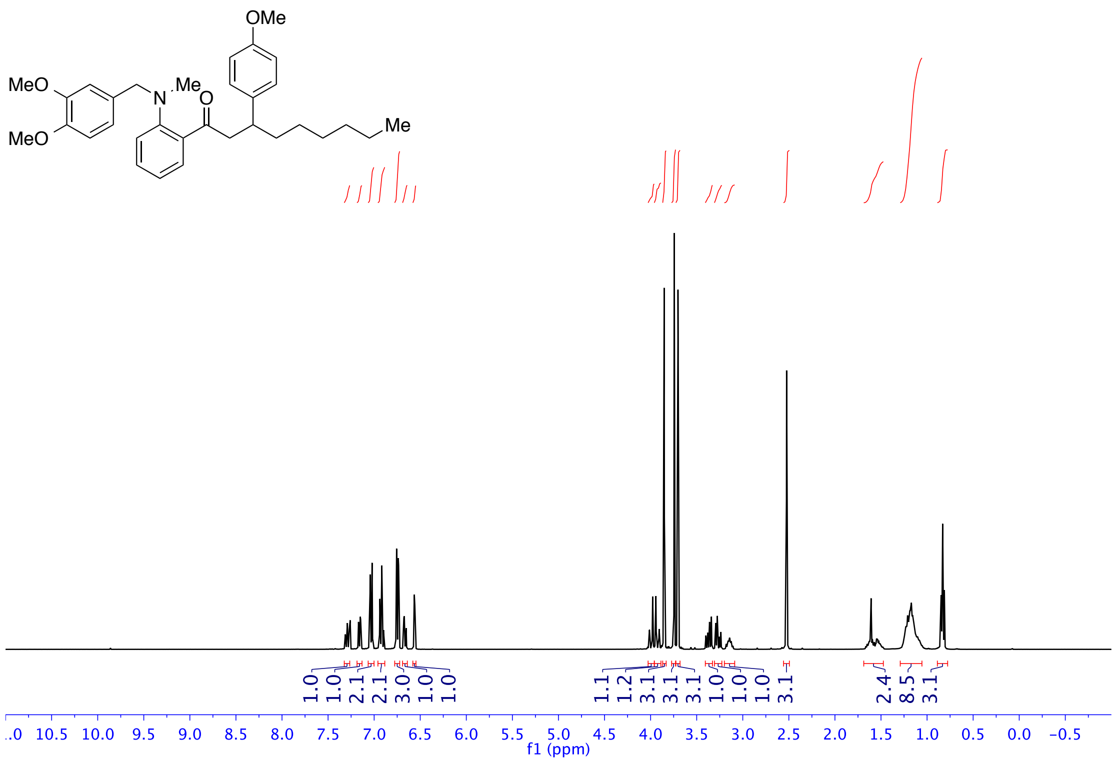

^{13}C NMR, 100 MHz, $CDCl_3$

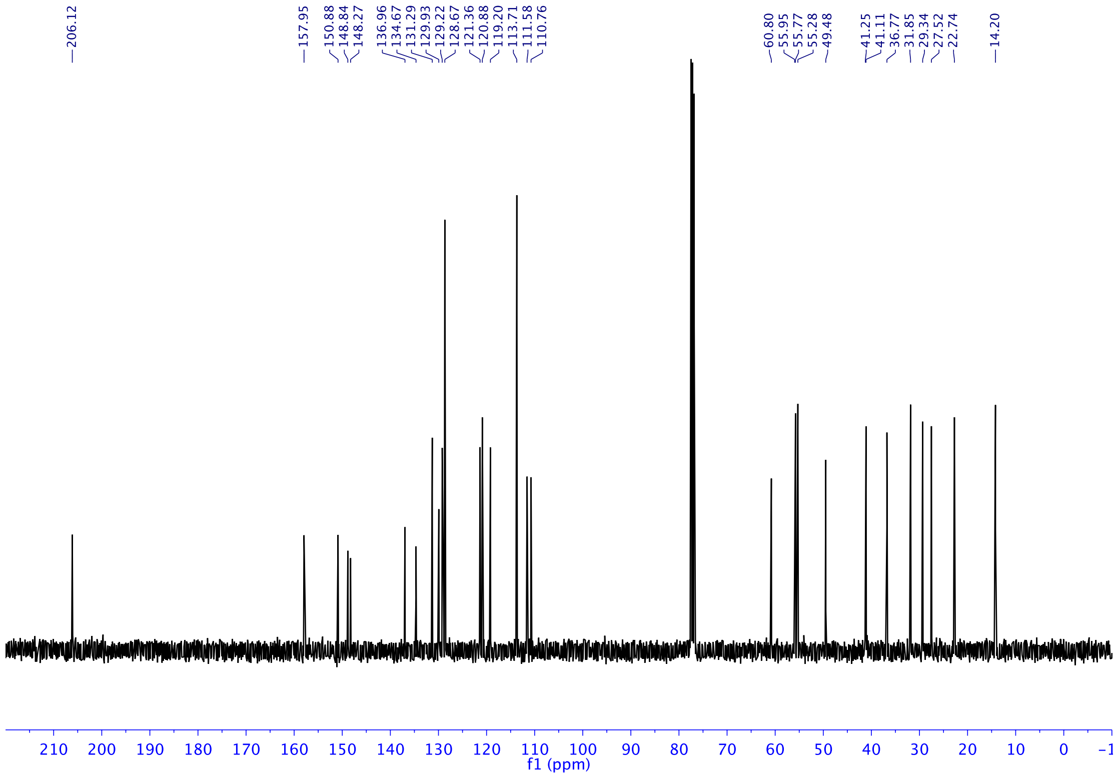

Figure 10. ^{1}H-NMR and ^{13}C-NMR spectra of compound **2h**

^{1}H NMR, 400 MHz, $CDCl_3$

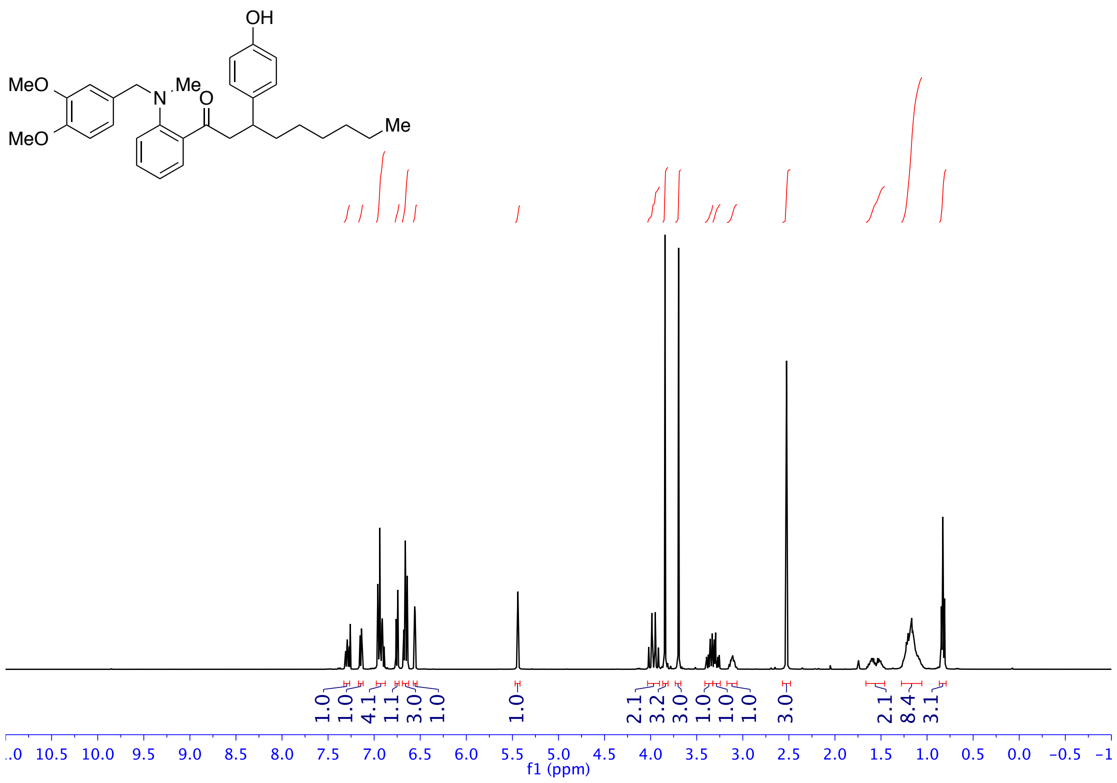

^{13}C NMR, 100 MHz, $CDCl_3$

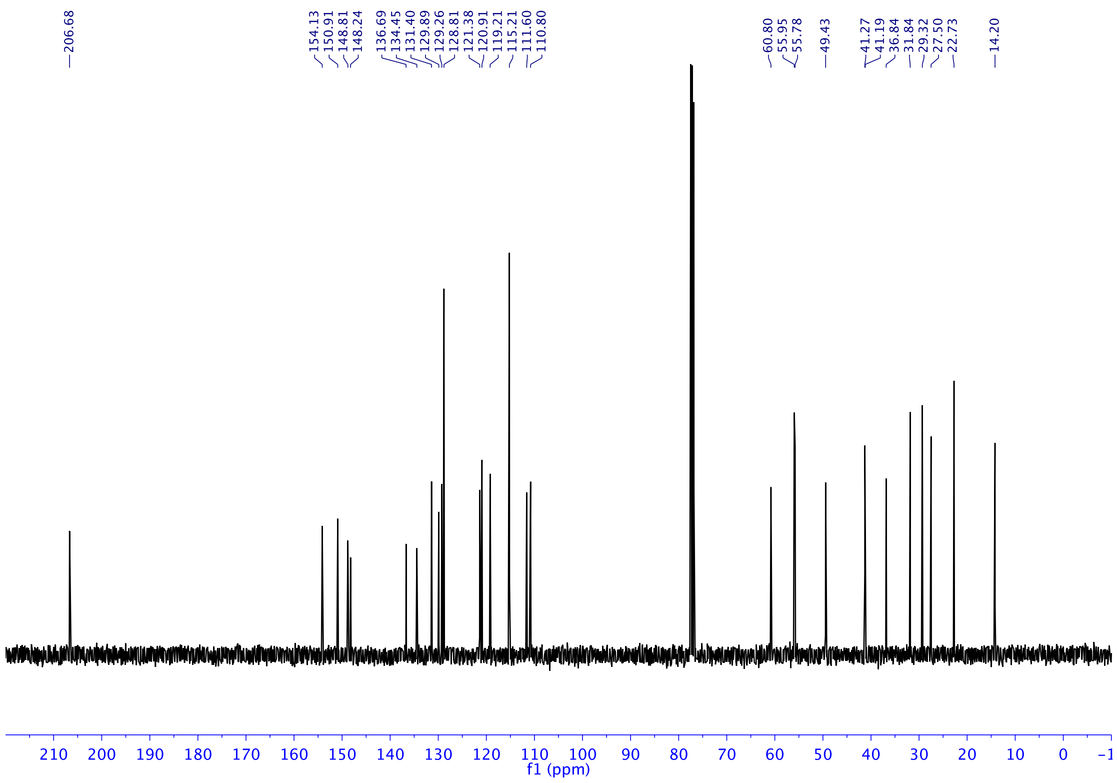

Figure 11. ^{1}H-NMR and ^{13}C-NMR spectra of compound **2i**

^{1}H NMR, 400 MHz, $CDCl_3$

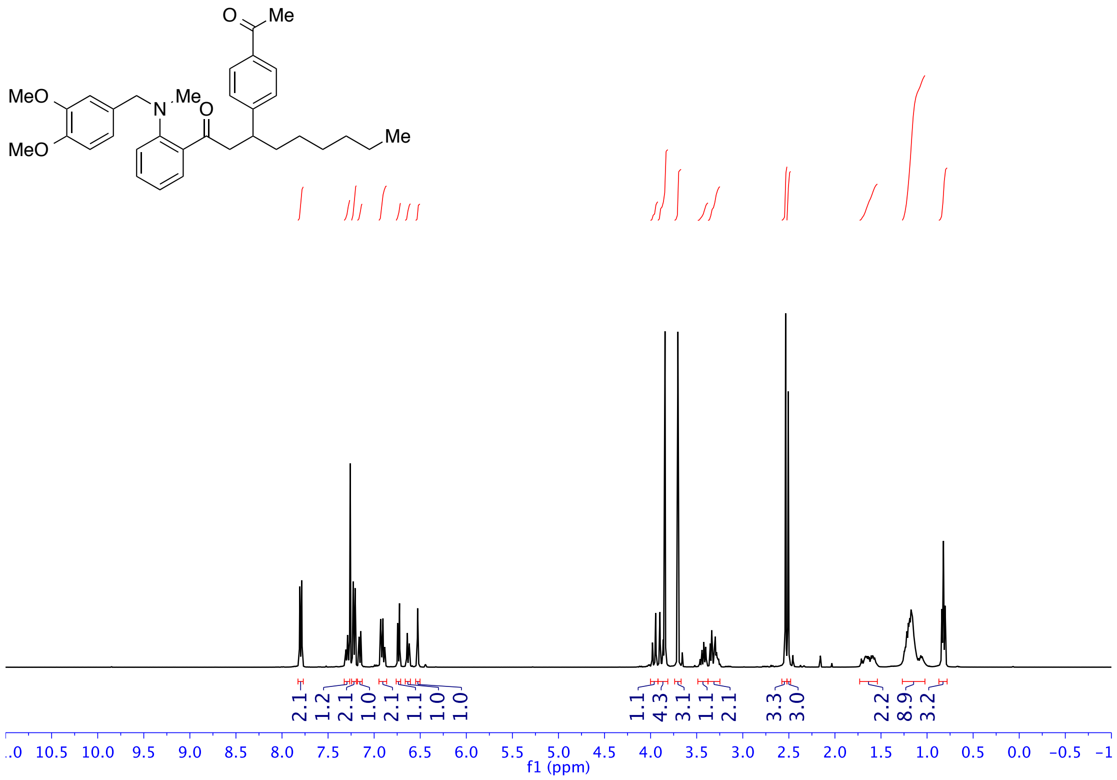

^{13}C NMR, 100 MHz, $CDCl_3$

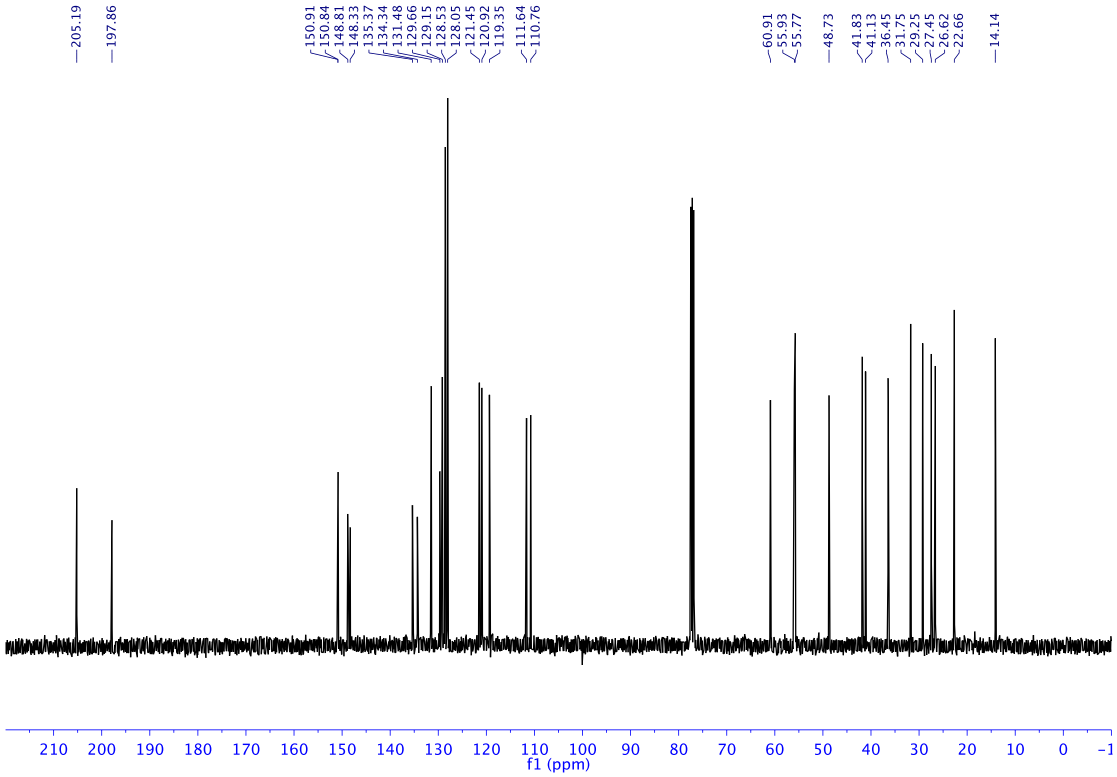

Figure 12. ^{1}H-NMR and ^{13}C-NMR spectra of compound **2j**

^{1}H NMR, 400 MHz, $CDCl_3$

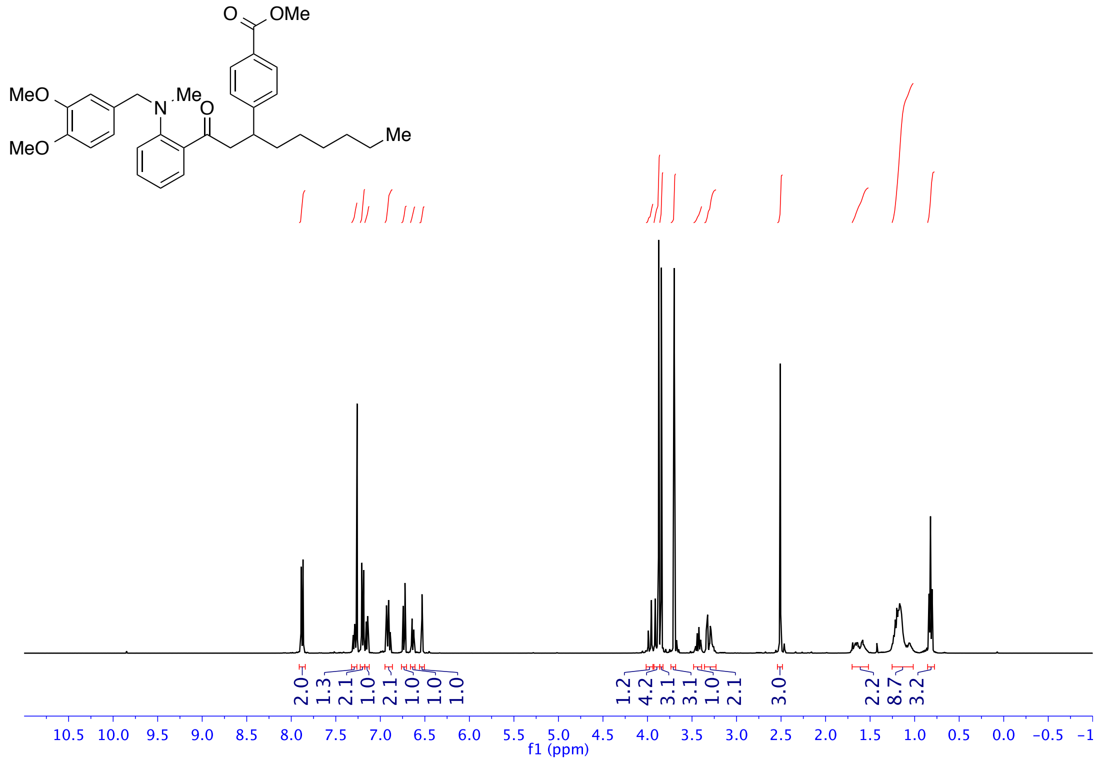

^{13}C NMR, 100 MHz, $CDCl_3$

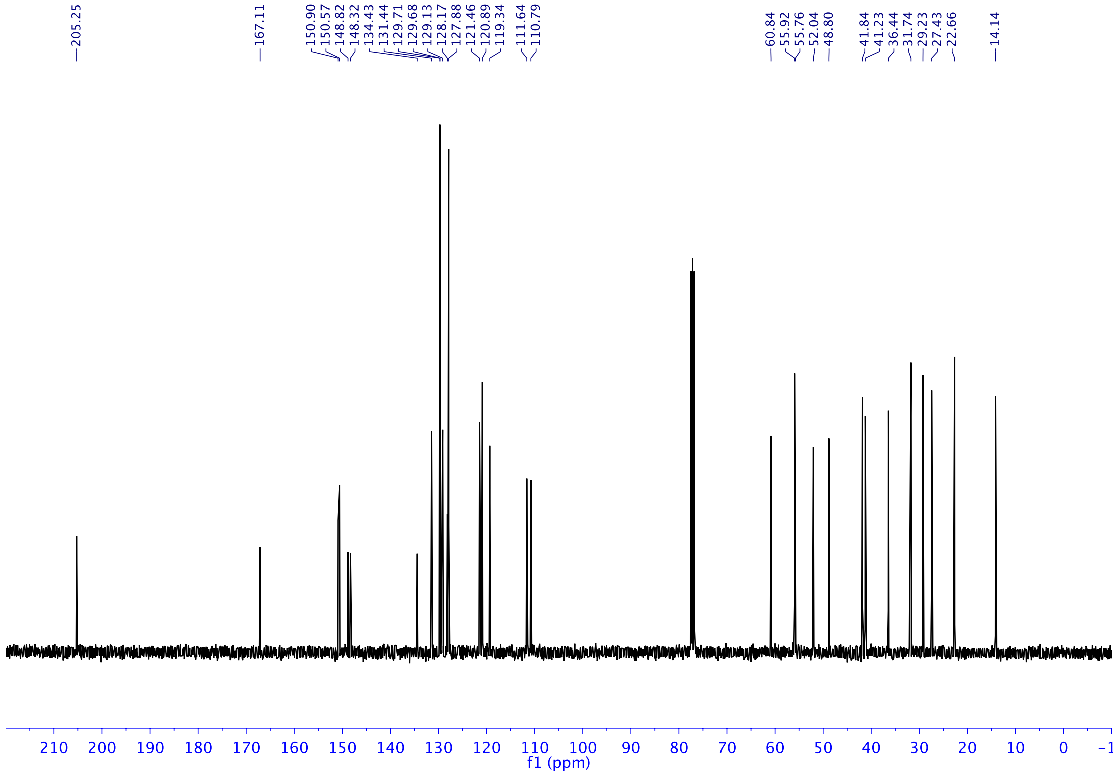

Figure 13. ^{1}H-NMR and ^{13}C-NMR spectra of compound **2k**

^{1}H NMR, 400 MHz, $CDCl_3$

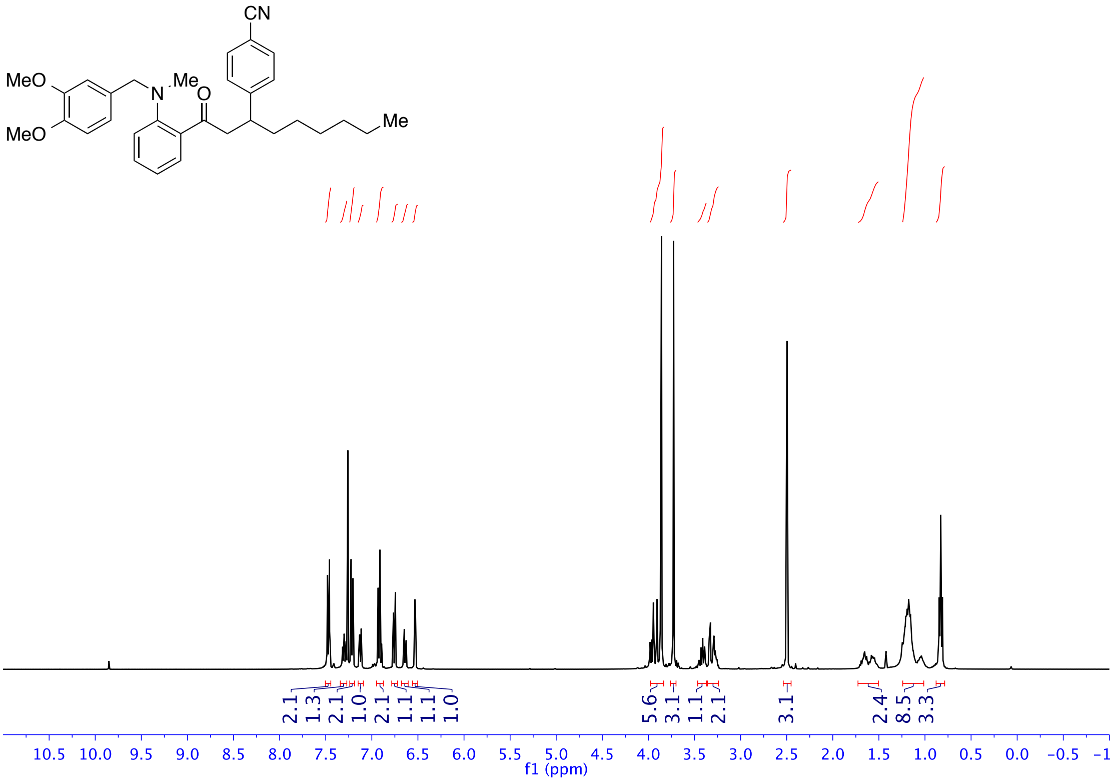

^{13}C NMR, 100 MHz, $CDCl_3$

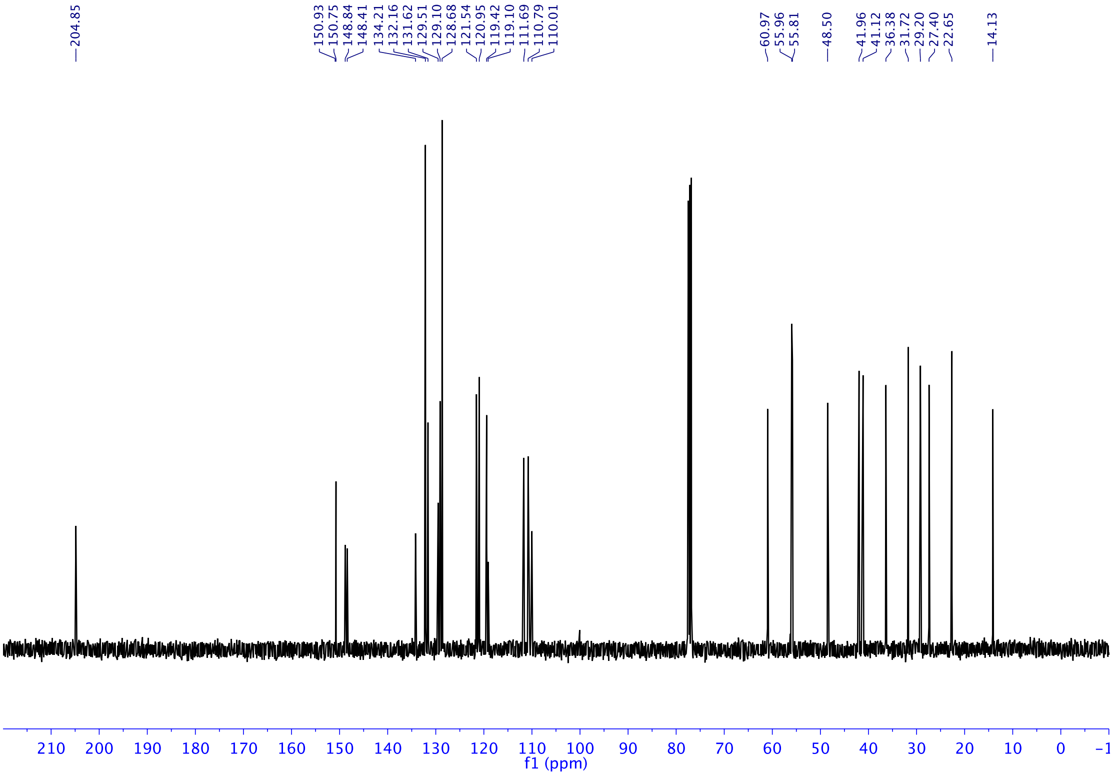

Figure 14. ^{1}H-NMR and ^{13}C-NMR spectra of compound **2l**

^{1}H NMR, 400 MHz, $CDCl_3$

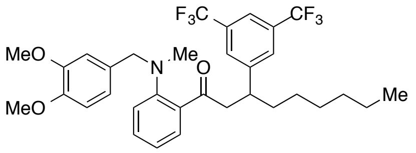

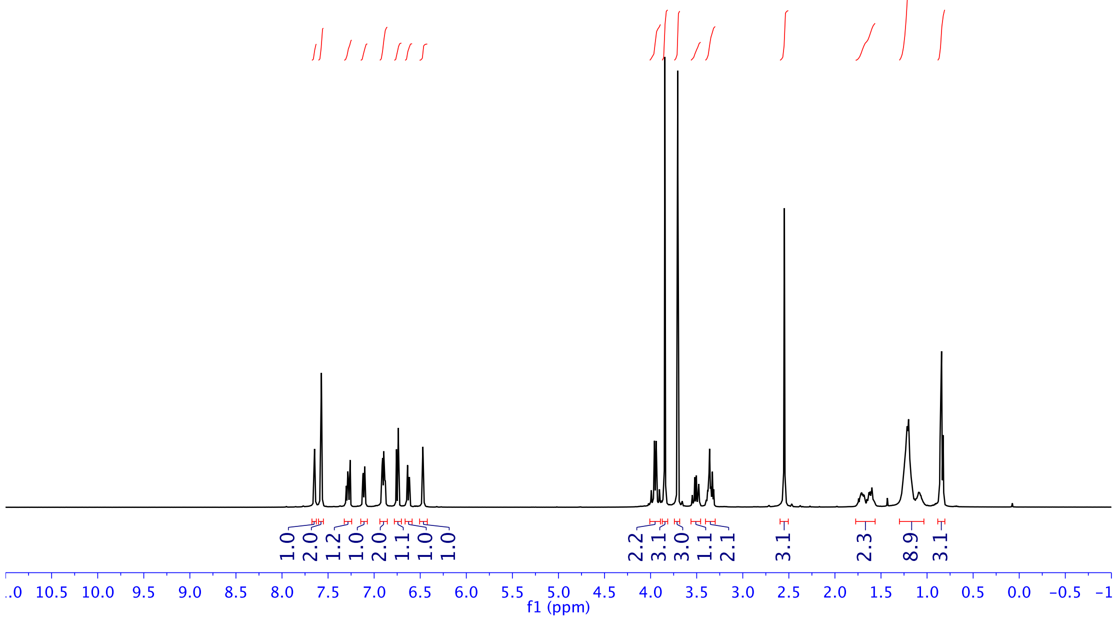

^{13}C NMR, 100 MHz, $CDCl_3$

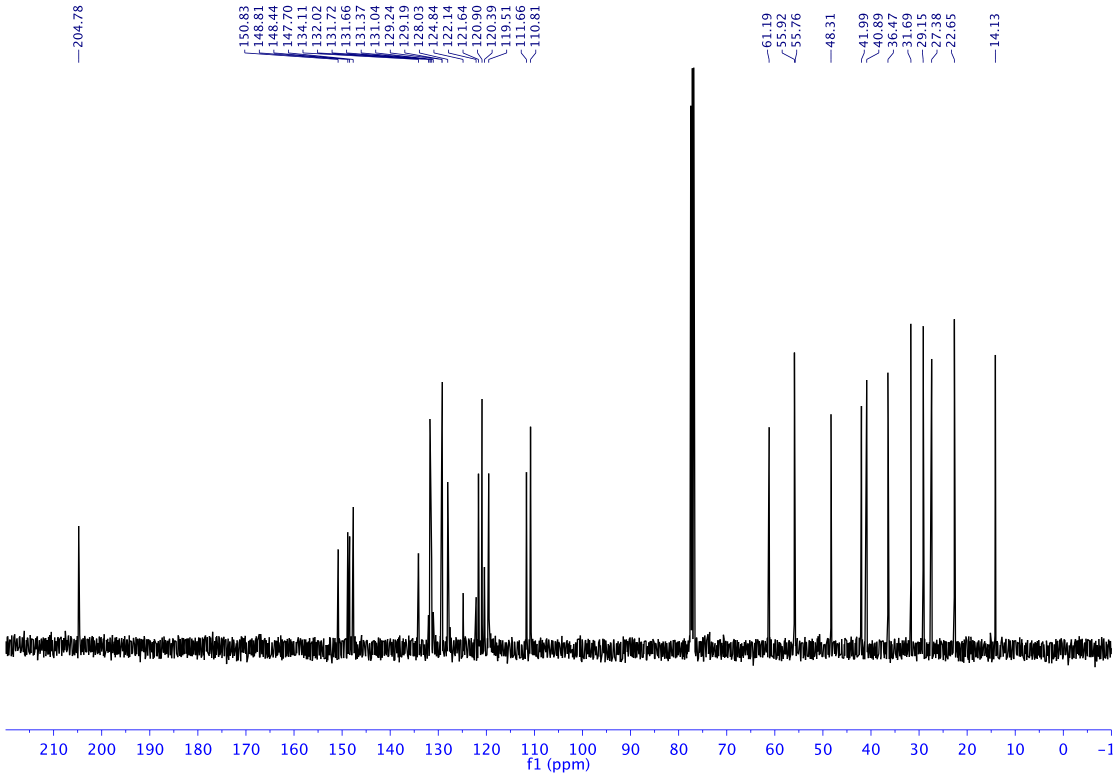

Figure 15. ^{1}H-NMR and ^{13}C-NMR spectra of compound **2m**

^{1}H NMR, 400 MHz, $CDCl_3$

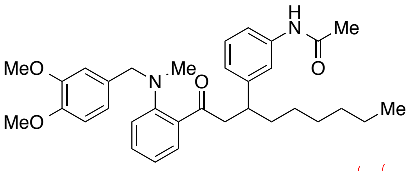

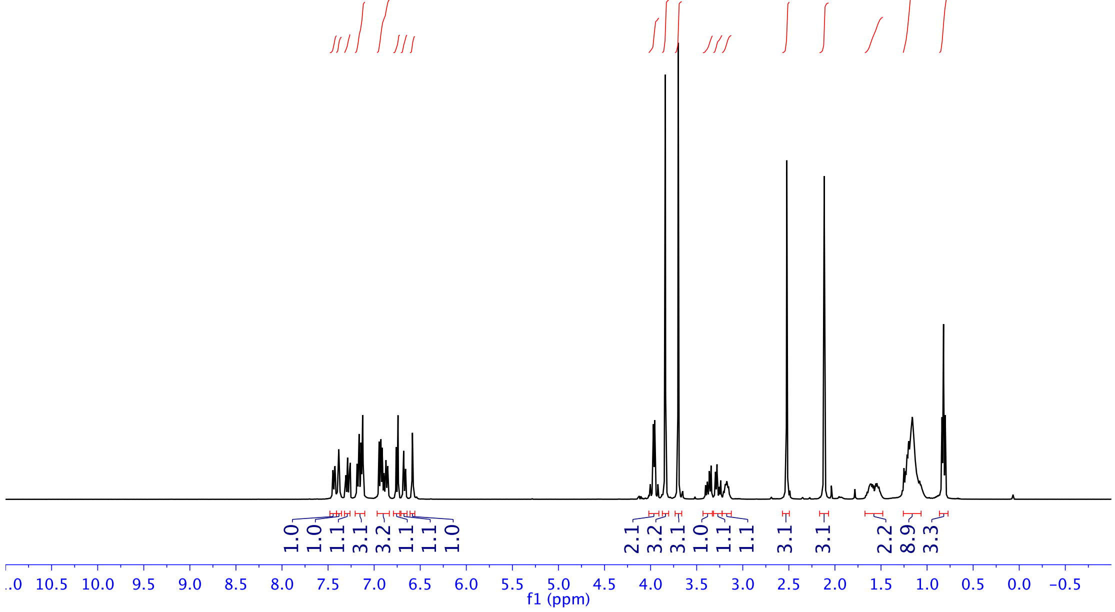

^{13}C NMR, 100 MHz, $CDCl_3$

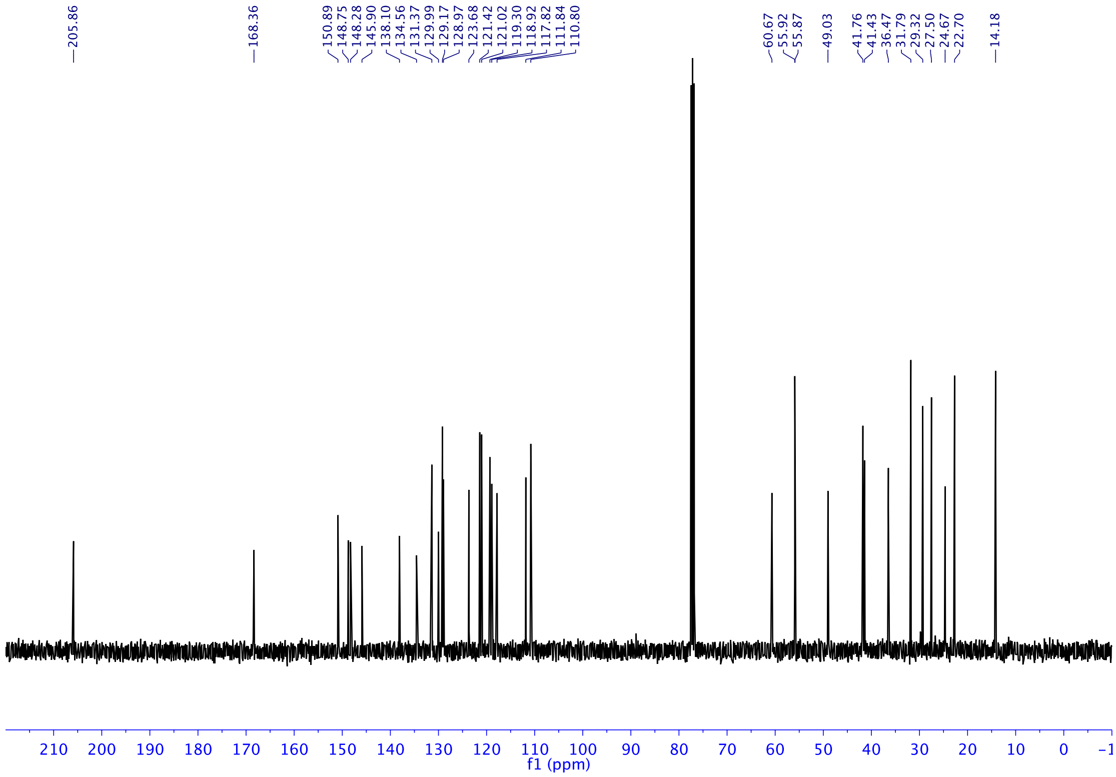

Figure 16. ^{1}H-NMR and ^{13}C-NMR spectra of compound **2n**

^{1}H NMR, 400 MHz, $CDCl_3$

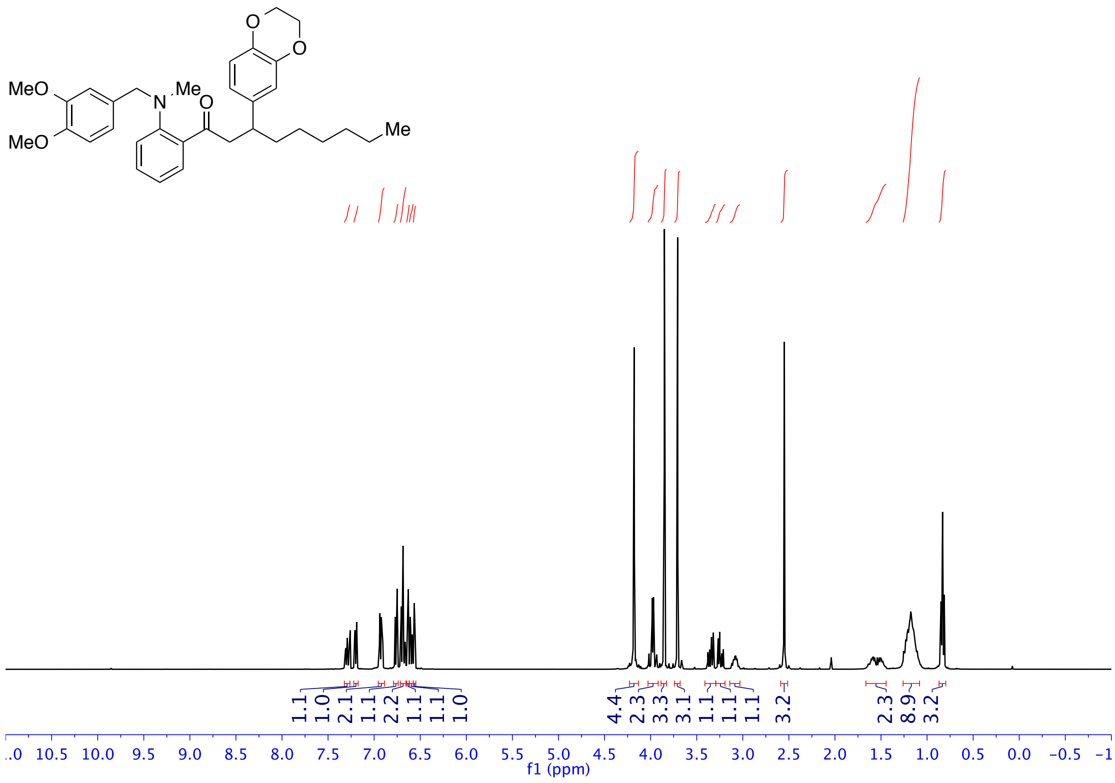

^{13}C NMR, 100 MHz, $CDCl_3$

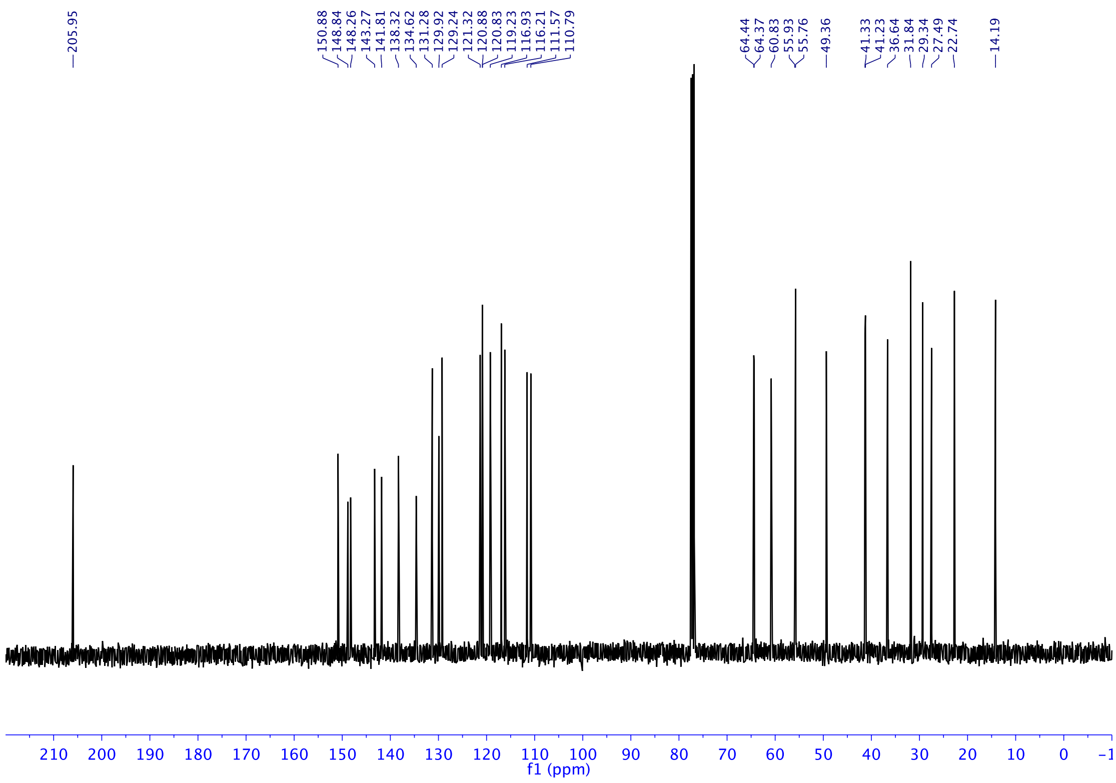

Figure 17. ^{1}H-NMR and ^{13}C-NMR spectra of compound **2o**

^{1}H NMR, 400 MHz, $CDCl_3$

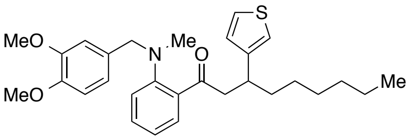

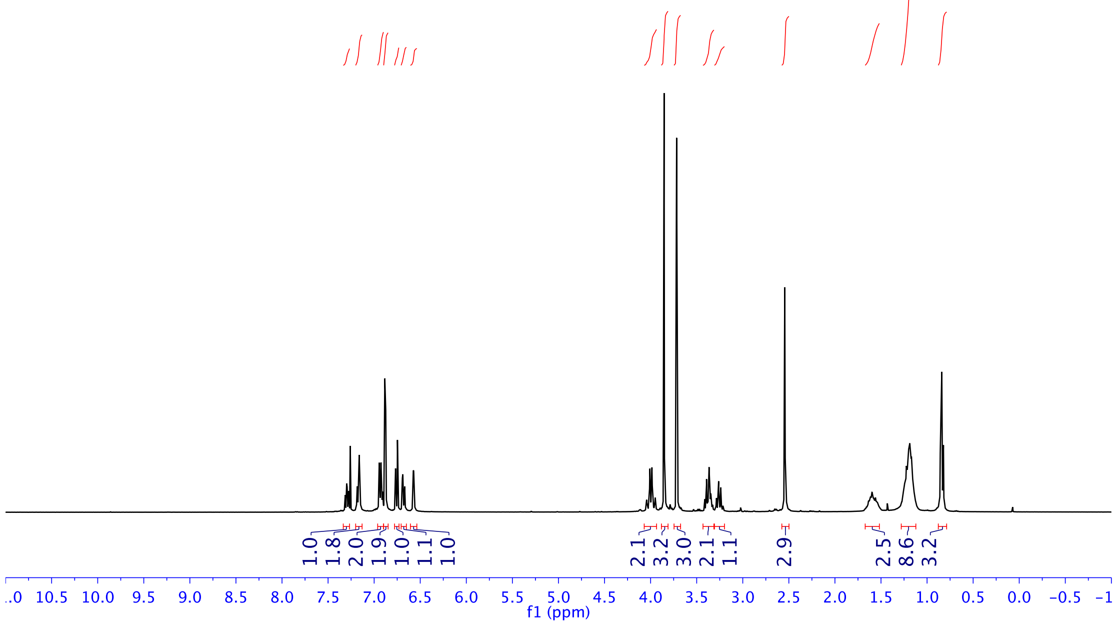

^{13}C NMR, 100 MHz, $CDCl_3$

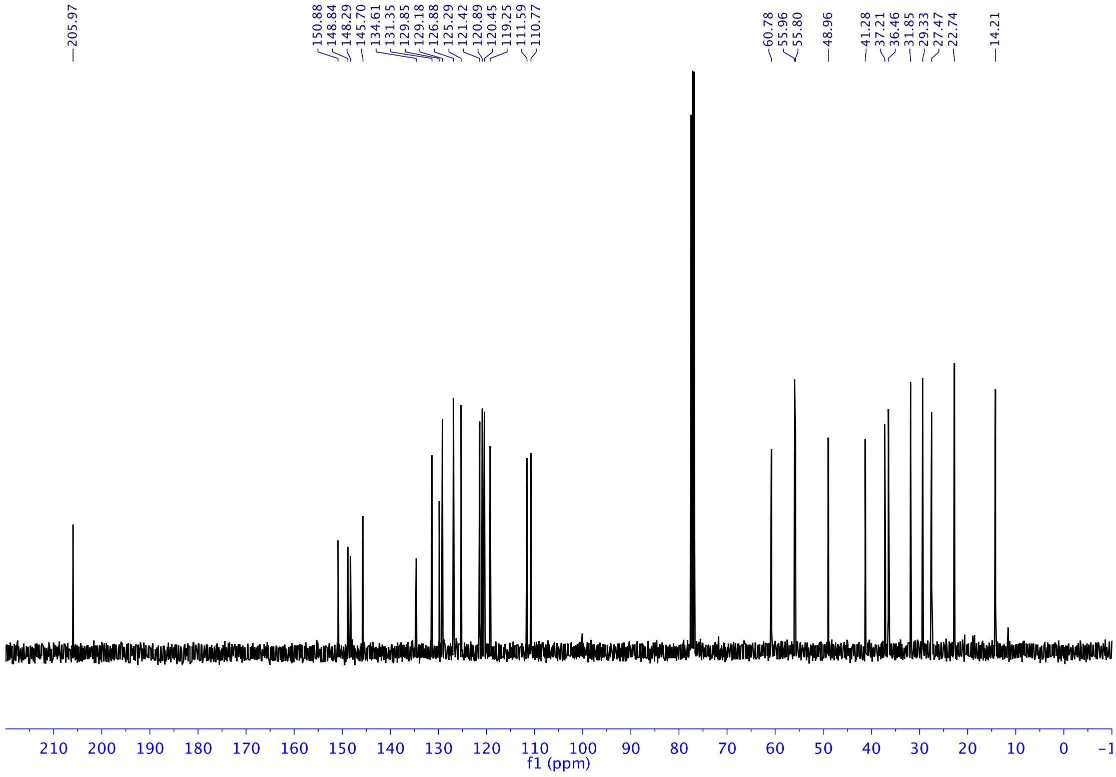

Figure 18. ^{1}H-NMR and ^{13}C-NMR spectra of compound **2p**

^{1}H NMR, 400 MHz, $CDCl_3$

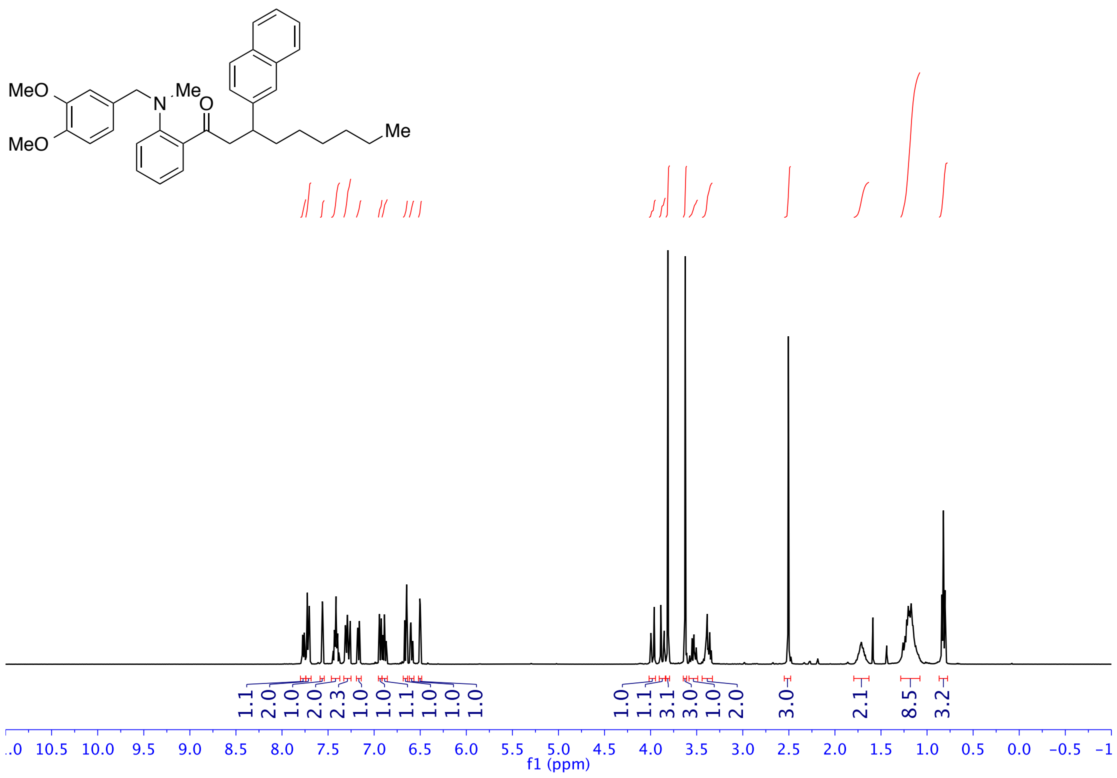

^{13}C NMR, 100 MHz, $CDCl_3$

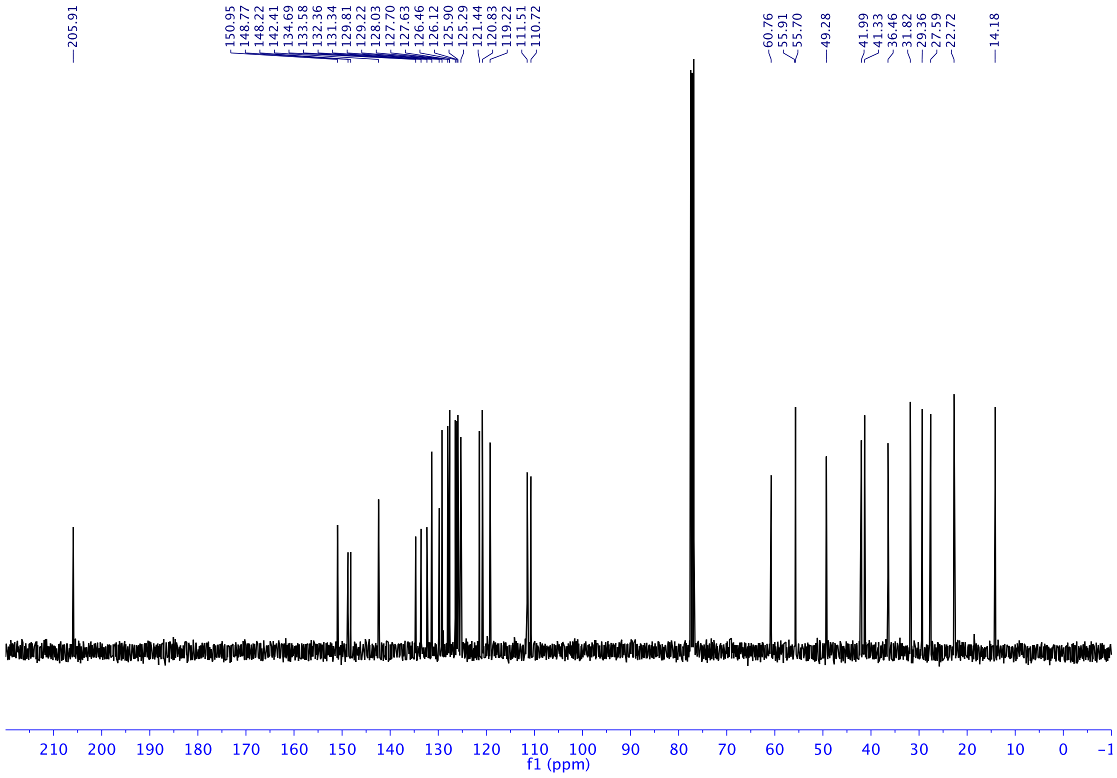

Figure 19. ^{1}H-NMR and ^{13}C-NMR spectra of compound **2q**

^{1}H NMR, 400 MHz, $CDCl_3$

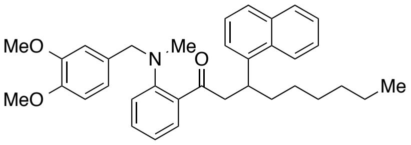

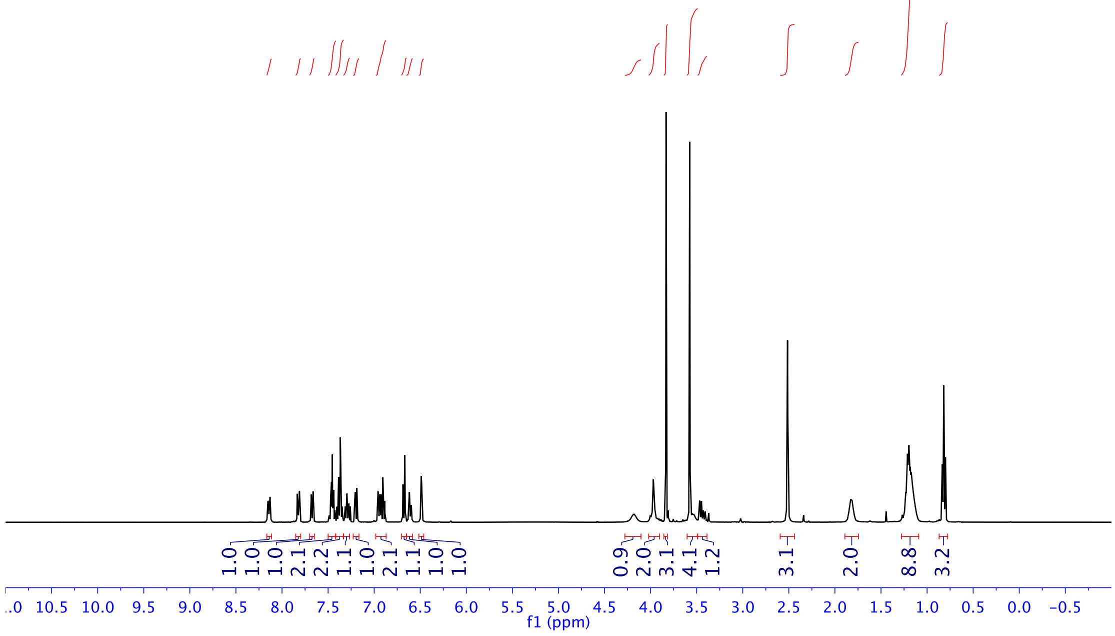

^{13}C NMR, 100 MHz, $CDCl_3$

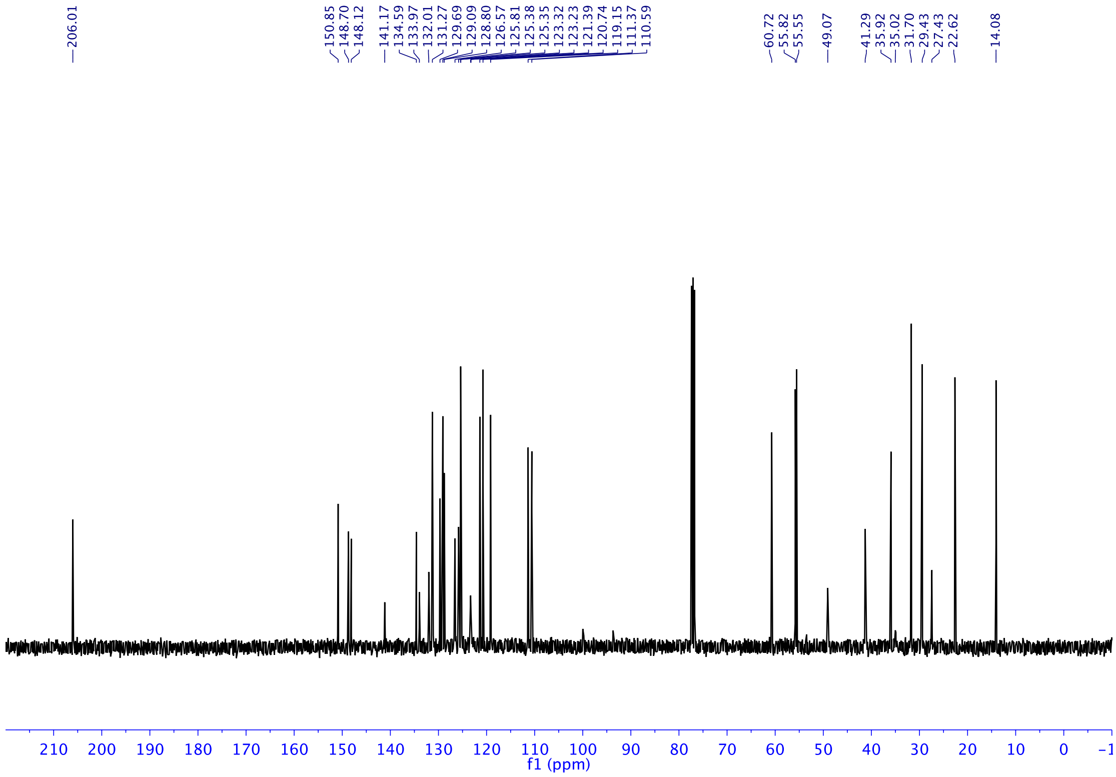

Figure 20. ^{1}H-NMR and ^{13}C-NMR spectra of compound **2r**

^{1}H NMR, 400 MHz, $CDCl_3$

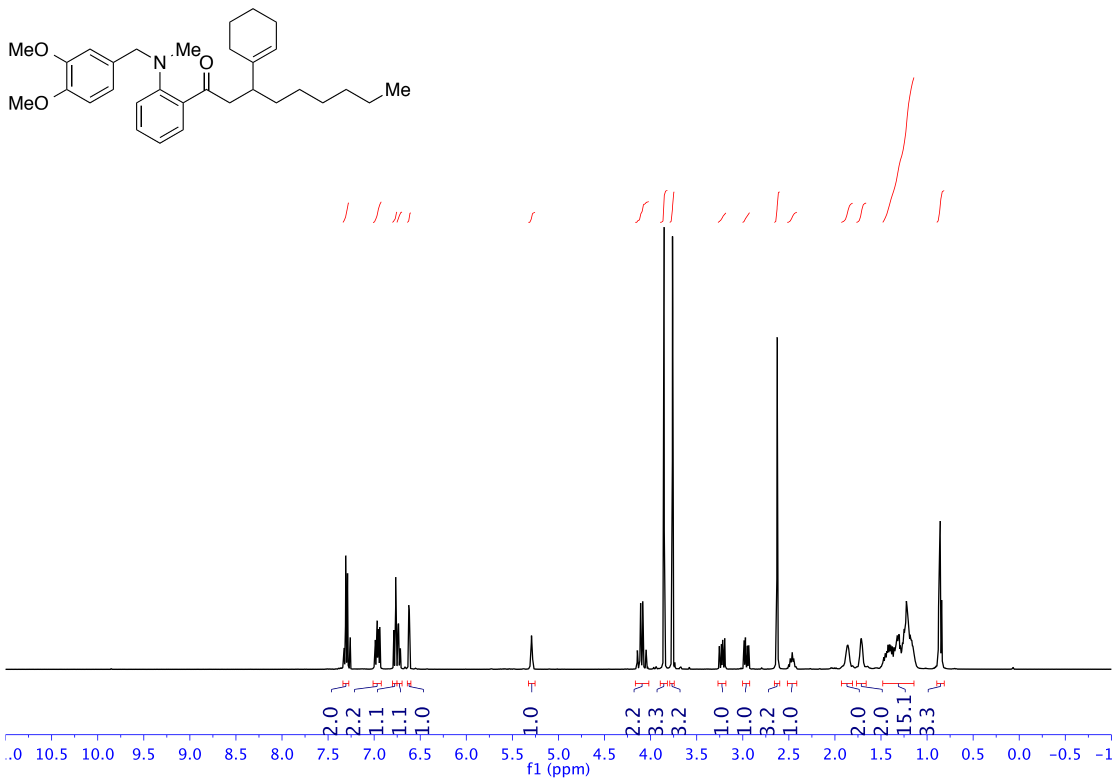

^{13}C NMR, 100 MHz, $CDCl_3$

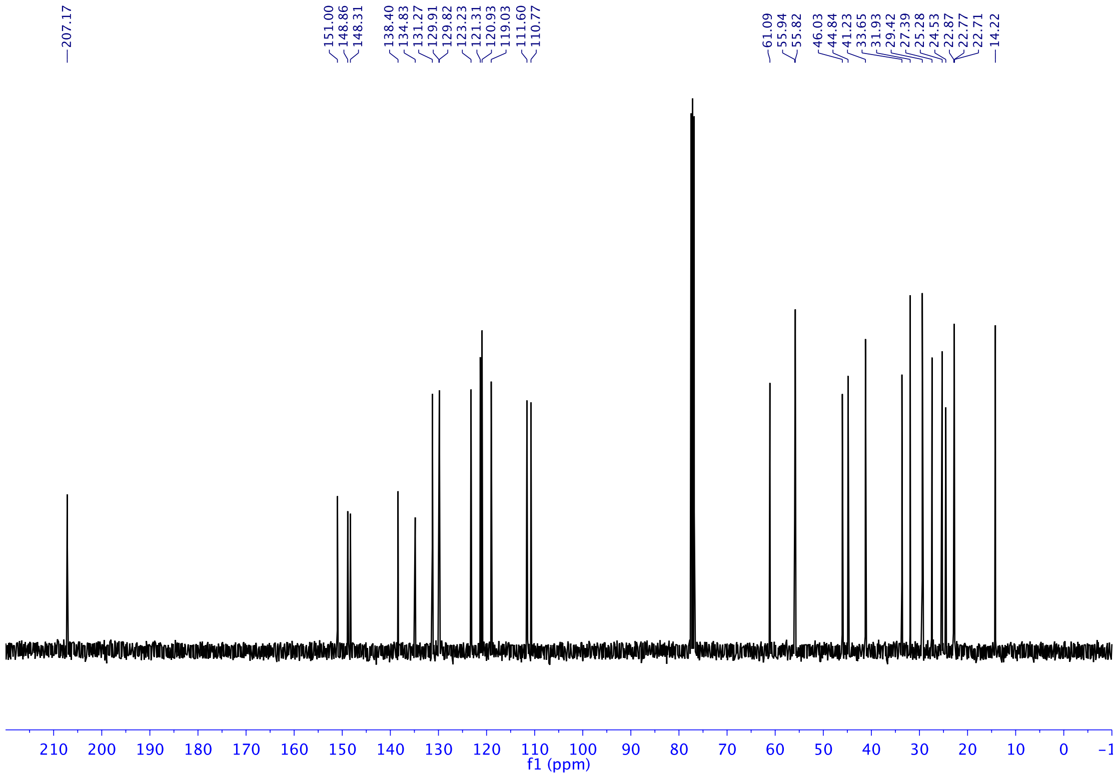

Figure 21. ^{1}H-NMR and ^{13}C-NMR spectra of compound **2s**

^{1}H NMR, 400 MHz, $CDCl_3$

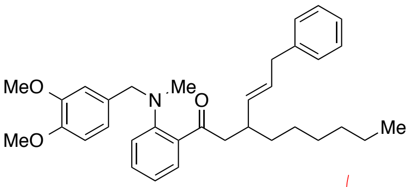

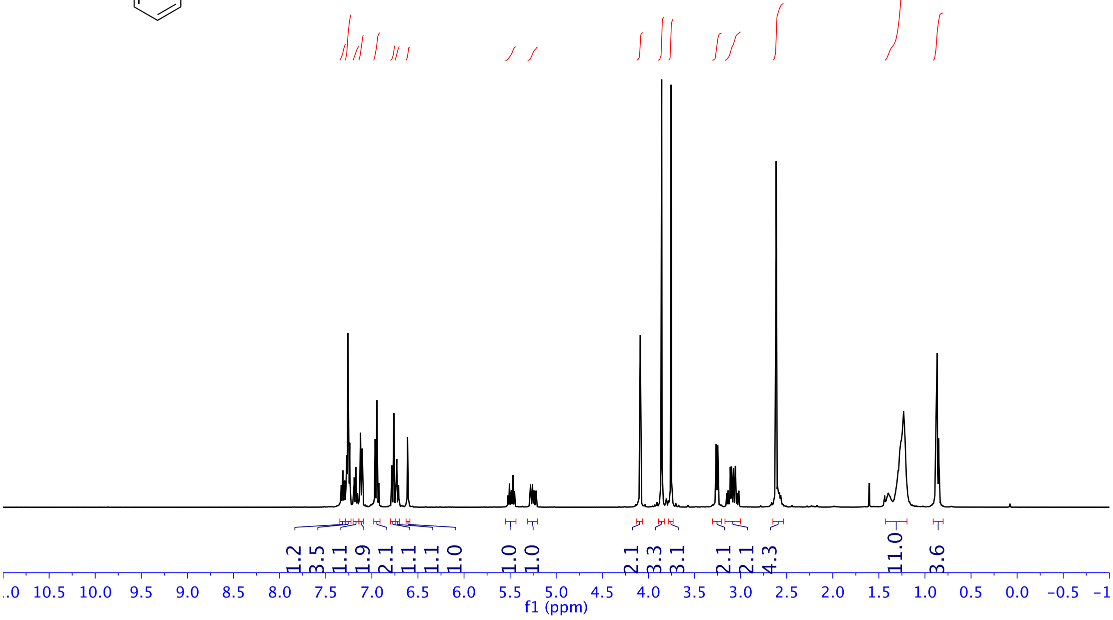

^{13}C NMR, 100 MHz, $CDCl_3$

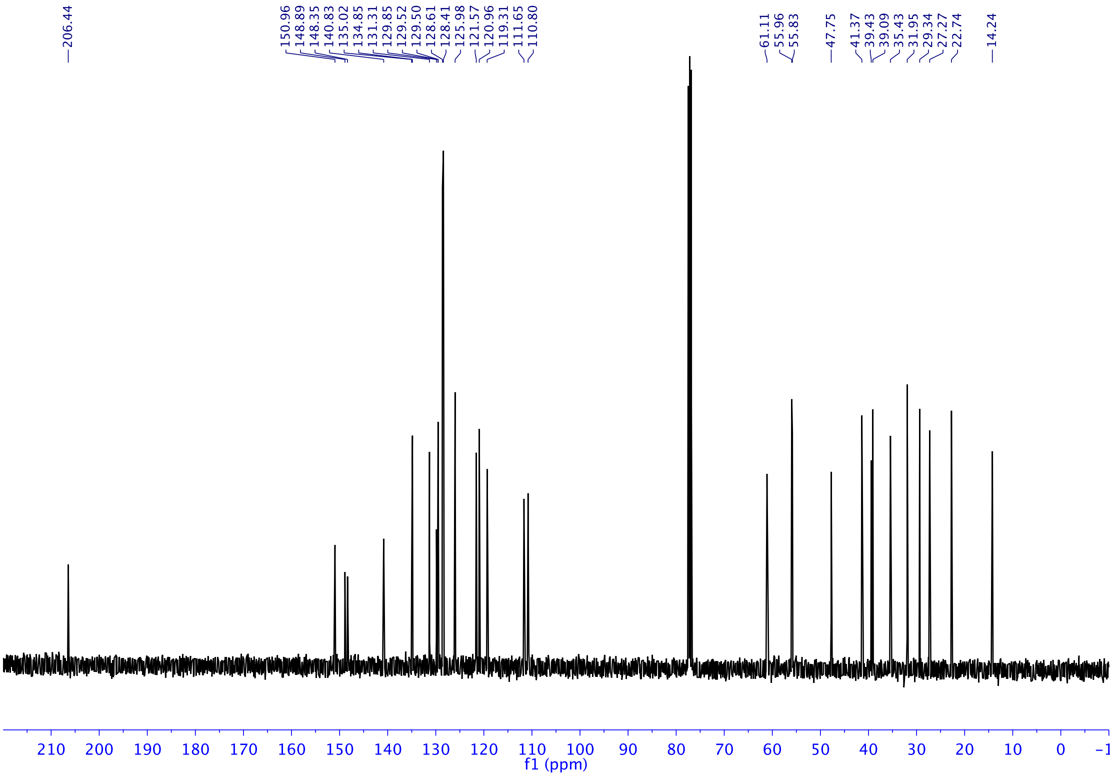

Figure 22. ^{1}H-NMR and ^{13}C-NMR spectra of compound **2t**

^{1}H NMR, 400 MHz, $CDCl_3$

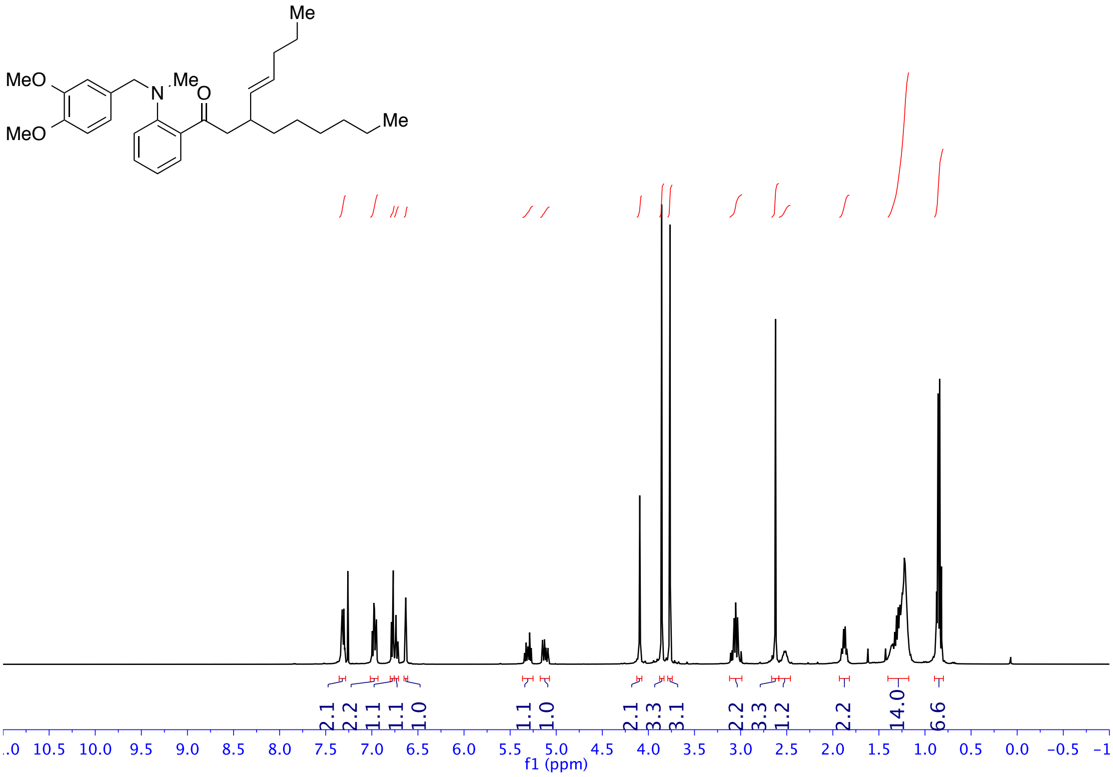

^{13}C NMR, 100 MHz, $CDCl_3$

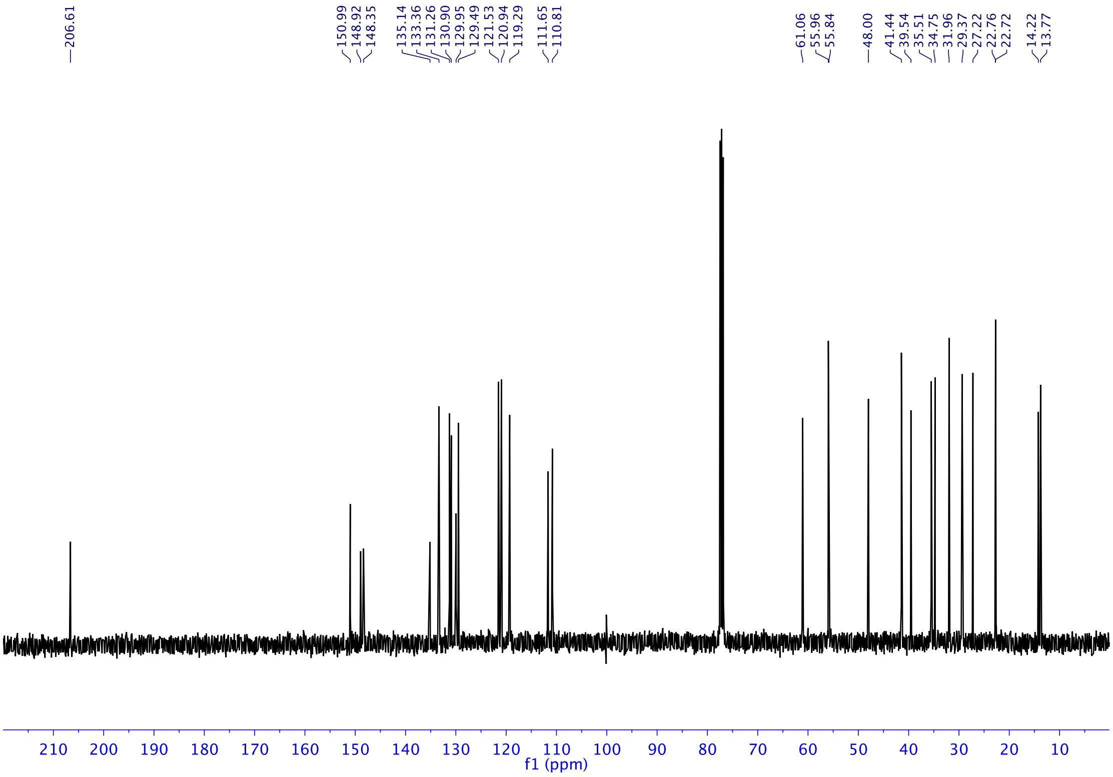

Figure 23. ^{1}H-NMR and ^{13}C-NMR spectra of compound **2u**

^{1}H NMR, 400 MHz, $CDCl_3$

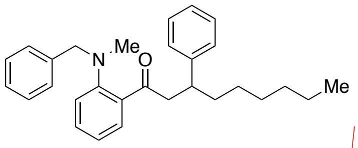

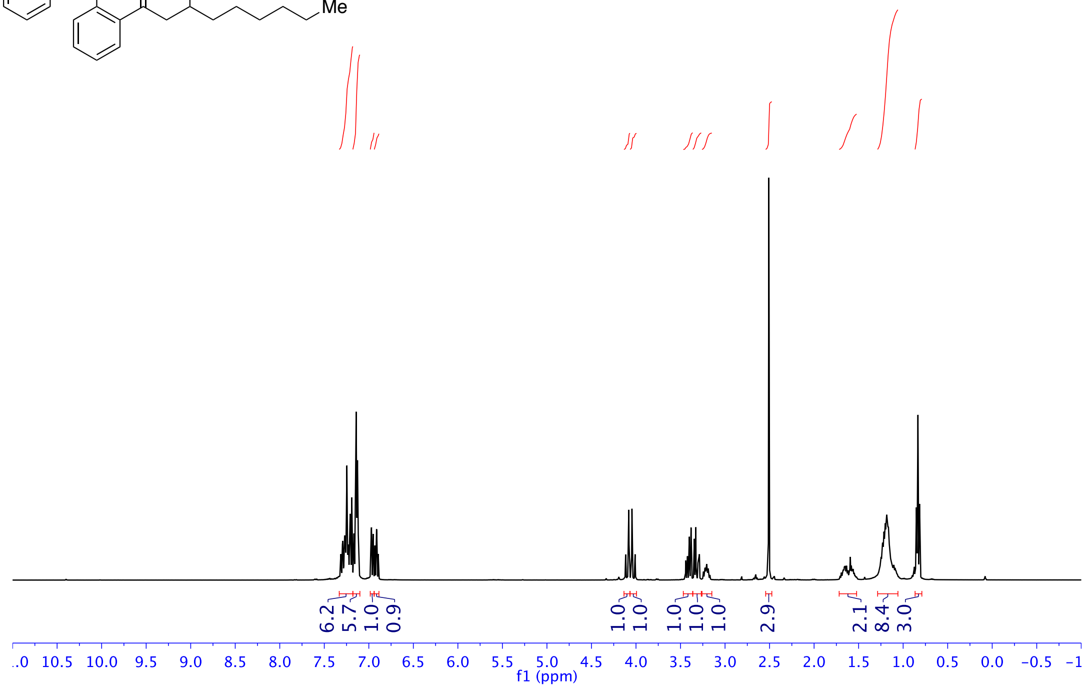

^{13}C NMR, 100 MHz, $CDCl_3$

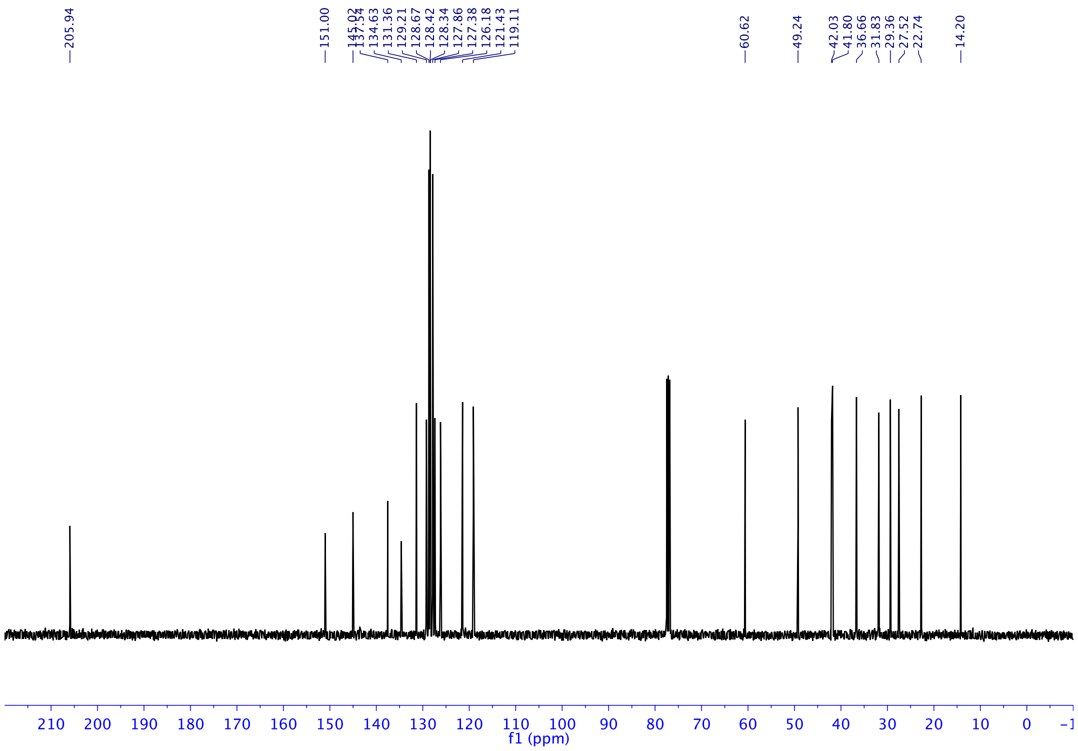

Figure 24. ^{1}H-NMR and ^{13}C-NMR spectra of compound **2v**

^{1}H NMR, 400 MHz, $CDCl_3$

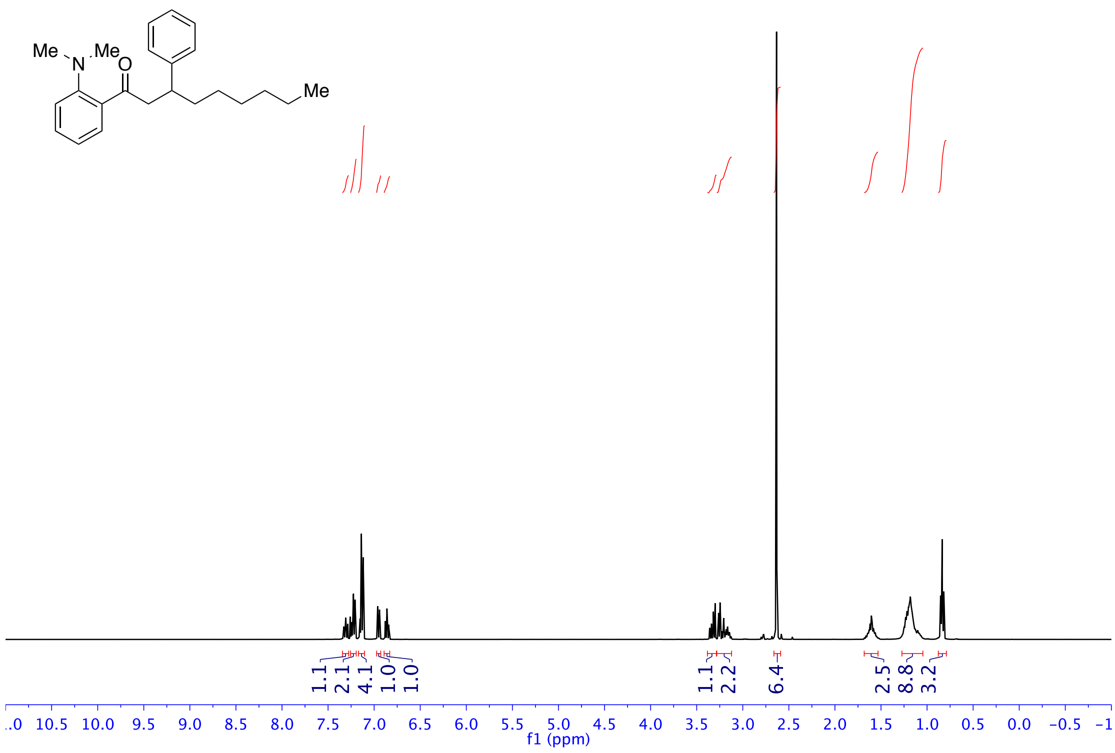

^{13}C NMR, 100 MHz, $CDCl_3$

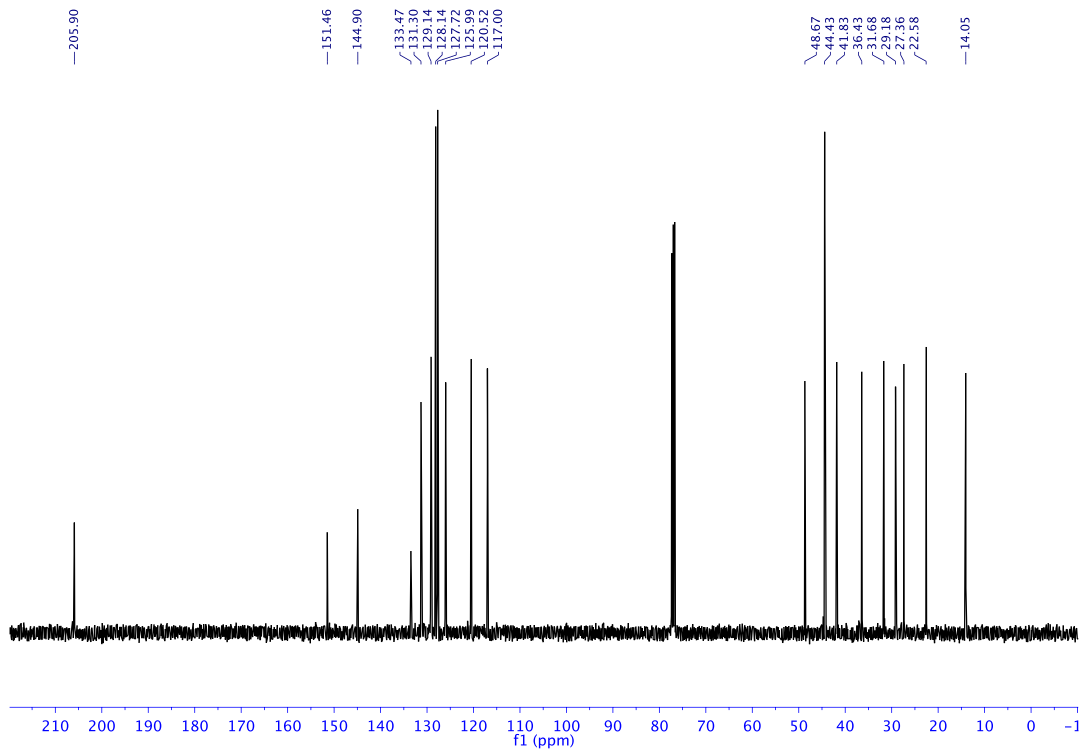

Figure 25. ^{1}H-NMR and ^{13}C-NMR spectra of compound **2w**

^{1}H NMR, 400 MHz, $CDCl_3$

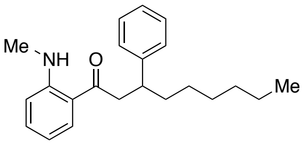

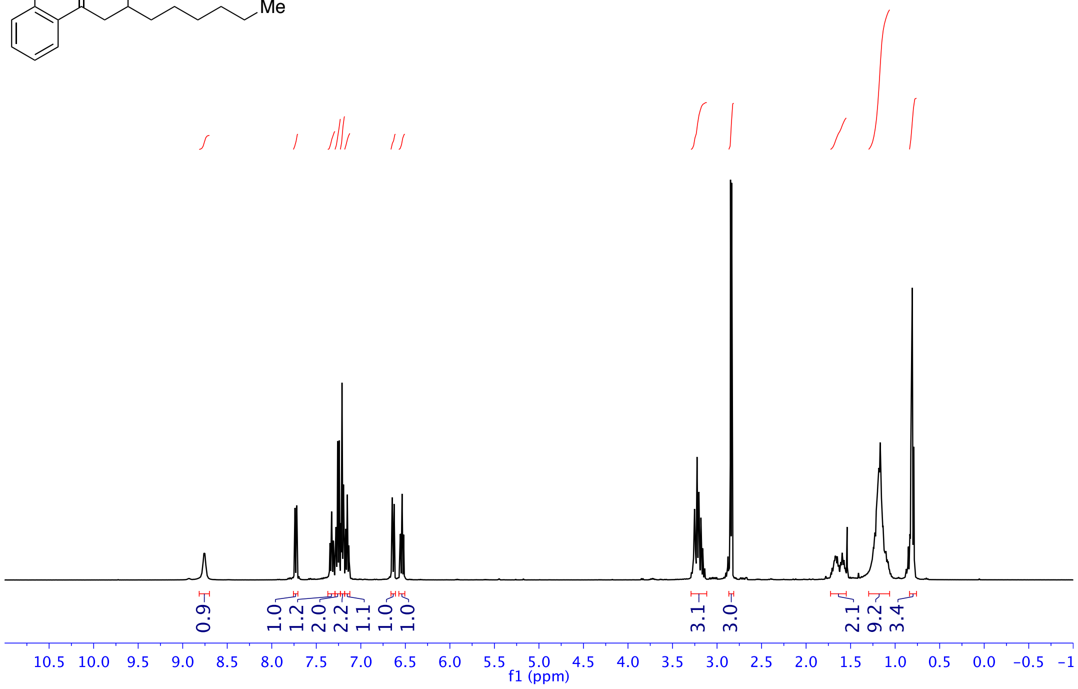

^{13}C NMR, 100 MHz, $CDCl_3$

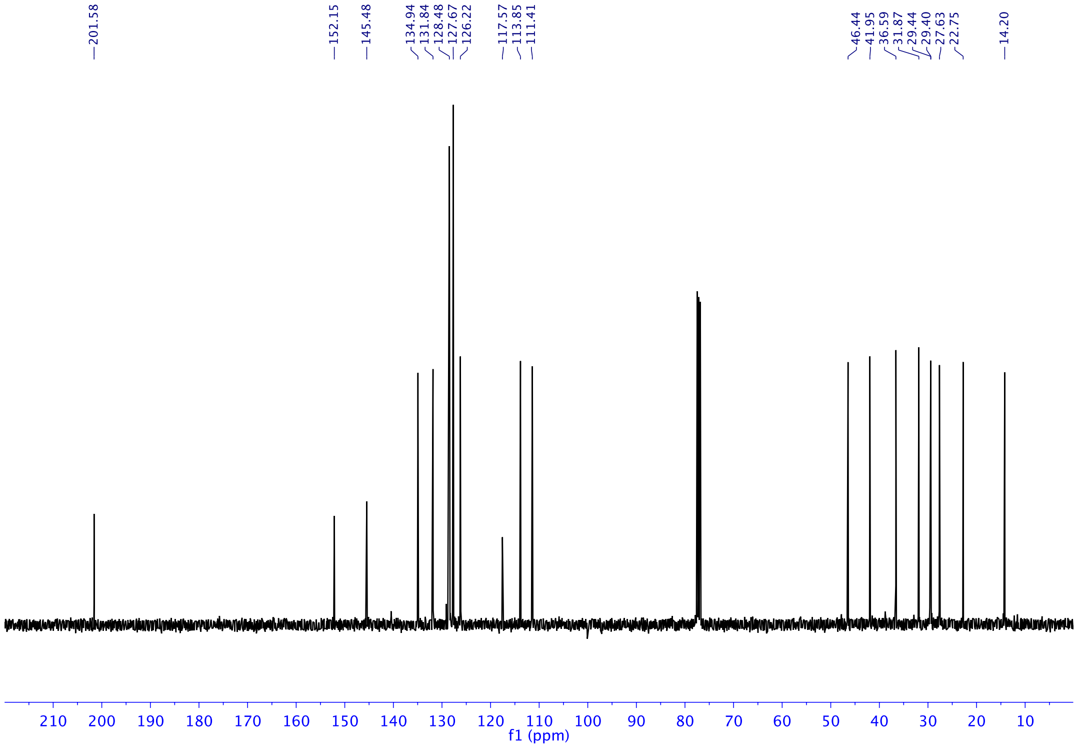

Figure 26. ^{1}H-NMR and ^{13}C-NMR spectra of compound **2x**

^{1}H NMR, 400 MHz, $CDCl_3$

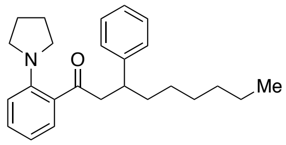

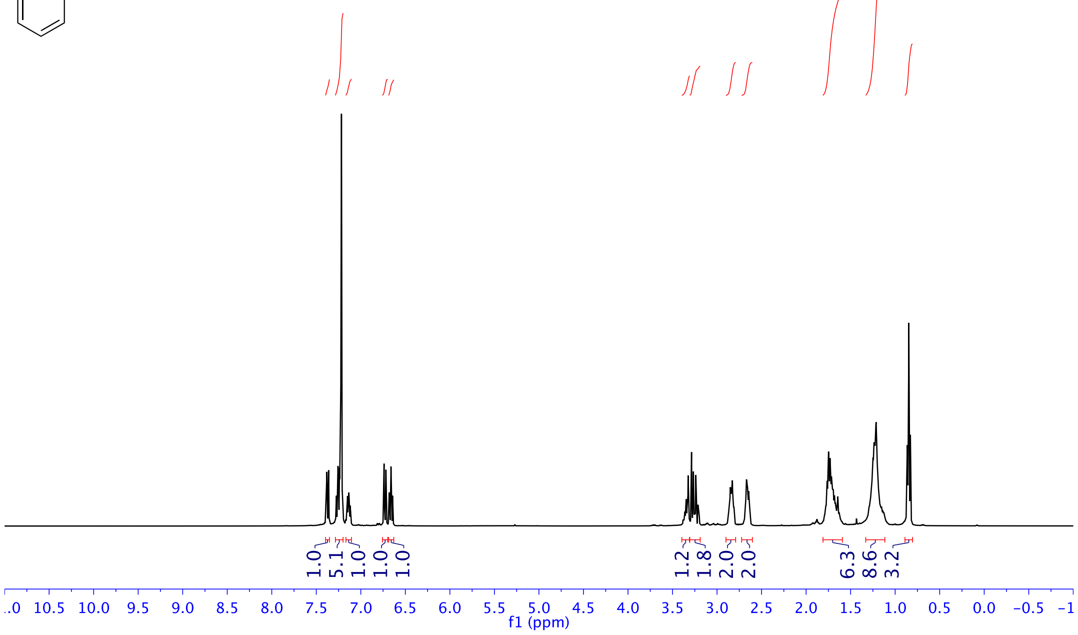

^{13}C NMR, 100 MHz, $CDCl_3$

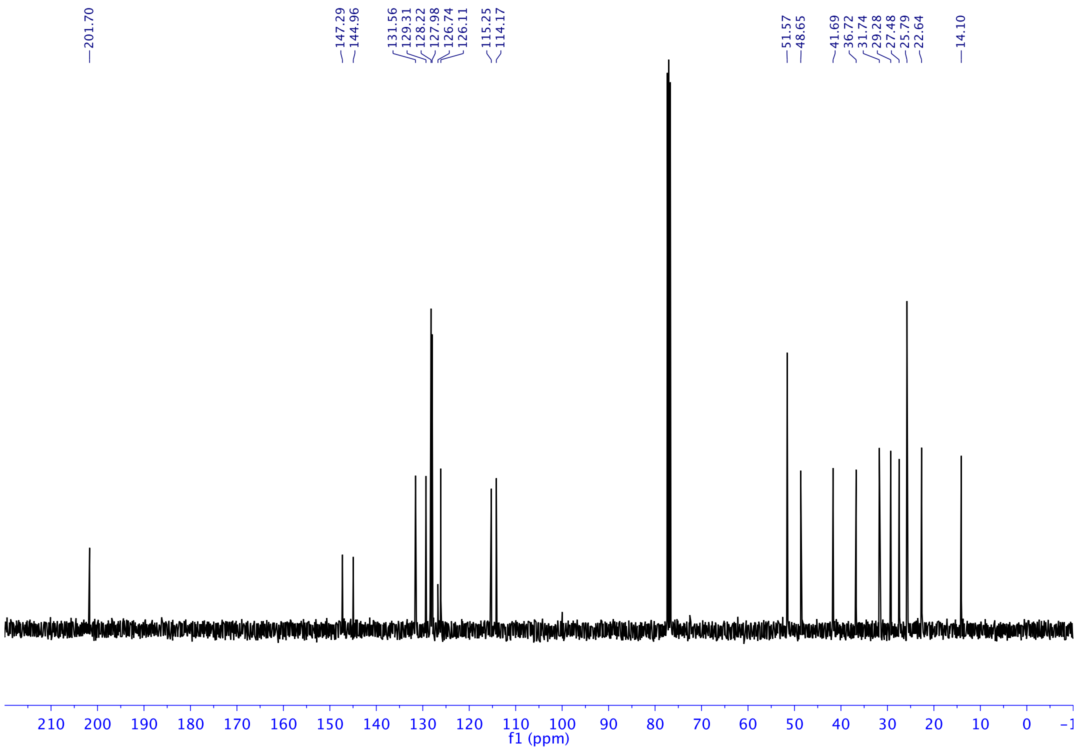

Figure 27. ^{1}H-NMR and ^{13}C-NMR spectra of compound **2y**

^{1}H NMR, 400 MHz, $CDCl_3$

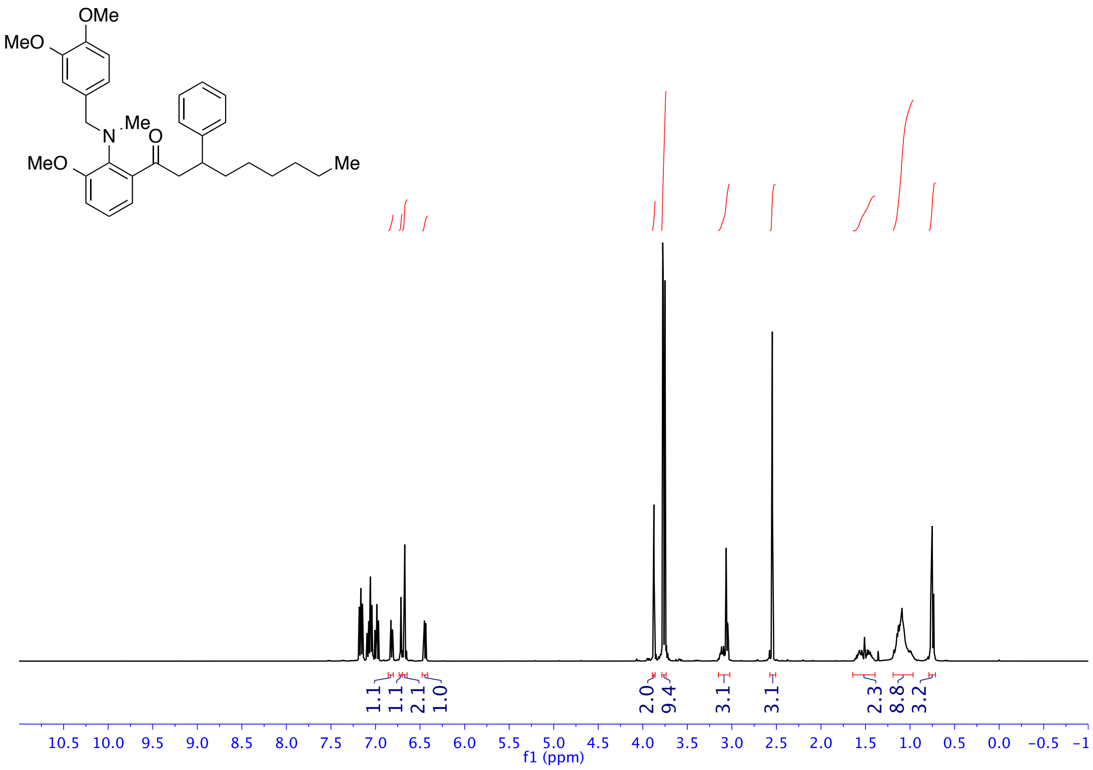

^{13}C NMR, 100 MHz, $CDCl_3$

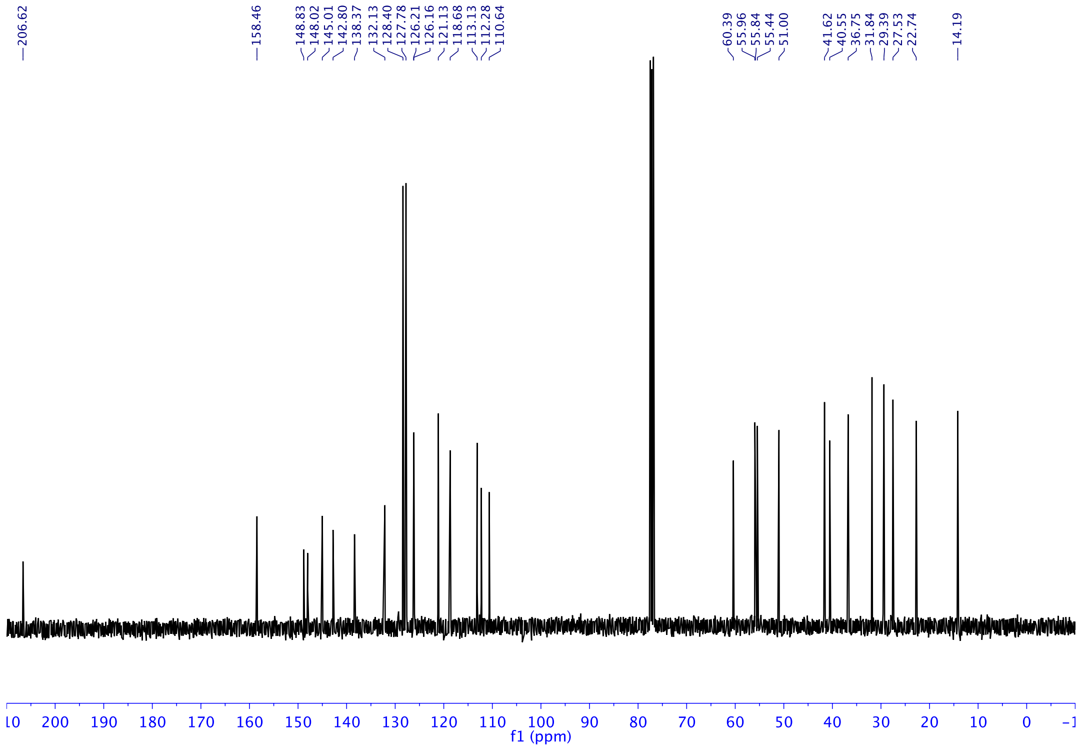

Figure 28. ^{1}H-NMR and ^{13}C-NMR spectra of compound **2z**

^{1}H NMR, 400 MHz, $CDCl_3$

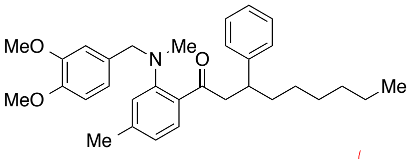

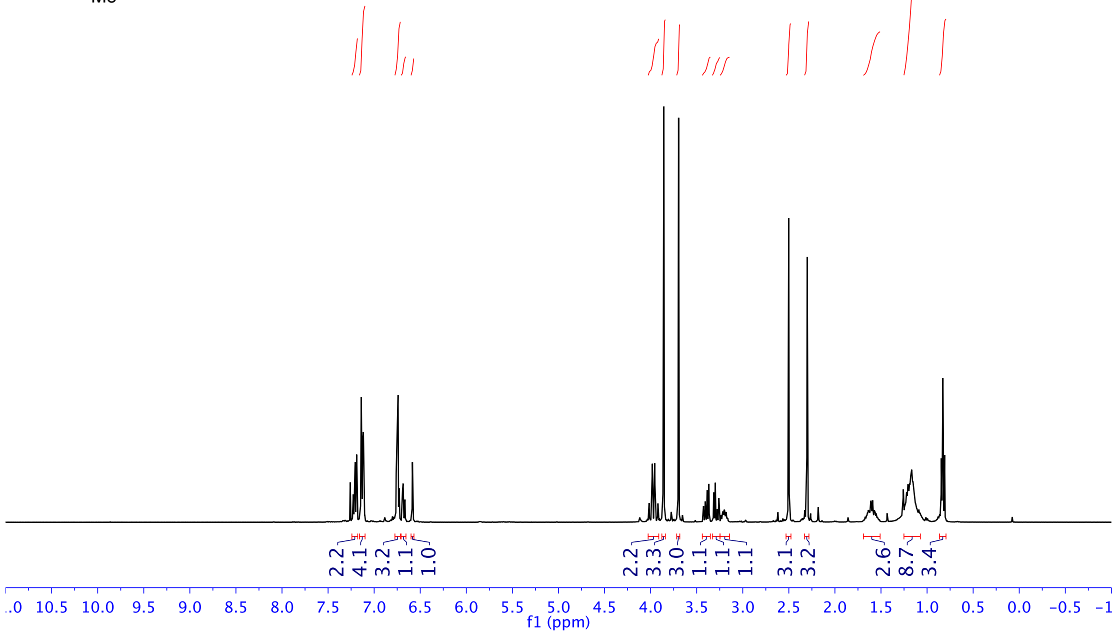

^{13}C NMR, 100 MHz, $CDCl_3$

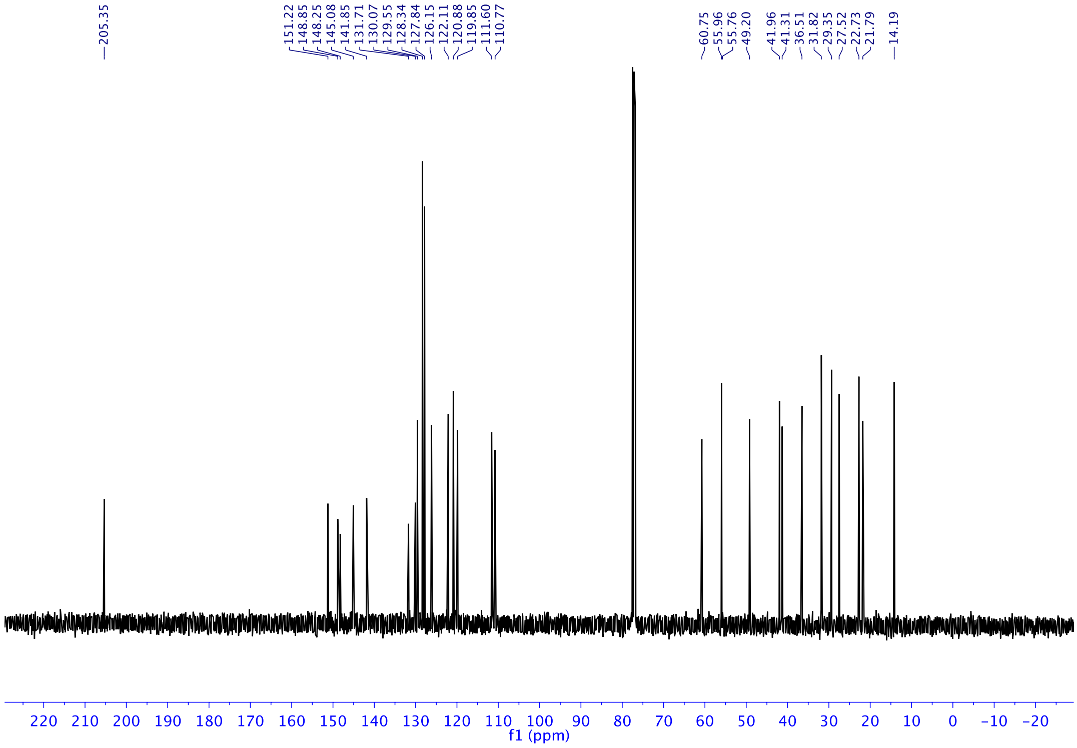

Figure 29. ^{1}H-NMR and ^{13}C-NMR spectra of compound **2aa**

^{1}H NMR, 400 MHz, $CDCl_3$

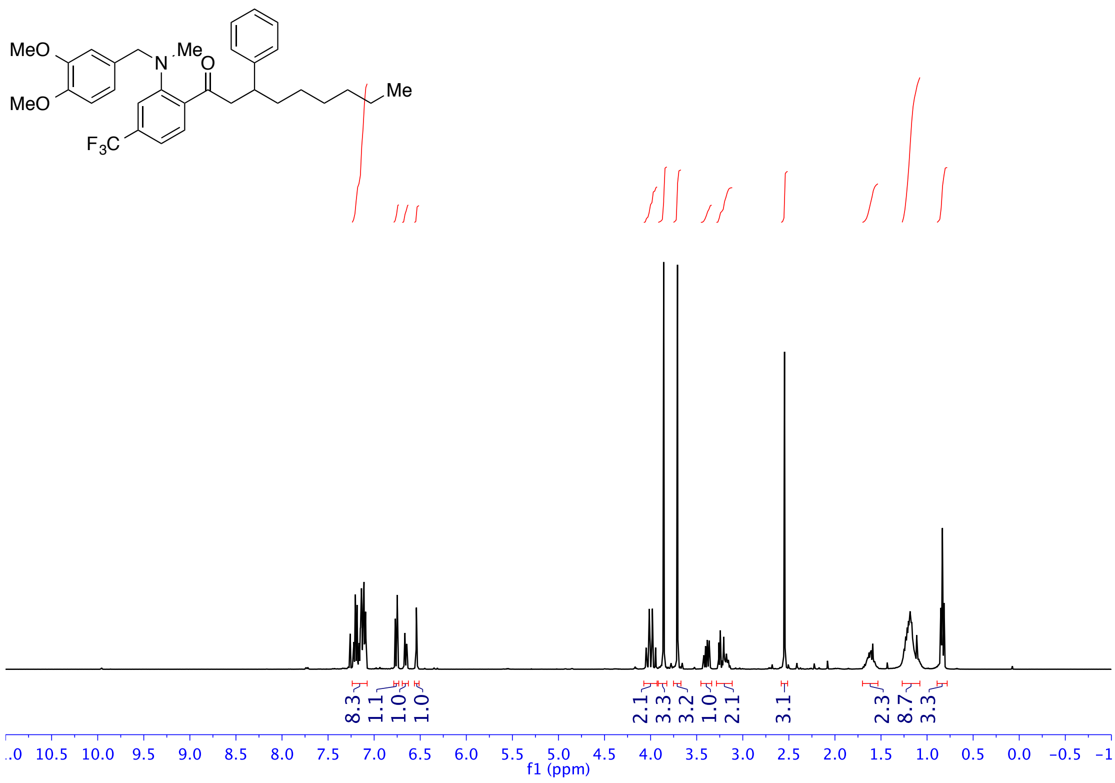

^{13}C NMR, 100 MHz, $CDCl_3$

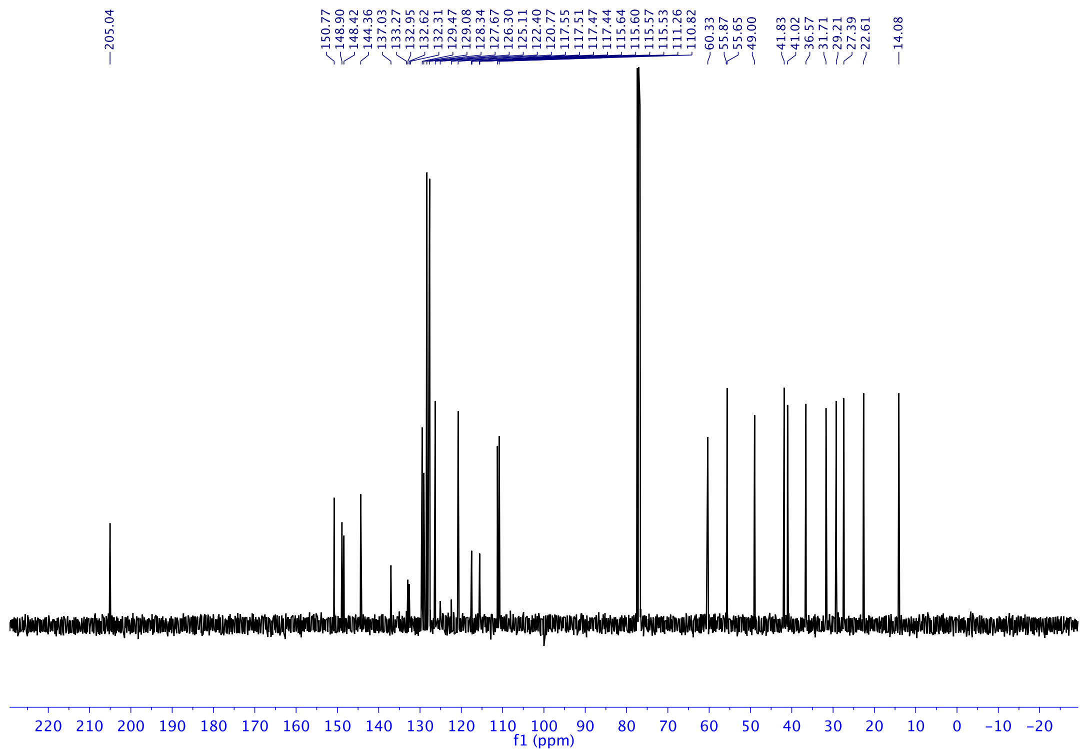

Figure 30. ^{1}H-NMR and ^{13}C-NMR spectra of compound **2ab**

^{1}H NMR, 400 MHz, $CDCl_3$

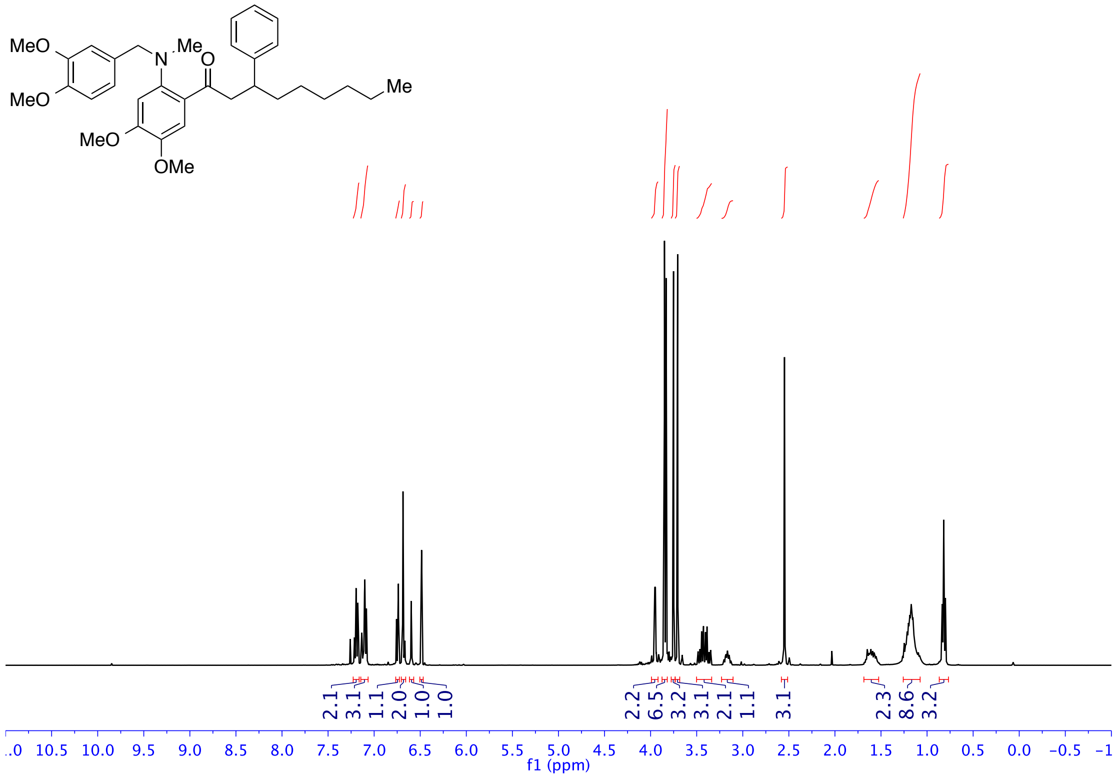

^{13}C NMR, 100 MHz, $CDCl_3$

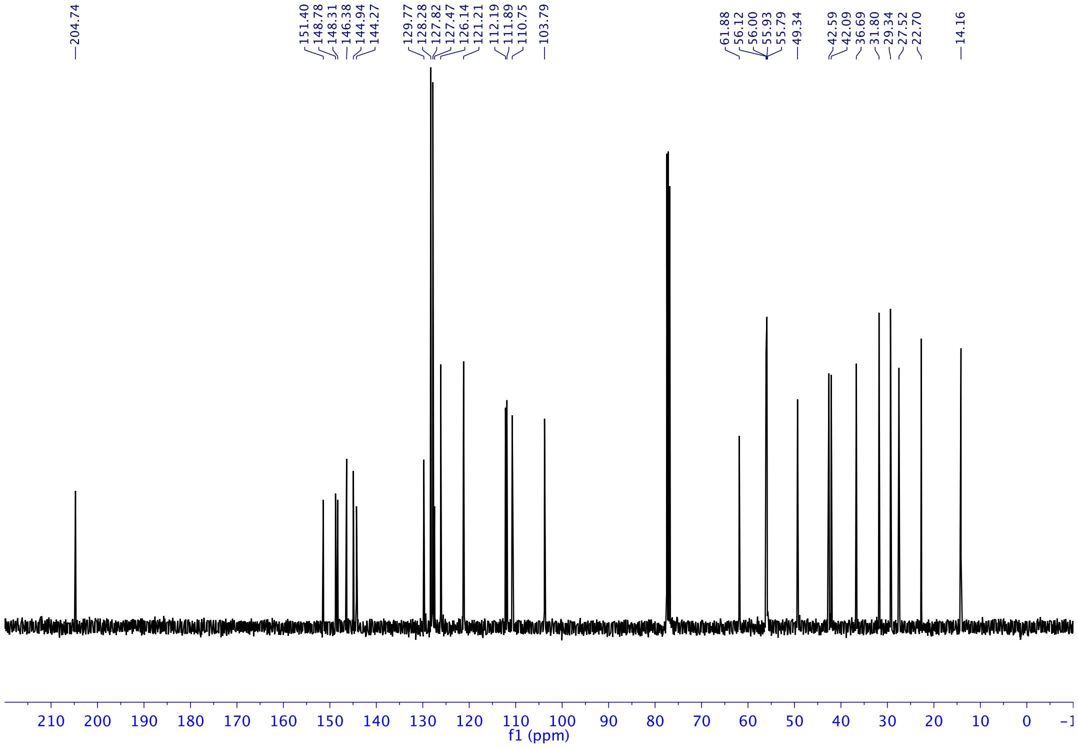

Figure 31. ^{1}H-NMR and ^{13}C-NMR spectra of compound **2ac**

^{1}H NMR, 400 MHz, $CDCl_3$

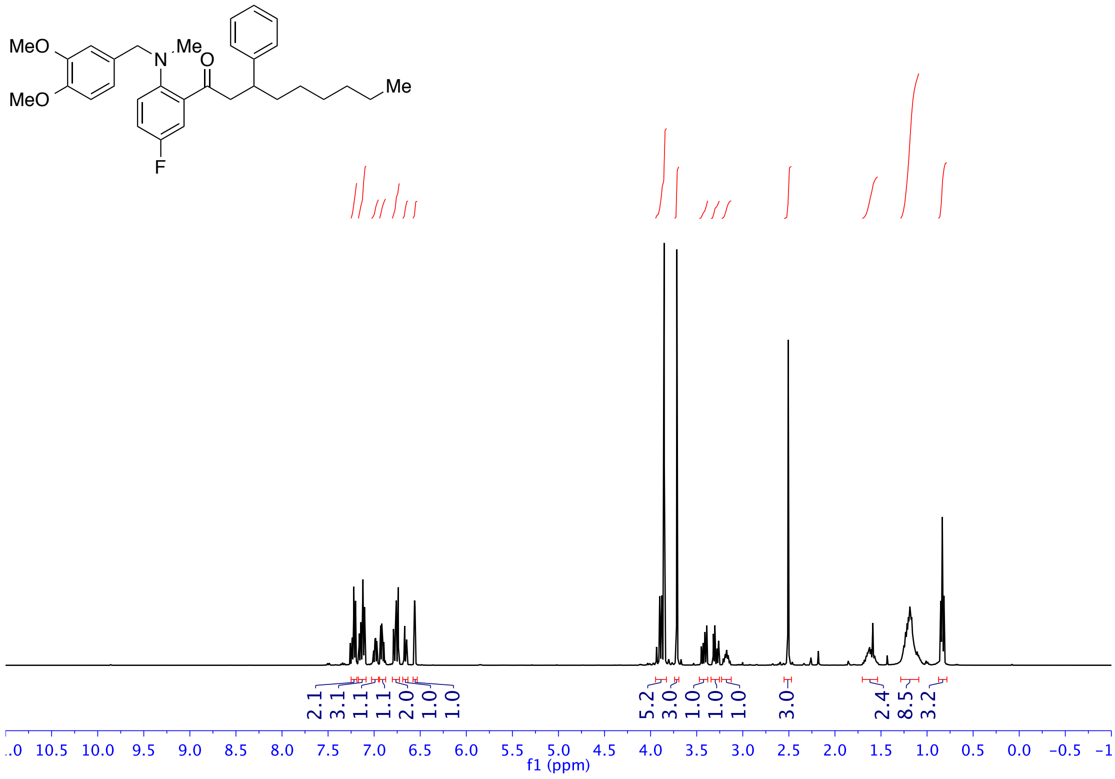

^{13}C NMR, 100 MHz, $CDCl_3$

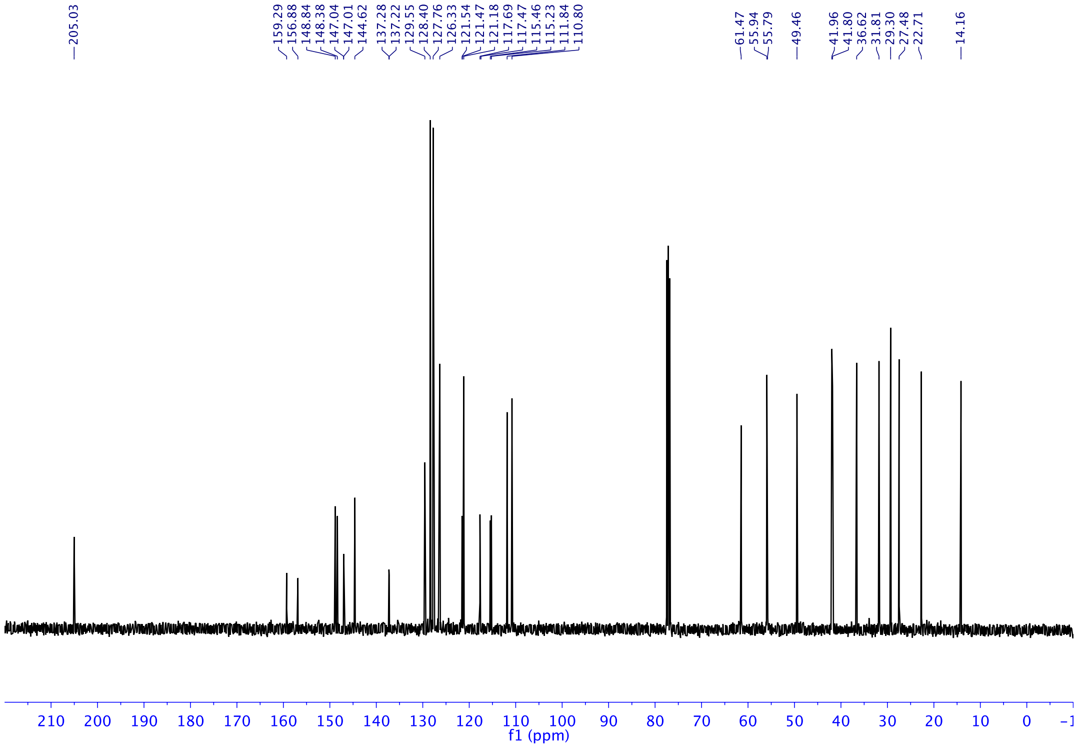

Figure 32. ^{1}H-NMR and ^{13}C-NMR spectra of compound **2ad**

^{1}H NMR, 400 MHz, $CDCl_3$

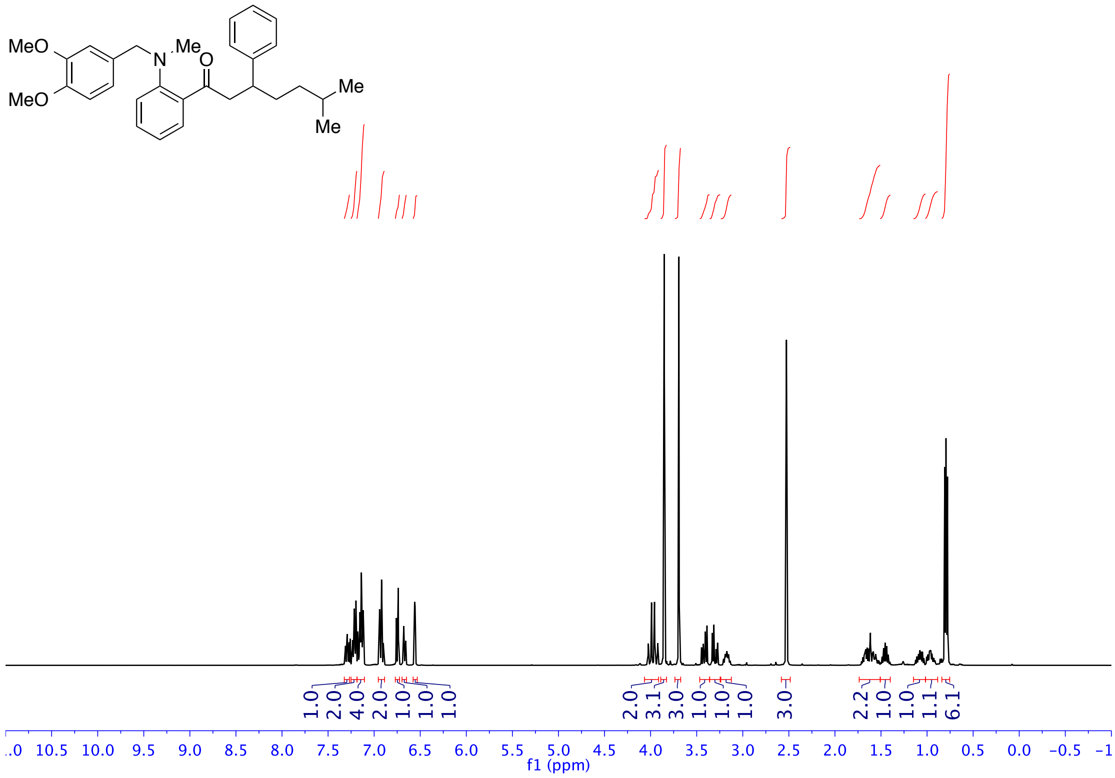

^{13}C NMR, 100 MHz, $CDCl_3$

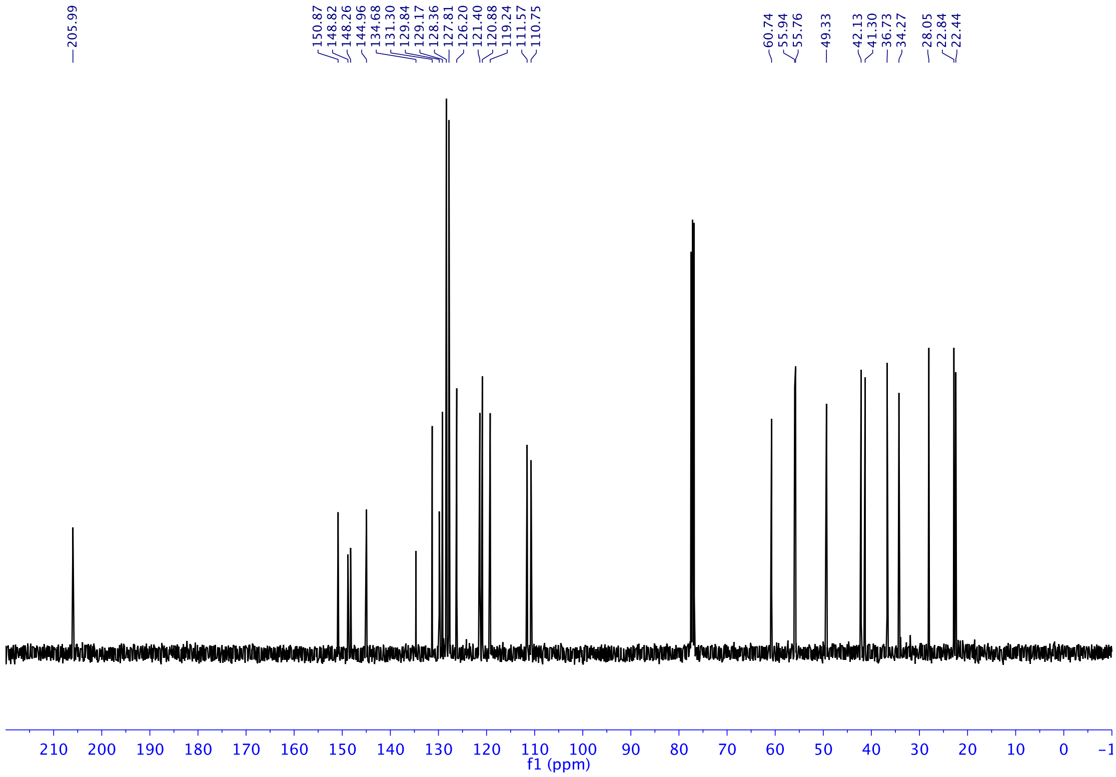

Figure 33. ^{1}H-NMR and ^{13}C-NMR spectra of compound **2ae**

^{1}H NMR, 400 MHz, $CDCl_3$

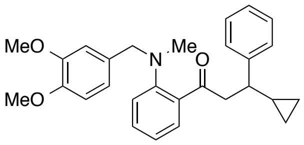

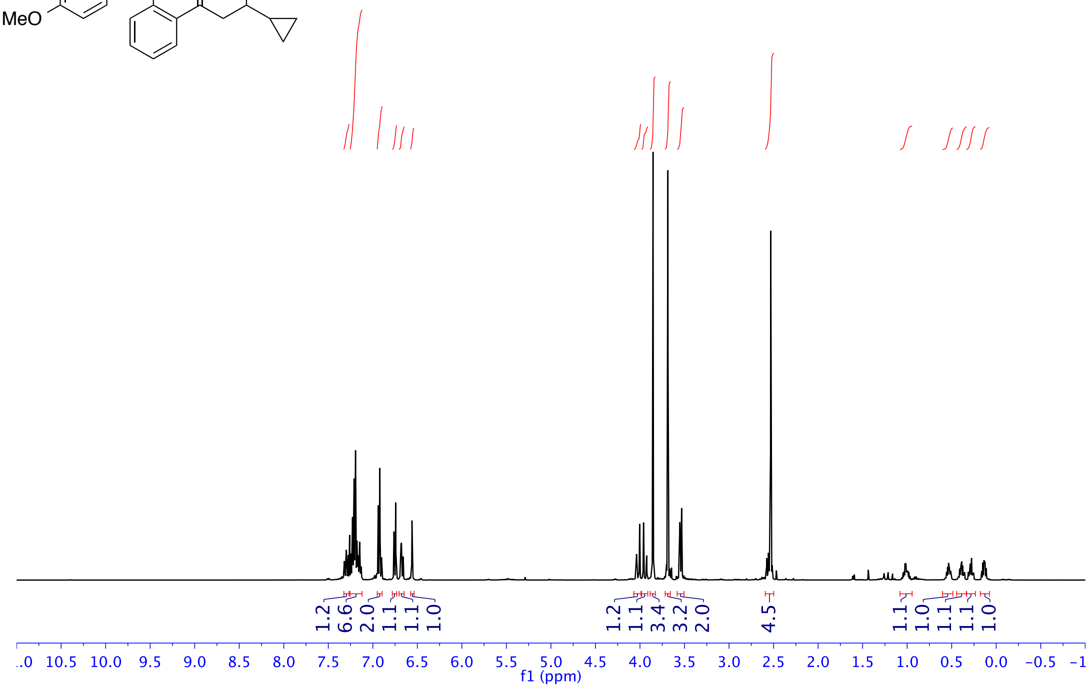

^{13}C NMR, 100 MHz, $CDCl_3$

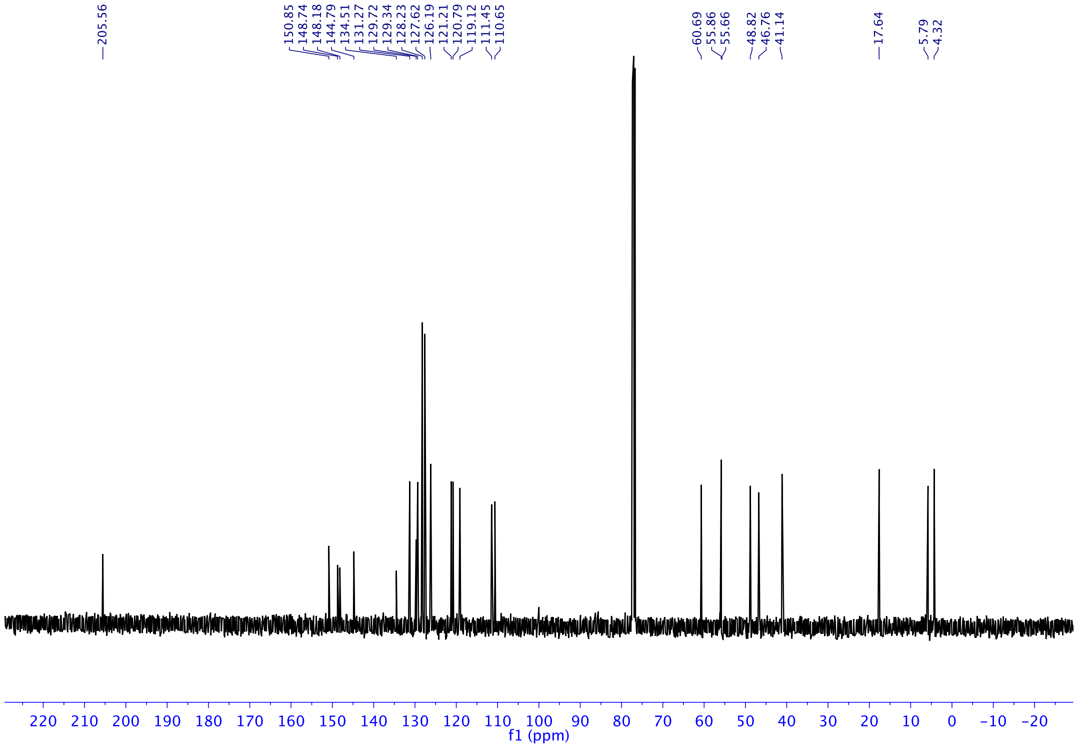

Figure 34. ^{1}H-NMR and ^{13}C-NMR spectra of compound **2af**

^{1}H NMR, 400 MHz, $CDCl_3$

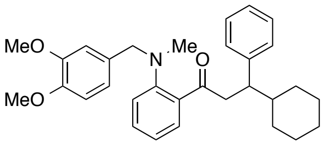

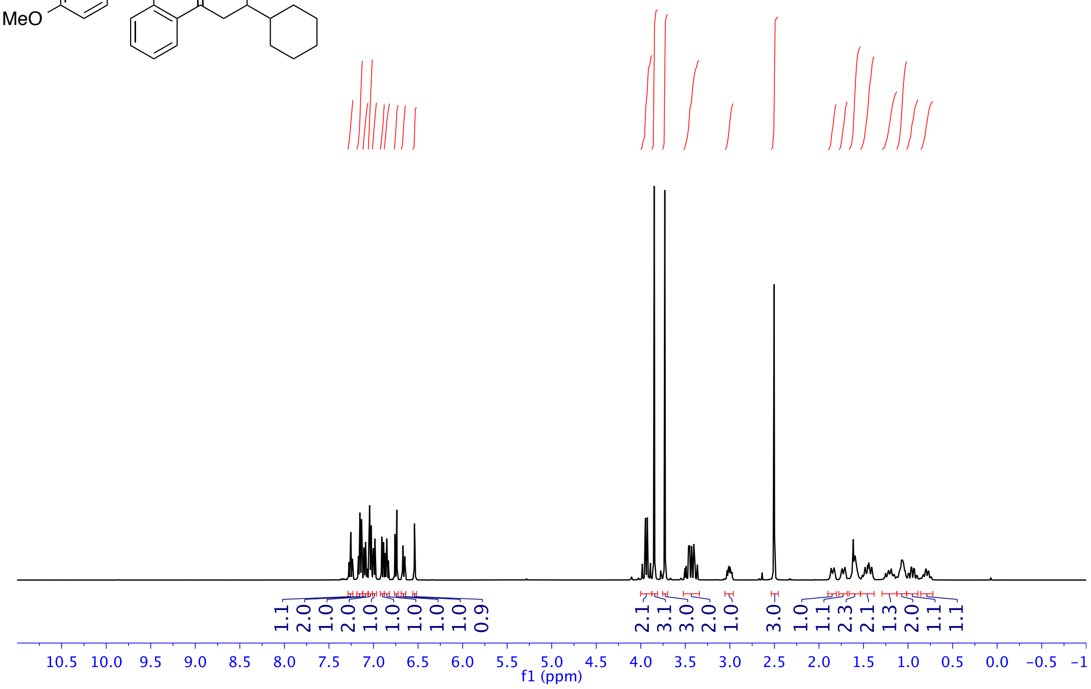

^{13}C NMR, 100 MHz, $CDCl_3$

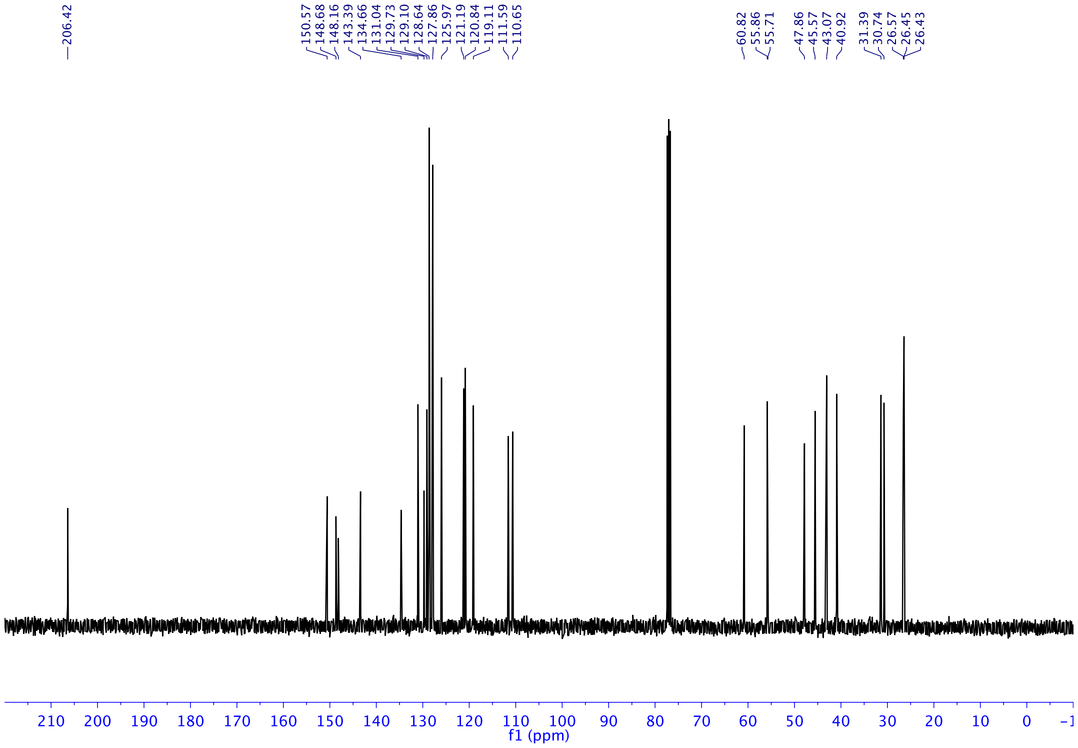

Figure 35. ^{1}H-NMR and ^{13}C-NMR spectra of compound **2ag**

^{1}H NMR, 400 MHz, $CDCl_3$

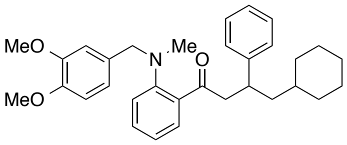

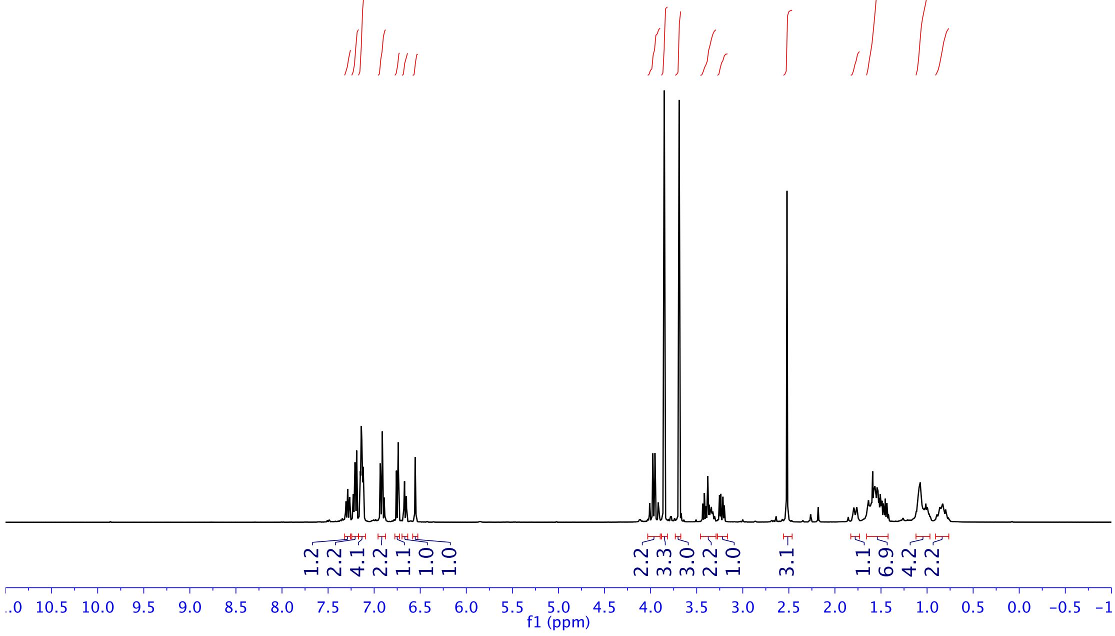

^{13}C NMR, 100 MHz, $CDCl_3$

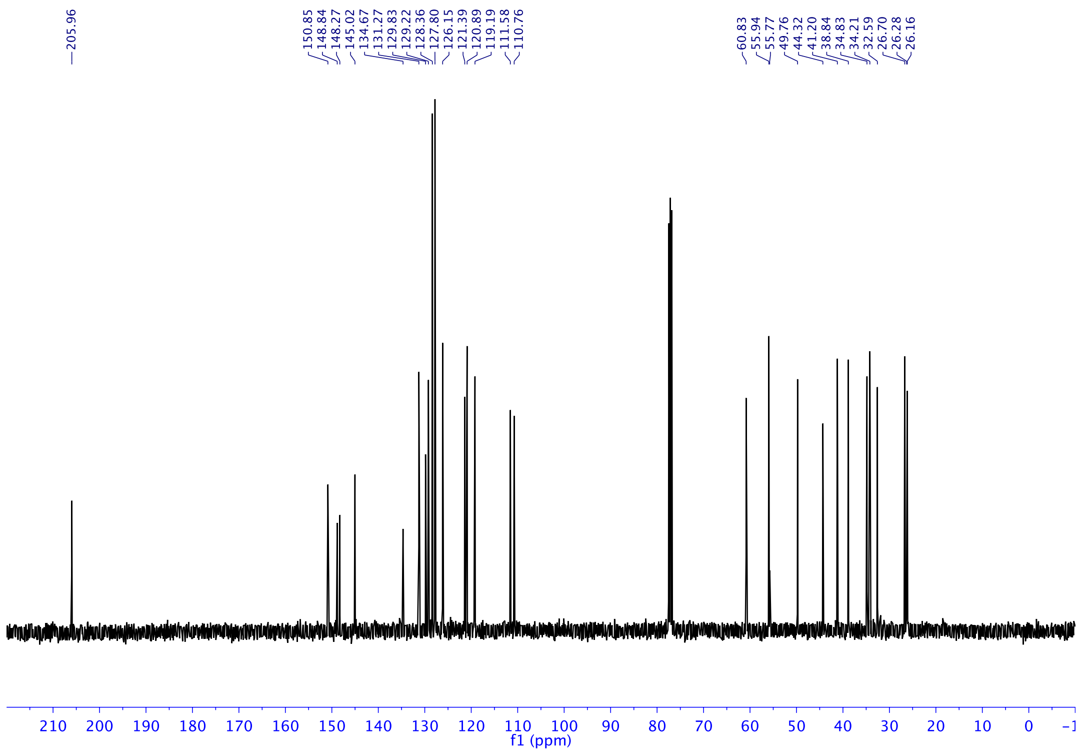

Figure 36. ^{1}H-NMR and ^{13}C-NMR spectra of compound **2ah**

^{1}H NMR, 400 MHz, $CDCl_3$

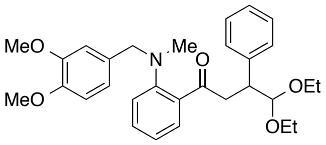

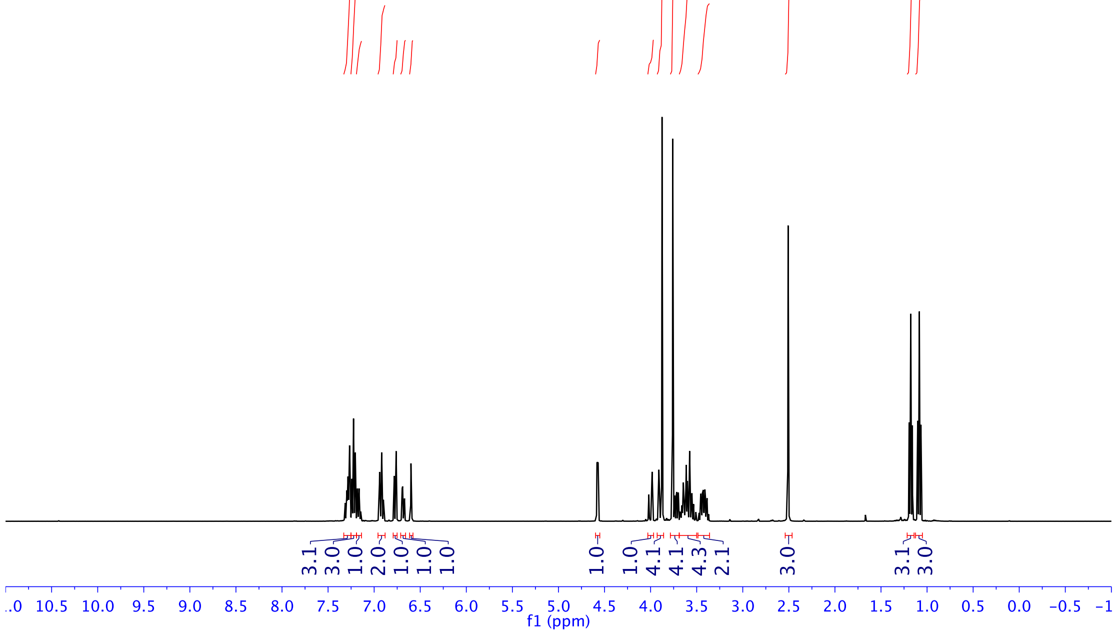

^{13}C NMR, 100 MHz, $CDCl_3$

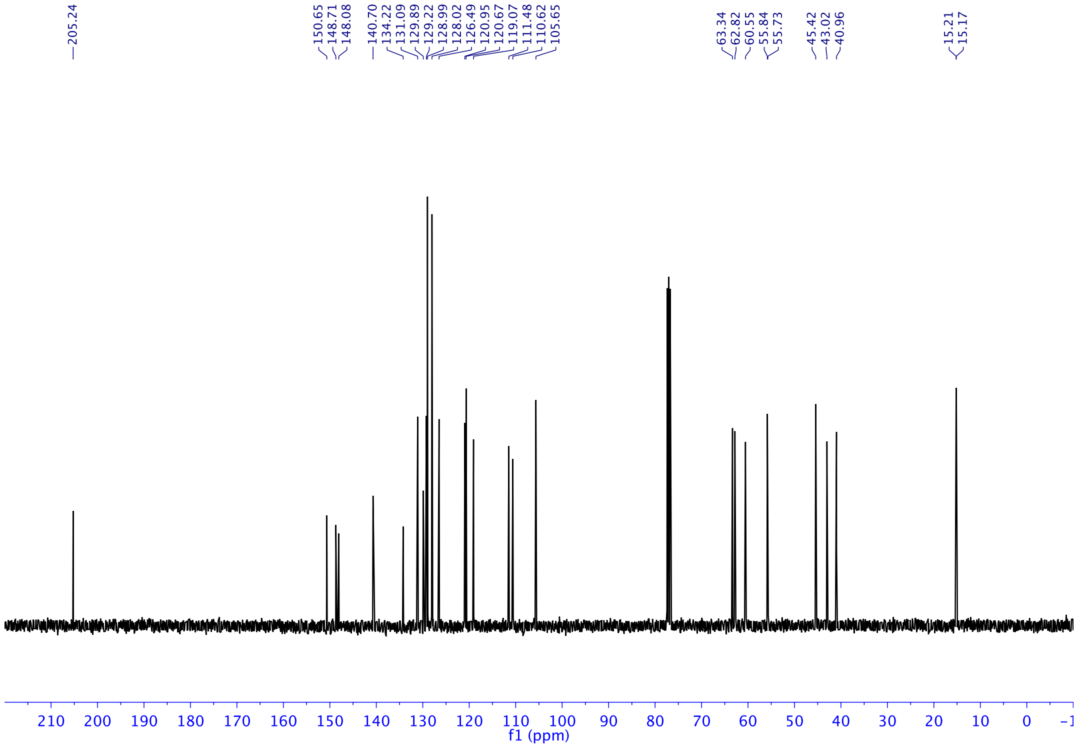

Figure 37. ^{1}H-NMR and ^{13}C-NMR spectra of compound **2ai**

^{1}H NMR, 400 MHz, $CDCl_3$

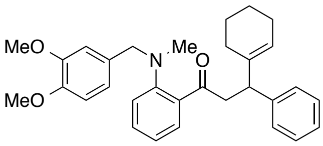

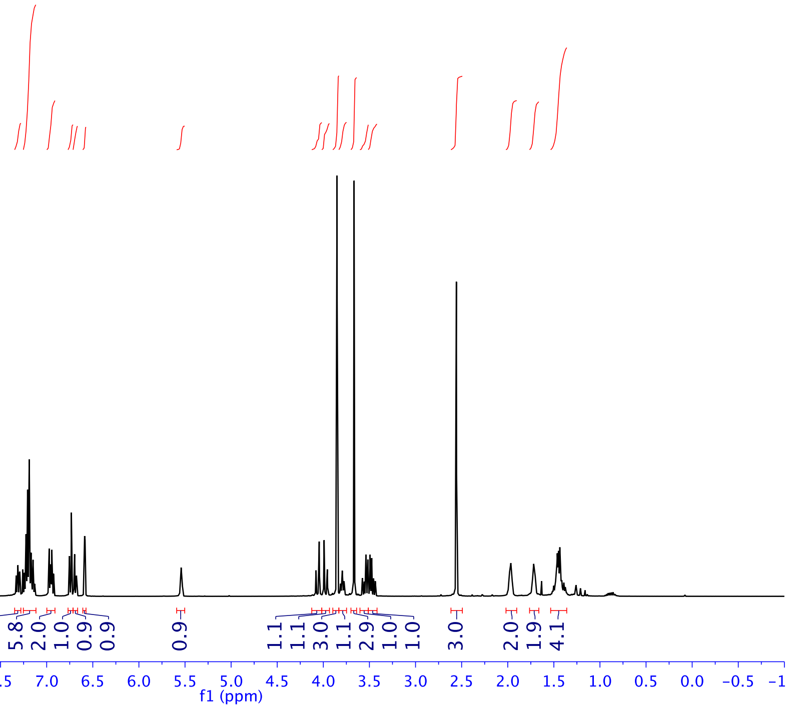

^{13}C NMR, 100 MHz, $CDCl_3$

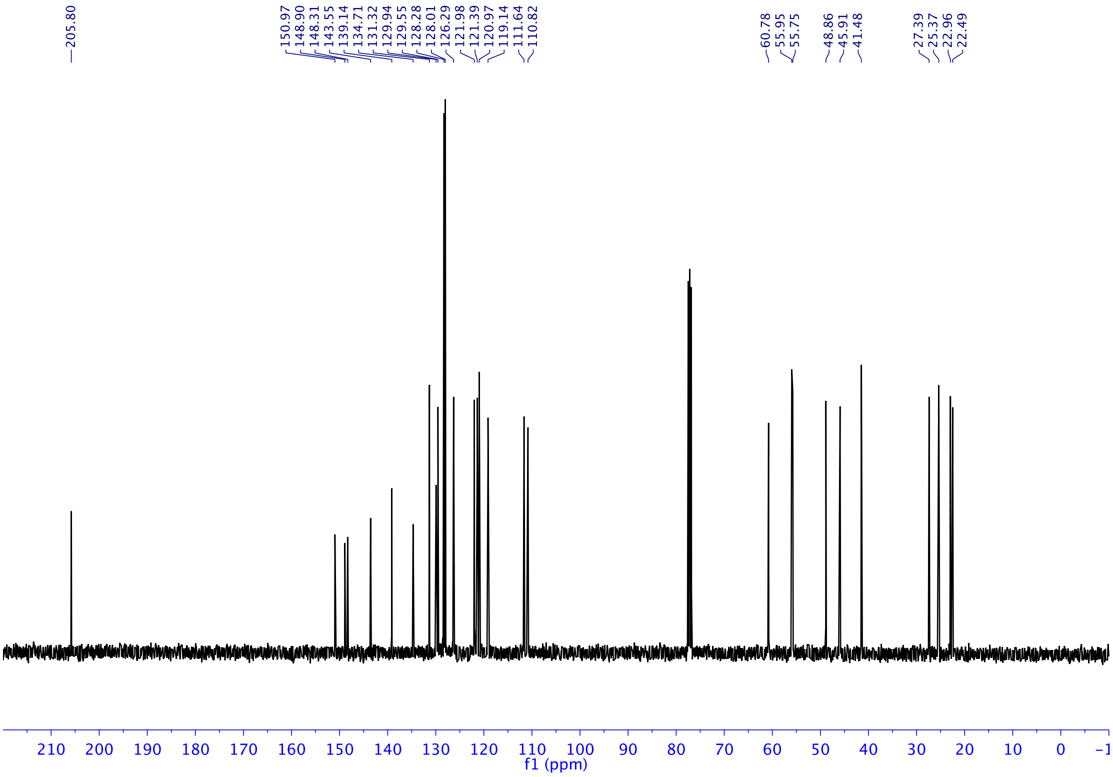

Figure 38. ^{1}H-NMR and ^{13}C-NMR spectra of compound **2aj**

^{1}H NMR, 400 MHz, $CDCl_3$

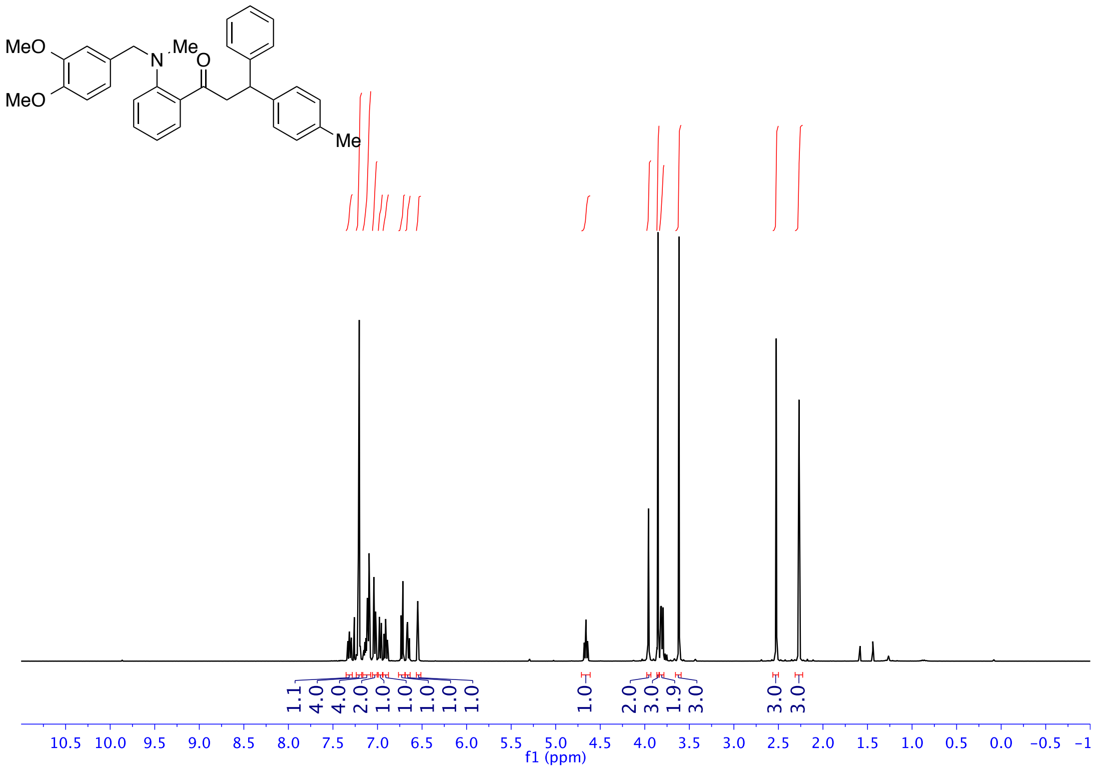

^{13}C NMR, 100 MHz, $CDCl_3$

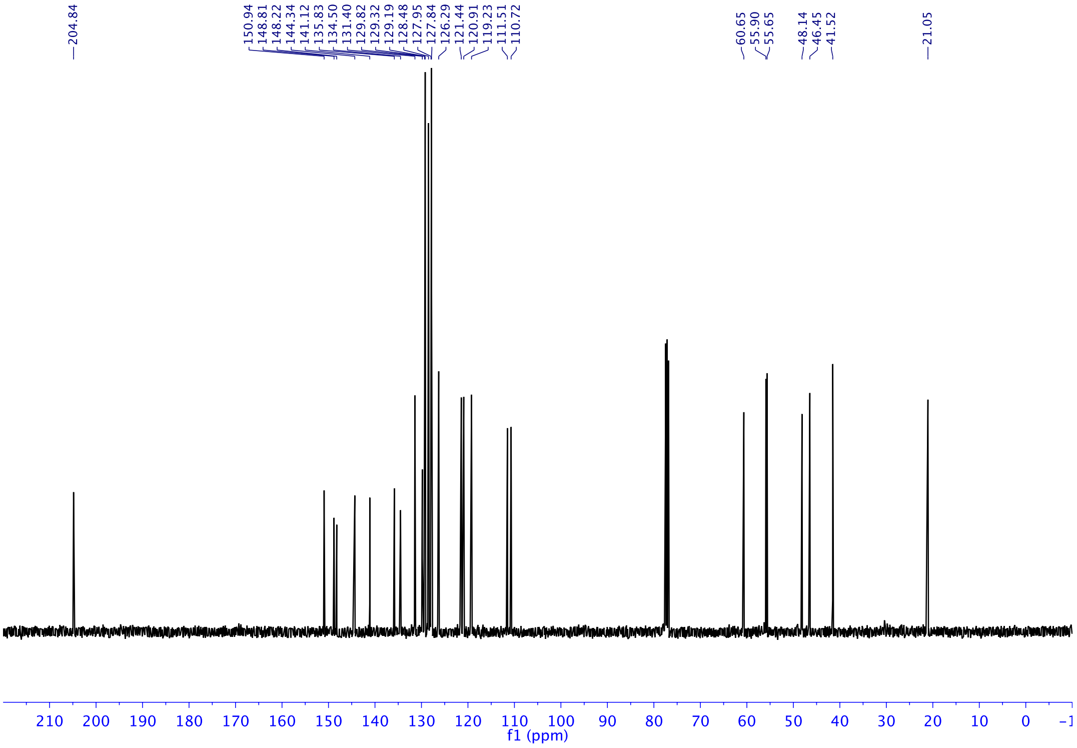

Figure 39. ^{1}H-NMR and ^{13}C-NMR spectra of compound **2ak**

^{1}H NMR, 400 MHz, $CDCl_3$

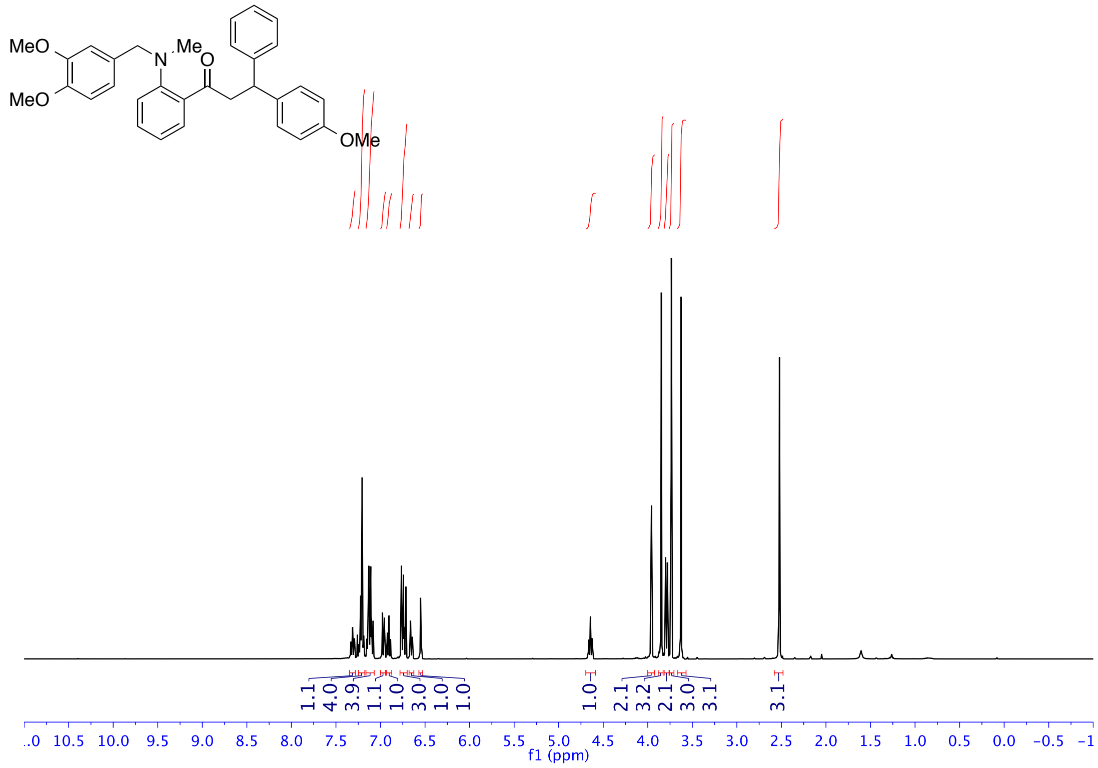

^{13}C NMR, 100 MHz, $CDCl_3$

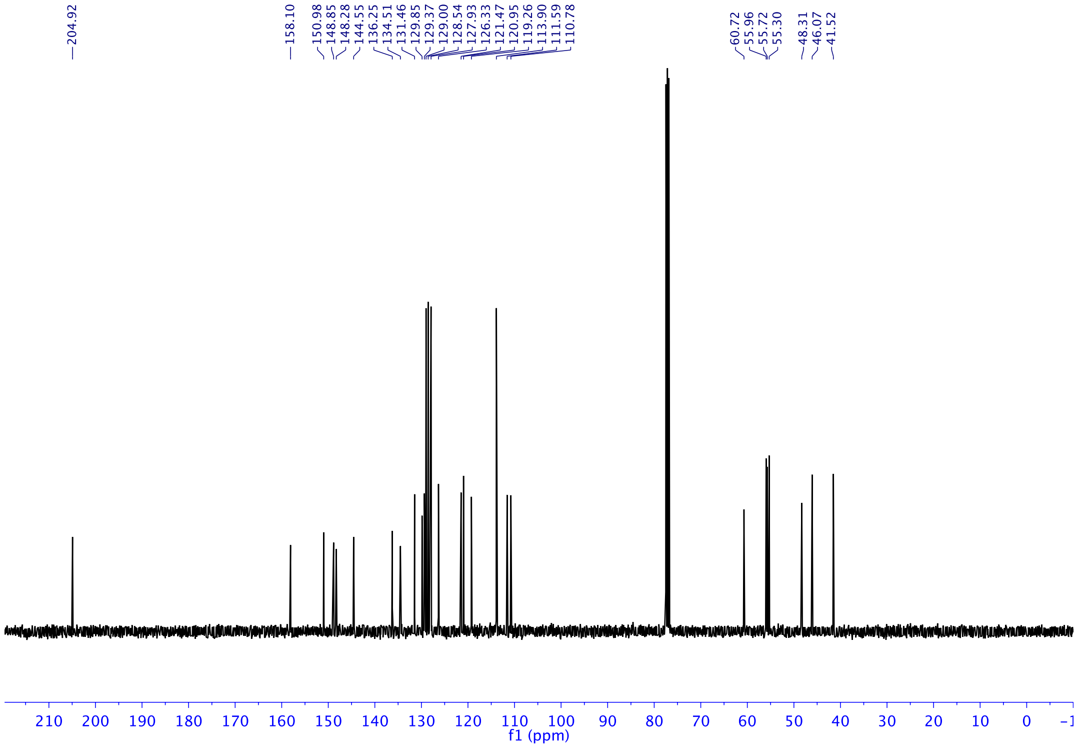

Figure 40. ^{1}H-NMR and ^{13}C-NMR spectra of compound **2al**

^{1}H NMR, 400 MHz, $CDCl_3$

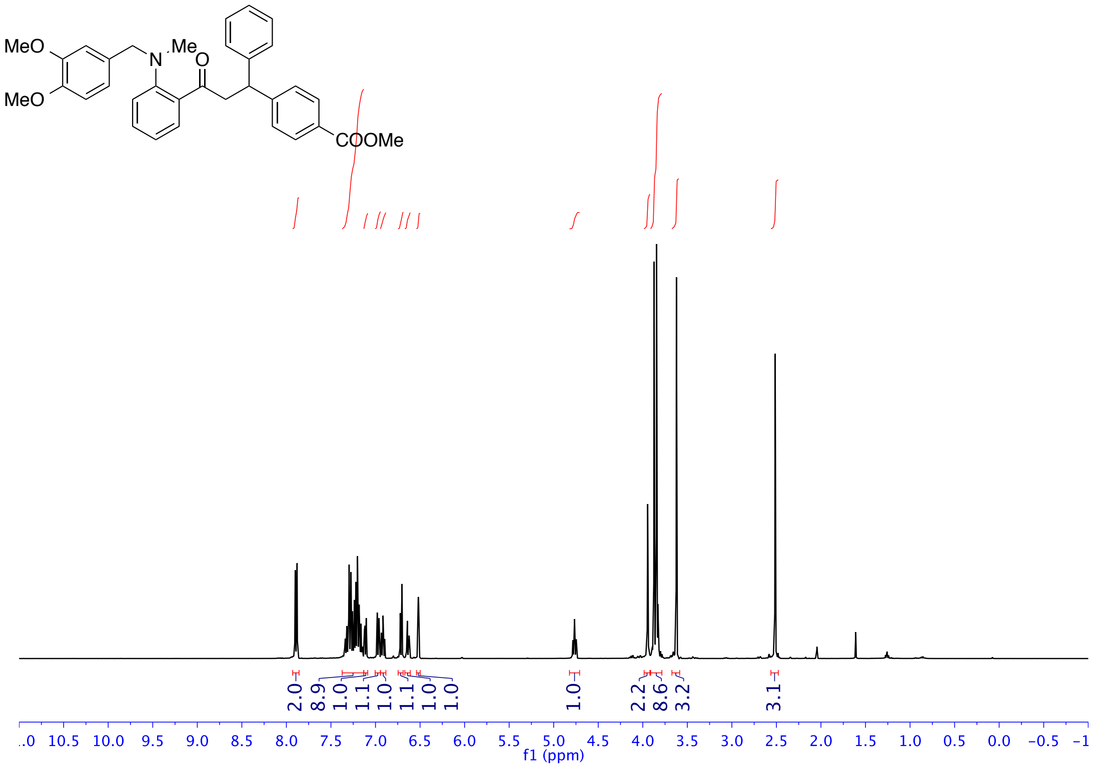

^{13}C NMR, 100 MHz, $CDCl_3$

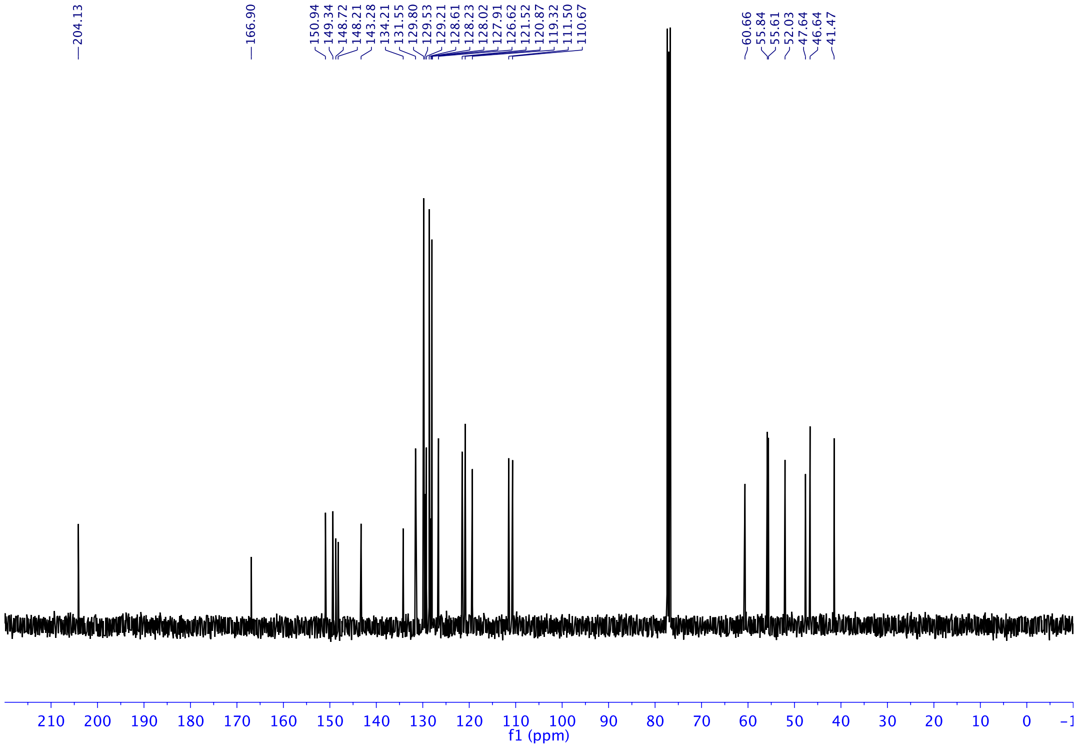

Figure 41. ^{1}H-NMR and ^{13}C-NMR spectra of compound **2am**

^{1}H NMR, 400 MHz, $CDCl_3$

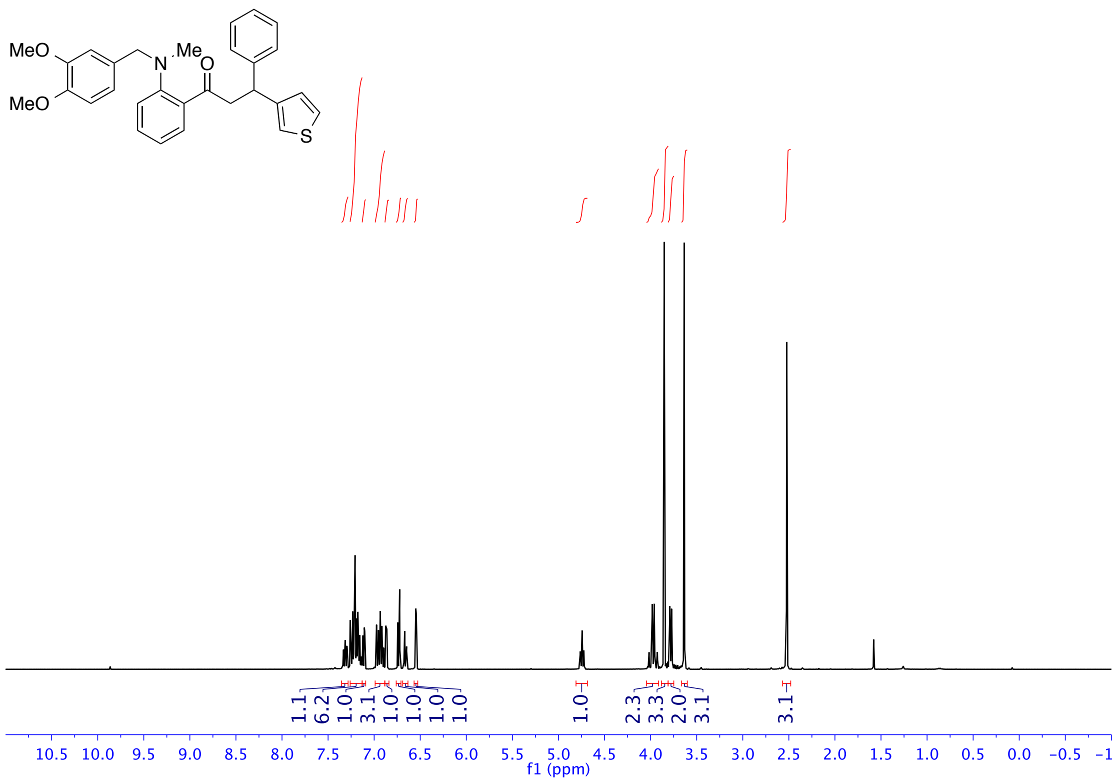

^{13}C NMR, 100 MHz, $CDCl_3$

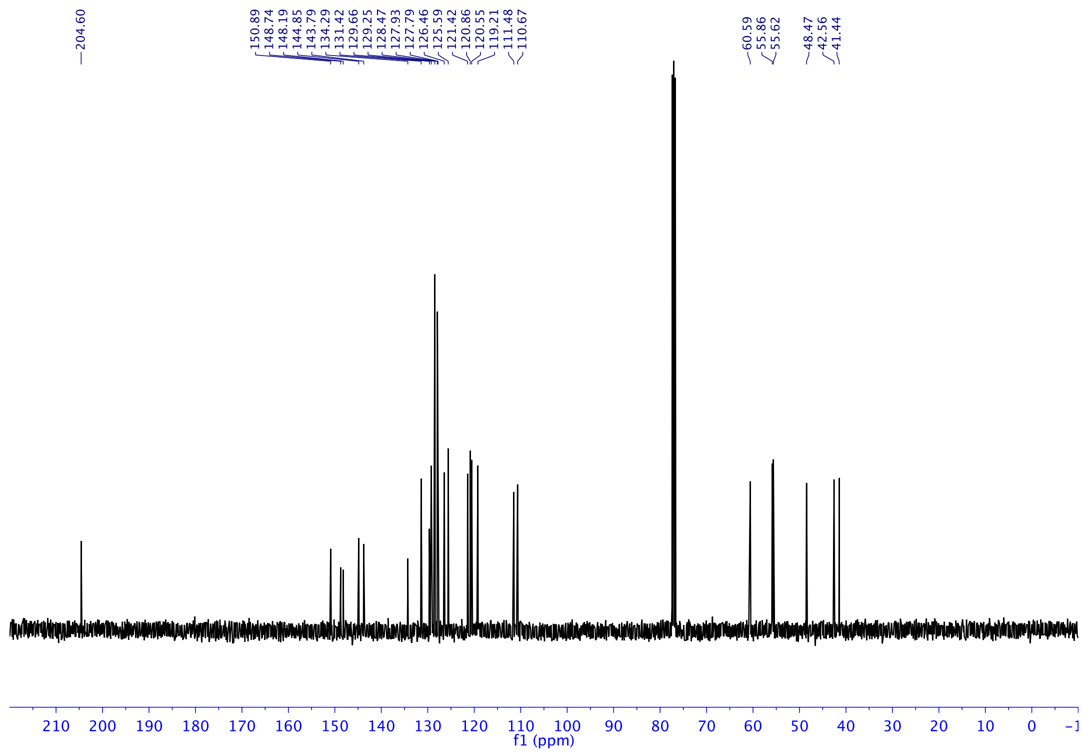

Figure 42. ^{1}H-NMR and ^{13}C-NMR spectra of compound **2an**

^{1}H NMR, 400 MHz, $CDCl_3$

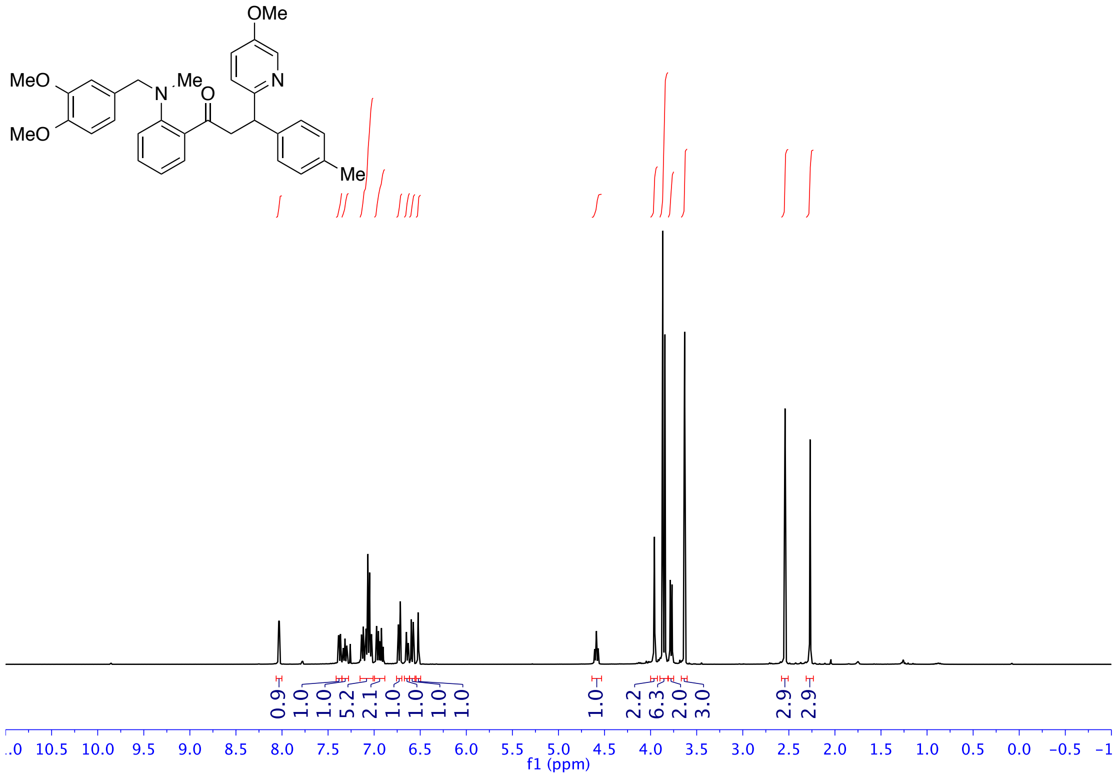

^{13}C NMR, 100 MHz, $CDCl_3$

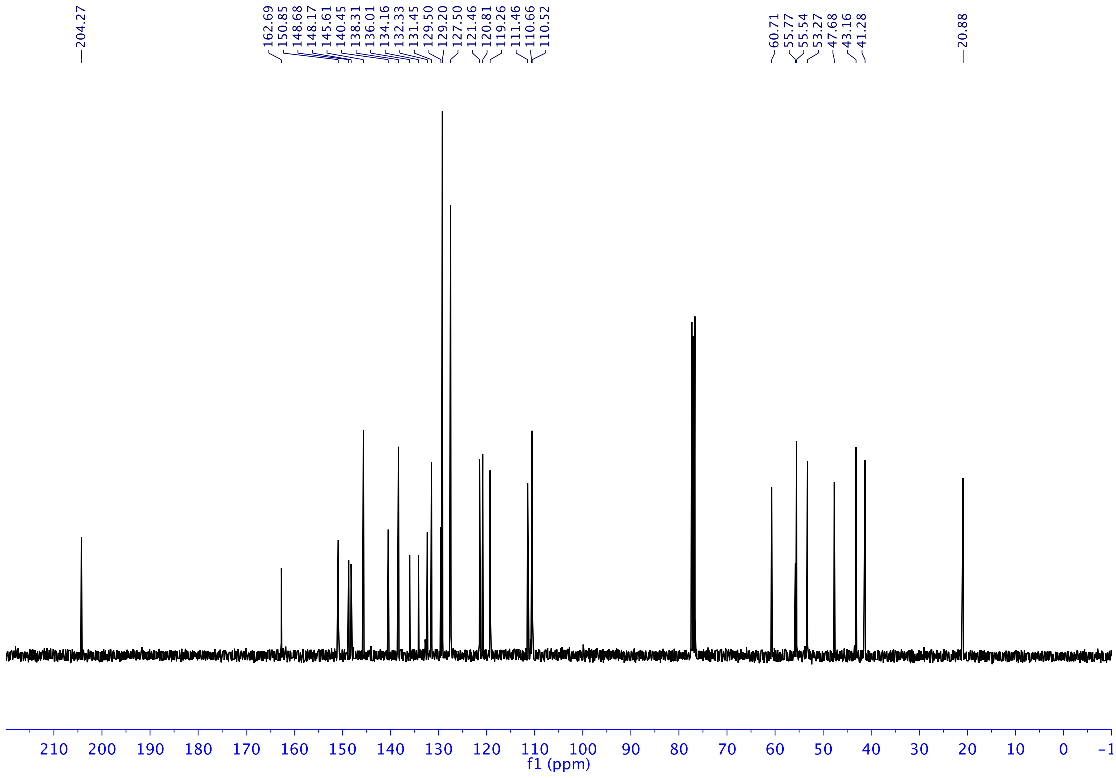

Figure 43. ^{1}H-NMR and ^{13}C-NMR spectra of compound **2ao**

^{1}H NMR, 400 MHz, $CDCl_3$

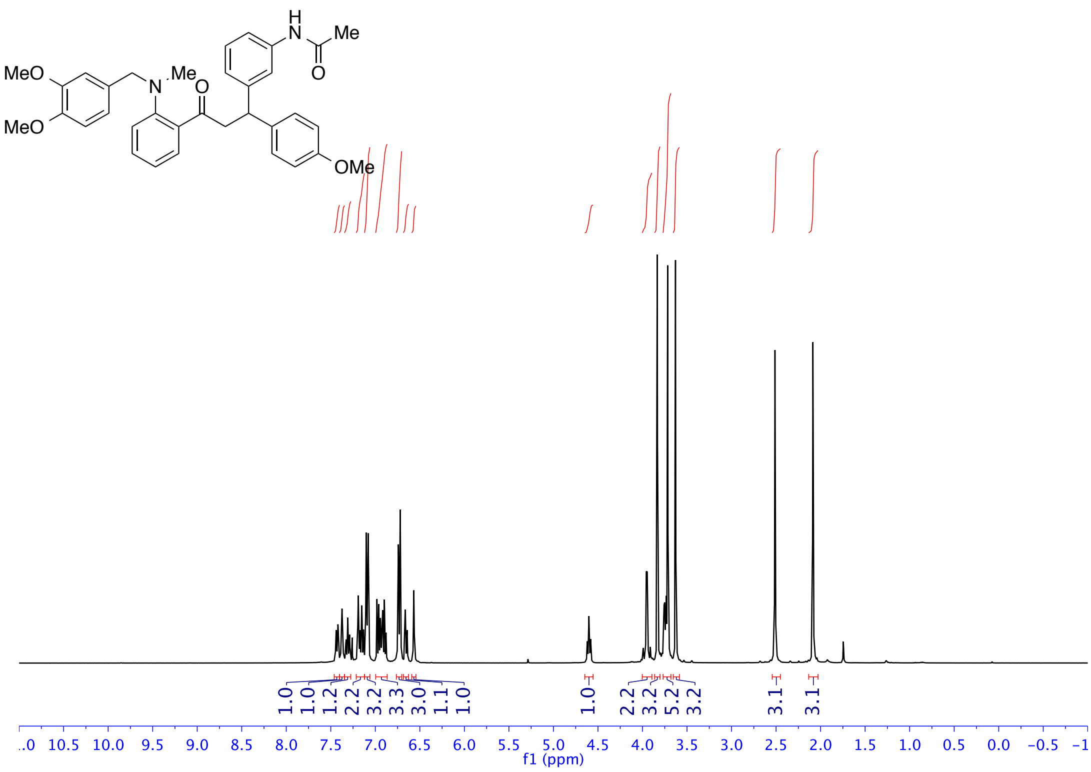

^{13}C NMR, 100 MHz, $CDCl_3$

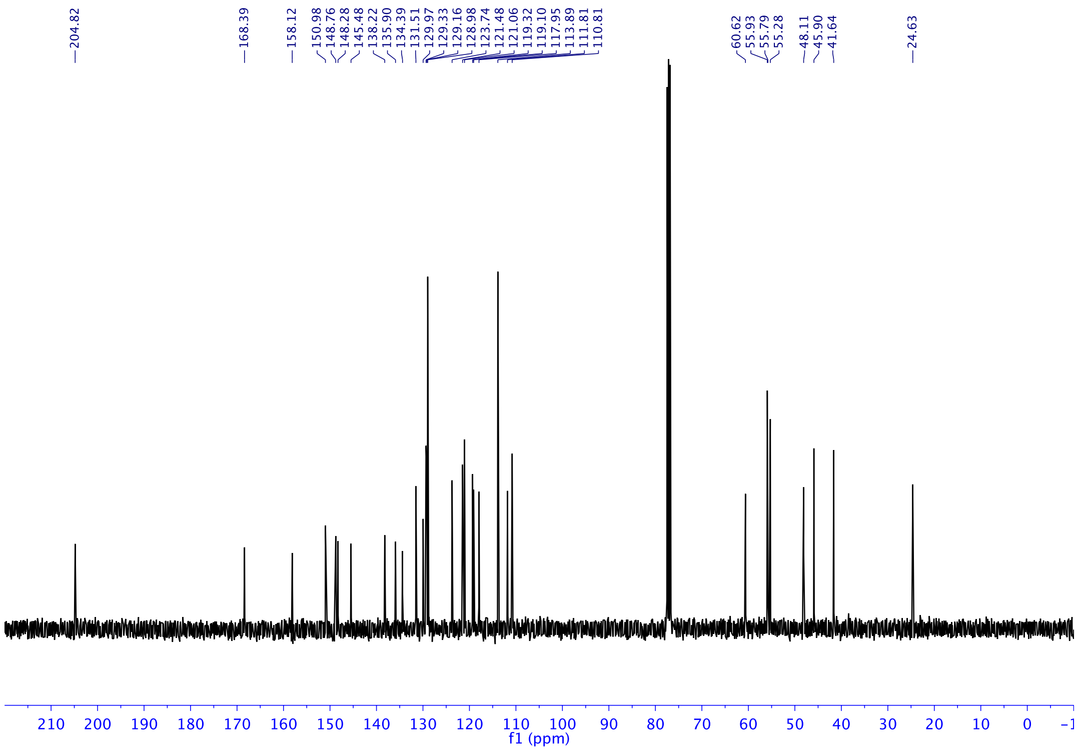

Figure 44. ^{1}H-NMR and ^{13}C-NMR spectra of compound **2ap**

^{1}H NMR, 400 MHz, $CDCl_3$

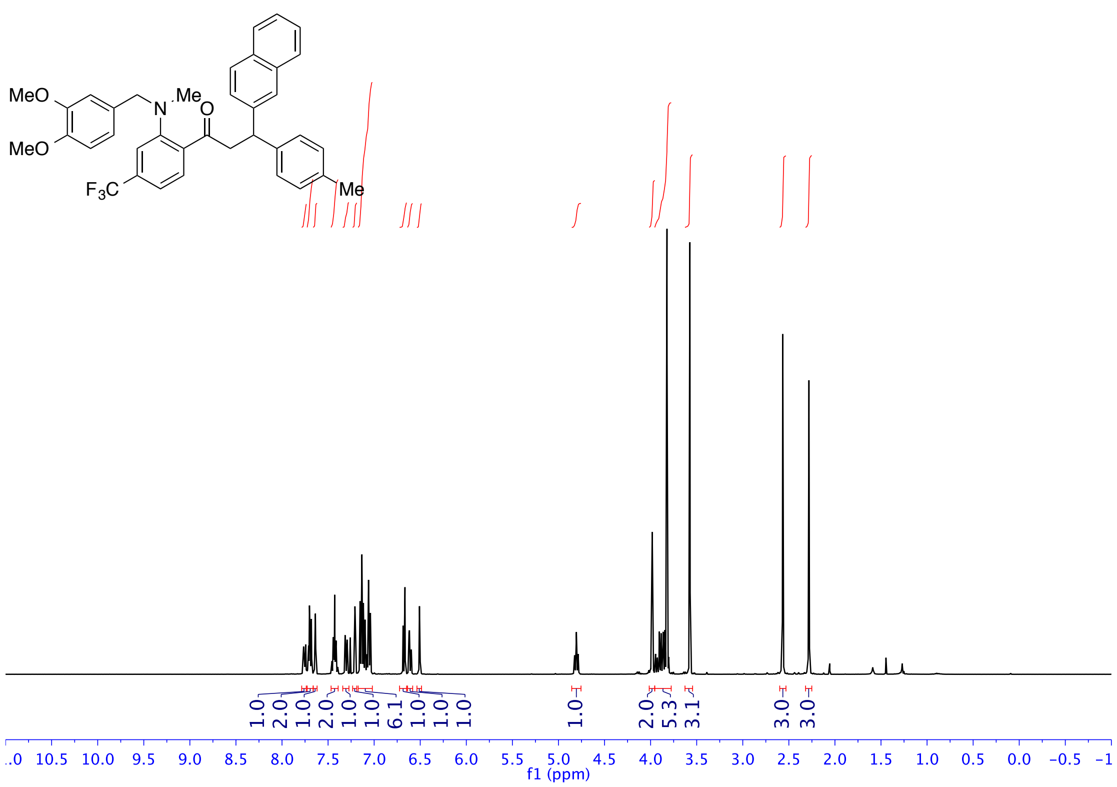

^{13}C NMR, 100 MHz, $CDCl_3$

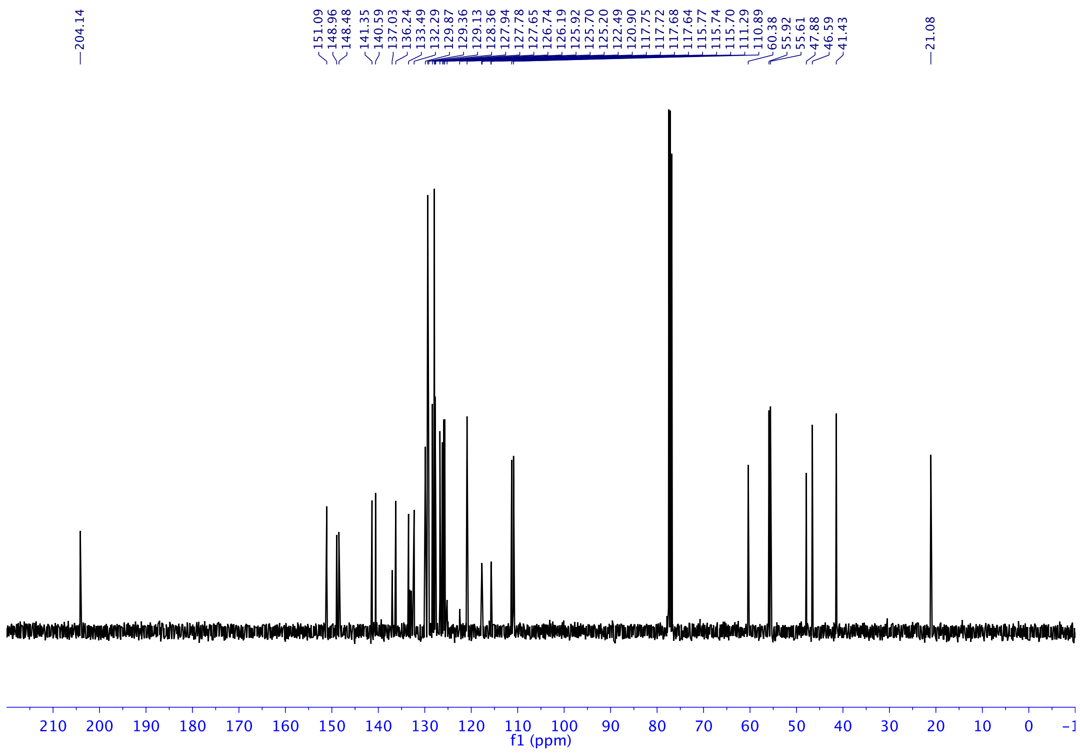

Figure 45. ^{1}H-NMR and ^{13}C-NMR spectra of compound **2aq**

^{1}H NMR, 400 MHz, $CDCl_3$

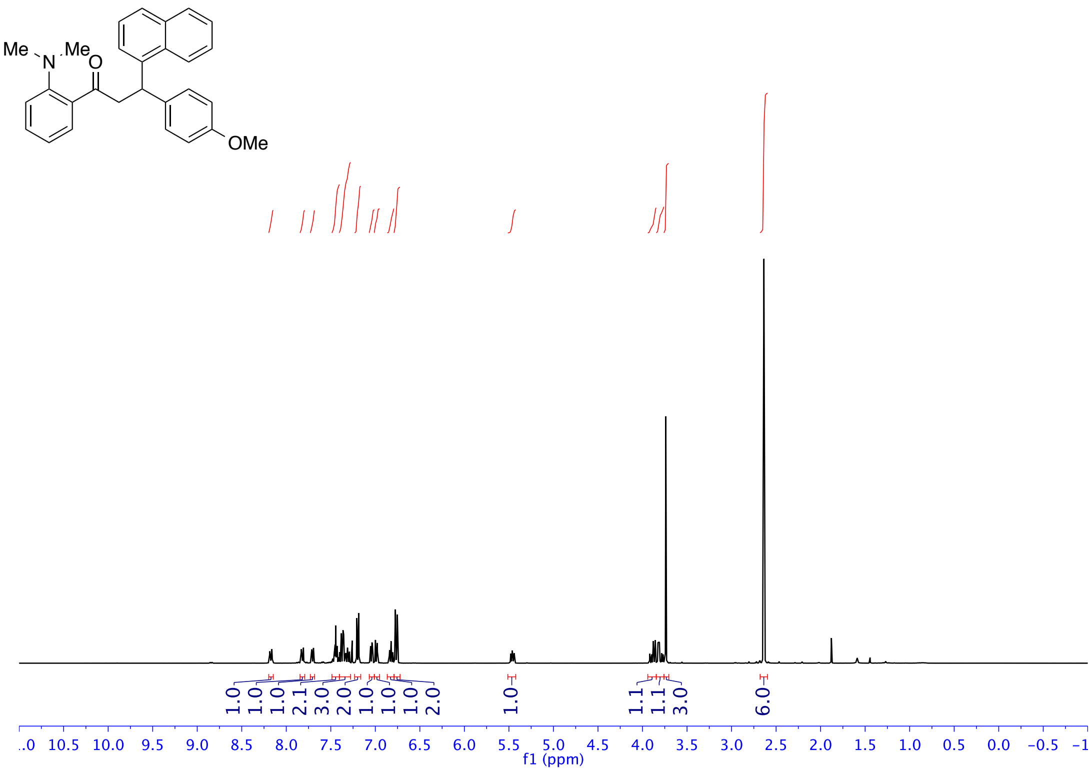

^{13}C NMR, 100 MHz, $CDCl_3$

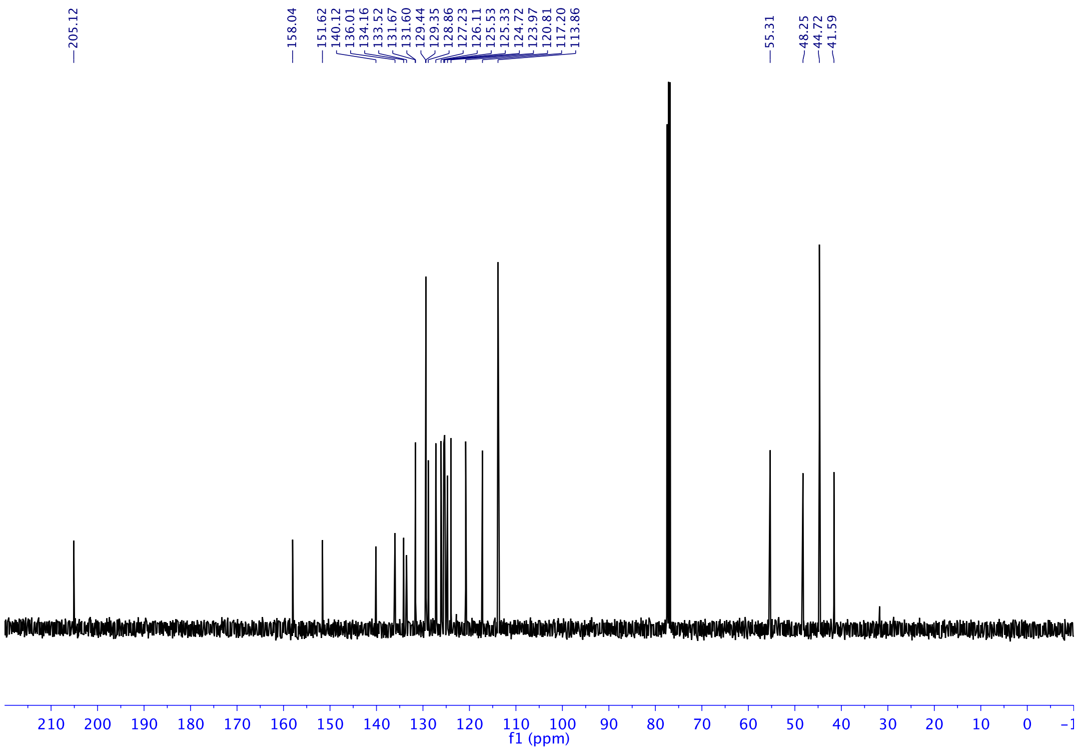

Figure 46. ^{1}H-NMR and ^{13}C-NMR spectra of compound **2ar**

^{1}H NMR, 400 MHz, $CDCl_3$

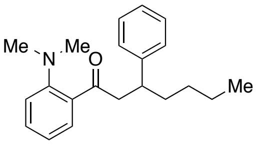

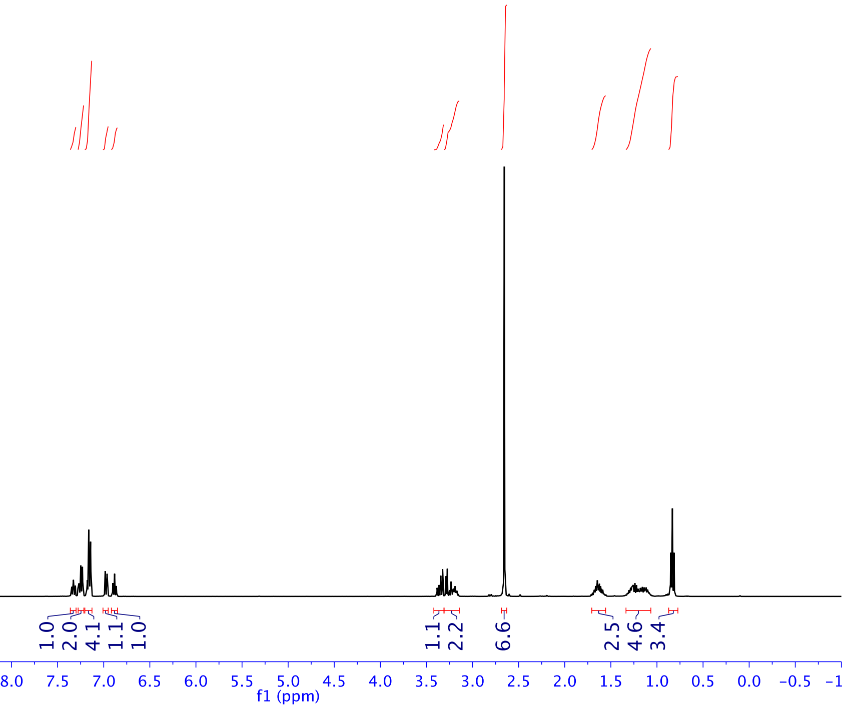

^{13}C NMR, 100 MHz, $CDCl_3$

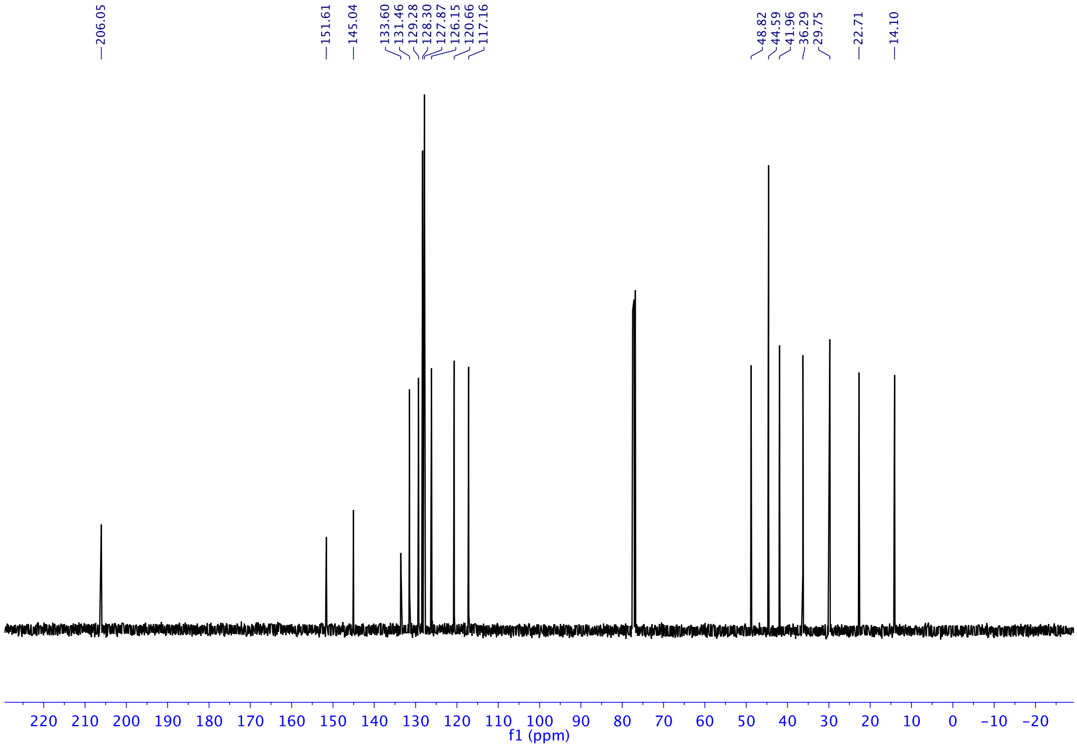

Figure 47. ^{1}H-NMR and ^{13}C-NMR spectra of compound **2as**

^{1}H NMR, 400 MHz, $CDCl_3$

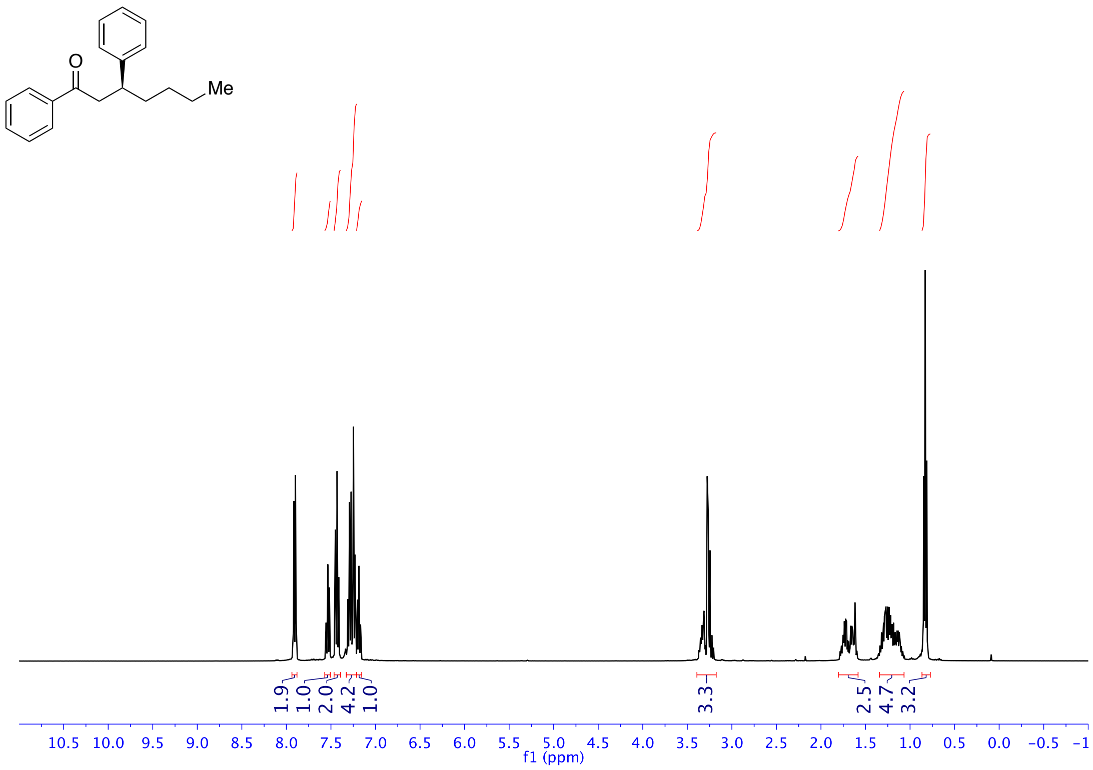

^{13}C NMR, 100 MHz, $CDCl_3$

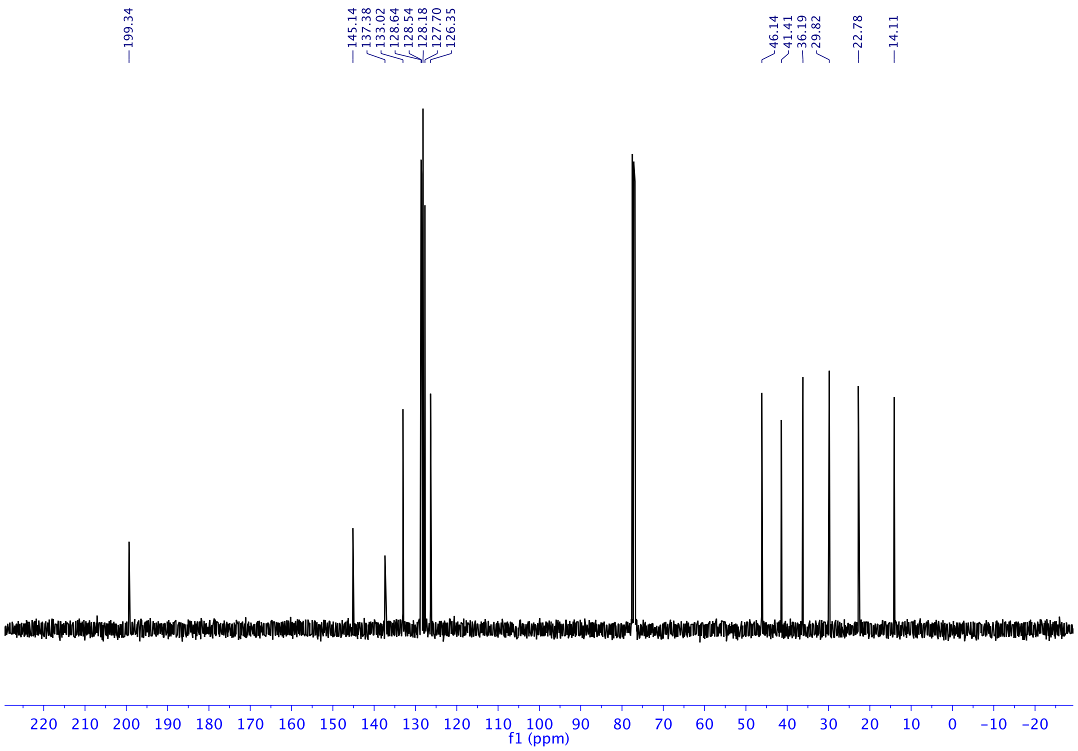

Figure 48. ^{1}H-NMR and ^{13}C-NMR spectra of compound **4as**

^{1}H NMR, 400 MHz, $CDCl_3$

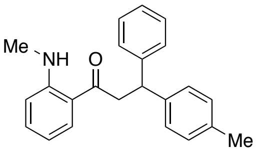

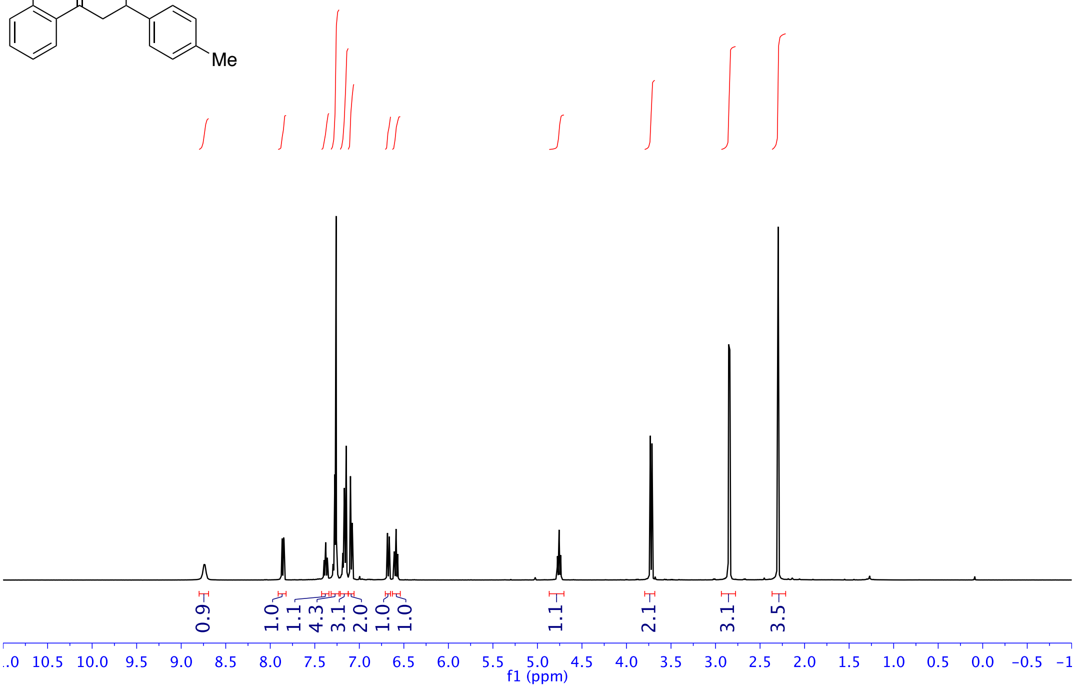

^{13}C NMR, 100 MHz, $CDCl_3$

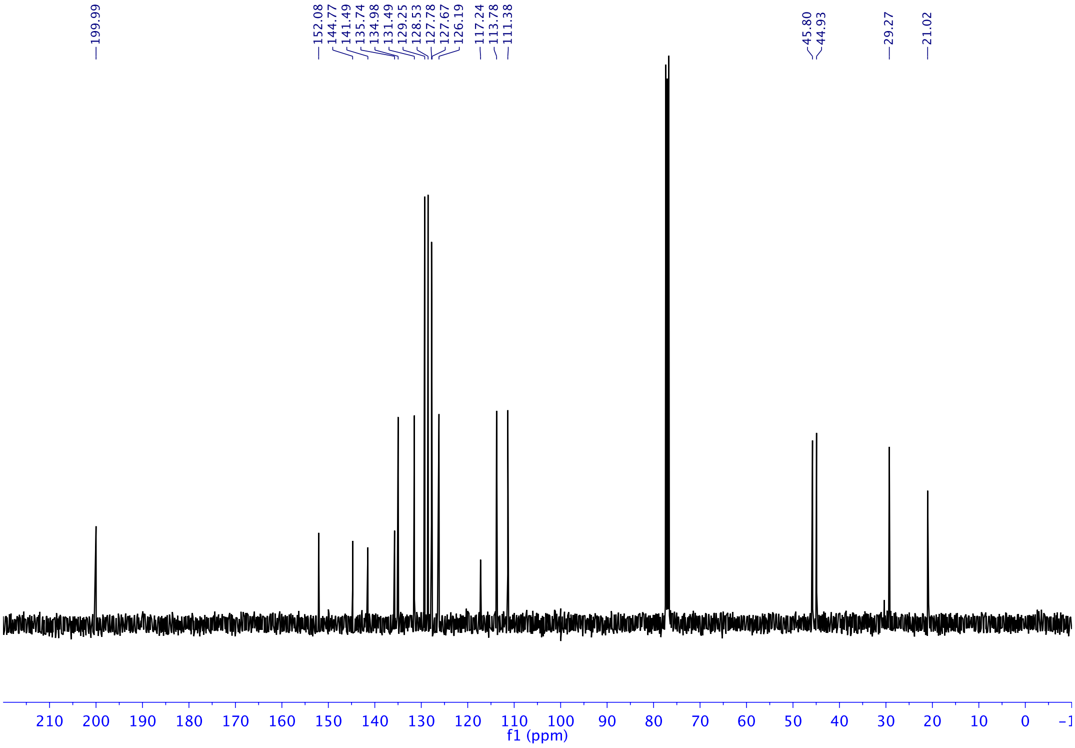

Figure 49. ^{1}H-NMR and ^{13}C-NMR spectra of compound **3ak**

^{1}H NMR, 400 MHz, $CDCl_3$

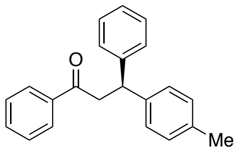

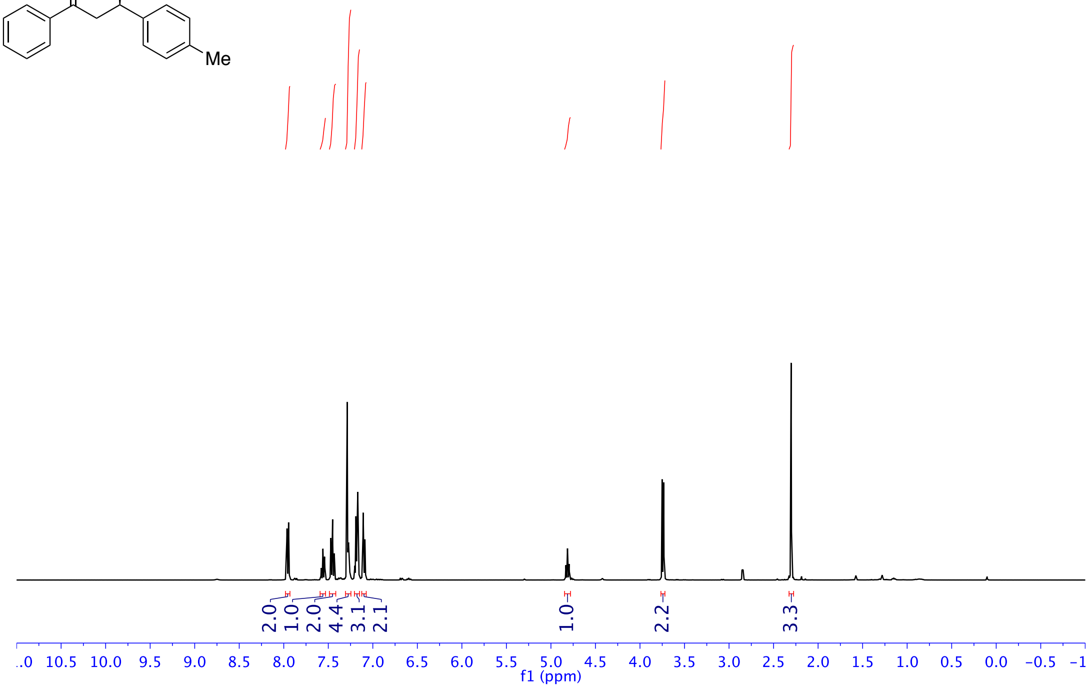

^{13}C NMR, 100 MHz, $CDCl_3$

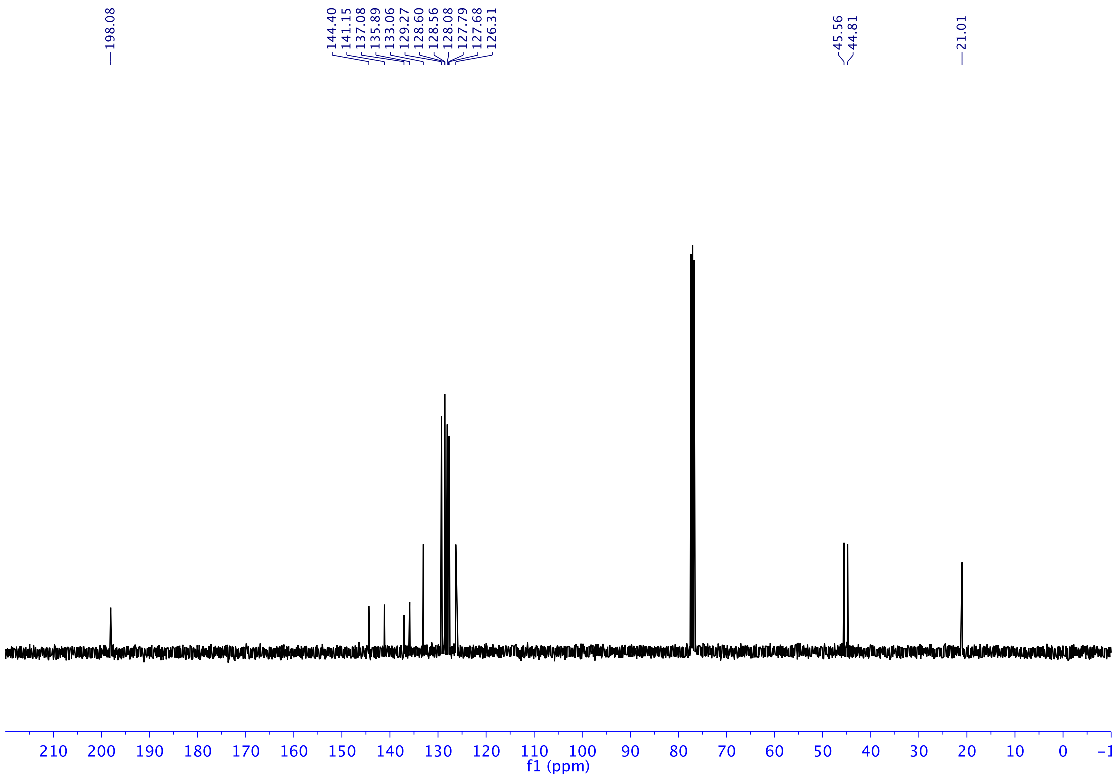

Figure 50. ^{1}H-NMR and ^{13}C-NMR spectra of compound **4ak**

HPLC traces

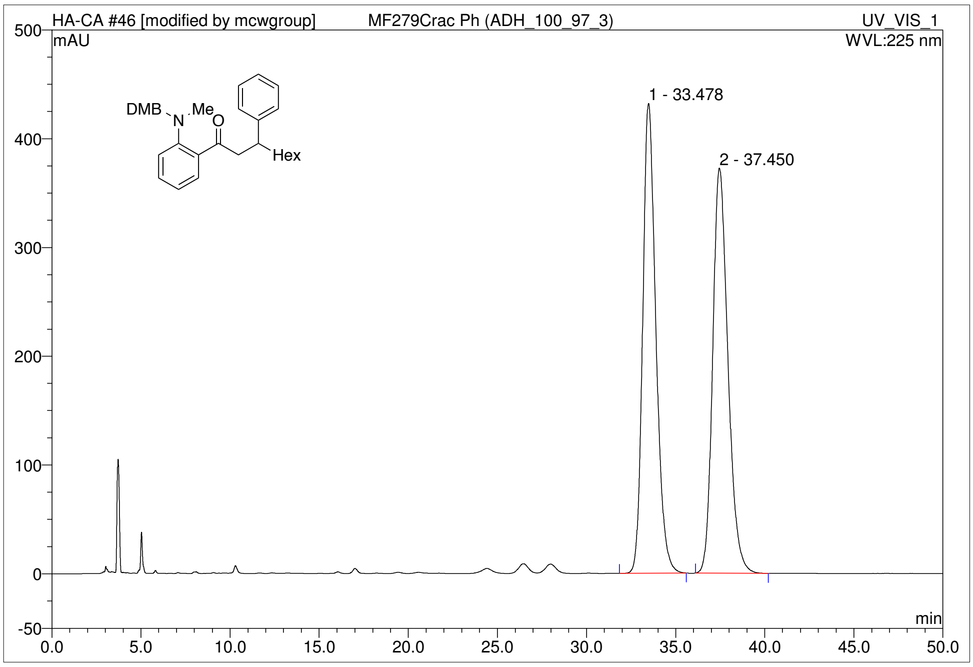

No.	Ret.Time min	Peak Name	Height mAU	Area mAU*min	Rel.Area %	Amount	Type
1	33.48	n.a.	431.928	369.409	50.07	n.a.	BMB*
2	37.45	n.a.	372.403	368.431	49.93	n.a.	BMB*
Total:			804.331	737.840	100.00	0.000	

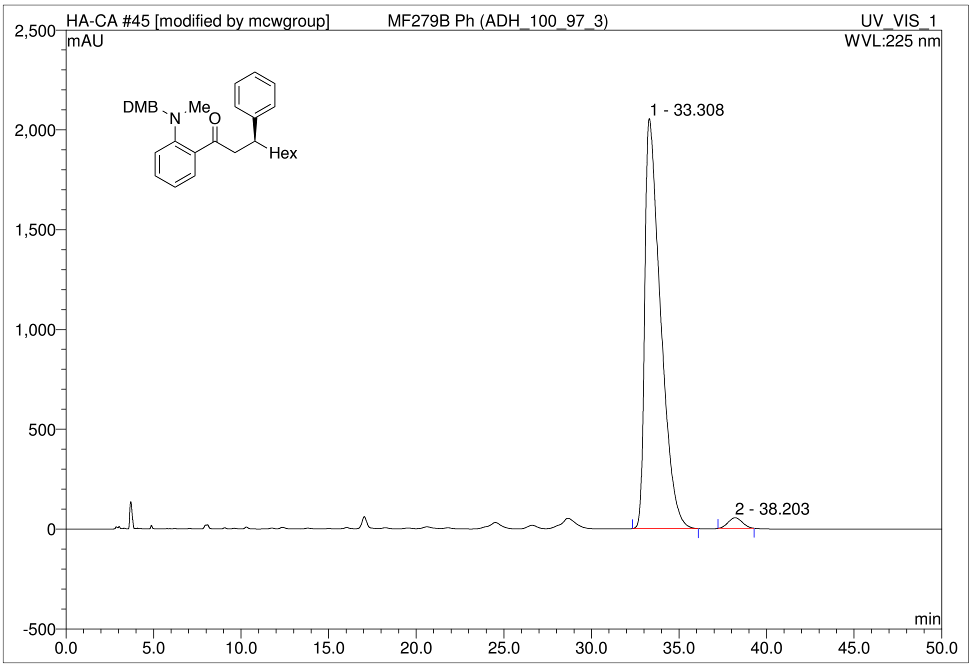

No.	Ret.Time min	Peak Name	Height mAU	Area mAU*min	Rel.Area %	Amount	Type
1	33.31	n.a.	2054.575	2148.159	97.75	n.a.	BMB*
2	38.20	n.a.	53.063	49.513	2.25	n.a.	BMB*
Total:			2107.638	2197.671	100.00	0.000	

Figure 51: HPLC chromatogram of compound **2a**

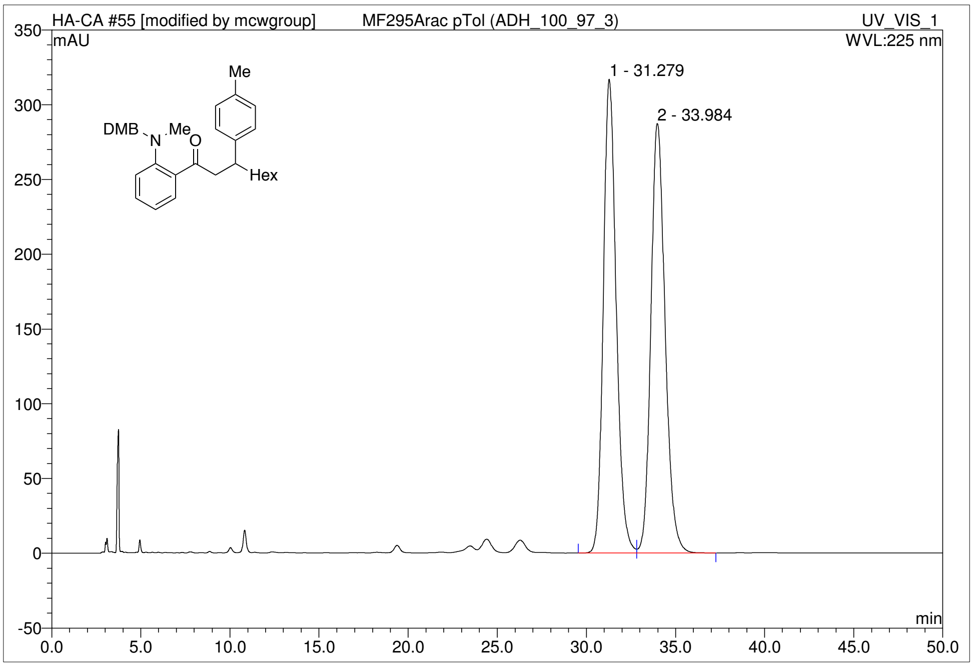

No.	Ret.Time min	Peak Name	Height mAU	Area mAU*min	Rel.Area %	Amount	Type
1	31.28	n.a.	316.857	265.954	49.94	n.a.	BM *
2	33.98	n.a.	287.247	266.548	50.06	n.a.	MB*
Total:			604.104	532.502	100.00	0.000	

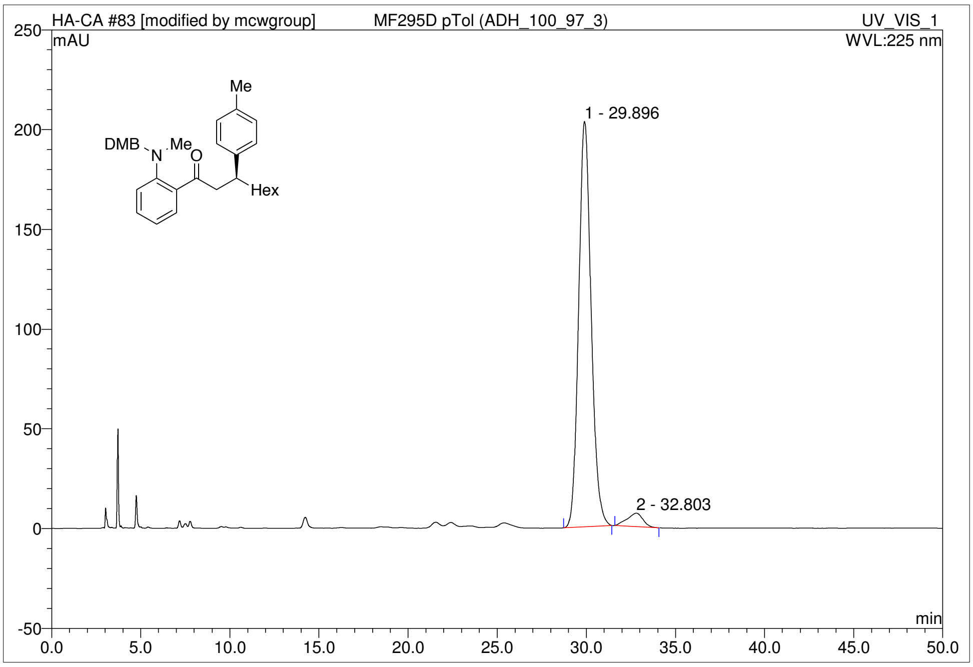

No.	Ret.Time min	Peak Name	Height mAU	Area mAU*min	Rel.Area %	Amount	Type
1	29.90	n.a.	203.216	165.049	96.04	n.a.	BMB*
2	32.80	n.a.	6.702	6.812	3.96	n.a.	BMB*
Total:			209.918	171.861	100.00	0.000	

Figure 52: HPLC chromatogram of compound **2b**

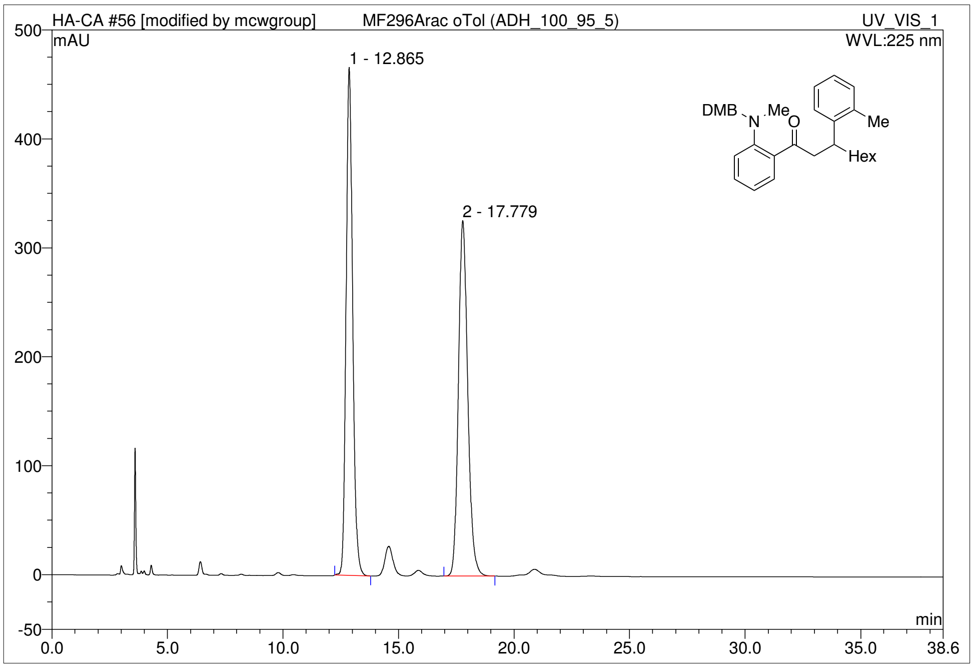

No.	Ret.Time min	Peak Name	Height mAU	Area mAU*min	Rel.Area %	Amount	Type
1	12.87	n.a.	466.276	152.981	50.03	n.a.	BMB*
2	17.78	n.a.	326.291	152.806	49.97	n.a.	BMB*
Total:			792.566	305.787	100.00	0.000	

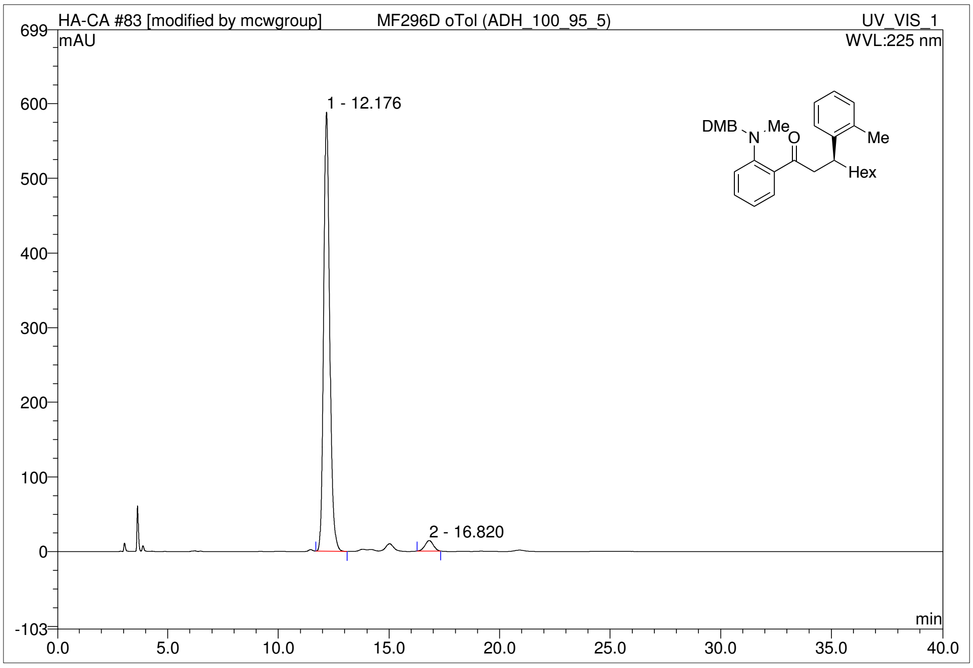

No.	Ret.Time min	Peak Name	Height mAU	Area mAU*min	Rel.Area %	Amount	Type
1	12.18	n.a.	588.022	185.245	96.79	n.a.	BMB*
2	16.82	n.a.	13.941	6.150	3.21	n.a.	BMB*
Total:			601.963	191.395	100.00	0.000	

Figure 53: HPLC chromatogram of compound **2c**

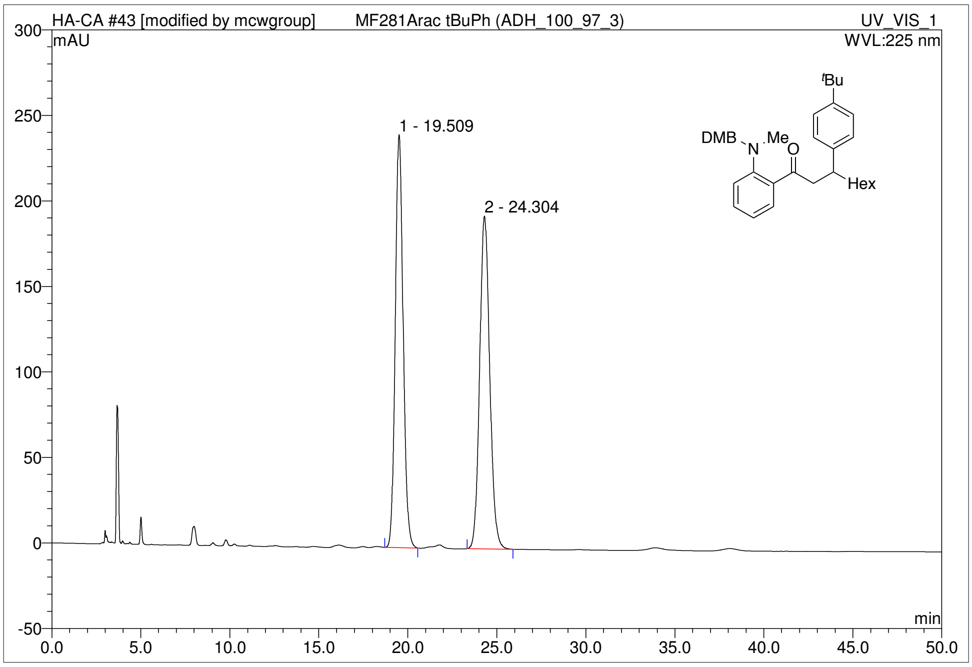

No.	Ret.Time min	Peak Name	Height mAU	Area mAU*min	Rel.Area %	Amount	Type
1	19.51	n.a.	241.554	124.086	49.24	n.a.	BMB*
2	24.30	n.a.	194.620	127.940	50.76	n.a.	BMB*
Total:			436.175	252.026	100.00	0.000	

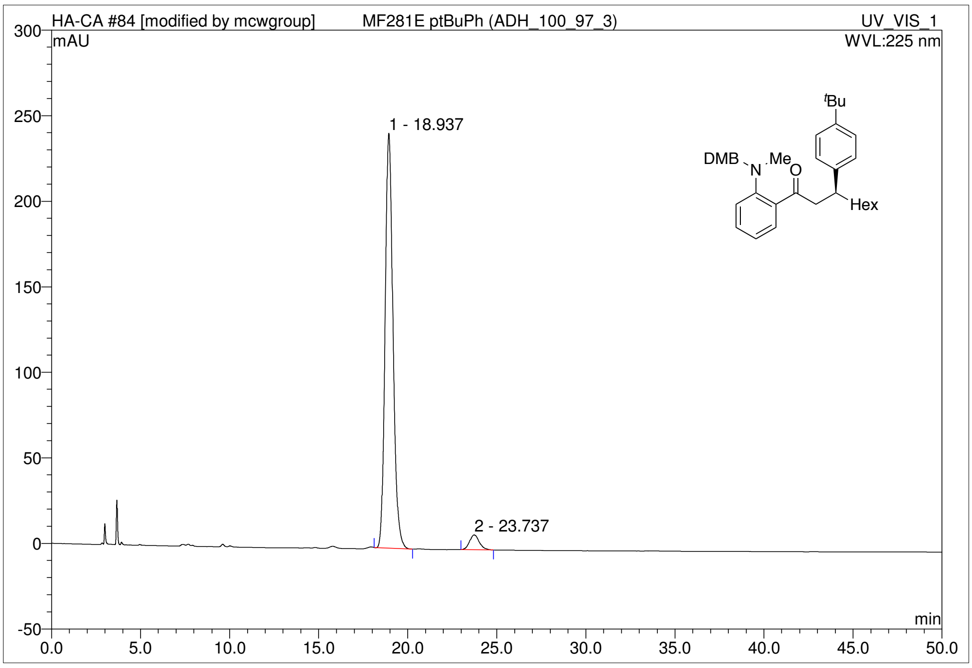

No.	Ret.Time min	Peak Name	Height mAU	Area mAU*min	Rel.Area %	Amount	Type
1	18.94	n.a.	242.523	124.115	95.75	n.a.	BMB*
2	23.74	n.a.	8.697	5.510	4.25	n.a.	BMB*
Total:			251.220	129.625	100.00	0.000	

Figure 54: HPLC chromatogram of compound **2d**

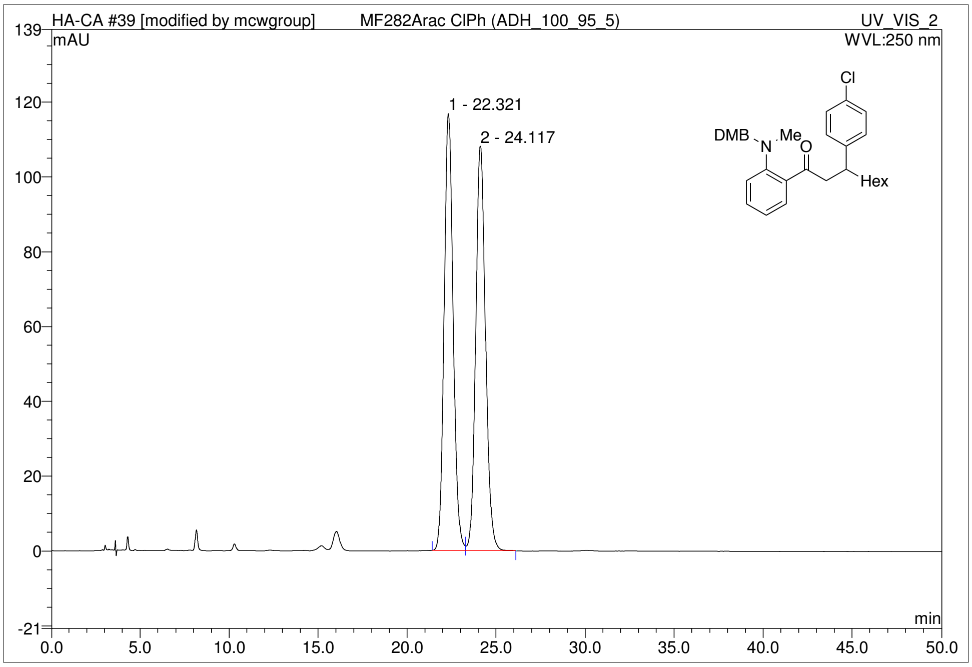

No.	Ret.Time min	Peak Name	Height mAU	Area mAU*min	Rel.Area %	Amount	Type
1	22.32	n.a.	116.806	68.661	49.86	n.a.	BM *
2	24.12	n.a.	108.143	69.042	50.14	n.a.	MB*
Total:			224.949	137.702	100.00	0.000	

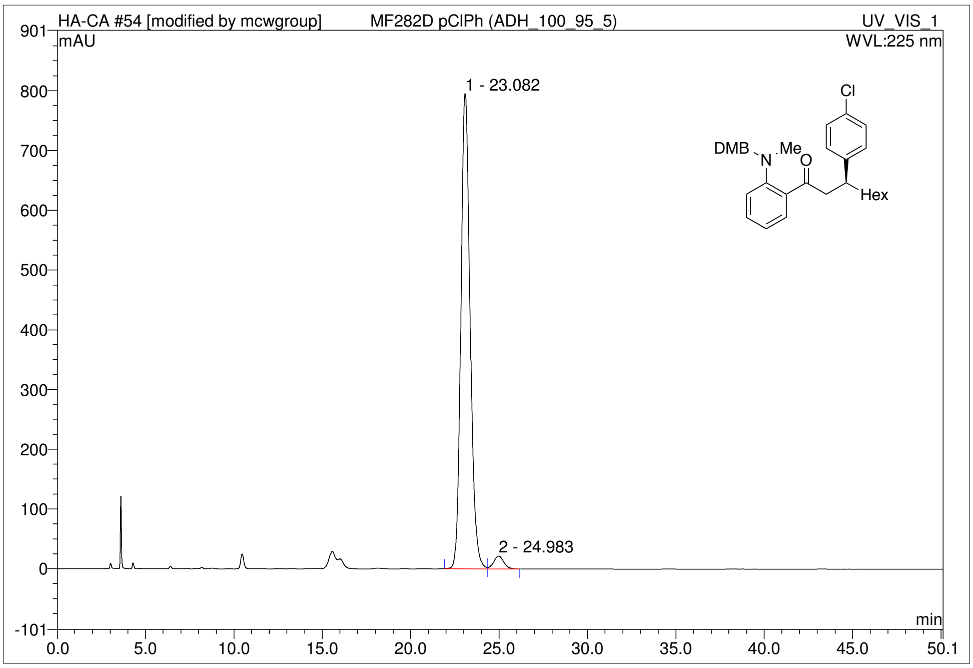

No.	Ret.Time min	Peak Name	Height mAU	Area mAU*min	Rel.Area %	Amount	Type
1	23.08	n.a.	795.227	482.165	97.14	n.a.	BM *
2	24.98	n.a.	21.543	14.215	2.86	n.a.	MB*
Total:			816.771	496.379	100.00	0.000	

Figure 55: HPLC chromatogram of compound **2e**

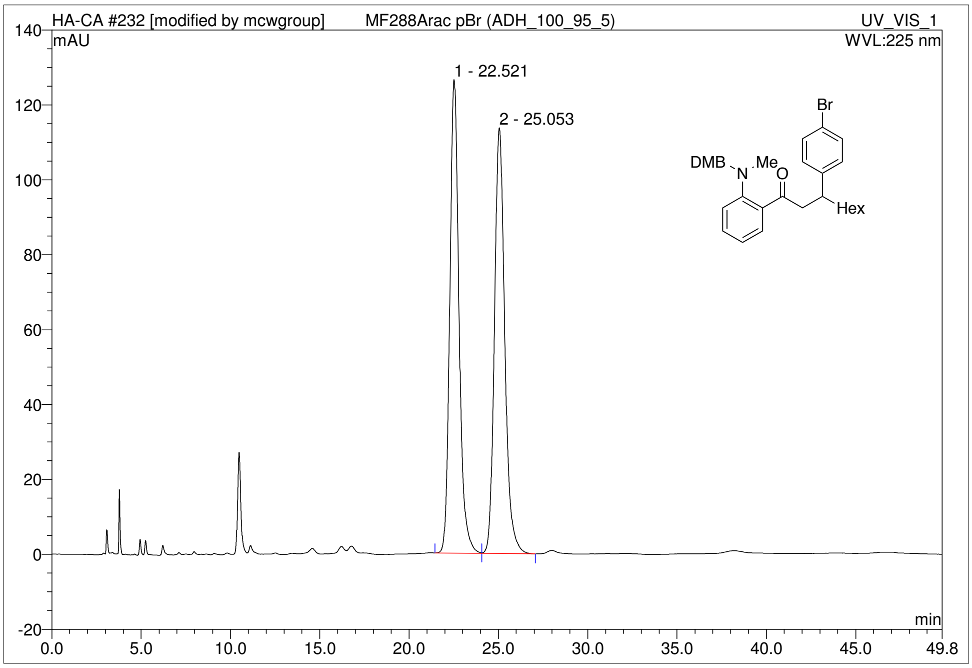

No.	Ret.Time min	Peak Name	Height mAU	Area mAU*min	Rel.Area %	Amount	Type
1	22.52	n.a.	126.399	75.761	49.97	n.a.	BM *
2	25.05	n.a.	113.646	75.853	50.03	n.a.	MB*
Total:			240.045	151.614	100.00	0.000	

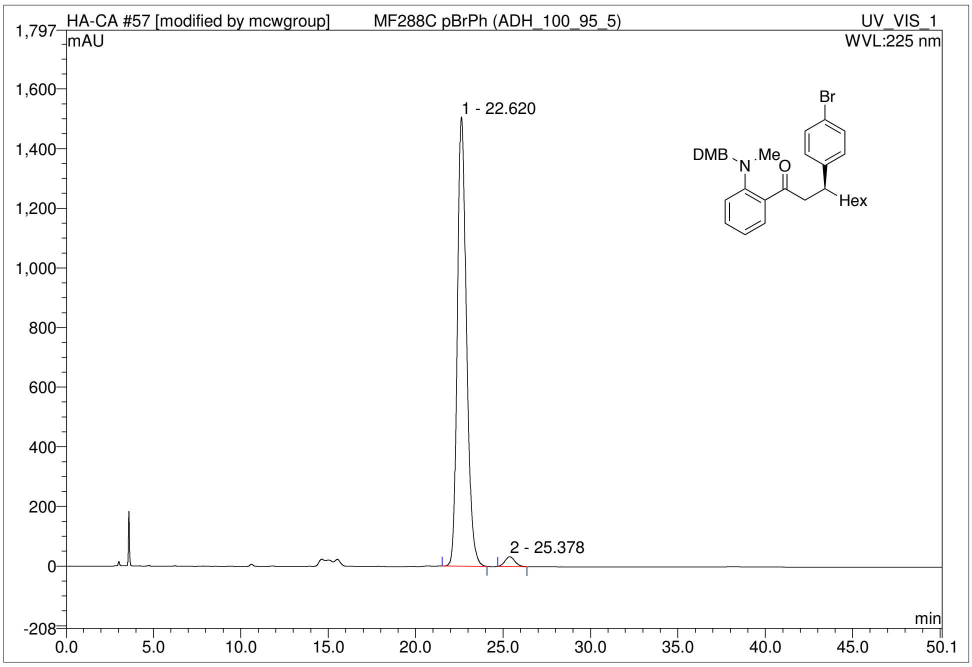

No.	Ret.Time min	Peak Name	Height mAU	Area mAU*min	Rel.Area %	Amount	Type
1	22.62	n.a.	1505.855	915.263	97.71	n.a.	BMB*
2	25.38	n.a.	32.891	21.405	2.29	n.a.	BMB*
Total:			1538.746	936.668	100.00	0.000	

Figure 56: HPLC chromatogram of compound **2f**

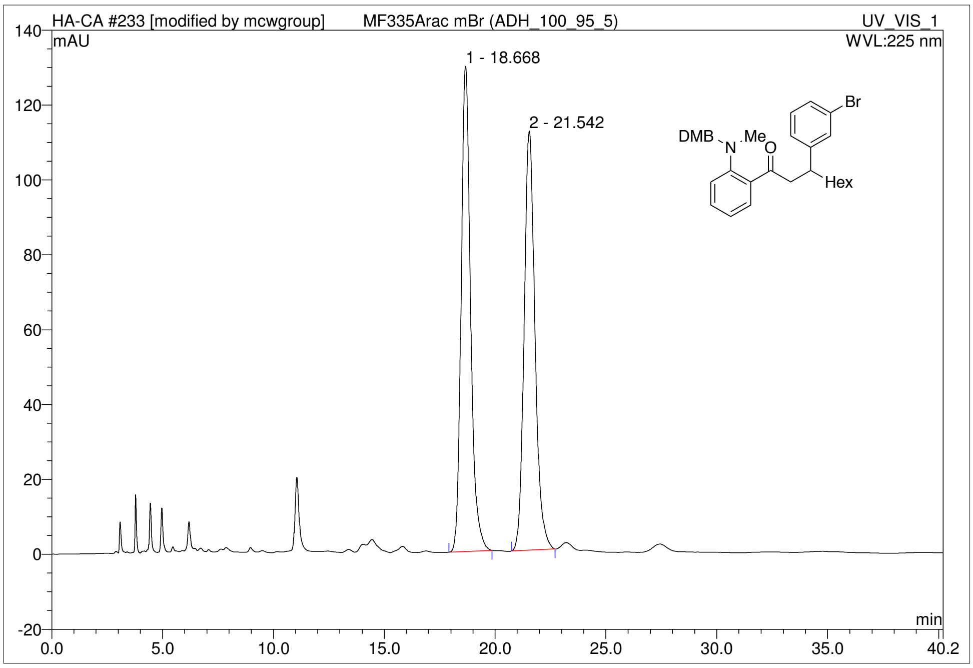

No.	Ret.Time min	Peak Name	Height mAU	Area mAU*min	Rel.Area %	Amount	Type
1	18.67	n.a.	129.535	63.952	50.19	n.a.	BMB*
2	21.54	n.a.	111.915	63.467	49.81	n.a.	BMB*
Total:			241.449	127.419	100.00	0.000	

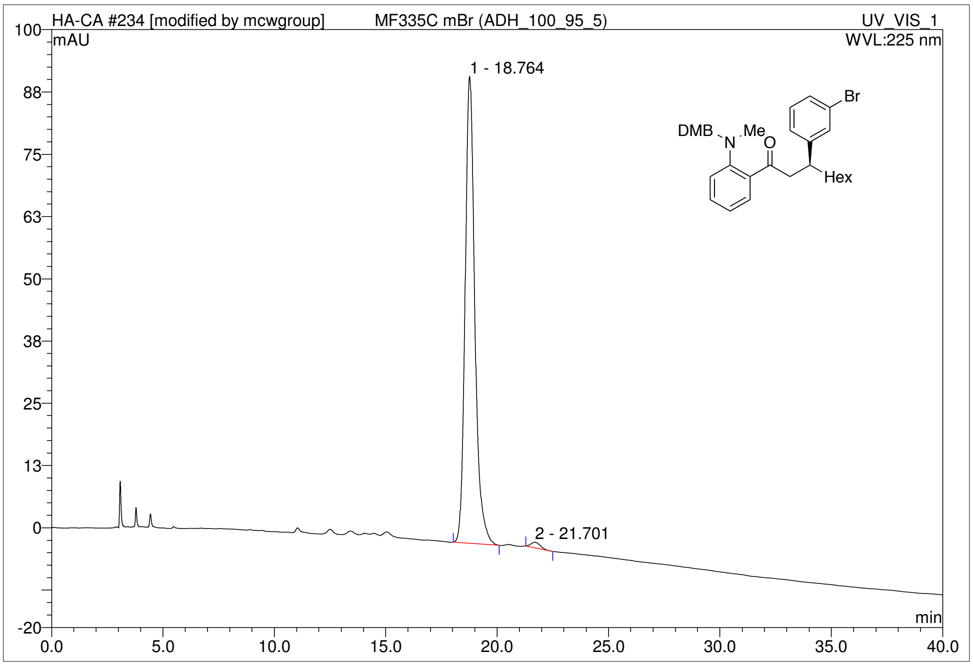

No.	Ret.Time min	Peak Name	Height mAU	Area mAU*min	Rel.Area %	Amount	Type
1	18.76	n.a.	93.719	47.261	98.89	n.a.	BMB*
2	21.70	n.a.	1.117	0.528	1.11	n.a.	BMB*
Total:			94.835	47.790	100.00	0.000	

Figure 57: HPLC chromatogram of compound **2g**

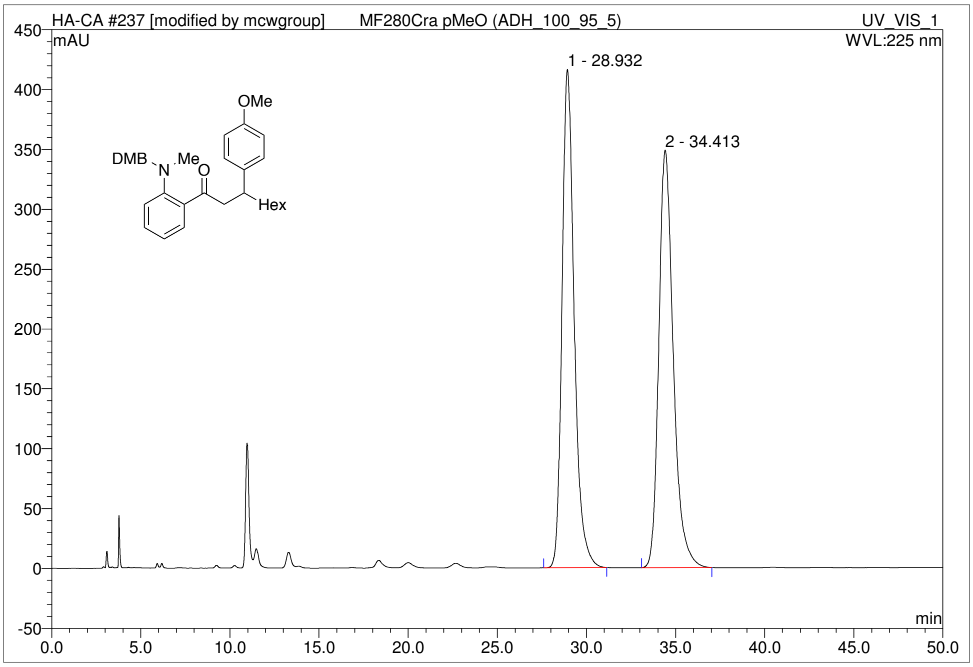

No.	Ret.Time min	Peak Name	Height mAU	Area mAU*min	Rel.Area %	Amount	Type
1	28.93	n.a.	416.325	328.771	50.02	n.a.	BMB*
2	34.41	n.a.	348.816	328.491	49.98	n.a.	BMB*
Total:			765.141	657.261	100.00	0.000	

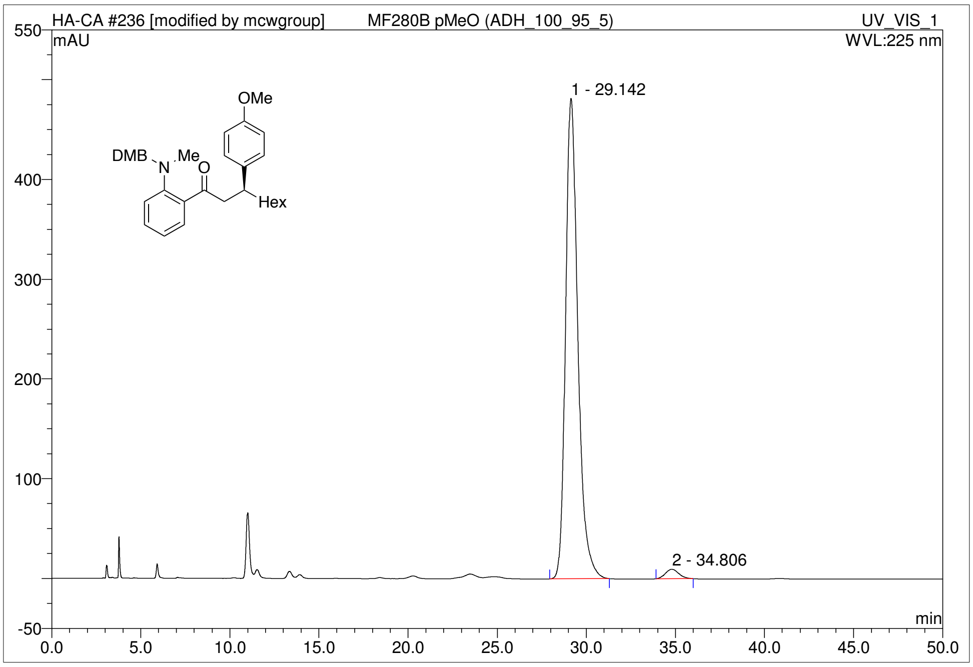

No.	Ret.Time min	Peak Name	Height mAU	Area mAU*min	Rel.Area %	Amount	Type
1	29.14	n.a.	481.674	385.005	97.92	n.a.	BMB*
2	34.81	n.a.	9.432	8.191	2.08	n.a.	BMB*
Total:			491.106	393.196	100.00	0.000	

Figure 58: HPLC chromatogram of compound **2h**

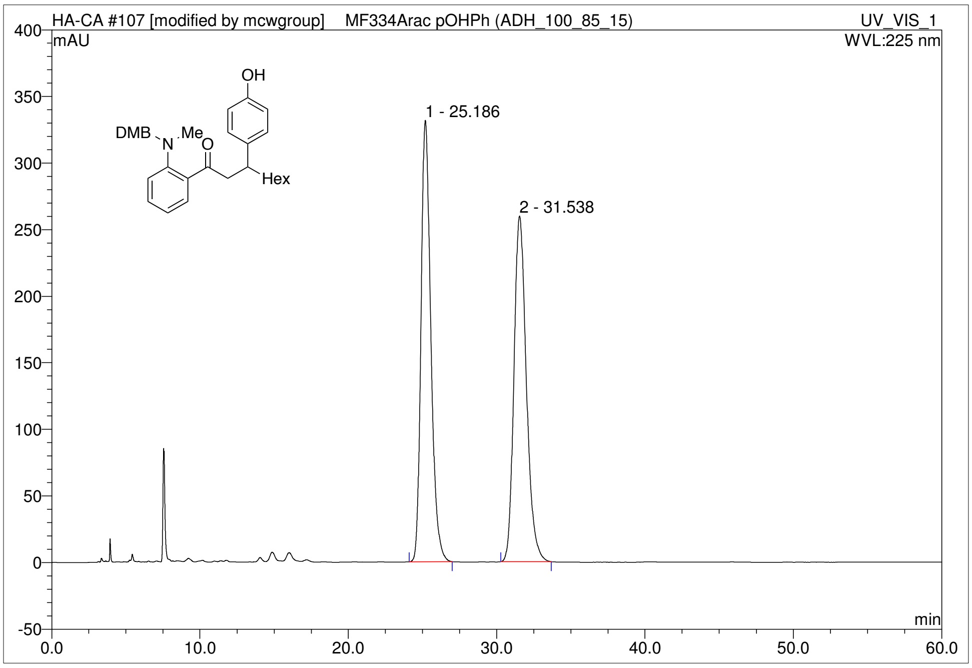

No.	Ret.Time min	Peak Name	Height mAU	Area mAU*min	Rel.Area %	Amount	Type
1	25.19	n.a.	331.525	253.686	50.19	n.a.	BMB*
2	31.54	n.a.	259.628	251.799	49.81	n.a.	BMB*
Total:			591.152	505.485	100.00	0.000	

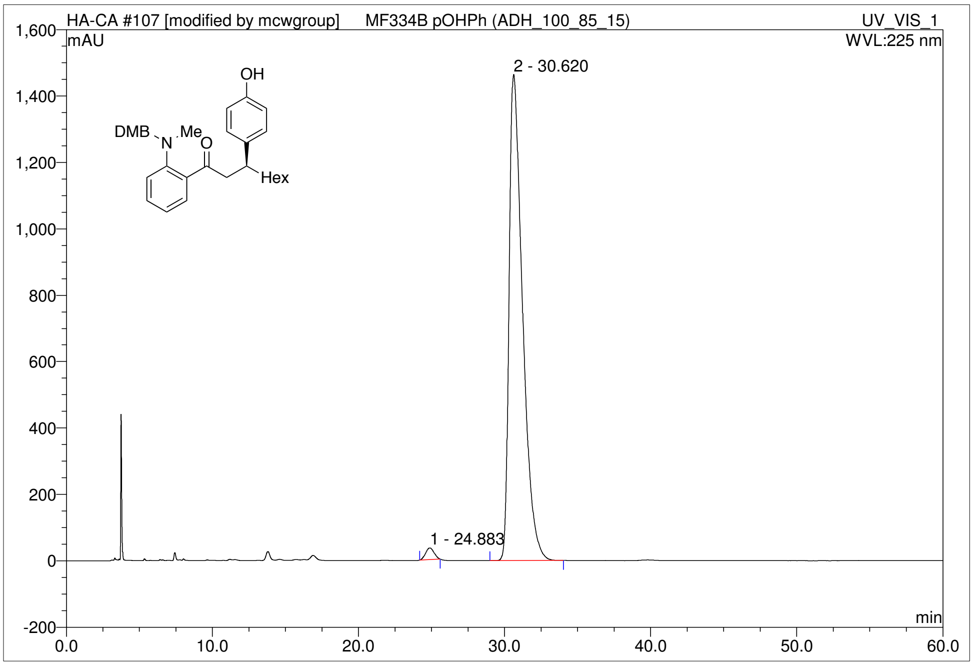

No.	Ret.Time min	Peak Name	Height mAU	Area mAU*min	Rel.Area %	Amount	Type
1	24.88	n.a.	35.081	23.643	1.50	n.a.	BMB*
2	30.62	n.a.	1463.812	1556.962	98.50	n.a.	BMB*
Total:			1498.893	1580.605	100.00	0.000	

Figure 59: HPLC chromatogram of compound **2i**

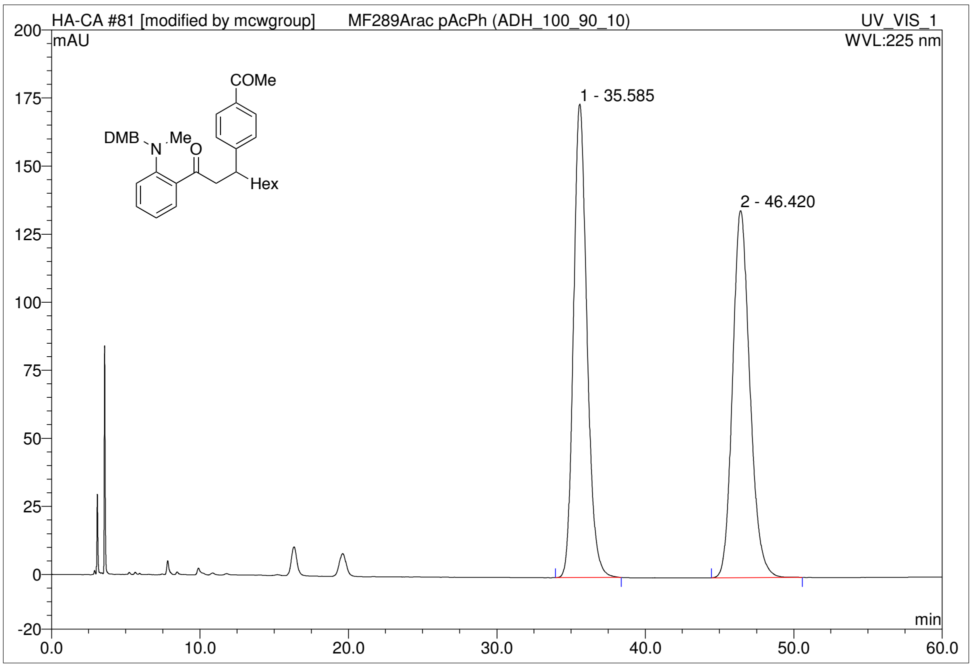

No.	Ret.Time min	Peak Name	Height mAU	Area mAU*min	Rel.Area %	Amount	Type
1	35.59	n.a.	173.873	182.294	50.05	n.a.	BMB*
2	46.42	n.a.	134.760	181.915	49.95	n.a.	BMB*
Total:			308.633	364.209	100.00	0.000	

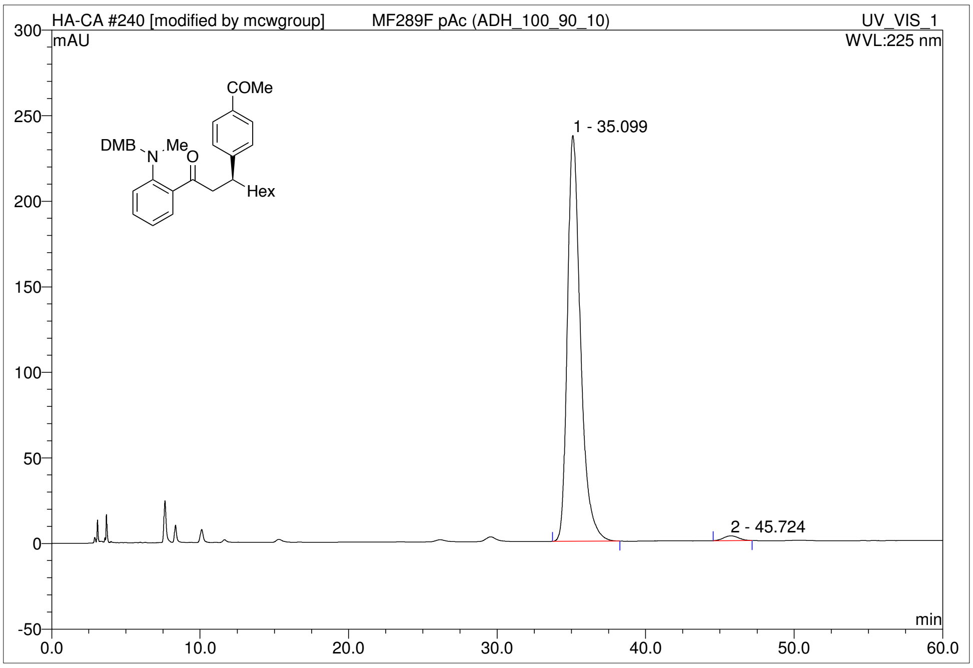

No.	Ret.Time min	Peak Name	Height mAU	Area mAU*min	Rel.Area %	Amount	Type
1	35.10	n.a.	237.178	241.979	98.66	n.a.	BMB*
2	45.72	n.a.	2.813	3.293	1.34	n.a.	BMB*
Total:			239.992	245.272	100.00	0.000	

Figure 60: HPLC chromatogram of compound **2j**

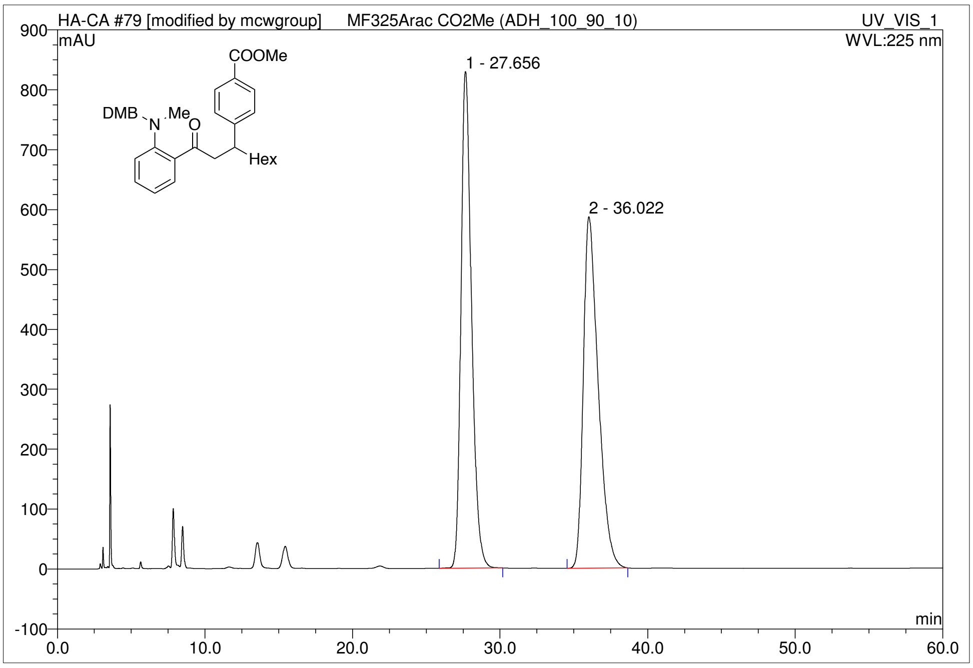

No.	Ret.Time min	Peak Name	Height mAU	Area mAU*min	Rel.Area %	Amount	Type
1	27.66	n.a.	829.063	686.924	50.02	n.a.	BMB*
2	36.02	n.a.	586.921	686.443	49.98	n.a.	BMB*
Total:			1415.984	1373.368	100.00	0.000	

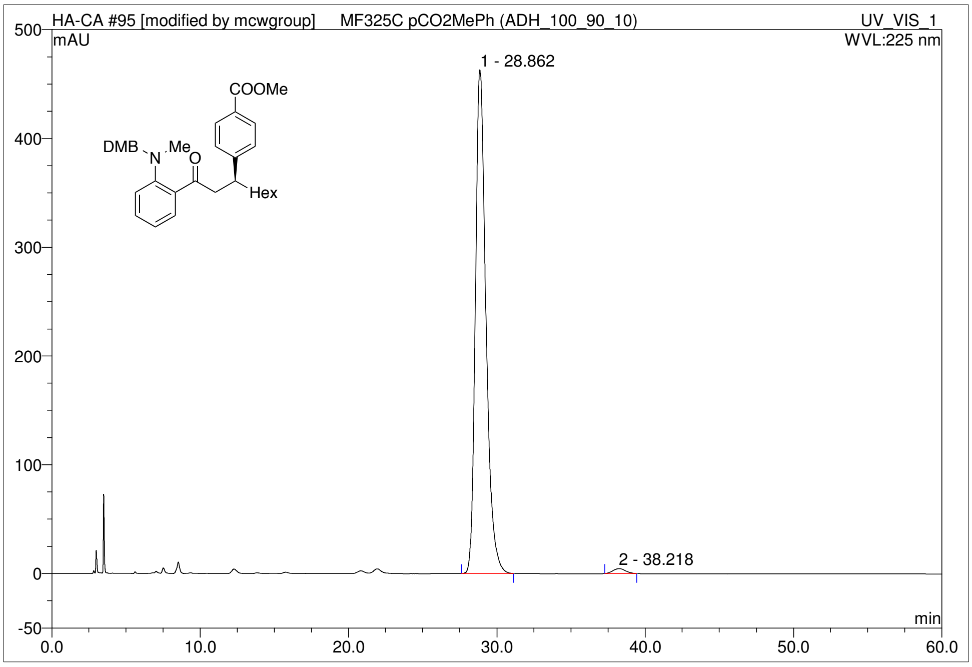

No.	Ret.Time min	Peak Name	Height mAU	Area mAU*min	Rel.Area %	Amount	Type
1	28.86	n.a.	463.183	382.554	98.86	n.a.	BMB*
2	38.22	n.a.	4.523	4.428	1.14	n.a.	BMB*
Total:			467.706	386.982	100.00	0.000	

Figure 61: HPLC chromatogram of compound **2k**

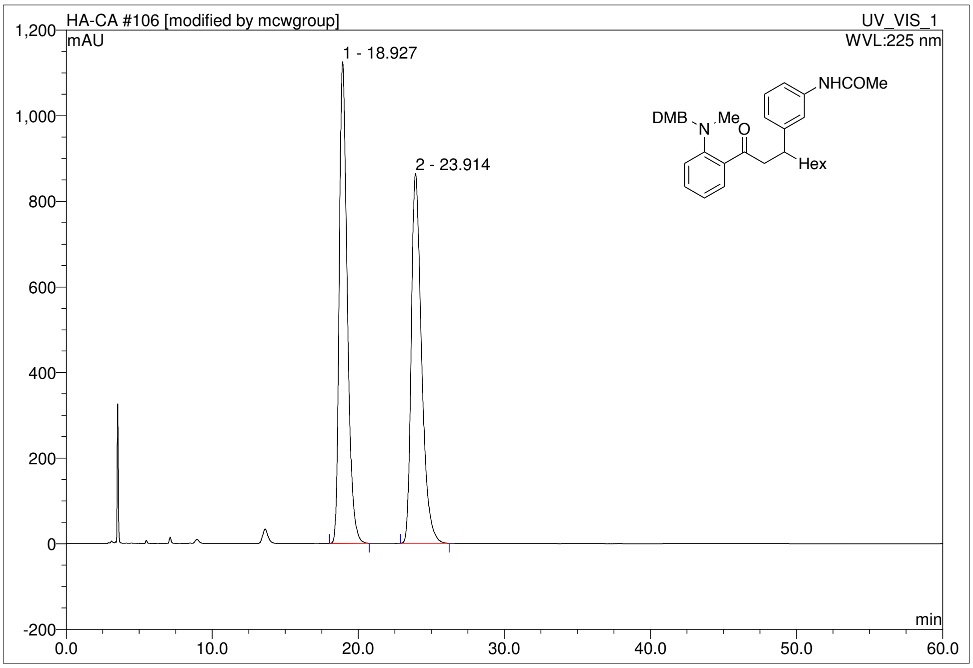

No.	Ret.Time min	Peak Name	Height mAU	Area mAU*min	Rel.Area %	Amount	Type
1	18.93	n.a.	1125.159	718.270	50.41	n.a.	BMB*
2	23.91	n.a.	864.286	706.684	49.59	n.a.	BMB*
Total:			1989.445	1424.954	100.00	0.000	

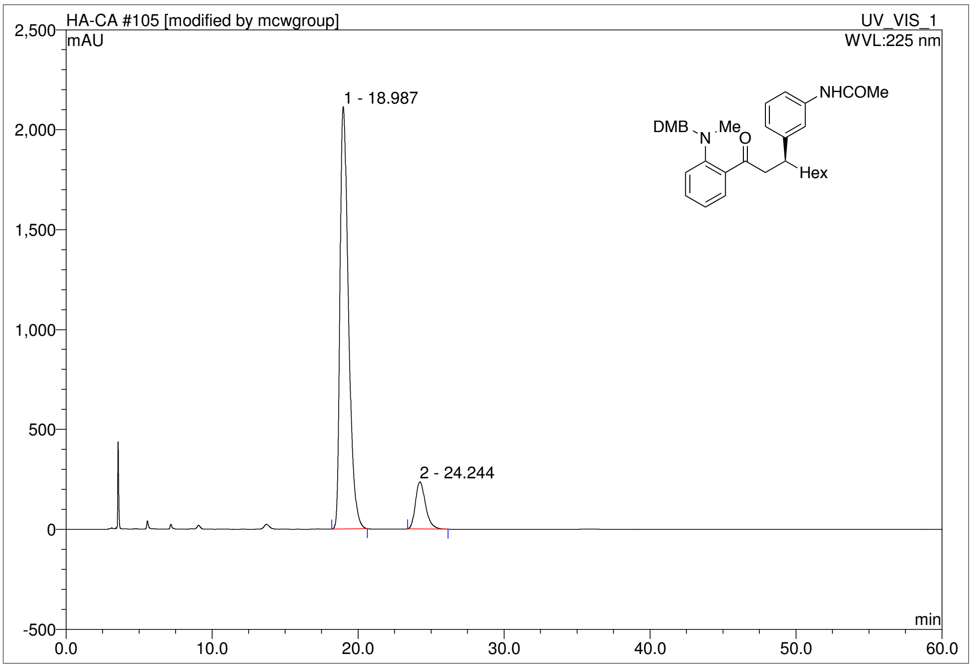

No.	Ret.Time min	Peak Name	Height mAU	Area mAU*min	Rel.Area %	Amount	Type
1	18.99	n.a.	2113.707	1425.873	88.36	n.a.	BMB*
2	24.24	n.a.	235.369	187.888	11.64	n.a.	BMB*
Total:			2349.076	1613.761	100.00	0.000	

Figure 62: HPLC chromatogram of compound **2n**

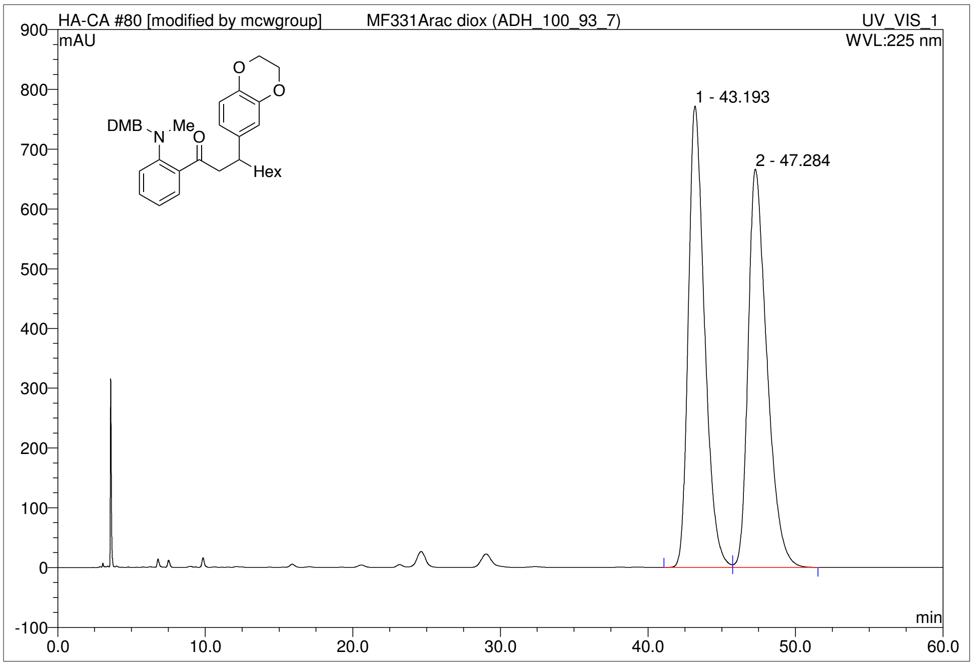

No.	Ret.Time min	Peak Name	Height mAU	Area mAU*min	Rel.Area %	Amount	Type
1	43.19	n.a.	772.004	983.259	49.96	n.a.	BM
2	47.28	n.a.	666.482	984.990	50.04	n.a.	MB
Total:			1438.486	1968.249	100.00	0.000	

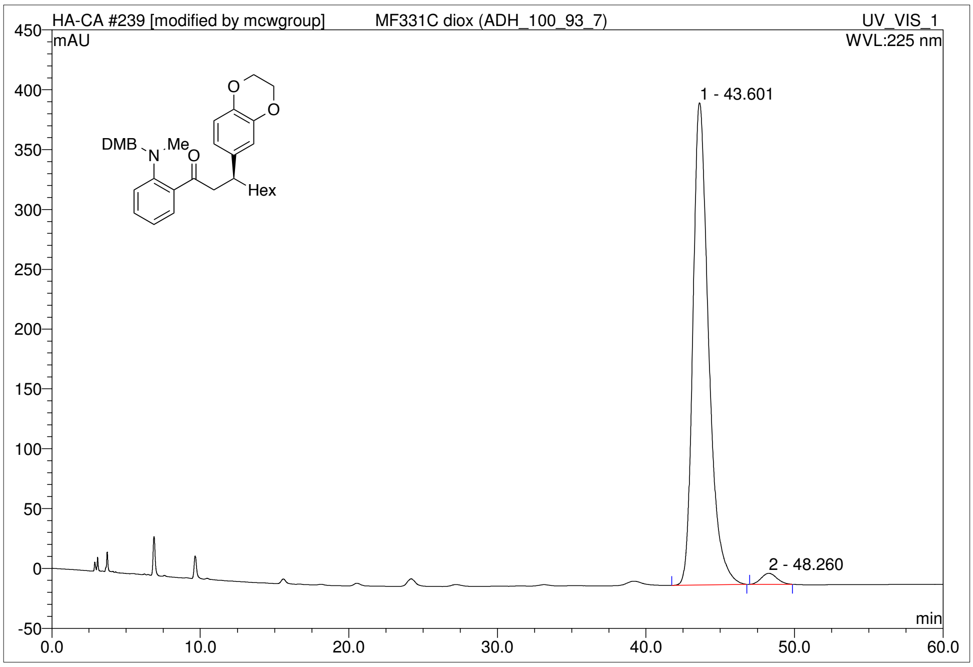

No.	Ret.Time min	Peak Name	Height mAU	Area mAU*min	Rel.Area %	Amount	Type
1	43.60	n.a.	403.015	493.716	97.78	n.a.	BMB*
2	48.26	n.a.	9.206	11.216	2.22	n.a.	BMB*
Total:			412.222	504.932	100.00	0.000	

Figure 63: HPLC chromatogram of compound **2o**

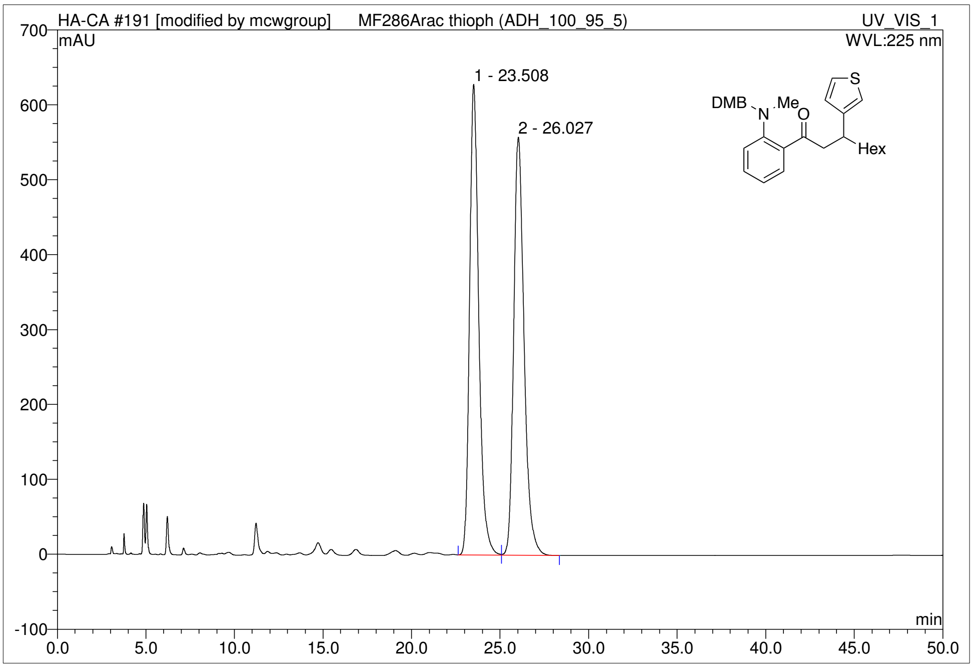

No.	Ret.Time min	Peak Name	Height mAU	Area mAU*min	Rel.Area %	Amount	Type
1	23.51	n.a.	628.198	378.614	49.95	n.a.	BM *
2	26.03	n.a.	558.257	379.301	50.05	n.a.	MB*
Total:			1186.455	757.915	100.00	0.000	

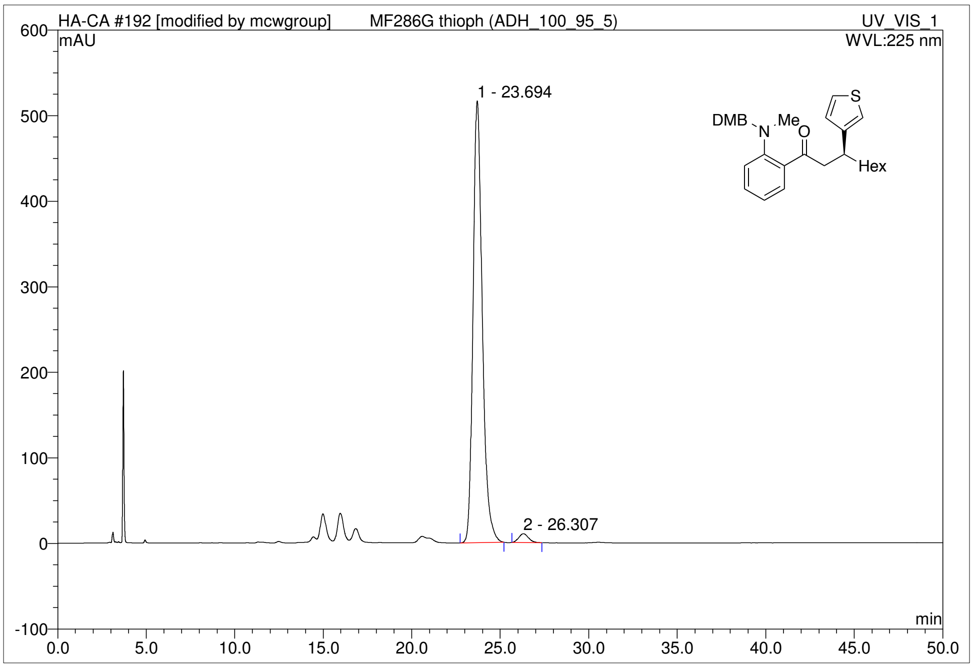

No.	Ret.Time min	Peak Name	Height mAU	Area mAU*min	Rel.Area %	Amount	Type
1	23.69	n.a.	516.356	313.631	97.95	n.a.	BMB*
2	26.31	n.a.	10.255	6.556	2.05	n.a.	BMB*
Total:			526.612	320.187	100.00	0.000	

Figure 64: HPLC chromatogram of compound **2p**

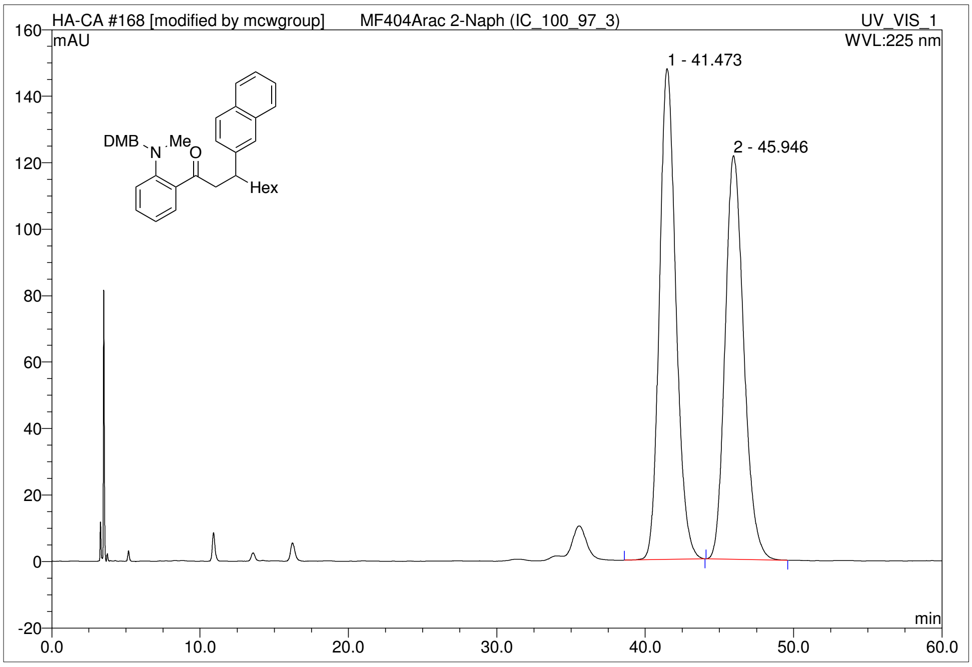

No.	Ret.Time min	Peak Name	Height mAU	Area mAU*min	Rel.Area %	Amount	Type
1	41.47	n.a.	147.692	190.135	51.78	n.a.	BMB*
2	45.95	n.a.	121.480	177.089	48.22	n.a.	BMB*
Total:			269.172	367.224	100.00	0.000	

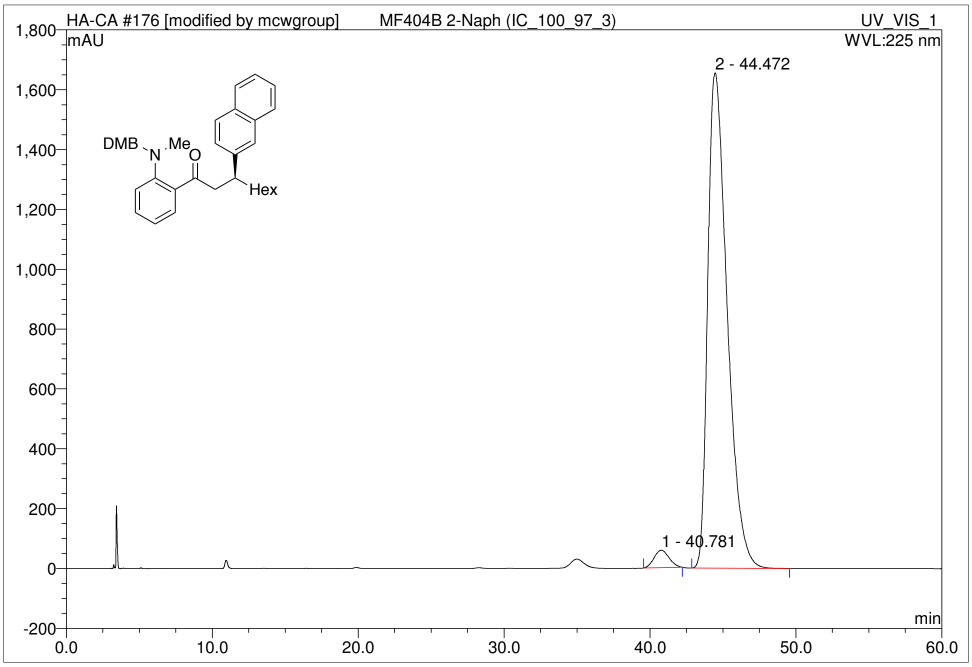

No.	Ret.Time min	Peak Name	Height mAU	Area mAU*min	Rel.Area %	Amount	Type
1	40.78	n.a.	58.277	69.217	2.65	n.a.	BMB*
2	44.47	n.a.	1655.160	2545.615	97.35	n.a.	BMB*
Total:			1713.437	2614.832	100.00	0.000	

Figure 65: HPLC chromatogram of compound **2q**

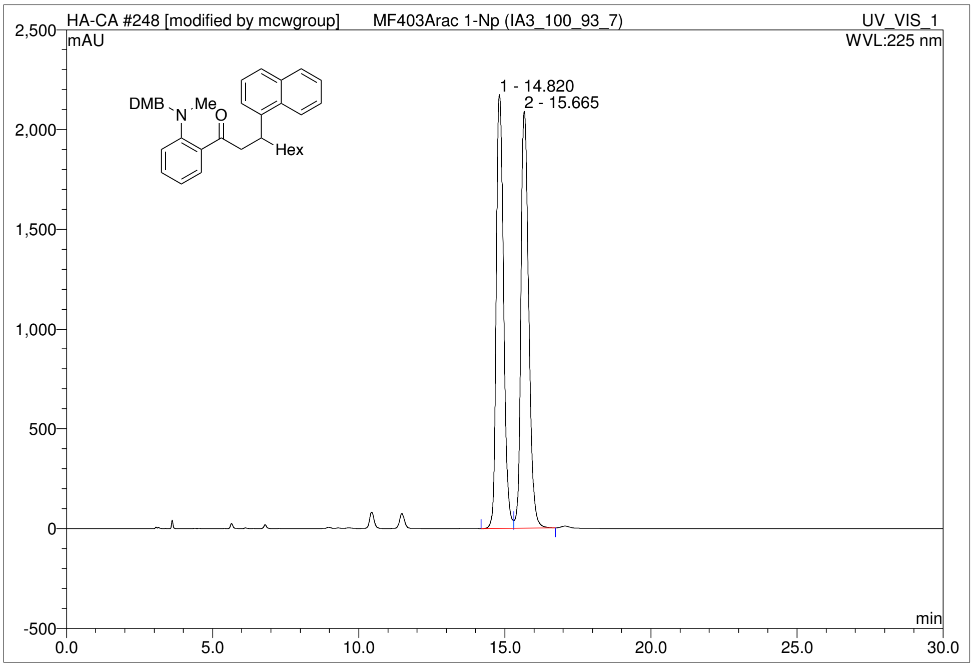

No.	Ret.Time min	Peak Name	Height mAU	Area mAU*min	Rel.Area %	Amount	Type
1	14.82	n.a.	2173.421	632.890	49.36	n.a.	BM *
2	15.67	n.a.	2089.155	649.242	50.64	n.a.	MB*
Total:			4262.576	1282.133	100.00	0.000	

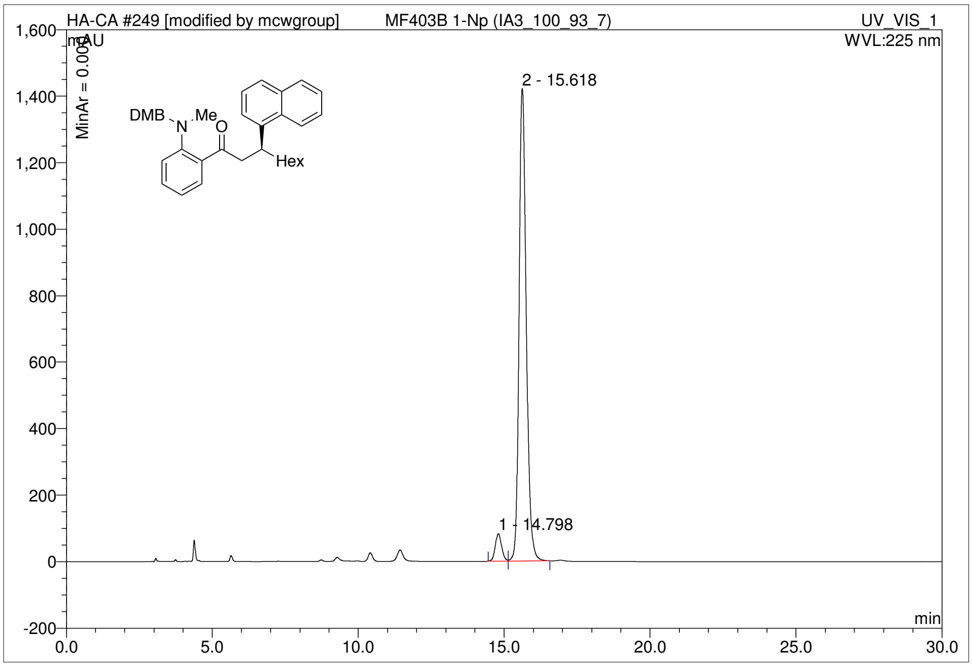

No.	Ret.Time min	Peak Name	Height mAU	Area mAU*min	Rel.Area %	Amount	Type
1	14.80	n.a.	82.676	20.992	4.92	n.a.	BM *
2	15.62	n.a.	1420.622	405.351	95.08	n.a.	MB*
Total:			1503.298	426.342	100.00	0.000	

Figure 66: HPLC chromatogram of compound **2r**

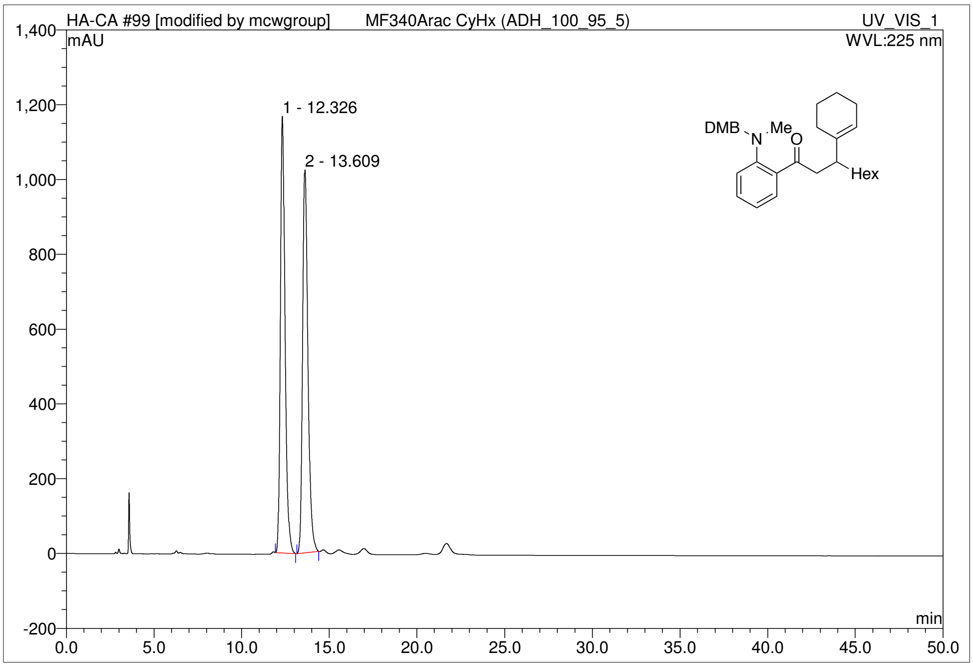

No.	Ret.Time min	Peak Name	Height mAU	Area mAU*min	Rel.Area %	Amount	Type
1	12.33	n.a.	1167.466	363.222	50.06	n.a.	BMB*
2	13.61	n.a.	1024.630	362.327	49.94	n.a.	BMB*
Total:			2192.096	725.549	100.00	0.000	

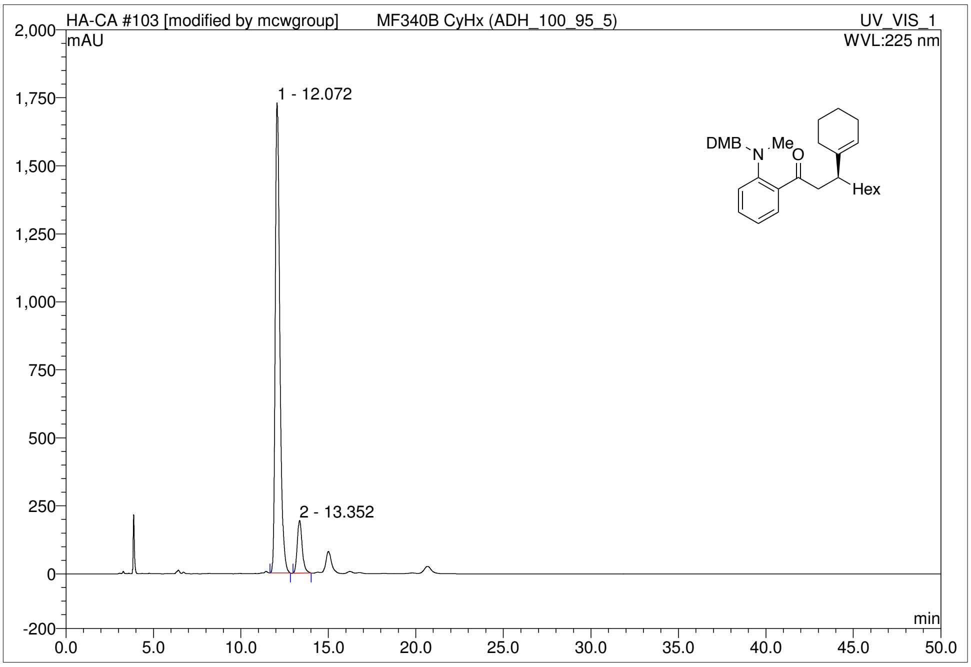

No.	Ret.Time min	Peak Name	Height mAU	Area mAU*min	Rel.Area %	Amount	Type
1	12.07	n.a.	1728.040	524.614	89.28	n.a.	BMB*
2	13.35	n.a.	193.089	63.009	10.72	n.a.	BMB*
Total:			1921.129	587.623	100.00	0.000	

Figure 67: HPLC chromatogram of compound **2s**

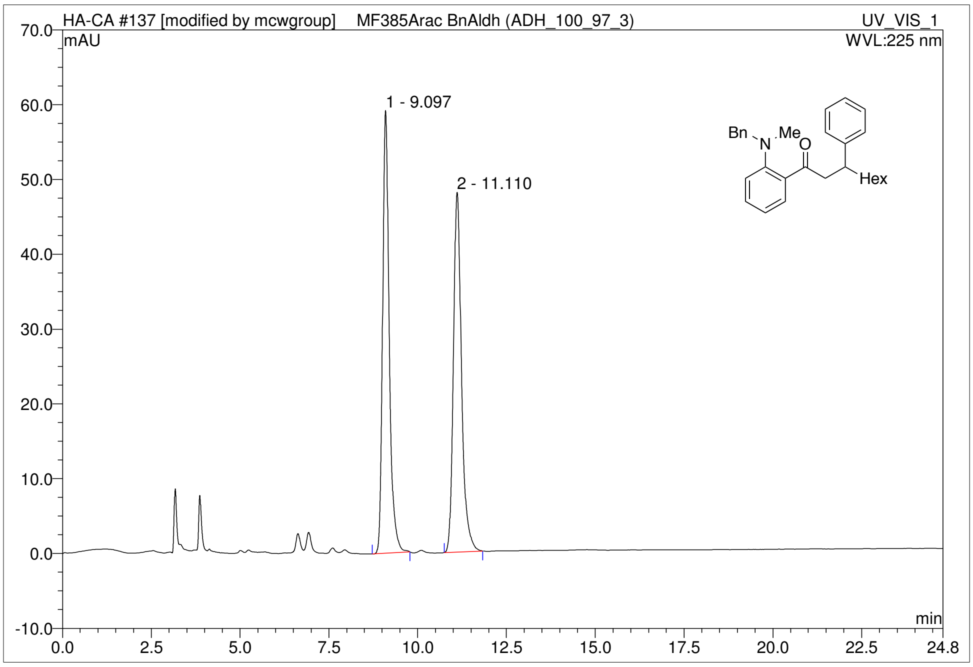

No.	Ret.Time min	Peak Name	Height mAU	Area mAU*min	Rel.Area %	Amount	Type
1	9.10	n.a.	59.153	12.763	50.02	n.a.	BMB*
2	11.11	n.a.	48.092	12.752	49.98	n.a.	BMB*
Total:			107.245	25.515	100.00	0.000	

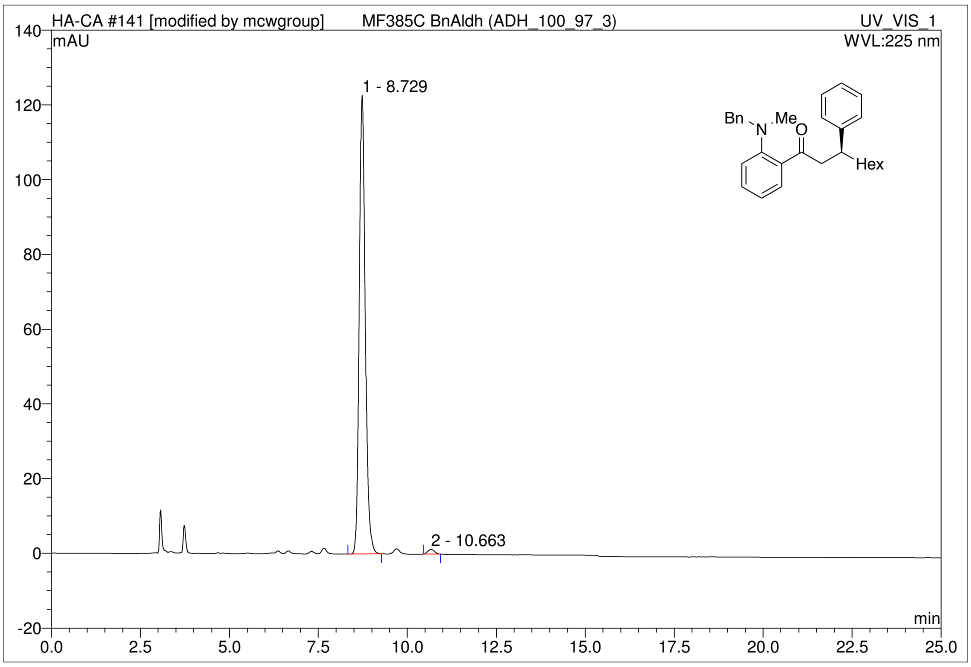

No.	Ret.Time min	Peak Name	Height mAU	Area mAU*min	Rel.Area %	Amount	Type
1	8.73	n.a.	122.700	24.268	98.90	n.a.	BMB*
2	10.66	n.a.	1.228	0.271	1.10	n.a.	BMB*
Total:			123.928	24.539	100.00	0.000	

Figure 68: HPLC chromatogram of compound **2v**

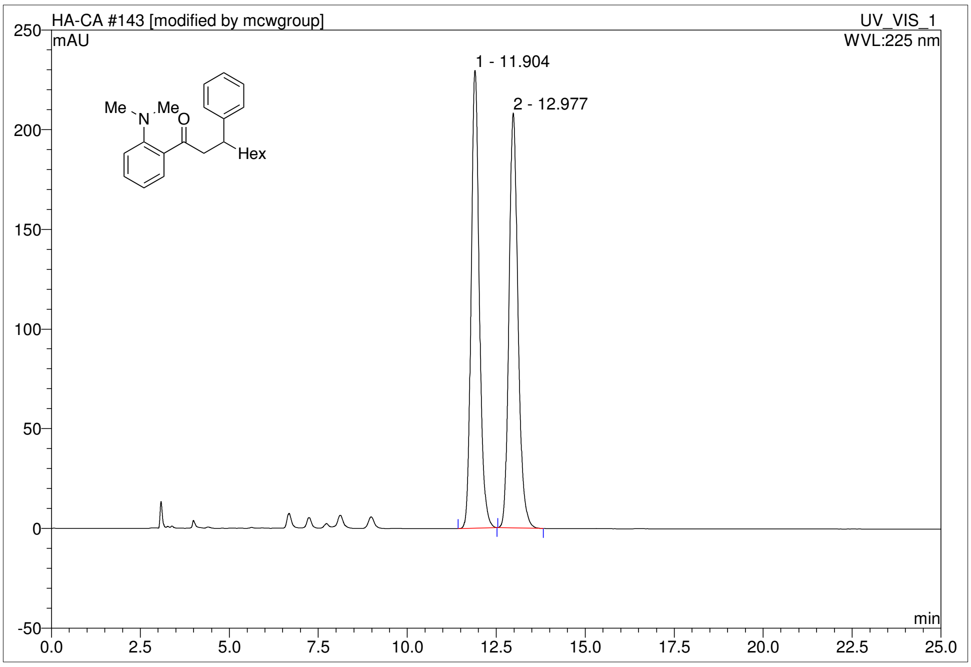

No.	Ret.Time min	Peak Name	Height mAU	Area mAU*min	Rel.Area %	Amount	Type
1	11.90	n.a.	229.609	60.987	49.99	n.a.	BMB*
2	12.98	n.a.	208.156	61.002	50.01	n.a.	BMB*
Total:			437.764	121.989	100.00	0.000	

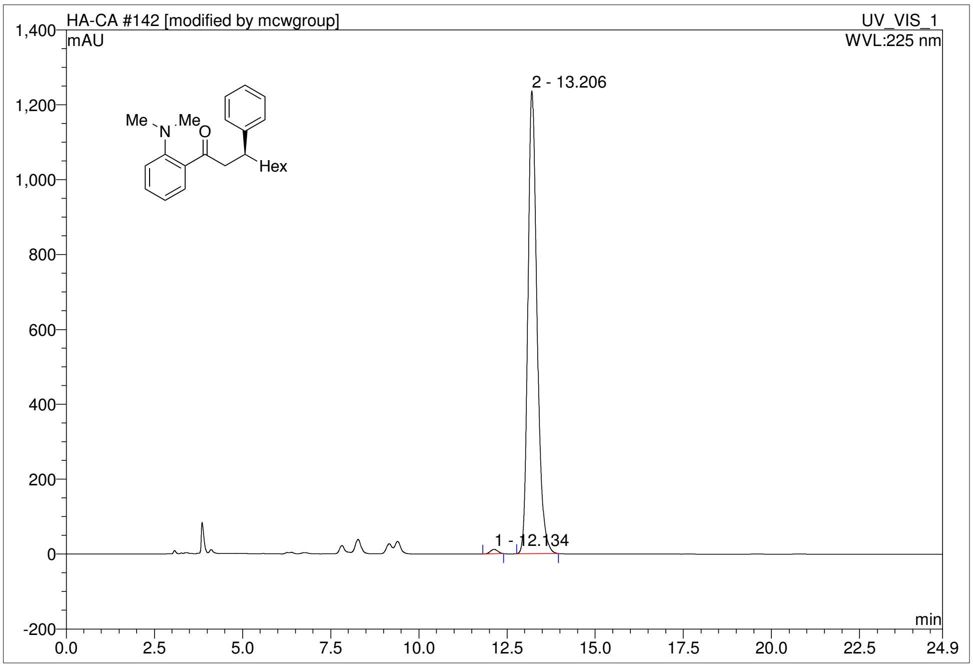

No.	Ret.Time min	Peak Name	Height mAU	Area mAU*min	Rel.Area %	Amount	Type
1	12.13	n.a.	11.920	2.907	0.78	n.a.	BMB*
2	13.21	n.a.	1235.843	371.348	99.22	n.a.	BMB*
Total:			1247.763	374.255	100.00	0.000	

Figure 69: HPLC chromatogram of compound **2w**

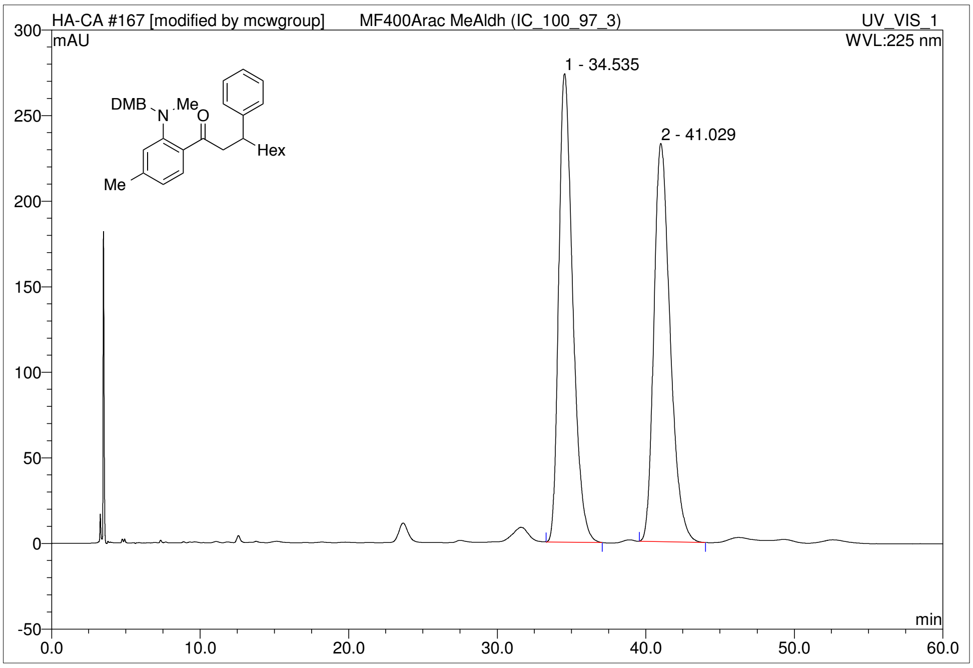

No.	Ret.Time<br>min	Peak Name	Height<br>mAU	Area<br>mAU*min	Rel.Area<br>%	Amount	Type
1	34.53	n.a.	273.737	298.999	50.31	n.a.	BMB*
2	41.03	n.a.	232.683	295.354	49.69	n.a.	BMB*
Total:			506.421	594.353	100.00	0.000	

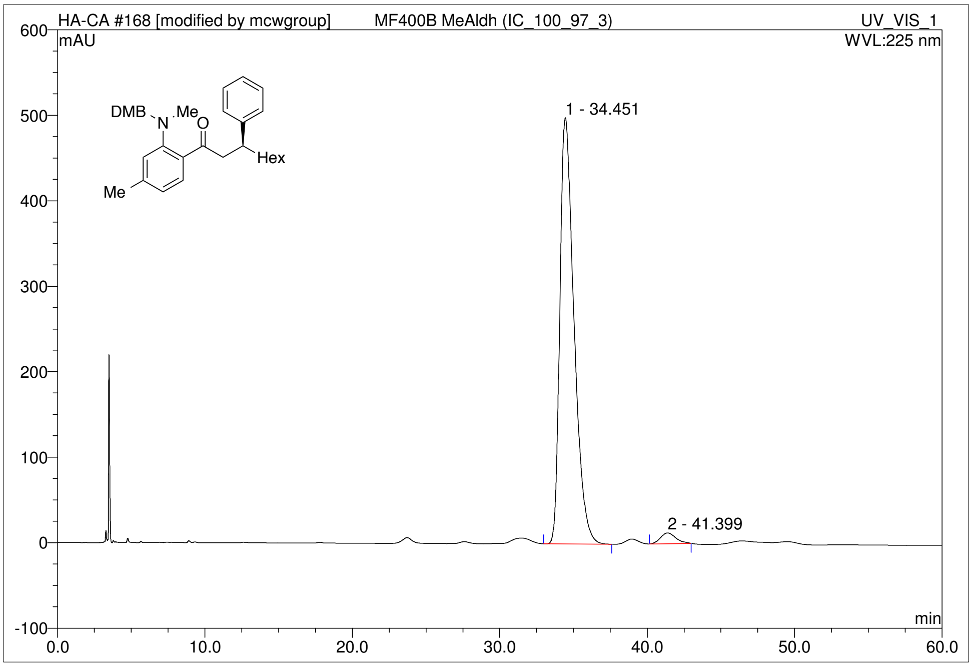

No.	Ret.Time<br>min	Peak Name	Height<br>mAU	Area<br>mAU*min	Rel.Area<br>%	Amount	Type
1	34.45	n.a.	498.682	554.031	97.36	n.a.	BMB*
2	41.40	n.a.	12.486	15.007	2.64	n.a.	BMB*
Total:			511.168	569.038	100.00	0.000	

Figure 70: HPLC chromatogram of compound **2aa**

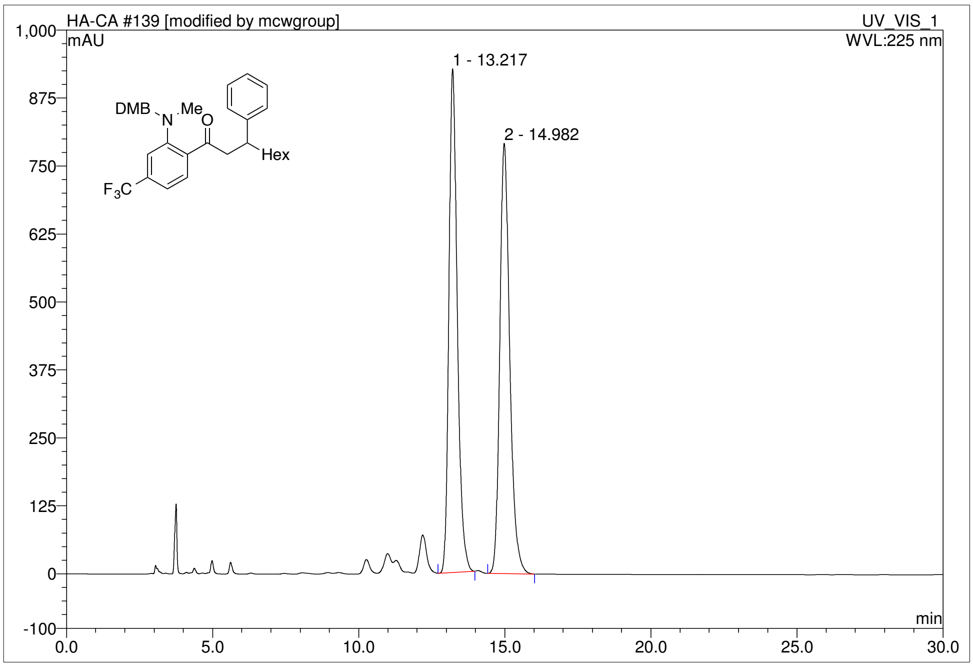

No.	Ret.Time min	Peak Name	Height mAU	Area mAU*min	Rel.Area %	Amount	Type
1	13.22	n.a.	926.046	307.529	49.75	n.a.	BMB*
2	14.98	n.a.	791.699	310.558	50.25	n.a.	BMB*
Total:			1717.745	618.087	100.00	0.000	

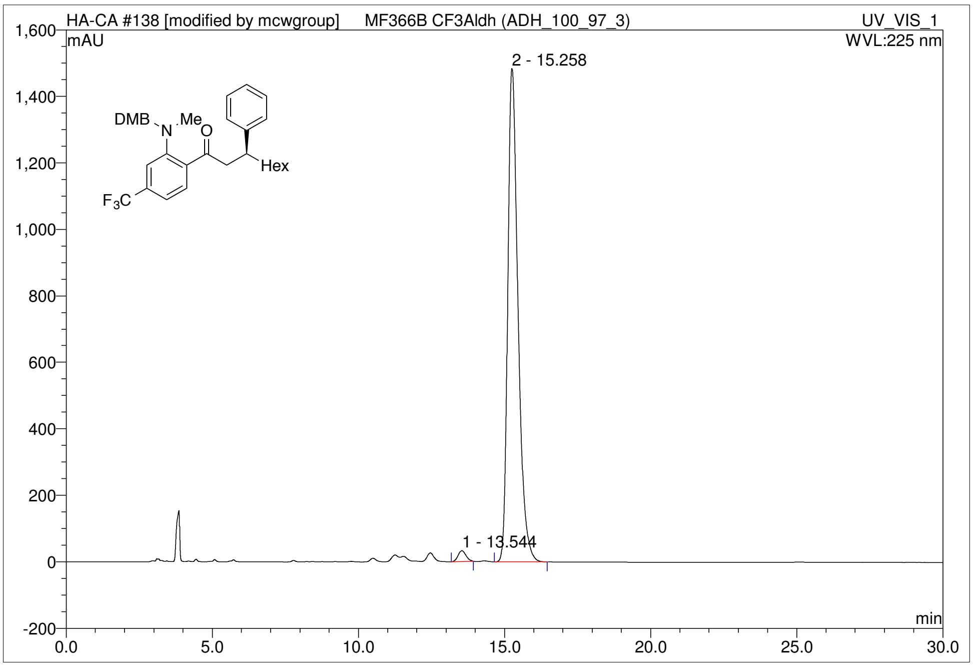

No.	Ret.Time min	Peak Name	Height mAU	Area mAU*min	Rel.Area %	Amount	Type
1	13.54	n.a.	32.664	10.237	1.67	n.a.	BMB*
2	15.26	n.a.	1484.144	604.087	98.33	n.a.	BMB*
Total:			1516.807	614.324	100.00	0.000	

Figure 71: HPLC chromatogram of compound **2ab**

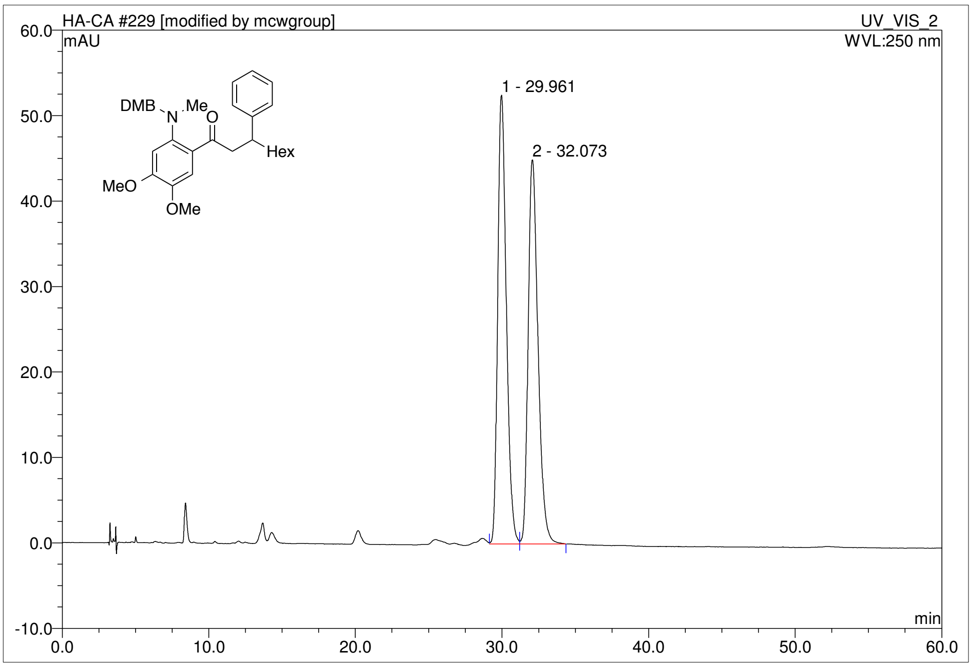

No.	Ret.Time min	Peak Name	Height mAU	Area mAU*min	Rel.Area %	Amount	Type
1	29.96	n.a.	52.510	35.713	50.52	n.a.	M *
2	32.07	n.a.	44.946	34.976	49.48	n.a.	MB*
Total:			97.456	70.689	100.00	0.000	

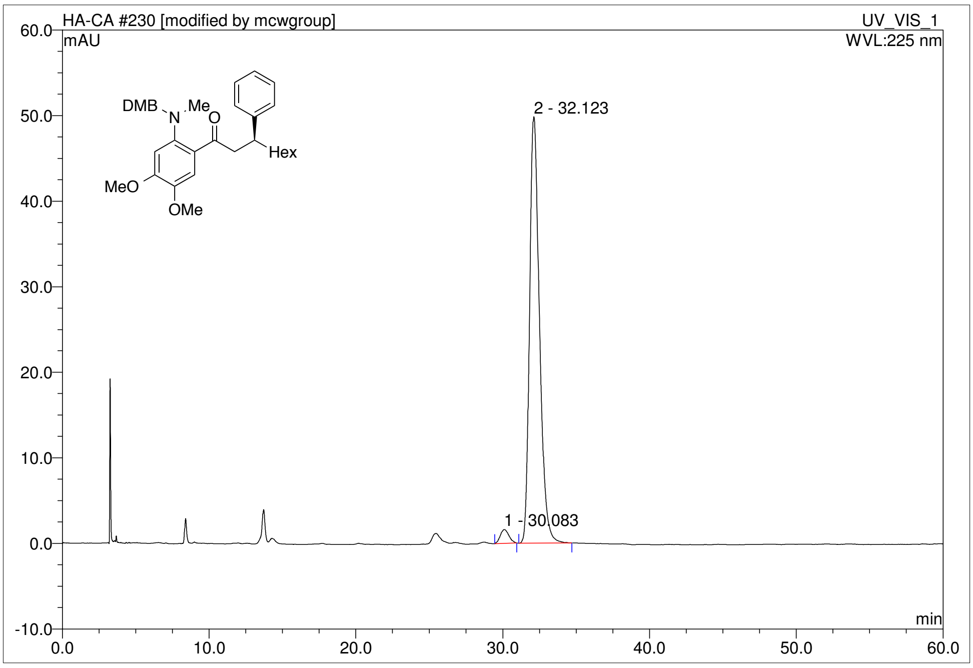

No.	Ret.Time min	Peak Name	Height mAU	Area mAU*min	Rel.Area %	Amount	Type
1	30.08	n.a.	1.608	1.093	2.73	n.a.	BMB*
2	32.12	n.a.	49.850	38.888	97.27	n.a.	BMB*
Total:			51.458	39.981	100.00	0.000	

Figure 72: HPLC chromatogram of compound **2ac**

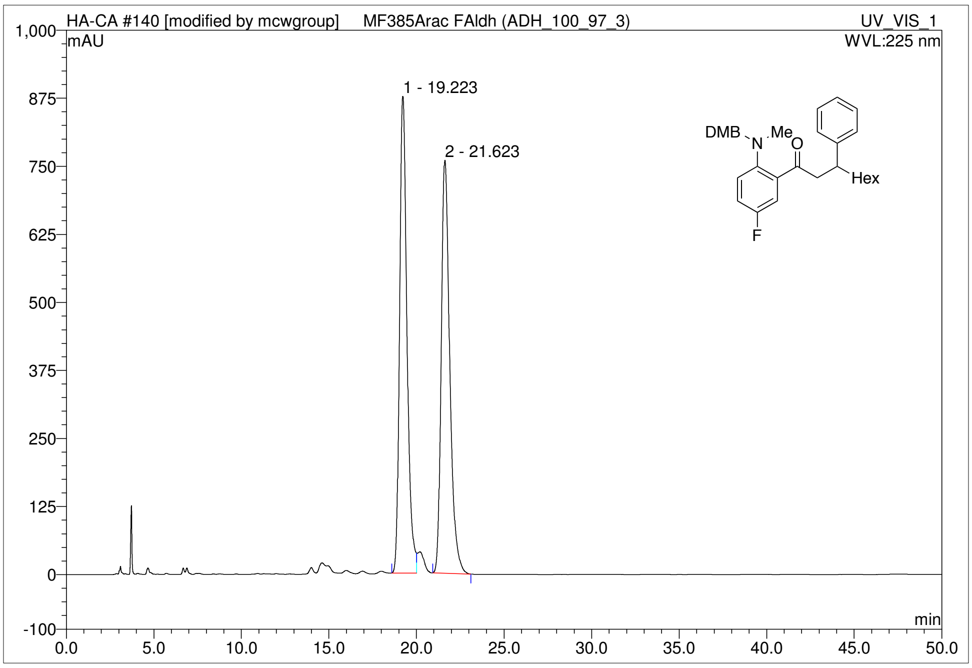

No.	Ret.Time min	Peak Name	Height mAU	Area mAU*min	Rel.Area %	Amount	Type
1	19.22	n.a.	875.585	427.415	49.94	n.a.	BM *
2	21.62	n.a.	758.619	428.365	50.06	n.a.	BMB*
Total:			1634.204	855.780	100.00	0.000	

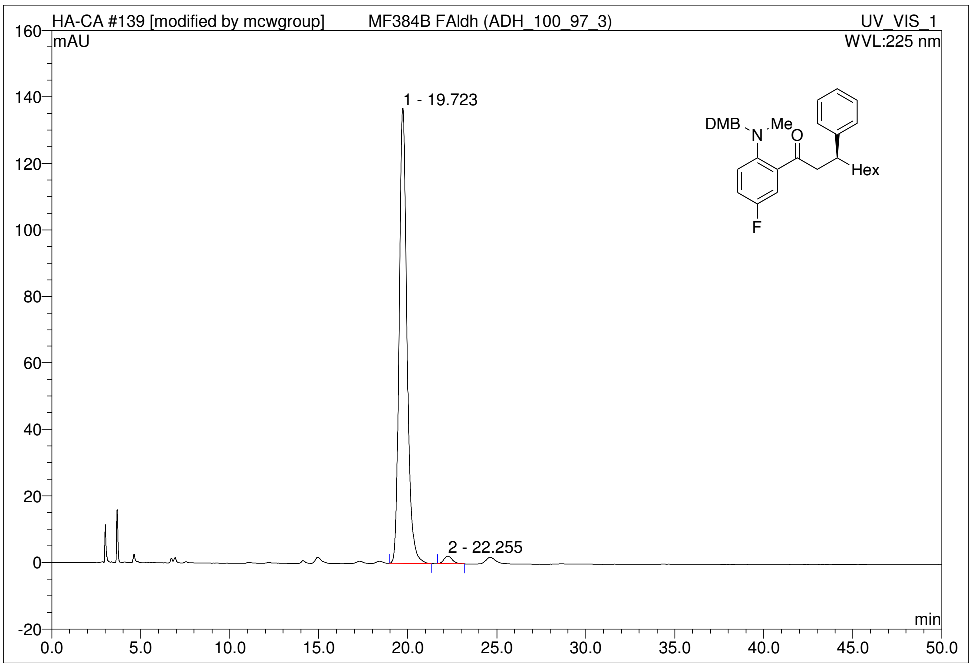

No.	Ret.Time min	Peak Name	Height mAU	Area mAU*min	Rel.Area %	Amount	Type
1	19.72	n.a.	136.774	69.203	98.21	n.a.	BMB*
2	22.26	n.a.	2.279	1.260	1.79	n.a.	BMB*
Total:			139.053	70.463	100.00	0.000	

Figure 73: HPLC chromatogram of compound **2ad**

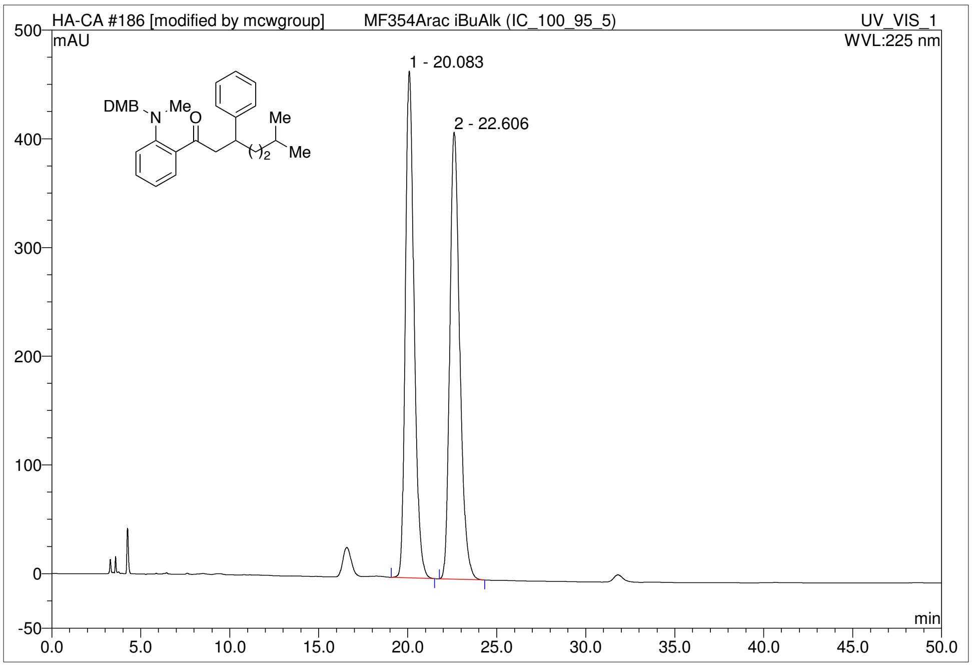

No.	Ret.Time min	Peak Name	Height mAU	Area mAU*min	Rel.Area %	Amount	Type
1	20.08	n.a.	466.041	265.107	49.89	n.a.	BMB*
2	22.61	n.a.	410.961	266.297	50.11	n.a.	BMB*
Total:			877.002	531.404	100.00	0.000	

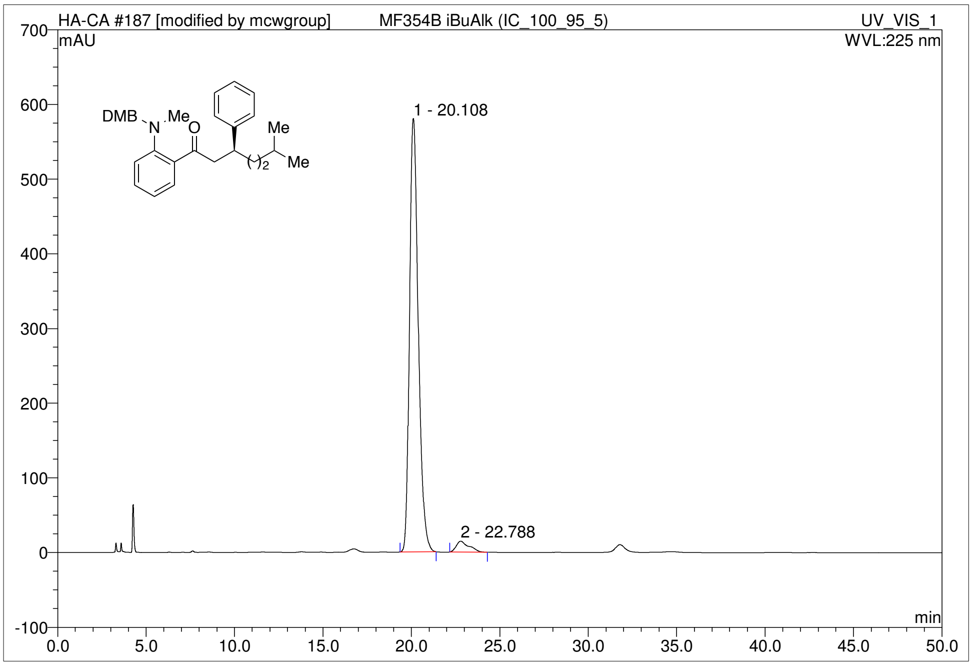

No.	Ret.Time min	Peak Name	Height mAU	Area mAU*min	Rel.Area %	Amount	Type
1	20.11	n.a.	580.171	332.973	96.47	n.a.	BMB*
2	22.79	n.a.	14.525	12.198	3.53	n.a.	BMB*
Total:			594.695	345.171	100.00	0.000	

Figure 74: HPLC chromatogram of compound **2ae**

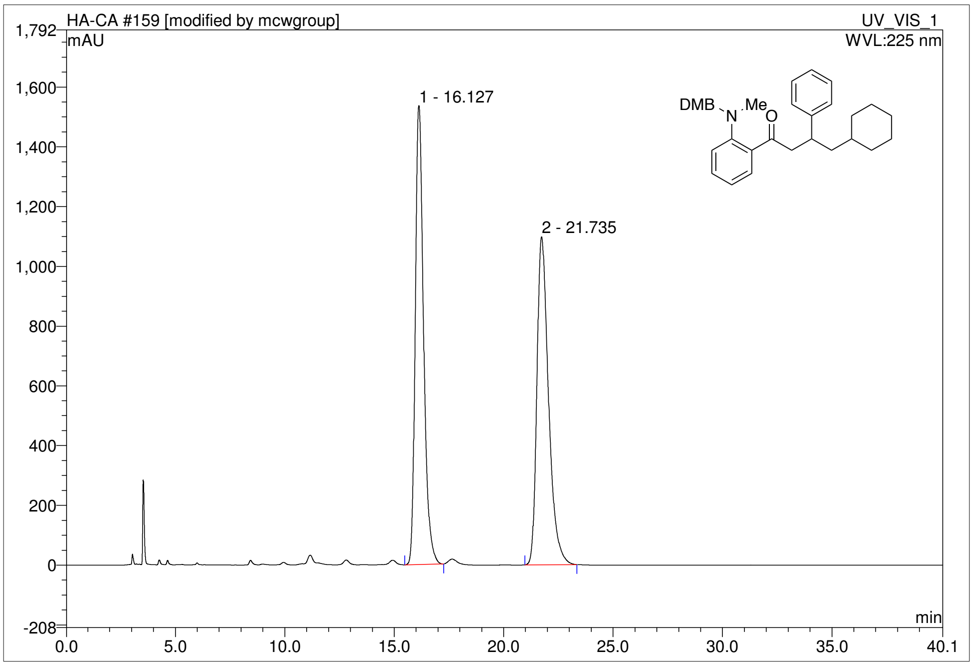

No.	Ret.Time min	Peak Name	Height mAU	Area mAU*min	Rel.Area %	Amount	Type
1	16.13	n.a.	1535.733	674.140	49.90	n.a.	BMB*
2	21.73	n.a.	1098.146	676.814	50.10	n.a.	BMB*
Total:			2633.879	1350.954	100.00	0.000	

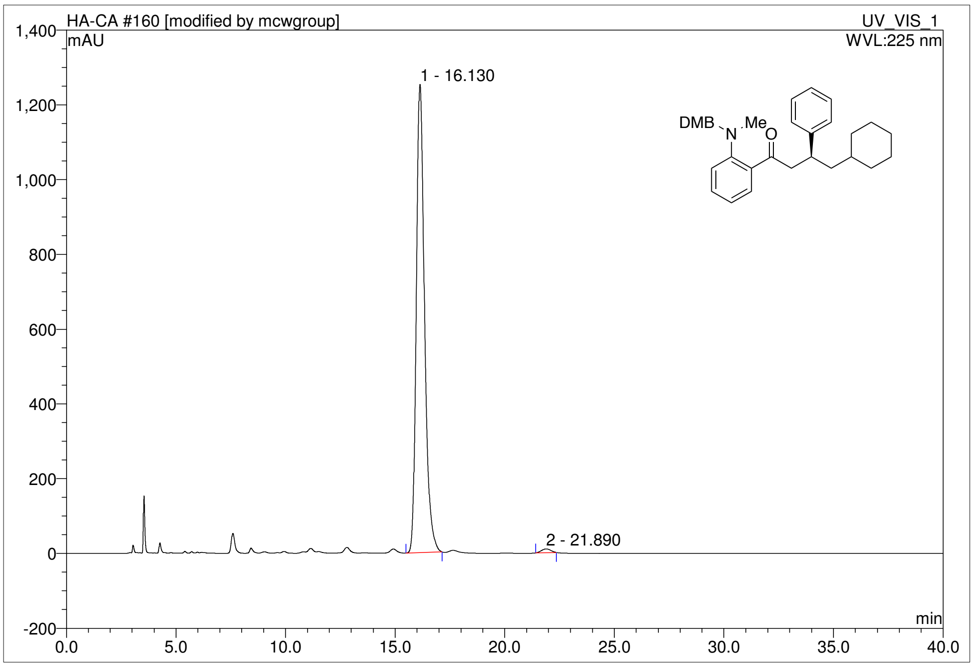

No.	Ret.Time min	Peak Name	Height mAU	Area mAU*min	Rel.Area %	Amount	Type
1	16.13	n.a.	1252.655	544.522	99.09	n.a.	BMB*
2	21.89	n.a.	10.221	4.989	0.91	n.a.	BMB*
Total:			1262.876	549.511	100.00	0.000	

Figure 75: HPLC chromatogram of compound **2ah**

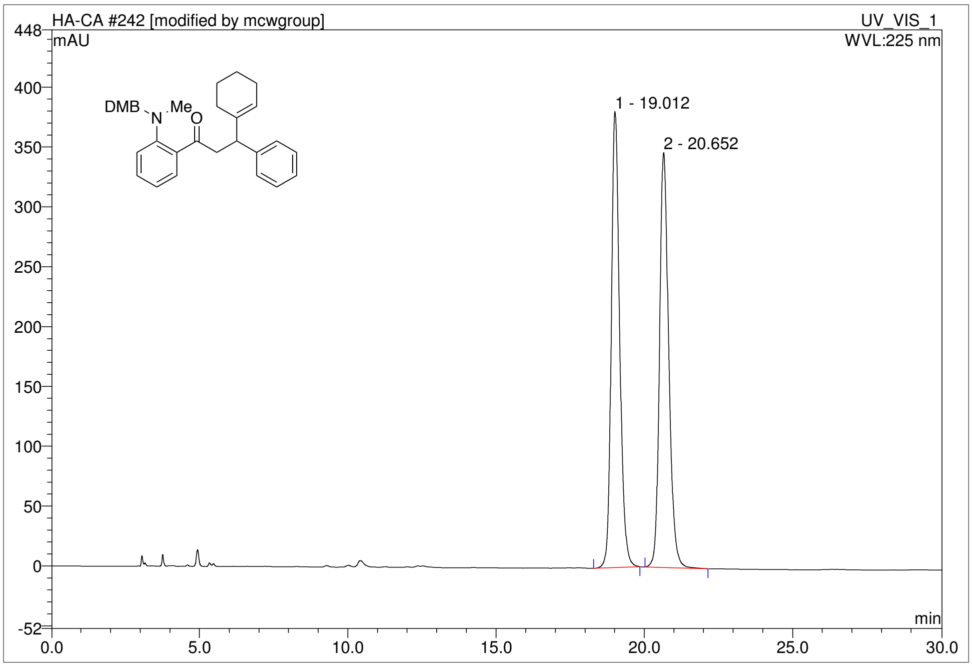

No.	Ret.Time min	Peak Name	Height mAU	Area mAU*min	Rel.Area %	Amount	Type
1	19.01	n.a.	380.870	125.361	50.08	n.a.	BMB*
2	20.65	n.a.	346.666	124.964	49.92	n.a.	BMB*
Total:			727.536	250.326	100.00	0.000	

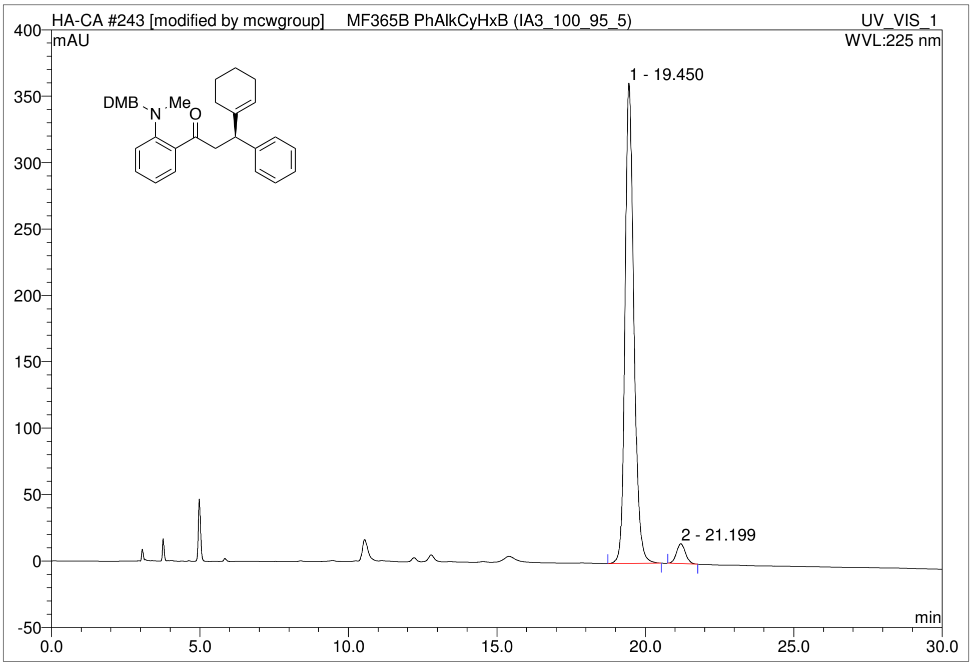

No.	Ret.Time min	Peak Name	Height mAU	Area mAU*min	Rel.Area %	Amount	Type
1	19.45	n.a.	361.636	124.277	95.90	n.a.	BMB*
2	21.20	n.a.	15.023	5.312	4.10	n.a.	BMB*
Total:			376.658	129.590	100.00	0.000	

Figure 76: HPLC chromatogram of compound **2aj**

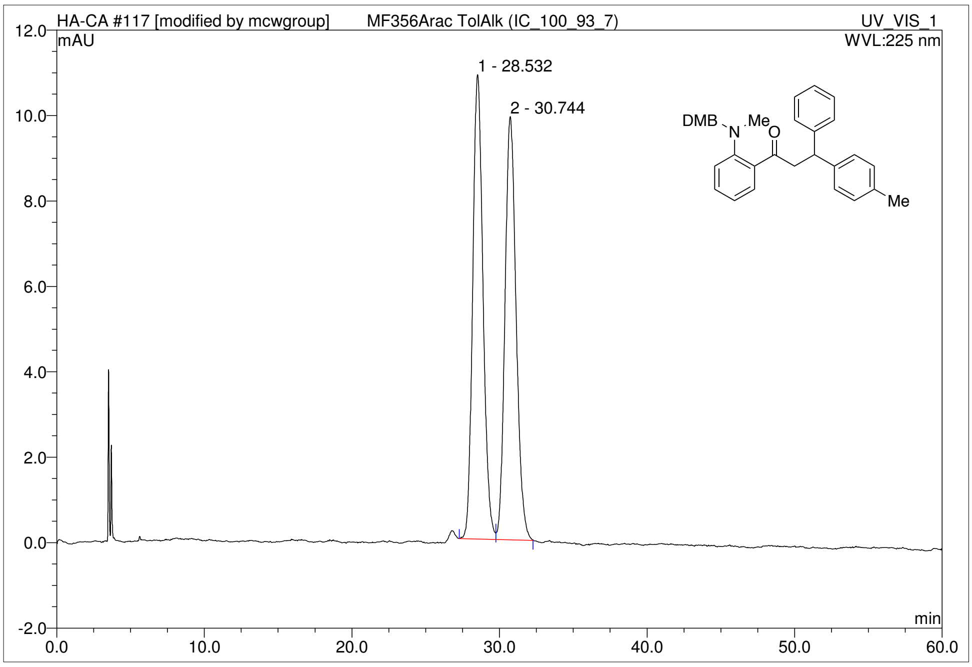

No.	Ret.Time min	Peak Name	Height mAU	Area mAU*min	Rel.Area %	Amount	Type
1	28.53	n.a.	10.872	8.797	50.31	n.a.	BM *
2	30.74	n.a.	9.909	8.688	49.69	n.a.	MB*
Total:			20.781	17.484	100.00	0.000	

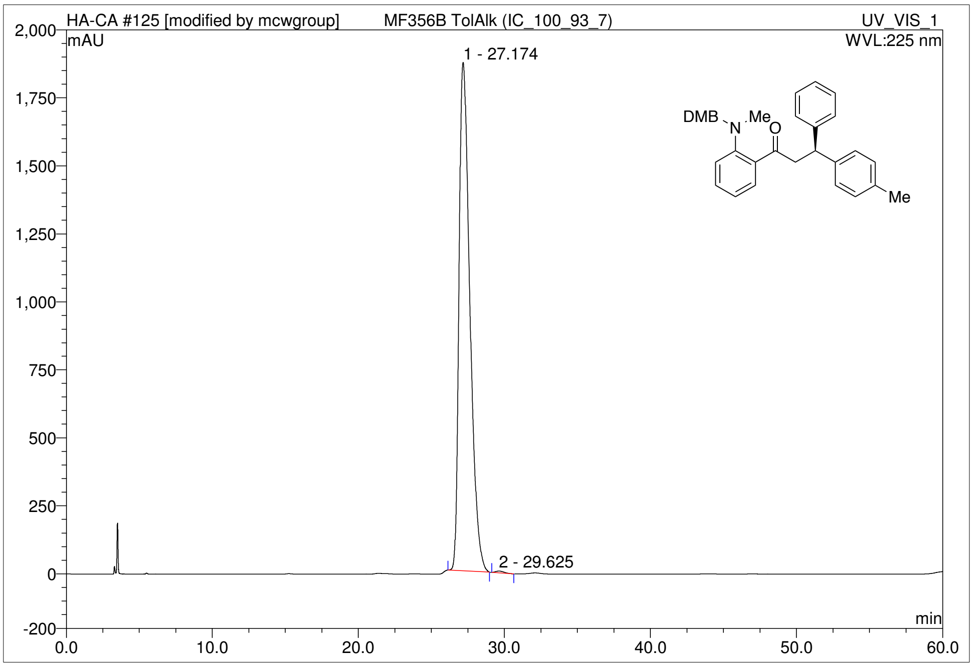

No.	Ret.Time min	Peak Name	Height mAU	Area mAU*min	Rel.Area %	Amount	Type
1	27.17	n.a.	1868.427	1605.675	99.72	n.a.	BMB*
2	29.63	n.a.	6.735	4.480	0.28	n.a.	BMB*
Total:			1875.162	1610.155	100.00	0.000	

Figure 77: HPLC chromatogram of compound **2ak**

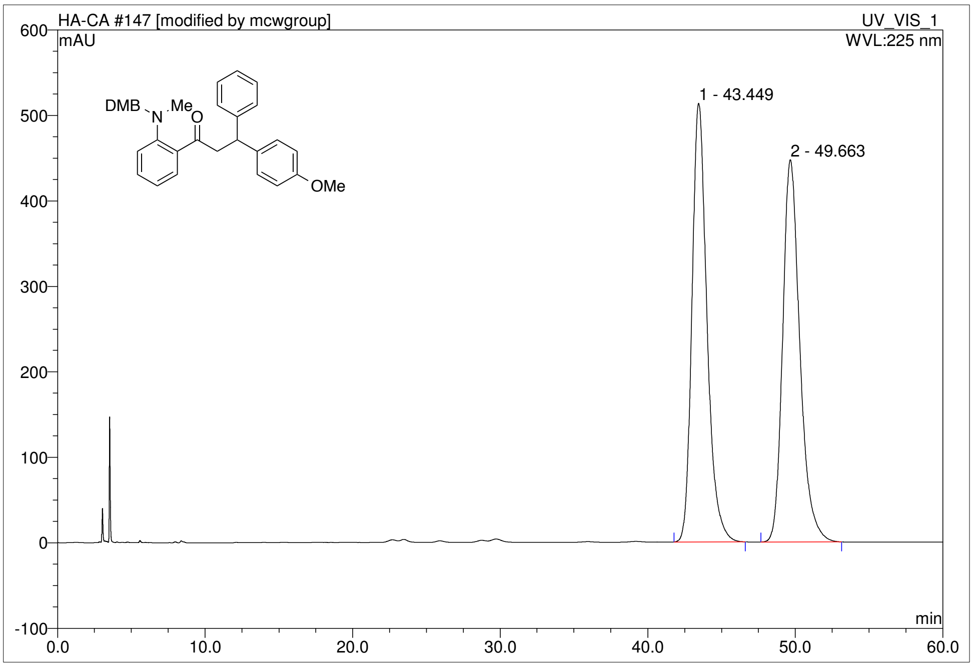

No.	Ret.Time min	Peak Name	Height mAU	Area mAU*min	Rel.Area %	Amount	Type
1	43.45	n.a.	513.292	605.426	49.99	n.a.	BMB*
2	49.66	n.a.	447.288	605.567	50.01	n.a.	BMB*
Total:			960.580	1210.993	100.00	0.000	

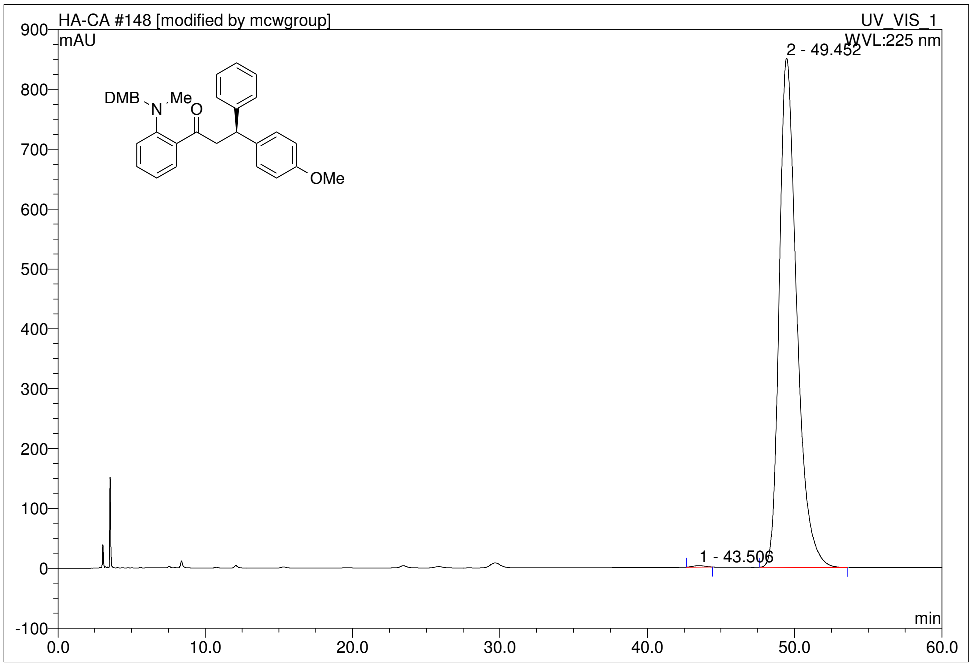

No.	Ret.Time min	Peak Name	Height mAU	Area mAU*min	Rel.Area %	Amount	Type
1	43.51	n.a.	2.455	2.285	0.19	n.a.	BMB*
2	49.45	n.a.	849.968	1170.213	99.81	n.a.	BMB*
Total:			852.422	1172.498	100.00	0.000	

Figure 78: HPLC chromatogram of compound **2al**

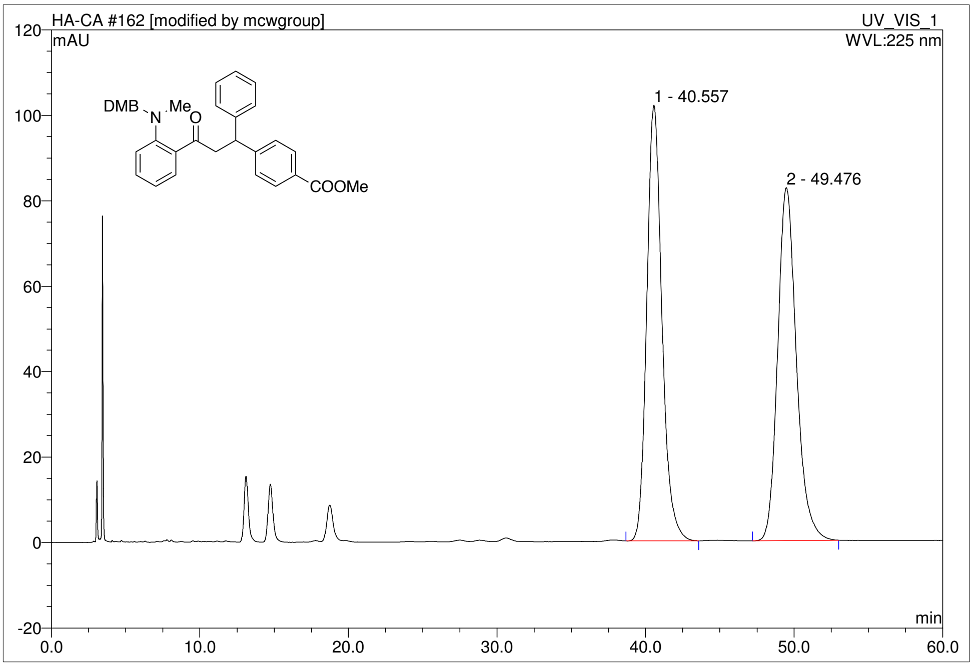

No.	Ret.Time min	Peak Name	Height mAU	Area mAU*min	Rel.Area %	Amount	Type
1	40.56	n.a.	101.962	120.680	49.99	n.a.	BMB*
2	49.48	n.a.	82.632	120.726	50.01	n.a.	BMB*
Total:			184.594	241.406	100.00	0.000	

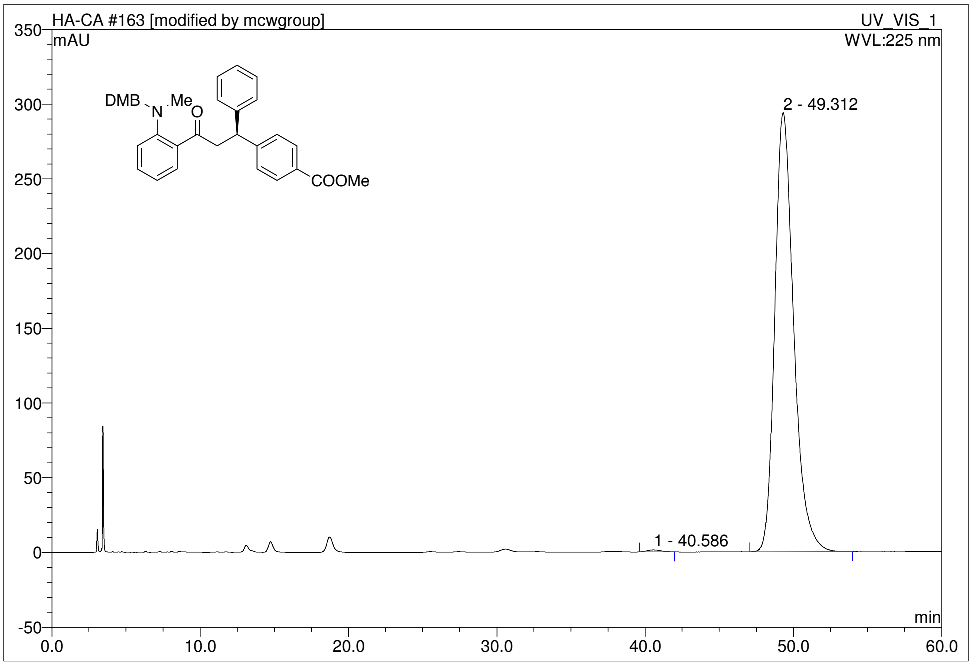

No.	Ret.Time min	Peak Name	Height mAU	Area mAU*min	Rel.Area %	Amount	Type
1	40.59	n.a.	1.313	1.392	0.32	n.a.	BMB*
2	49.31	n.a.	293.842	433.989	99.68	n.a.	BMB*
Total:			295.155	435.381	100.00	0.000	

Figure 79: HPLC chromatogram of compound **2am**

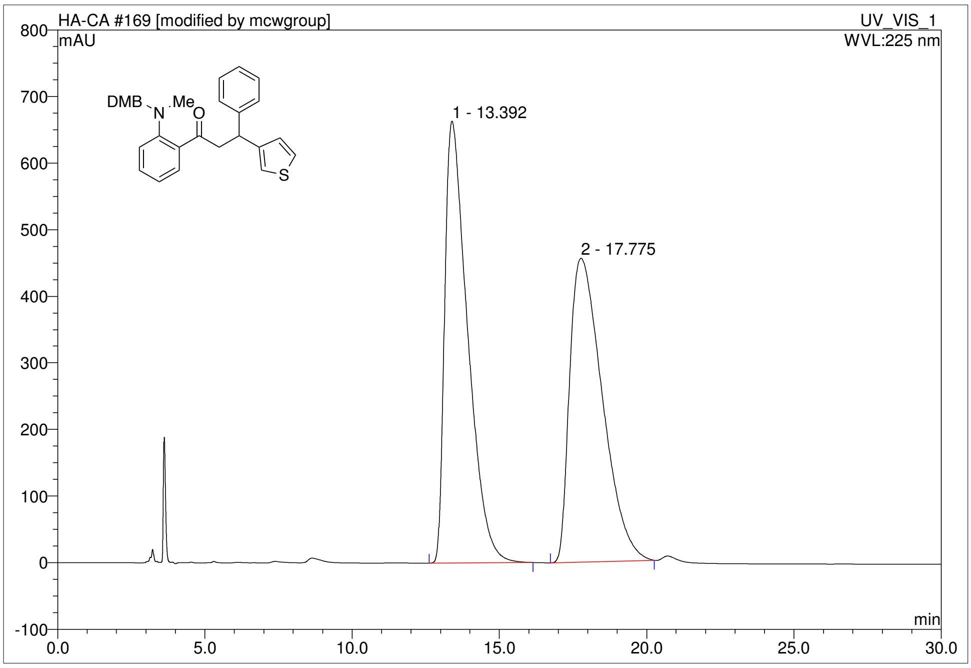

No.	Ret.Time min	Peak Name	Height mAU	Area mAU*min	Rel.Area %	Amount	Type
1	13.39	n.a.	663.785	598.440	50.51	n.a.	BMB*
2	17.77	n.a.	456.496	586.429	49.49	n.a.	BMB*
Total:			1120.281	1184.868	100.00	0.000	

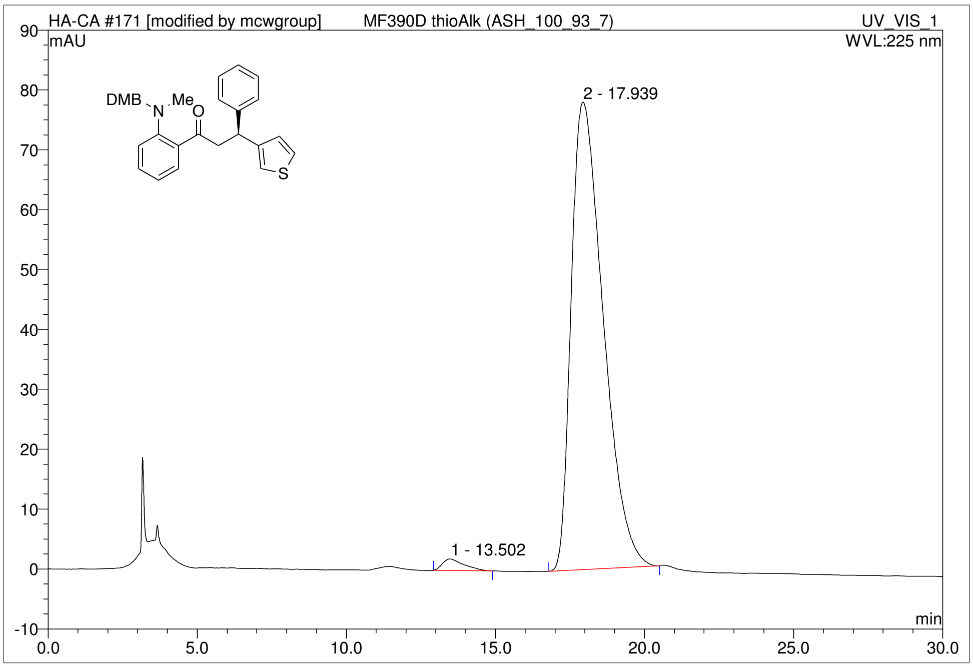

No.	Ret.Time min	Peak Name	Height mAU	Area mAU*min	Rel.Area %	Amount	Type
1	13.50	n.a.	1.908	1.579	1.60	n.a.	BMB*
2	17.94	n.a.	78.081	97.084	98.40	n.a.	BMB*
Total:			79.989	98.663	100.00	0.000	

Figure 80: HPLC chromatogram of compound **2an**

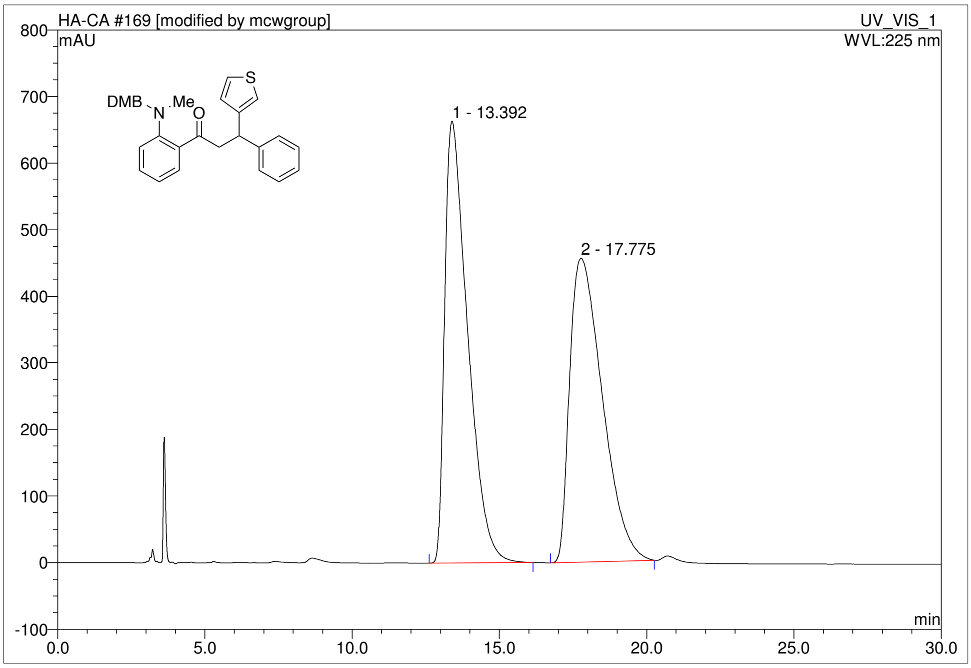

No.	Ret.Time min	Peak Name	Height mAU	Area mAU*min	Rel.Area %	Amount	Type
1	13.39	n.a.	663.785	598.440	50.51	n.a.	BMB*
2	17.77	n.a.	456.496	586.429	49.49	n.a.	BMB*
Total:			1120.281	1184.868	100.00	0.000	

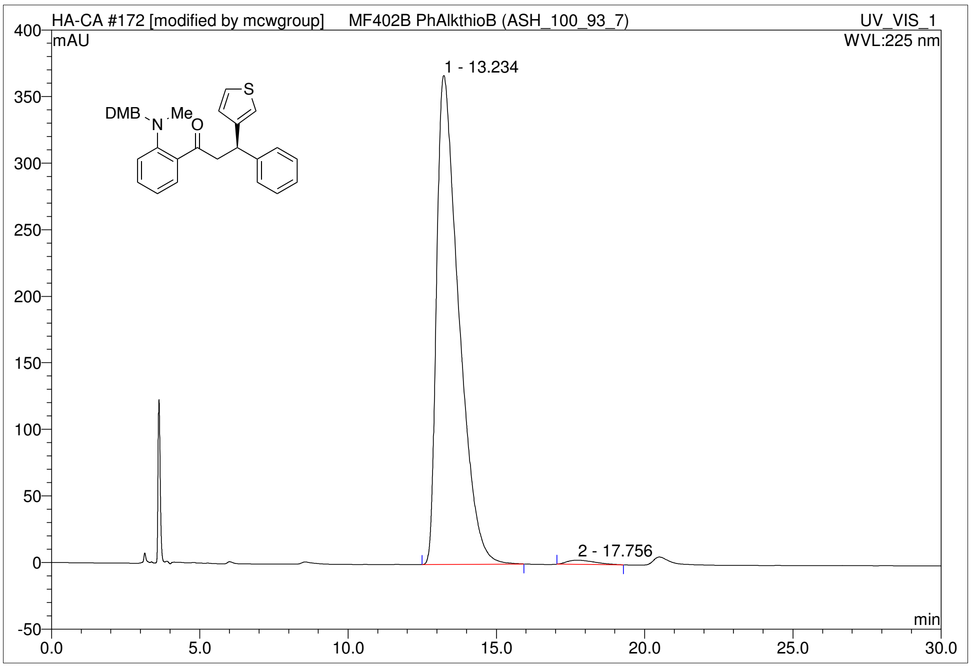

No.	Ret.Time min	Peak Name	Height mAU	Area mAU*min	Rel.Area %	Amount	Type
1	13.23	n.a.	367.216	316.404	98.93	n.a.	BMB*
2	17.76	n.a.	3.186	3.430	1.07	n.a.	BMB*
Total:			370.402	319.835	100.00	0.000	

Figure 81: HPLC chromatogram of compound **2an**

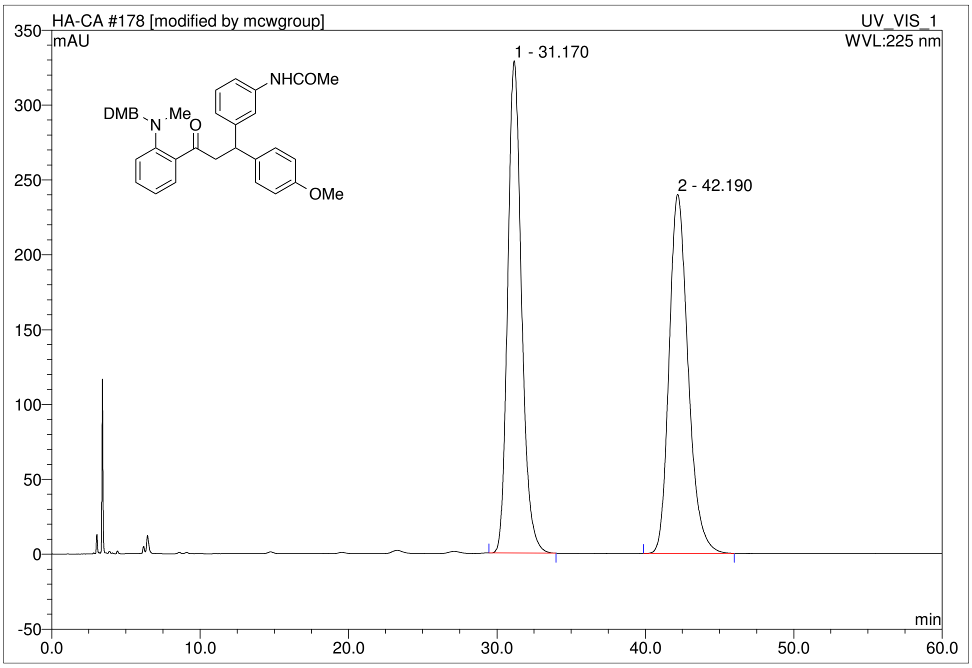

No.	Ret.Time min	Peak Name	Height mAU	Area mAU*min	Rel.Area %	Amount	Type
1	31.17	n.a.	328.716	355.657	49.97	n.a.	BMB
2	42.19	n.a.	239.928	356.135	50.03	n.a.	BMB
Total:			568.644	711.792	100.00	0.000	

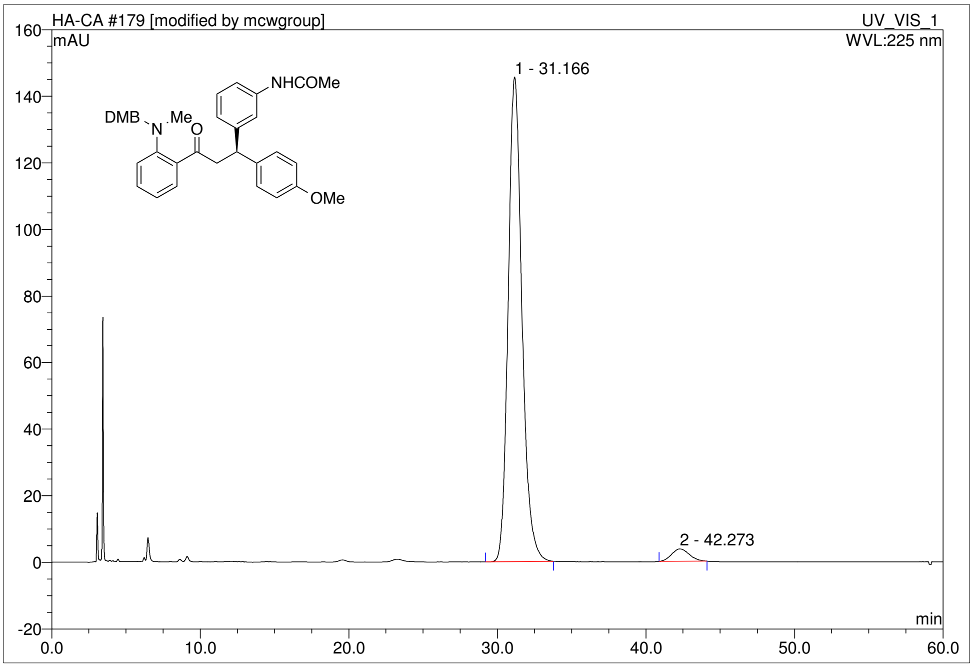

No.	Ret.Time min	Peak Name	Height mAU	Area mAU*min	Rel.Area %	Amount	Type
1	31.17	n.a.	145.557	156.370	96.81	n.a.	BMB*
2	42.27	n.a.	3.736	5.159	3.19	n.a.	BMB*
Total:			149.293	161.529	100.00	0.000	

Figure 82: HPLC chromatogram of compound **2ap**

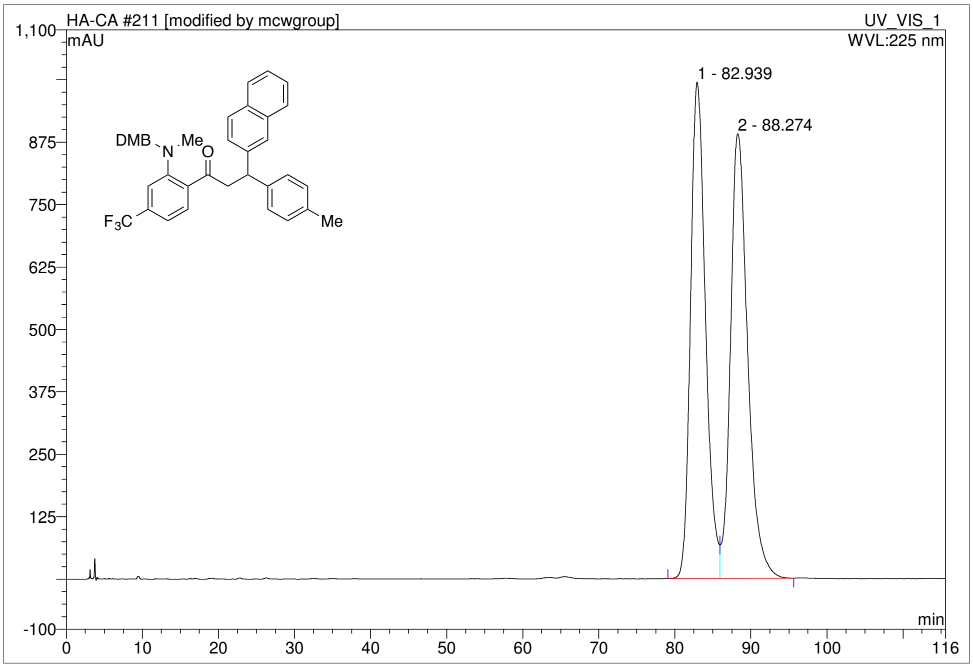

No.	Ret.Time min	Peak Name	Height mAU	Area mAU*min	Rel.Area %	Amount	Type
1	82.94	n.a.	993.627	2267.760	49.29	n.a.	BM *
2	88.27	n.a.	890.047	2333.008	50.71	n.a.	MB*
Total:			1883.675	4600.768	100.00	0.000	

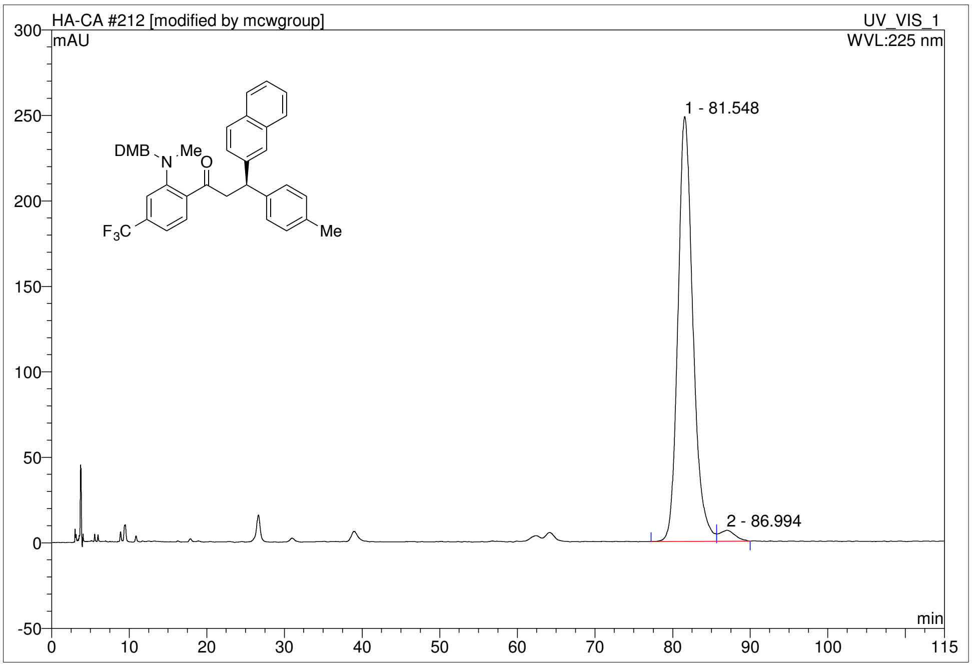

No.	Ret.Time min	Peak Name	Height mAU	Area mAU*min	Rel.Area %	Amount	Type
1	81.55	n.a.	248.493	555.882	97.39	n.a.	BM *
2	86.99	n.a.	6.420	14.918	2.61	n.a.	MB*
Total:			254.913	570.800	100.00	0.000	

Figure 83: HPLC chromatogram of compound **2aq**

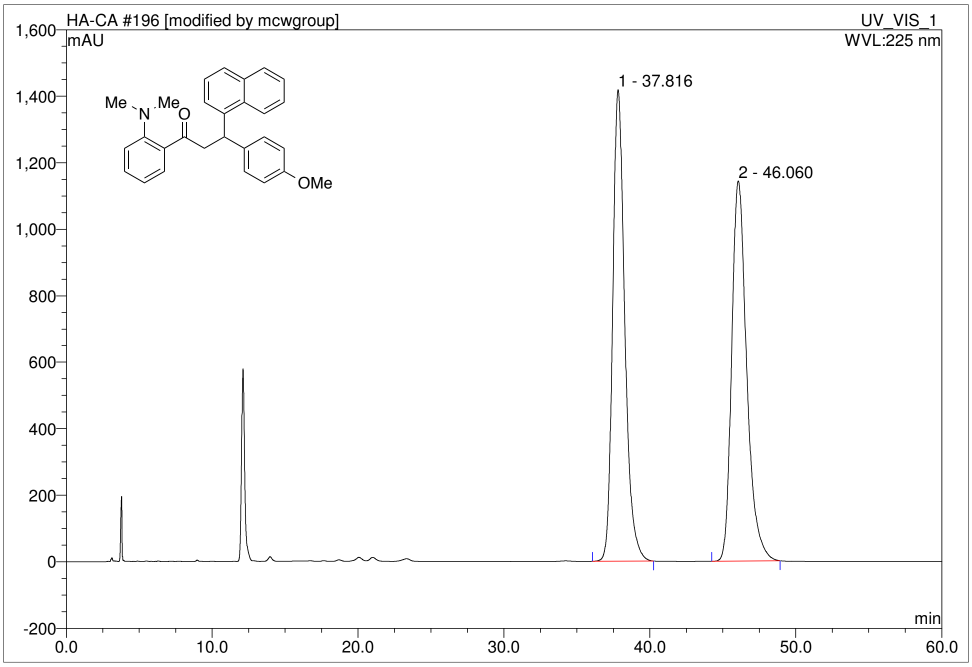

No.	Ret.Time min	Peak Name	Height mAU	Area mAU*min	Rel.Area %	Amount	Type
1	37.82	n.a.	1417.832	1361.795	49.95	n.a.	BMB*
2	46.06	n.a.	1143.435	1364.712	50.05	n.a.	BMB*
Total:			2561.268	2726.507	100.00	0.000	

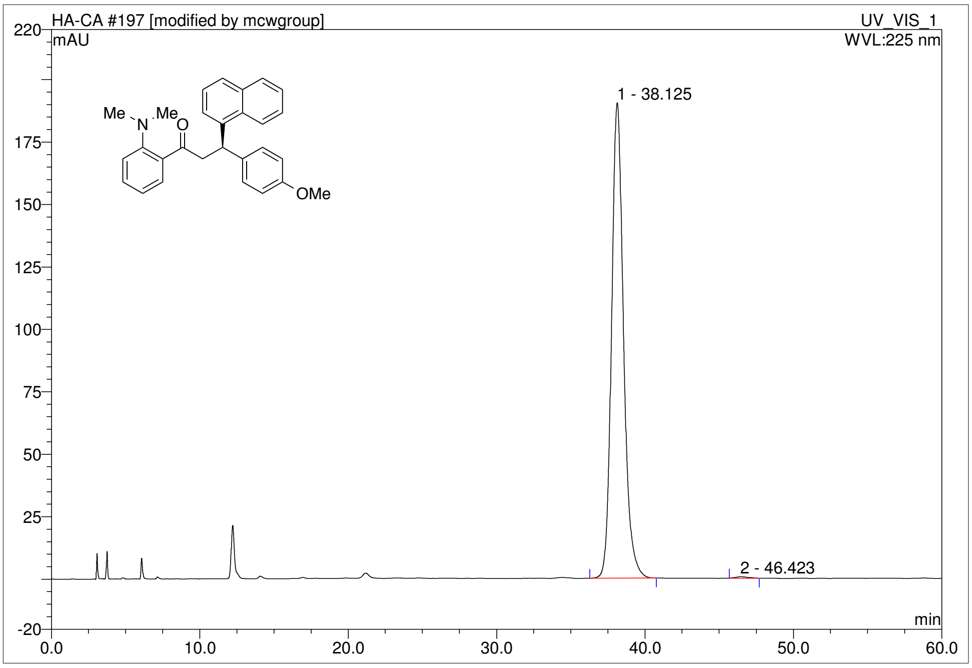

No.	Ret.Time min	Peak Name	Height mAU	Area mAU*min	Rel.Area %	Amount	Type
1	38.12	n.a.	190.340	177.906	99.70	n.a.	BMB*
2	46.42	n.a.	0.557	0.530	0.30	n.a.	BMB*
Total:			190.896	178.436	100.00	0.000	

Figure 84: HPLC chromatogram of compound **2ar**

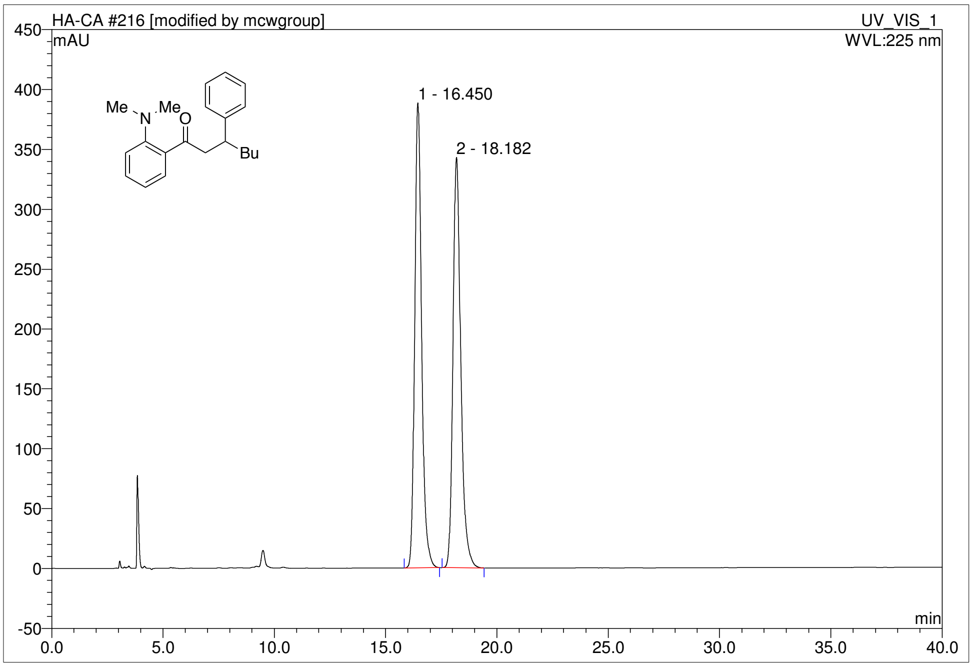

No.	Ret.Time min	Peak Name	Height mAU	Area mAU*min	Rel.Area %	Amount	Type
1	16.45	n.a.	388.474	139.382	49.96	n.a.	BMB*
2	18.18	n.a.	342.871	139.602	50.04	n.a.	BMB*
Total:			731.345	278.984	100.00	0.000	

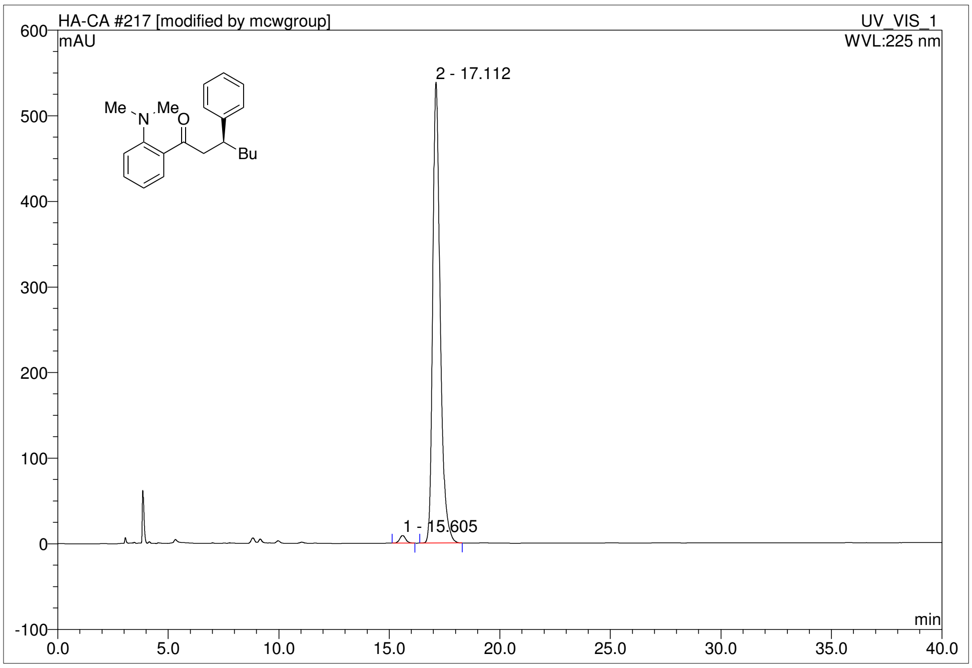

No.	Ret.Time min	Peak Name	Height mAU	Area mAU*min	Rel.Area %	Amount	Type
1	15.61	n.a.	8.932	2.868	1.34	n.a.	BMB*
2	17.11	n.a.	538.378	211.148	98.66	n.a.	BMB*
Total:			547.311	214.016	100.00	0.000	

Figure 85: HPLC chromatogram of compound **2as**

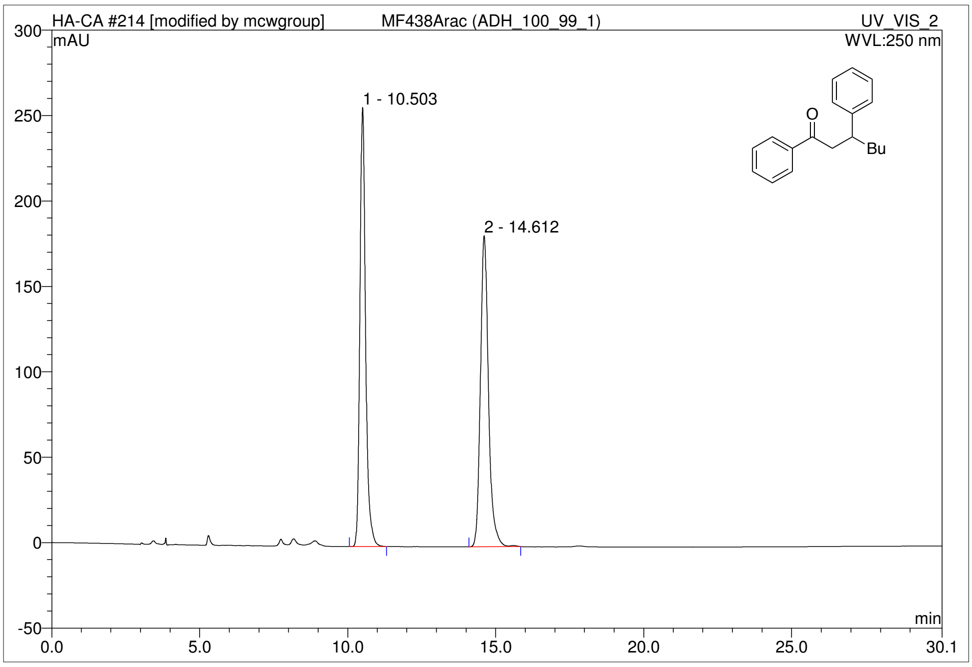

No.	Ret.Time<br>min	Peak Name	Height<br>mAU	Area<br>mAU*min	Rel.Area<br>%	Amount	Type
1	10.50	n.a.	256.978	57.378	49.90	n.a.	BMB*
2	14.61	n.a.	182.118	57.618	50.10	n.a.	BMB*
Total:			439.097	114.995	100.00	0.000	

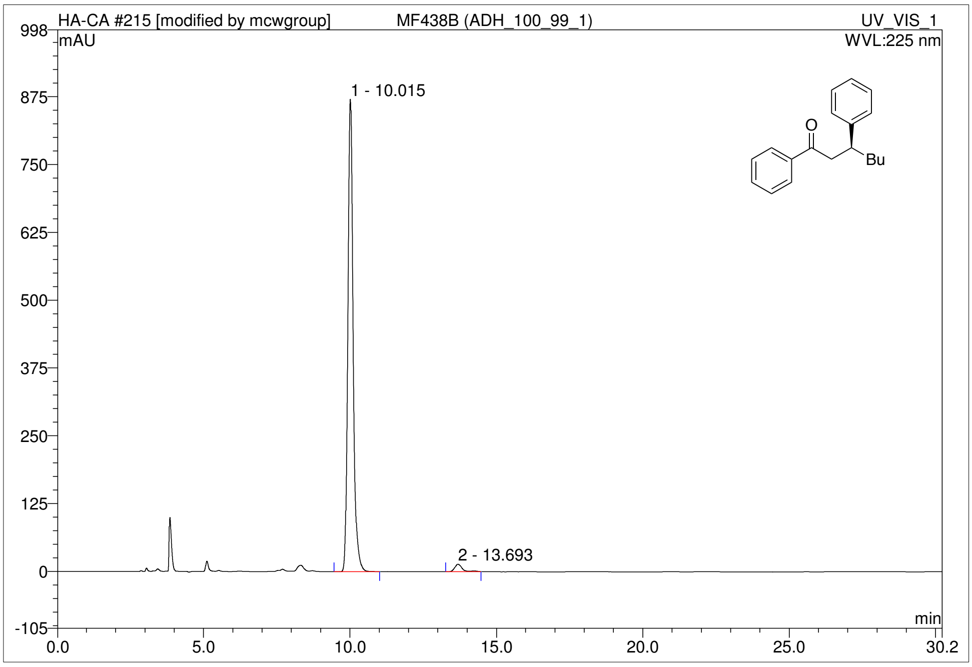

No.	Ret.Time<br>min	Peak Name	Height<br>mAU	Area<br>mAU*min	Rel.Area<br>%	Amount	Type
1	10.02	n.a.	870.798	180.567	97.74	n.a.	BMB*
2	13.69	n.a.	13.911	4.182	2.26	n.a.	BMB*
Total:			884.709	184.749	100.00	0.000	

Figure 86: HPLC chromatogram of compound **4as**

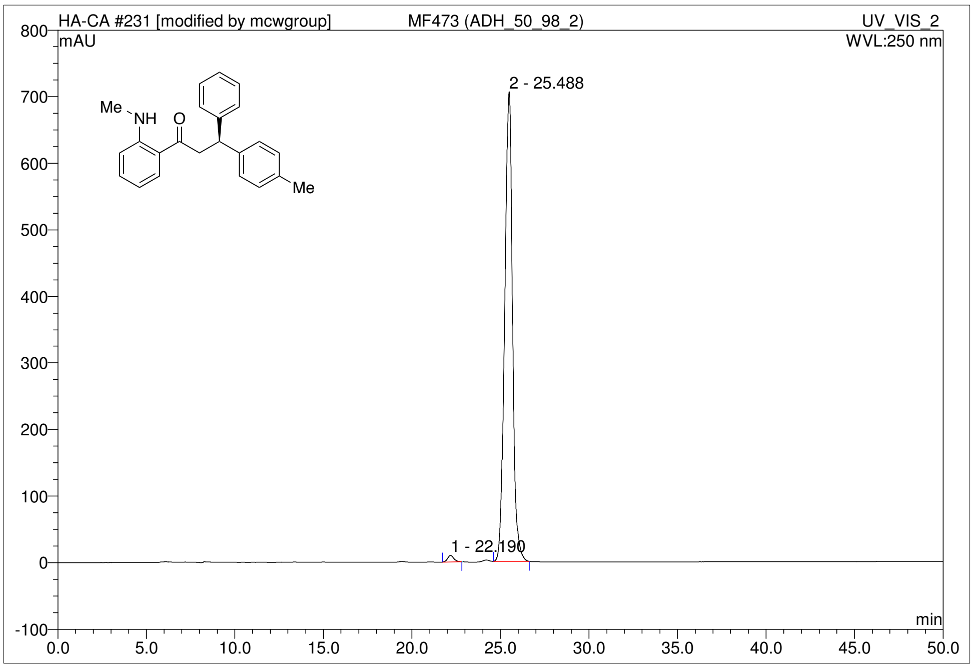

No.	Ret.Time min	Peak Name	Height mAU	Area mAU*min	Rel.Area %	Amount	Type
1	22.19	n.a.	9.611	3.885	1.07	n.a.	BMB*
2	25.49	n.a.	705.406	360.580	98.93	n.a.	BMB*
Total:			715.017	364.465	100.00	0.000	

Figure 87: HPLC chromatogram of compound **3ak**

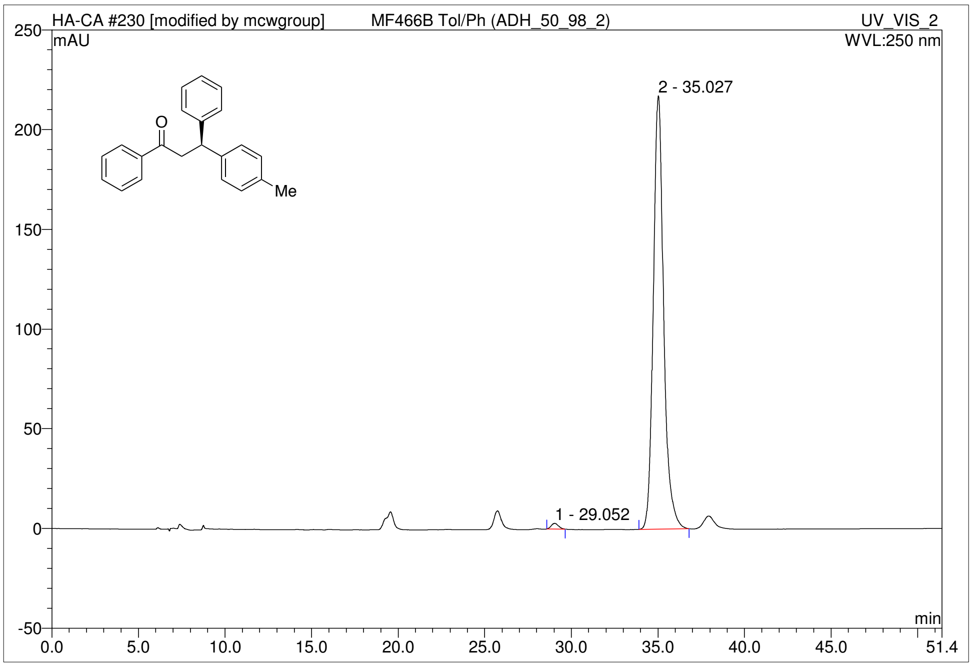

No.	Ret.Time min	Peak Name	Height mAU	Area mAU*min	Rel.Area %	Amount	Type
1	29.05	n.a.	2.814	1.349	0.89	n.a.	BMB*
2	35.03	n.a.	217.333	150.767	99.11	n.a.	BMB*
Total:			220.147	152.116	100.00	0.000	

Figure 88: HPLC chromatogram of compound **4ak**
